# Dynamic coupling enables impulsive ejection in a hydrogel catapult

**DOI:** 10.1038/s41467-026-77391-6

**Published:** 2026-09-07

**Authors:** Xin Wang, Neng Xia, Chengfeng Pan, Bo Hao, Chaoyu Yang, Xinyu Wu, Tiantian Xu, Qingsong Xu, Li Zhang

**Affiliations:** 1 Department of Mechanical and Automation Engineering, The Chinese University of Hong Kong, Hong Kong, SAR, P.R. China; 2 The State Key Laboratory of Fluid Power and Mechatronic Systems, College of Mechanical Engineering, Zhejiang University, Hangzhou, Zhejiang P.R. China; 3 Guangdong Provincial Key Laboratory of Robotics and Intelligent System, Shenzhen Institutes of Advanced Technology, Chinese Academy of Sciences, Shenzhen, P. R. China; 4 Department of Electromechanical Engineering, Faculty of Science and Technology, University of Macau, Macau, P.R. China; 5 CUHK T Stone Robotics Institute, The Chinese University of Hong Kong, Hong Kong, SAR, P.R. China; 6 Chow Yuk Ho Technology Center for Innovative Medicine, The Chinese University of Hong Kong, Hong Kong, SAR, P.R. China; 7 Department of Surgery, The Chinese University of Hong Kong, Hong Kong, SAR, P.R. China

**Keywords:** Gels and hydrogels, Mechanical engineering

## Abstract

Ejection dynamics can provide miniature robots with high-speed, cross-scale locomotion and non-contact operation. Here, we present a dynamic coupling ejection strategy for generating and transferring kinetic energy in miniature robotics. We demonstrate this strategy using a bilayer hydrogel catapult, in which rapid phase-transition-driven deformation couples dynamically with the projectile and the substrate, enabling substantial power output and kinetic energy release. The resulting ejection actuation system achieves a broadened projectile mass range (0-1000 times the catapult’s body weight), a launch velocity of 16.6 m/s for a same-mass projectile, and a power density ( > 8.74×10^4 ^W/kg) that surpassing most natural and engineered miniature catapults. As a proof of concept, we demonstrate a hydrogel catapult-driven biopsy robot that ejects a thick needle to acquire deep tissue samples in a single operation. These results establish a platform for advancing rapid motion and high-power output in miniature soft robotics.

## Abstract (web summary)

Researchers developed a hydrogel catapult that rapidly ejects projectiles across a wide weight range using dynamic coupling. The system also powered a biopsy robot capable of collecting deep tissue samples.

## Introduction

Ejection is a ubiquitous yet indispensable motion behavior for organisms at miniature scale in nature^1–3^, e.g., ballistic seeds/spores dispersal in plants/fungi for reproduction^4–7^, droplet super propulsion or catapult in insects for excretion^8,9^ and hunting^10,11^, respectively. Ejection motion differs from jumping in that it enables high-speed projectile launch while maintaining positional stability of the catapult^12,13^. In miniature robotics (millimeter to centimeter size)^14^, catapults are considered promising tools that can be integrated into robot systems to achieve efficient remote and non-contact operation in confined spaces^15^. The substantial force output and transmission during the ejection motion hold promising potentials for some specific robot-assisted medical procedures that demand high force (e.g., grasping, clamping, cutting, biopsy, and stent deployment require the exertion of substantial forces >1 N)^16–21^. At miniature scales, ejection motion relies on instantaneous high-power output, posing significant challenges for miniature robots due to their small body size and limited energy storage capacity^22–25^. The common strategy is based on power amplification (e.g., latch-mediated spring-actuated (LaMSA) system)^26–28^, which stores substantial potential energy in elastic structures then converting and transmitting it into the kinetic energy of projectiles, thus enabling high-speed ejection and significant power output^29–33^. Such capabilities are crucial for miniature robots to achieve rapid macroscopic displacement in confined spaces or to execute tasks requiring high-impact intensity and velocity (e.g., deep subcutaneous tissue penetration).

Tremendous progress has been made to achieve the ejection functions of miniature robots based on biomimetic design. Typical examples include a shooting manipulator that mimics chameleon tongue dynamics for prey capture^34^, and a microrobot pitcher achieving record ejection velocity (10.6 m/s) through mantis shrimp-inspired mechanics^35^. The utilization of rigid materials and complex mechanical structure design enables high performance through increased energy storage and a reduced energy release period. However, the rigid latch-spring system faces challenges in miniaturization due to the sophisticated integration process of multiple components (e.g., latch, spring, and motor) and safety concerns for interaction in biomedical applications^36^. Further development is the construction of an untethered catapult system based on stimuli-responsive materials. For example, the liquid crystal elastomer (LCE)-based catapults achieve near-infrared (NIR) light-controlled projectile ejection, effectively mimicking the spore ejection mechanism observed in fern sporangia^37,38^. However, soft materials and their corresponding energy accumulation-release strategies exhibit inherent limitations, including constrained energy storage capacity and limited initial momentum generation due to prolonged energy release durations, resulting in inefficient kinetic energy transfer and poor ejection performance (e.g., projectile weights are limited to the milligram level)^39,40^. These fundamental challenges have consequently prevented current catapults from demonstrating practical application potential. Therefore, it remains necessary to develop robotic devices and actuation mechanisms to improve the performance of the catapult for advancing the development of miniature soft robotics.

**Fig. 1:**
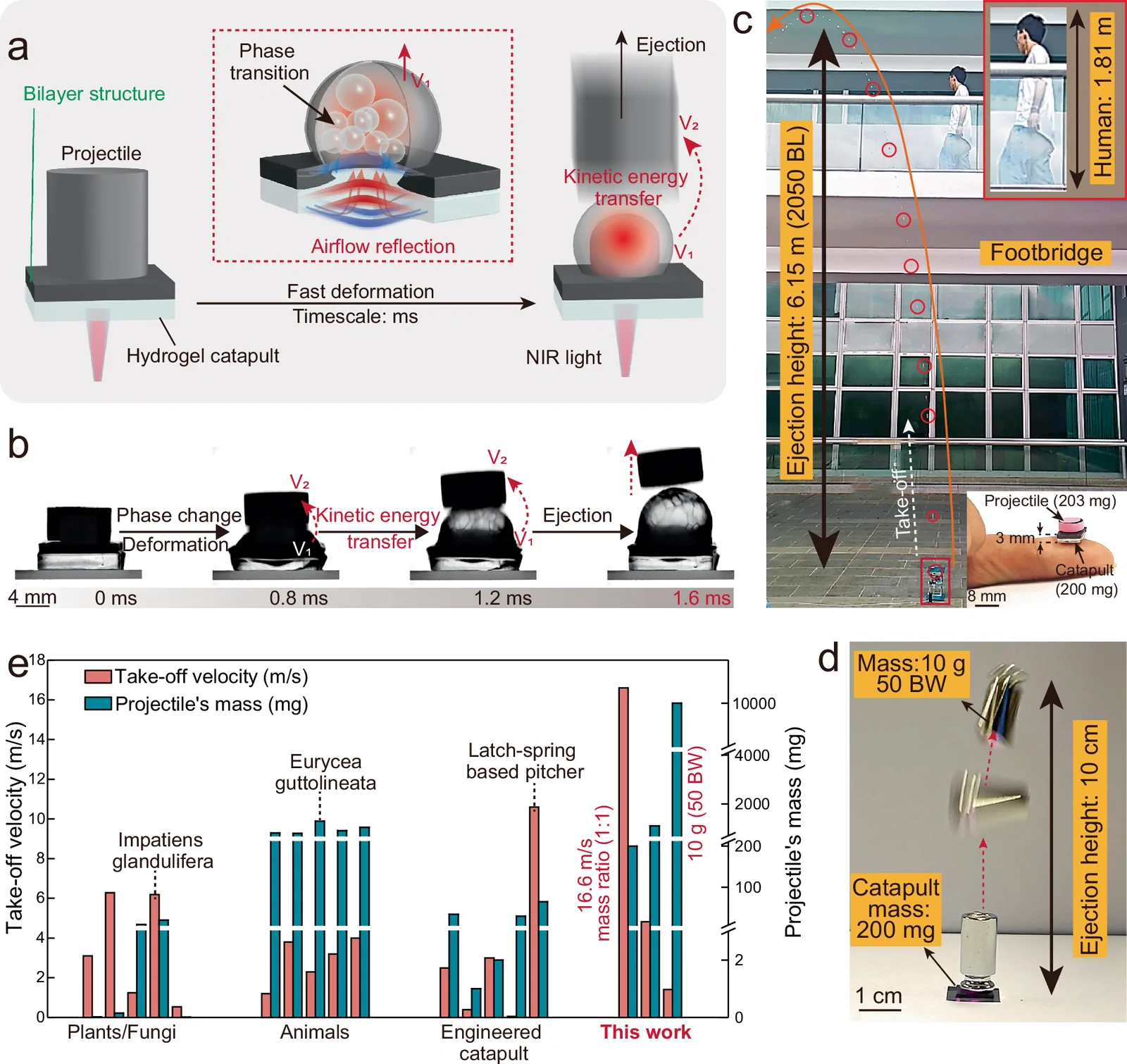
The ejection actuation mechanism of the hydrogel catapult. **a** Schematic illustration of the ejection actuation mechanism. **b** The high-speed camera images obtained from Supplementary Movie 1 show the ejection process of the hydrogel catapult. **c** An overlaid image obtained from Supplementary Movie 2 indicating the ejection height of the catapult actuated by NIR light. Inset: size comparison of the hydrogel catapult and projectile used in Fig. 1c, the mass of the projectile is 203 mg that is similar to the BW of the catapult (200 mg). **d** An overlaid image obtained from Supplementary Movie 2 indicating the ejection of the projectile with the mass of 10 g (i.e., 50 BW of the hydrogel catapult). **e** The take-off velocity and projectile’s mass comparison of the light-driven hydrogel catapult with engineered catapult, plants/fungi and animals capable of ejection motion in nature. Note that the data shown are limited to miniature organisms/engineered catapults with a weight in *mg-g* level. For representative images in Fig. 1 (high-speed camera snapshots and overlaid trajectories), experiments were independently repeated at least three times with similar results, and representative data are shown.

Here, we developed a dynamic coupling strategy for actuating ejection motion in miniature robotics. This strategy relies on phase change-induced bilayer structure separation and dynamic coupling interactions to generate and transfer substantial initial momentum (Fig. 1a, b). We demonstrate this principle through a hydrogel catapult, which achieves the broadest reported projectile mass range (0–1000 times its body weight (BW)) and record-breaking ejection performance (i.e., launching height is 6.15 m (2050 times body length (BL)), initial velocity and take-off acceleration is 16.6 m/s and 1.04 × 10^4^ m/s^2^ for a payload matching its self-weight, respectively) of any miniature engineered robotic devices and most natural ejection systems (Fig. 1c–e, Supplementary Tables 1 and 2, and Supplementary Fig. 13). In addition, the hydrogel catapult also delivers a high instantaneous power output (8.74 × 10^4^ W/kg), exceeding values reported for most natural and engineered miniature catapults. This high output is enabled by rapid deformation (~1.6 ms) and dynamic coupling among the hydrogel catapult, the substrate and the projectiles. By constructing an asymmetric interaction between the hydrogel catapult and projectiles, controllable directional ejection motion is realized. As a proof of concept, we developed a hydrogel catapult-actuated biopsy robot that ejects a needle in a single operation to acquire sufficient deep tissue samples. The explored dynamic coupling mechanism and the demonstrated ejection performance provide a basis for developing miniature soft robots with impulsive motion capabilities and high power/force output in confined environments.

## The ejection actuation mechanism of the hydrogel catapult

The developed ejection actuation strategy is conceptually related to sporangium dispersal in *Sphaerobolus*, a fungus with a separable multilayer elastic membrane structure that enables rapid delamination and deformation-mediated momentum transfer for long-distance seed dispersal^41,42^ (Supplementary Fig. 14). Motivated by these structural and dynamical features, we developed a hydrogel catapult and an associated actuation strategy capable of generating high force and power output for miniature robotic systems. Specifically, the hydrogel catapult is designed with a bilayer architecture analogous to the separable multilayer membrane in the fungal system (Supplementary Fig. 15), the upper layer is composed of high-toughness hydrogel (Poly(acrylic acid-co-acrylamide); P(AA-co-AM)) doped with graphene. The bottom layer is a pristine, transparent hydrogel serving as the constraint to control the deformation direction towards the projectile. As shown in Fig. 1a, the projectile is placed on the upper surface of the hydrogel catapult, when irradiated with NIR light from the bottom surface, the transparent pristine hydrogel allows the NIR light across and absorbed by graphene. This leads to rapid temperature increase (exceeding 120 °C within 2 s) in the upper layer of the irradiated area due to the photothermal effect of the doped graphene (Supplementary Fig. 16), the graphene acts as both photothermal converter and an optical barrier, thereby inducing the heat production occurred inside the hydrogel matrix. Note that the graphene-enabled rapid heat production is only a representative example, any other material system or trigger method capable of inducing a localized and rapid heat production inside the hydrogel can be employed (Supplementary Note 1). Continued irradiation of NIR light further induces the phase transition of the water (i.e., from liquid to vapor) inside the hydrogel network rapidly (Fig. 1b and Supplementary Fig. 17). This phase transition process has been demonstrated to achieve high-speed self-actuation of hydrogel devices, as pioneered by ref. ^43^. As a physical process rather than a chemical reaction, no chemical bonds are formed or broken during this transition (Supplementary Fig. 18). In addition, the jumping and ejection represent two fundamentally distinct modes of motion, ejection require more complex and difficult energy transfer process, we adopted this phase change triggering method to explore potential coupling principles with interfaces and projectiles, thereby achieving the high power output of the catapult. Specifically, the rapid steam expansion drives the highly stretchable and tough hydrogel matrix to undergo fast, drastic deformation, generating substantial initial momentum that is subsequently transferred to the projectile to activate the ejection process. Crucially, this deformation is directionally guided toward the projectile by introducing an asymmetric thickness profile. The constraint layer at the bottom of the catapult is deliberately designed to be thicker than the upper layer near the phase change area (i.e., the thinner upper surface provides less resistance to expansion), thereby ensuring targeted energy focus (Supplementary Fig. 19). The kinetic energy transfer and ejection arise from the dynamic coupling among three elements: phase change-induced hydrogel deformation, the substrate and the projectile. As shown in Fig. 1a, b, the hydrogel catapult is placed on a rigid substrate (e.g., glass plate) to maximize ejection performance. Upon phase transition, airflow is generated at the interface and impacts the elastic hydrogel symmetrically from the NIR irradiation center. In the vertical direction, the upward airflow first induces deformation of the upper-layer hydrogel network. The downward airflow-driven deformation compresses the transparent bottom layer beneath the phase change center. However, the combination of the thicker bottom layer and the rigid substrate (a non-deformable glass slide), secured by interfacial adhesion, forms a strong constraint that blocks downward vapor expansion and redirects the airflow upward, where the thinner upper layer offers the less resistance (Supplementary Fig. 20). Consequently, the combined effect of the initial upward airflow and the redirected downward airflow results in rapid upward deformation of the hydrogel catapult, generating the initial momentum for ejection (Fig. 1a). The thickness of the bottom layer plays an important role in controlling the deformation direction, maintaining the stability of the catapult, and inducing airflow rebound, the optimal thickness ratio of the upper layer to the bottom layer is 1:1.2 (Method and Supplementary Fig. 21). The phase transition-induced deformation is completed within 1.6 ms (Fig. 1b), generating a peak force output ~2.34 N (Supplementary Fig. 22). During this process, the projectile’s gravity disperses the internal pressure within the deformed hydrogel network, delaying fracture and increasing overall deformation. Notably, the projectile acquires its momentum before the deformation reaches its maximum. We define this ~1.6 ms interaction period (i.e., from initial contact to complete projectile detachment) as the actuation time (Fig. 1b and Supplementary Movie 1). After the projectile detached with a hydrogel catapult, the pressure accumulated inside the deformation volume reached the limit of the hydrogel network as the light irradiation induced continuous water vaporization, resulting in an instantaneous energy release through fracture (Supplementary Fig. 23). In addition, we have developed a model to simulate the deformation and the stored energy before fracture, details can be found in Supplementary Note 2. Note that the fracture is not a necessary condition for ejection. Because the projectile detaches from the hydrogel catapult before fracture occurs (Fig. 1b). The essential factor is the rapid deformation to generate sufficient initial momentum. In this study, the phase-transition-induced rapid-deformation hydrogel catapult merely serves as a convenient and representative example of such an ejection method. In addition, high-performance ejection motion is not achieved by modifying the phase transition mechanism itself or materials system design, but rather by the dynamic coupling strategy during the actuation, which is the key to unlocking the full potential of phase transition-driven soft actuators. In principle, any actuators capable of producing similarly fast and large deformations could leverage the coupling relationships explored in this work to achieve high-force ejection output.

The initial momentum generation and kinetic energy transfer enable the hydrogel catapult to achieve high ejection performance in terms of take-off velocity (exhibit as ejection height). As shown in Fig. 1c and Supplementary Movie 2, a representative ejection event propelled a projectile with a weight similar to the BW of the hydrogel catapult over 6.15 m (2050 times BL) in anti-gravity direction. The take-off velocity of the projectile can be calculated from the recorded launching height (*h*) by considering the post-take-off motion of projectile until reaching the maximum launching height as a deceleration motion driven by the gravity and the air drag force^44^:

1 $${v}_{{{\mathrm{take}}}-{{\mathrm{off}}}}=\sqrt{\frac{\left[{e}^{\frac{h{C}_{d}\rho \pi {r}_{0}^{2}}{m}}-1\right]\times 2{{\mathrm{mg}}}}{{C}_{d}\rho \pi {r}_{0}^{2}}}\,$$

Where *C*_*d*_ is the drag coefficient (0.77)^45^, *ρ* is the density of air, *r*_*0*_ is the basal radius of cylindrical projectile (*r*_*0*_ = 4 mm), *m* is the mass of the projectile (i.e., 203 mg) and *g* is the gravitational acceleration. For the ejection event shown in Fig. 1c, the initial velocity was calculated to be 16.6 m/s. Considering the fast actuation process (i.e., 1.6 ms), the ejection acceleration was calculated to be 1.04 × 10^4^ m/s^2^. These results demonstrate the hydrogel catapult’s potential to enable rapid locomotion in miniature robots. In addition, the hydrogel catapult can eject projectiles across a broad mass range. As shown in Fig. 1d, a representative ejection event for a heavy projectile with a 10 g payload (50 times the catapult’s own weight) being launched to heights exceeding 10 cm (Supplementary Movie 2). Also, a 200 mg catapult can topple a 200 g weight (1000 times the catapult’s own weight) actuated by NIR light as shown in Supplementary Fig. 24, highlighting the substantial force output of the current actuation strategy and high ejection performance of the developed hydrogel catapult. We compared the ejection performance of the hydrogel catapult with other engineered and natural system (For plants/fungi with ejection motion, e.g., explosive seed dispersal of the plants and spores ejection of fungi. For animals with ejection motion, e.g., chameleon use tongue to prey) in terms of projectile’s take-off velocity and mass, and power output as shown in Fig. 1e and Supplementary Fig. 13. The results demonstrate that the developed actuation strategy enables the light-driven hydrogel catapult to achieve high take-off velocity and power density, exceeding those reported for miniature engineered robotic devices with ejection capability and for most natural ejection systems.

## Dynamic coupling influences the ejection performance

**Fig. 2:**
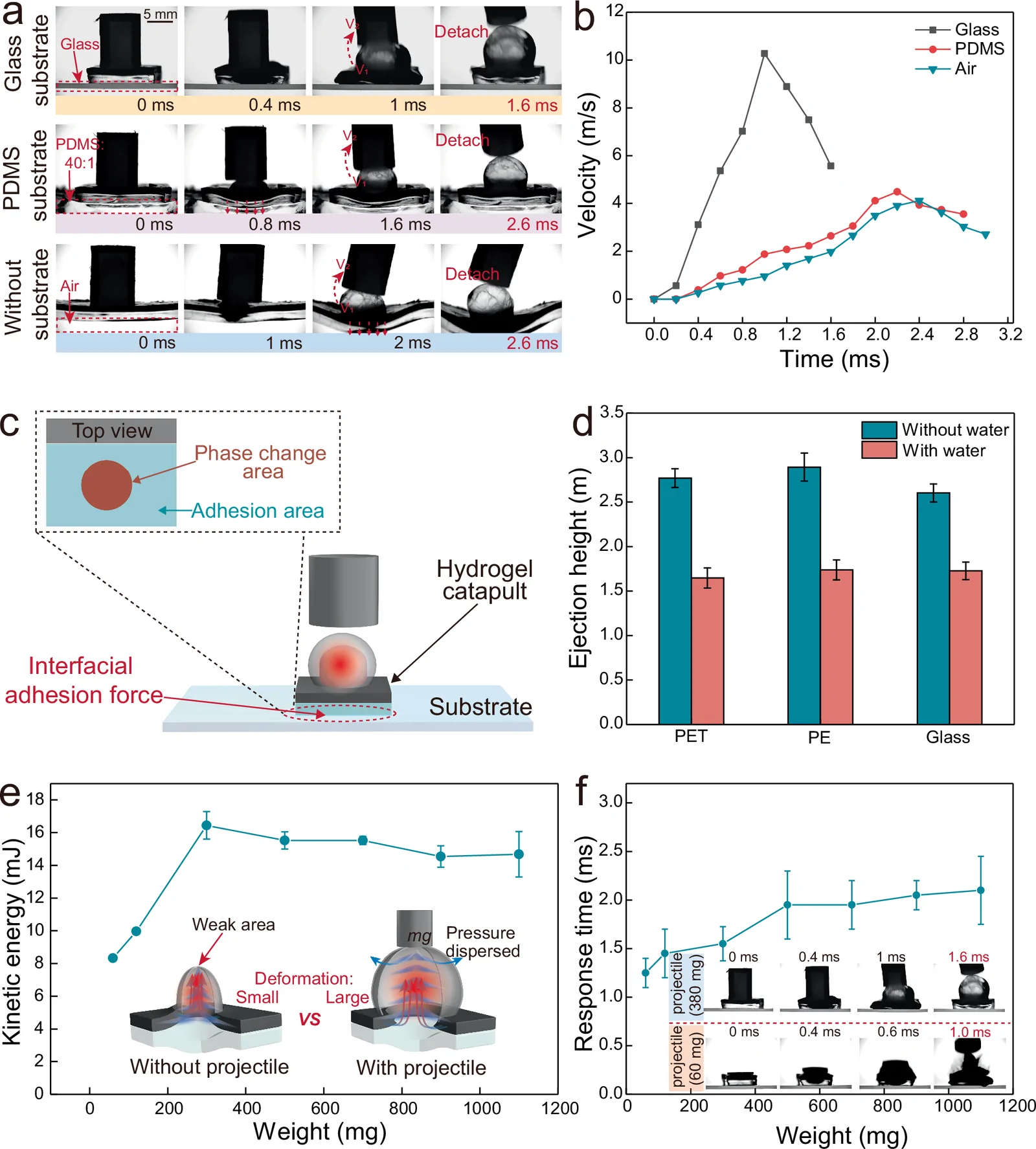
Factors influencing the ejection performance of the hydrogel catapult. **a** The deformation and actuation time comparison for hydrogel catapults on glass substrate, PDMS substrate and free-standing (without substrate). Note the weight of the projectile is 380 mg. **b** Projectile velocity profiles during actuation on the substrate of glass, PDMS, and free-standing. **c** Schematic of interfacial adhesion force between hydrogel catapult and substrate. **d** Ejection height comparison with/without water lubrication (blocks interfacial adhesion) across different substrates. Data points are shown as mean ± s.d. (*n* = 4). **e** The kinetic energy acquisition of the projectiles with different weights and **f** actuation time of the hydrogel catapult. Note that all the projectiles are fabricated in cylindrical (diameter is 10 mm, height is 10 mm) with different densities to reduce measurement errors. Data points are shown as mean ± s.d. (*n* = 4). For representative images in Fig. 2 (high-speed camera snapshots), experiments were independently repeated at least three times with similar results, and representative data were shown.

We conducted a detailed dynamic analysis of the factors affecting ejection performance. The material composition, geometry, and deformability of the hydrogel, as well as the external energy input, significantly influence the intensity and velocity of deformation, thereby affecting the momentum generation process. We have systematically investigated these factors on the deformation and ejection motion performance of the catapult, with detailed results provided in Supplementary Note 3. Effective ejection motion and output rely on the dynamic coupling between the deformation induced momentum generation, the substrate and launched projectiles. We focus on these dynamic interactions (i.e., stiffness of substrate, interfacial adhesion force, and projectile weight) to optimize the ejection actuation system’s output performance. First, the ejection motion of the hydrogel catapult is partially determined by the produced downward airflow rebound to achieve deformation and power output (Fig. 1a), therefore, the stiffness of the substrate significantly influences the ejection performance. We compared the ejection dynamics of hydrogel catapult on three representative substrates: rigid substrate (glass), flexible substrate (PDMS with the ratio of monomer to curing agent is 40:1), and free-standing (i.e., suspending the hydrogel catapult in air, as shown in Fig. 2a and Supplementary Fig. 25). We used a high-speed camera to capture the actuation dynamics and momentum transfer (Supplementary Movie 3). As shown in Fig. 2a, b, compared with the elastic substrate and no substrate, the rigid substrate enabled the hydrogel catapult to achieve larger deformation (Fig. 2a) and faster initial momentum transfer to the projectile during the actuation process (~1.6 ms *vs* ~2.6 ms). The potential reason is that the rigid substrate minimizes the energy dissipation due to elastic deformation of the substrate (Supplementary Fig. 26), allowing the reflected airflow inside the phase transition volume acquire greater upward impact speed, thus contributing to the overall deformation speed of the hydrogel network and initial kinetic energy generation (Supplementary Fig. 27). In addition, the obvious oscillation and buffering effect in elastic/free-standing configurations lead to considerable energy dissipation, thus reducing the kinetic energy output (Fig. 2b and Supplementary Fig. 28). Furthermore, the mathematical analysis presented in Supplementary Note 4 is consistent well with experimental results, confirming that the rigid substrate enhances the projectile’s initial momentum acquisition.

The interfacial adhesion force between the hydrogel catapult and substrate significantly influences ejection performance. This adhesion stabilizes the catapult against bidirectional deformation impacts from internal phase changes. Insufficient interfacial adhesion leads to oscillation and unstable deformation of the hydrogel catapult (Supplementary Fig. 29). To prevent the catapult from displacing during actuation process, the area of the catapult should be larger than the phase change region (Fig. 2c and Supplementary Fig. 30). In addition, we compared the ejection performance of the catapult when the adhesion force was changed on different surfaces (i.e., glass surface, polyethylene terephthalate (PET), and polyethylene (PE) surface), where water was used as lubricant between the catapult and contact surface to prevent the formation of hydrogen bonds that significantly reduce adhesion force (Supplementary Fig. 31). As shown in Fig. 2d, the ejection performance of the hydrogel catapult under high adhesion condition (i.e., without water as lubricants) is much higher than that under low adhesion force condition (i.e., with water as lubricants). A potential reason is that the interfacial adhesion reduces the kinetic energy loss caused by hydrogel displacement and reduces the rebound airflow dispersion, thereby improving initial kinetic energy generation and momentum transfer. Furthermore, the surface roughness alters the interfacial adhesion by changing the contact area between the hydrogel catapult and the substrate. A rougher surface reduces the contact area, thereby decreasing adhesion and altering the ejection performance (Supplementary Fig. 32).

In addition to the stiffness of the substrate and the interfacial adhesion force, the weight of the projectile placed above the deformation zone affects the internal pressure distribution during hydrogel deformation, thus altering the interaction period and momentum transfer. We prepared cylindrical projectiles of constant dimensions (radius and height is 5 and 10 mm, respectively) with varying masses to investigate the influence of the projectile’s weight on the ejection performance. As shown in Supplementary Fig. 33, the ejection height first increases and then decreases with increasing projectile weight. Based on the recorded ejection heights of projectiles with different weights, we further calculated the kinetic energy of these projectiles, accounting for aerodynamic drag. As the mass increases, the kinetic energy transferred to the projectile initially rises and then reaches a plateau (Fig. 2e). We conducted the analysis of the actuation process for projectiles with different weights. Specifically, during the phase transition, upward airflow exerts a dynamic impact on the hydrogel network, inducing substantial structural deformation. The gravity of the projectile disperses the impact pressure that was originally concentrated on the weakest point of the deformed area, thereby delaying the fracture time and increasing the overall deformation. The impulse (*I*) acting on the projectile can be quantified through the integral formulation $I=\int {Fdt}$. Where *F* is the dynamic force produced by the phase transition induced fast deformation, *t* is the interaction period. Assuming the hydrogel is homogeneous and an equal volume of water undergoes phase transition and then induces the deformation, the initial-stage deformation rate and force output of the catapults are similar. Therefore, the weight of the heavier projectiles delays the fracture of the hydrogel network and extends the interaction duration (e.g., 1.0 ms for a 60 mg projectile *vs* 1.6 ms for a 380 mg projectile, as shown in Fig. 2f-inset), thus enhancing momentum transfer. Consequently, this process yields a higher initial projectile velocity and improved ejection performance. Furthermore, we performed a theoretical analysis using a mathematical model (Supplementary Note 5), and the results exhibited strong agreement with our experimental findings. Note that as the weight of the projectile continues to increase (e.g., >400 mg), more energy is required to overcome the gravity of the projectile after launching, which induced the decreased ejection height accordingly (Supplementary Fig. 33). For a 200 mg hydrogel catapult, the highest ejection height is achieved when the projectile weight is 1–2 times its BW.

## Ejection performance of the hydrogel catapult with wide applicability

**Fig. 3:**
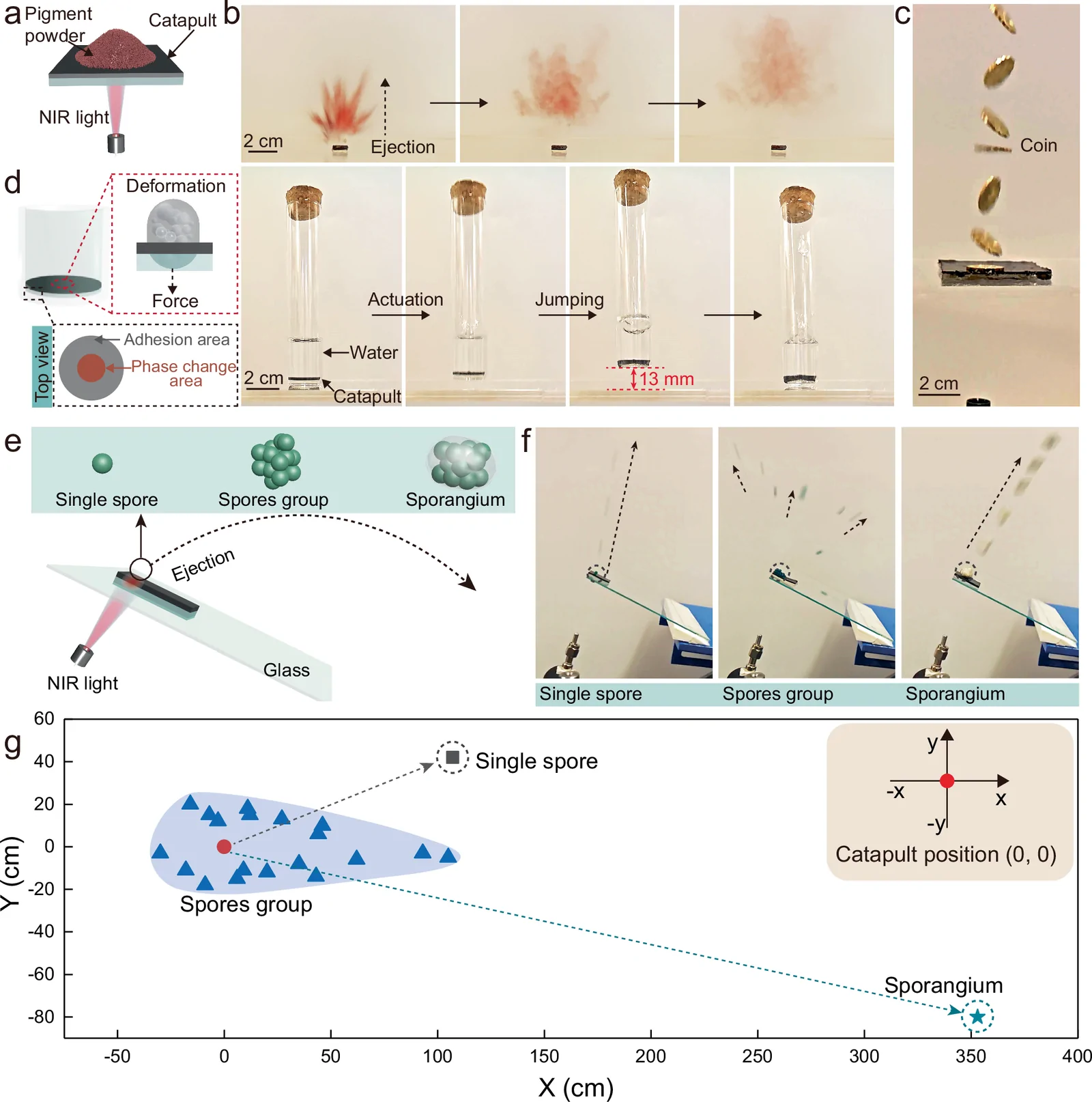
Ejection performance of the hydrogel catapult with diverse projectiles. **a** Schematic and **b** snapshots obtained from Supplementary Movie 4 showing the powder ejection process. **c** Overlaid images obtained from Supplementary Movie 4 showing the coin ejection process. **d** Schematic illustration and snapshots obtained from Supplementary Movie 4 showing hydrogel catapult as the actuator to drive the burette jump. **e** Schematic of a physical model representing sporangium-like ejection behavior. **f** High-speed images illustrate the ejection motion of the simulated projectiles (i.e., single spore, spore cluster and sporangium). **g** Location distribution for three representative ejection events. For representative images in Fig. 3 (photographs and overlaid trajectories), experiments were independently repeated at least three times with similar results, and representative data were shown.

The hydrogel catapult exhibits wide applicability for different projectiles due to its notable momentum transfer and force output. As shown in Fig. 3a and Supplementary Movie 4, the pigment powder serving as a representative example of the lightweight projectiles is deposited on the upper surface of the hydrogel catapult. The catapult ejects the powders at high speed through NIR light stimulation from the bottom surface (Fig. 3b), the actuation force originates from the synergetic interactions of rapid elastic deformation and fracture induced upward vapor jetting. In contrast, for the ejection of heavier projectiles (such as the coin shown in Fig. 3c and Supplementary Movie 4), the actuation force mainly originates from the force output caused by phase transition-induced kinetic energy transfer. The distinct actuation dynamics between lightweight and heavy projectiles highlight the wide adaptability of the developed ejection actuation system. In addition, by combining the adhesion force and an applied constraint on its upper surface, the hydrogel catapult can function as an actuator to actuate the system jump (Fig. 3d). As a proof of concept, the hydrogel catapult is placed on the bottom of a burette. The hydrogel exhibits considerable adhesion force with glass due to the hydrogen bond interactions between the functional groups, combined with the infused water on the upper surface of the catapult as a constraint to increase the downwards deformation for impacting the table. Upon NIR light actuation from the bottom surface, the increased downwards deformation hits the bottom of the burette and generates an upward reaction force which is sufficient to propel the entire system (the total mass is 20.84 g, 104.2 times the weight of the hydrogel catapult) jump to a height of 13 mm (Supplementary Movie 4). Note that the system maintains structural integrity during actuation due to the interfacial adhesion and the constraint provided by the water’s weight (Fig. 3d).

In addition, we used the hydrogel catapult as a physical model to examine how encapsulation influences ejection performance in a *Sphaerobolus*-like dispersal system. Specifically, we examined why the ejected seeds are encapsulated sporangia rather than individual or aggregated spores. For systematic comparison, we simulated three ejection scenarios using 1 mm radius hydrogel spheres (Fig. 3e): (1) a single hydrogel sphere (representing individual spores), (2) an unencapsulated aggregate (a cluster of 20 hydrogel spheres), and (3) a sporangium analog (20 hydrogel spheres encapsulated in an elastic film). As shown in Fig. 3f, all projectiles were launched at high velocities. Representative landing statistics reveal that the sporangium analog achieved the longest ejection distance (Fig. 3g). These results align with mathematical analysis (Supplementary Note 6), which may help to explain the dispersal strategy observed in *Sphaerobolus*, namely that reproduction through the ejection of the sporangium, despite the increased energy expenditure, offers a longer propagation distance and an enhanced spore survival rate. The above demonstration validates the excellent ejection performance of the catapult enabled by the dynamic coupling framework. It not only exhibits wide applicability for different projectiles but also allows the catapult size to be adjusted according to the dimensions of the ejected objects. We have achieved a minimal hydrogel catapult (1 × 1 × 0.8 mm) along with high-speed ejection behavior (Supplementary Fig. 34), thereby proving the broad adaptability and size-independence of the proposed dynamic coupling framework.

Furthermore, benefiting from the powerful force output, the hydrogel catapult can be used not only in the vertical direction but also in the horizontal direction, which is visually demonstrated by the ejection of iron balls with different volumes and masses as shown in Supplementary Fig. 35. The iron balls with different masses are ejected at different speeds and to different distances, the smaller the mass of the ball, the faster it is ejected and the longer the distance it travels. Surprisingly, a 200 mg catapult can eject a ball of ten times its self-weight (i.e., 2 g) and a ball of 600 times its self-weight (i.e., 120 g) over 1 and 0.1 m, respectively, confirming the force output and wide application potential in miniature soft robotics (Supplementary Movie 4).

## The ejection motion control of the hydrogel catapult

**Fig. 4:**
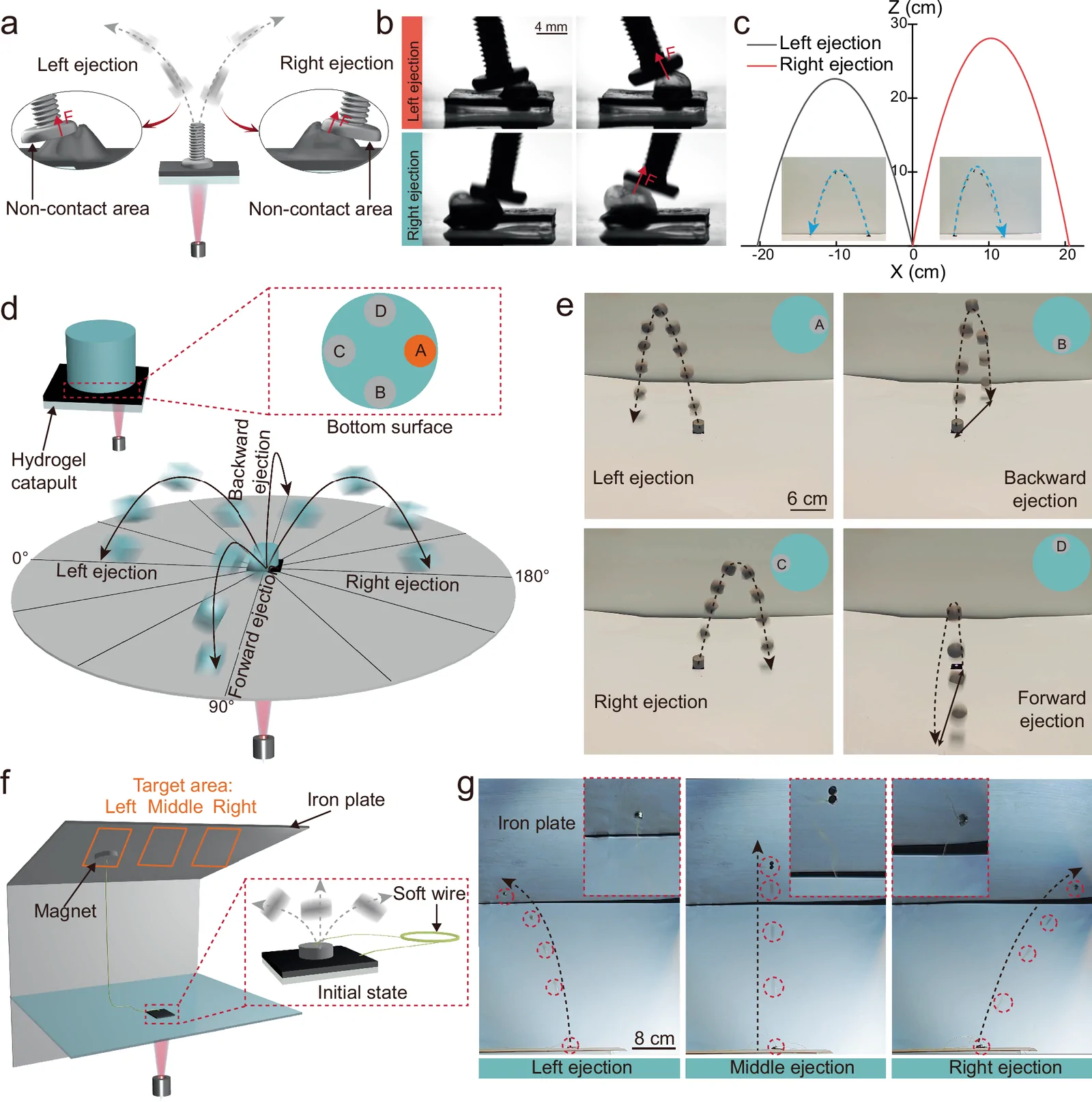
Directional control of the ejection motion of the hydrogel catapult. **a** Schematic illustration showing the directional control principle of the ejection motion. **b** The high-speed images in the activation moment and **c** launching trajectory obtained from Supplementary Movie 5 showing left and right ejection motion. Note that the light intensity and projectile is 15.68 W/cm^2^ and 2280 mg, respectively. **d** Schematic illustration and **e** overlaid images obtained from Supplementary Movie 5 showing the multidirectional ejection capability. **f** Schematic and **g** trajectory analysis of magnet ejection to a predefined target area on an iron plate. For representative images in Fig. 4 (high-speed camera snapshots and overlaid trajectories), experiments were independently repeated at least three times with similar results, and representative data were shown.

In addition to the vertical and horizontal ejection capabilities of the hydrogel catapult, directional ejection motion can be realized by tailoring the position at which the output force is applied to the projectile, i.e., the deformation region relative to the projectile’s bottom surface. Specifically, the projectile can be launched in different directions, depending on the controllable action position of the deformation-generated force on its bottom surface. By controlling the NIR light activation area to construct partial interaction with the projectile’s bottom surface, as shown in Fig. 4a–c, the projectile is ejected to the right when the interaction occurs on the left side of its bottom surface. Likewise, leftward directional ejection motion can be realized when the deformation contact occurs on the right side of the projectile’s bottom surface (Supplementary Movie 5). The asymmetric force production from a relatively eccentric position leads to the same magnitude but different directions of the take-off velocity as evidenced by high-speed camera images at the activation moment (Fig. 4b), thus inducing the directional ejection motion with similar performance (i.e., similar launching height and horizontal displacements) (Fig. 4c). This control mechanism extends to multidirectional ejection capabilities through programmable NIR activations positions (A–D in Fig. 4d). The resulting actuation force enables precise ejection along the four cardinal directions (left, right, forward, and backward) while maintaining uniform ejection height and travel distance (Fig. 4e and Supplementary Movie 5). The current directional control method exhibits high repeatability and robustness. Moreover, the property of substate shows limited influence on the direction control (Supplementary Fig. 36).

Furthermore, various biological motion strategies can inform engineering design. We approximately simulated the target tongue ejection process of a chameleon based on the proposed directional ejection strategy. As shown in Fig. 4f, a magnet was connected to the hydrogel catapult via a soft wire to simulate the chameleon’s extensible tongue. An iron plate with three pre-designed areas (i.e., left, middle and right areas shown in Fig. 4f) was fixed above so that the magnet could be attached to simulate the hunting behavior of a chameleon. The magnet can be ejected at high speed by changing the NIR light activation position of the hydrogel catapult, which finally absorbed to the target area on the iron plate (Fig. 4e and Supplementary Movie 5). This tethered directional ejection strategy could potentially be applied to long-distance sampling and adhesion in miniature robotics.

## Hydrogel catapult-based biopsy robot for deep tissue sampling

**Fig. 5:**
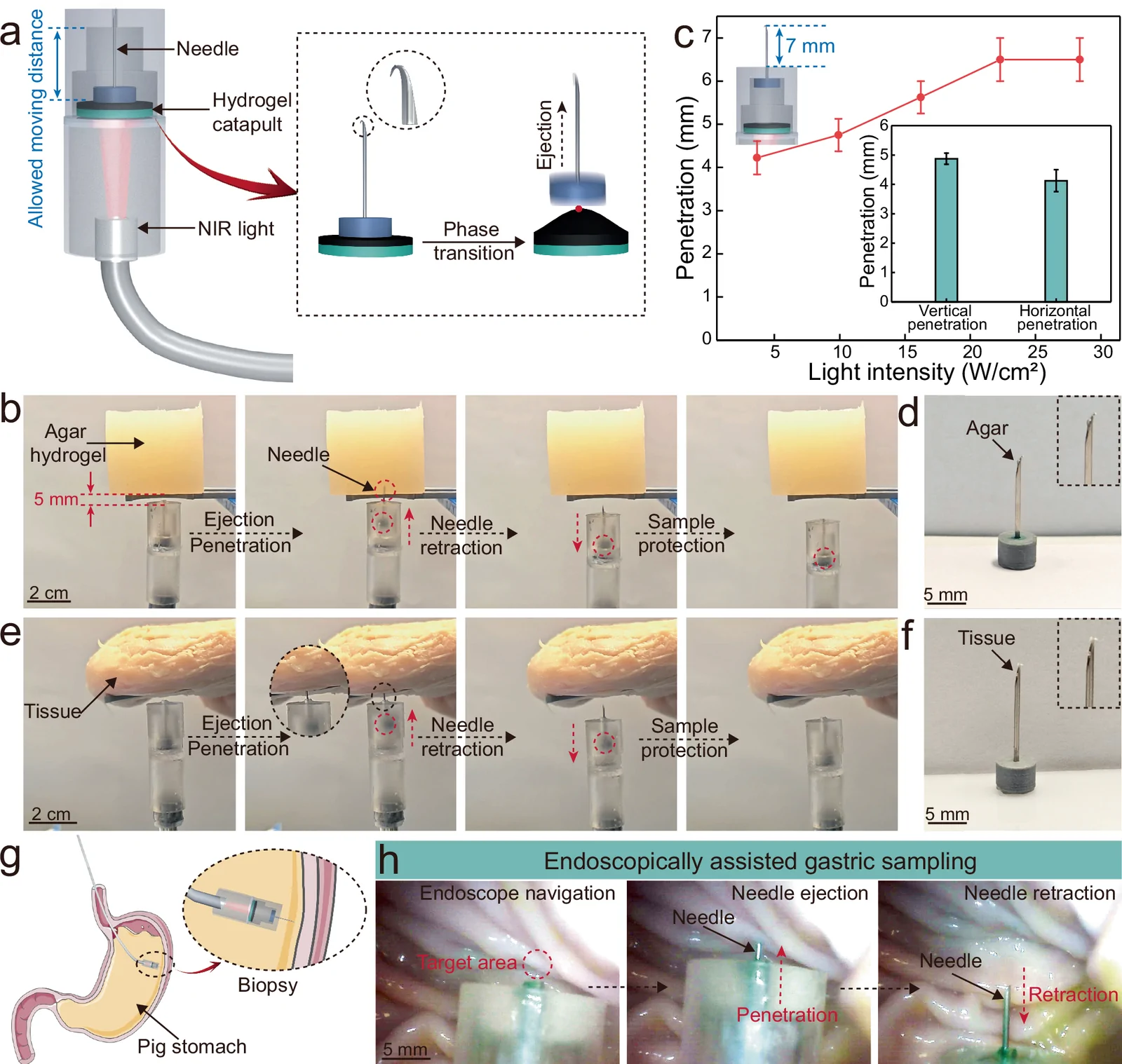
Hydrogel catapult-actuated biopsy robot for gastric tissue sampling. **a** Schematic illustration of the structure and working principle of the developed biopsy robot. **b** A series of snapshots obtained from Supplementary Movie 6 illustrating the penetration process into tissue-like material (i.e., 5 wt% agarose gel). **c** The penetration depth in agarose gel with different light intensity. Inset: the comparison of the vertical and horizontal penetration depth. Data points are shown as mean ± s.d. (*n* = 4). **d** The image of the pulled-out needle from agarose showing the successful sampling. **e** A series of snapshots obtained from Supplementary Movie 6 and **f** image of the needle after actuation indicating the successful sampling process. **g** Schematic illustration and **h** a series of snapshots obtained from Supplementary Movie 7 showing the biopsy process inside the pig’s stomach. For representative images in Fig. 5 (photographs), technical repeated experiments (i.e., use one agarose gel, tissue and porcine stomachs) were independently carried at least three times with similar results, and representative data are shown.

Biopsy is crucial for precision medicine, early disease detection and patient stage monitoring. Currently, fine-needle aspiration biopsy (FNAB) and fine-needle capillary biopsy (FNCB) are widely employed for subsurface tissue sampling in endoscopic procedures^16,46^. However, due to the limited capacity of the fine needle, multiple punctures (usually 3–7 passes) are often required to obtain sufficient diagnostic material^47^, which increases procedure time, patient discomfort, and the risk of complications^48^. For regions that are difficult to access directly, such as gastric fundus sampling, the endoscope must be retroflexed^49^, and the puncture force is transmitted from the operator’s hand through the entire length of the endoscope to its tip, resulting in insufficient force or inaccurate puncture. Therefore, using a thicker needle to obtain sufficient samples and generating a driving force at the tip are beneficial for reducing the need for repeated surgical maneuvers and improving precision. The kinetic energy transfer and force output enable the hydrogel catapult to be potentially suitable for the actuation of deep tissue biopsy robots. As a proof of concept, we developed a hydrogel catapult-based biopsy system, as shown in Fig. 5a. The system incorporates a sampling needle with a larger diameter than traditional fine needles to increase single-sample volume, which is mounted on a 3D-printed base attached to the hydrogel catapult. The hydrogel catapult exhibits normal operation in aqueous or wet environments, which guarantees the feasibility of use in wet or mucous environments inside the body (Supplementary Fig. 37). However, the presence of water reduces the adhesion between the hydrogel catapult and the substrate, leading to performance degradation. A customized PDMS shell is utilized to prevent NIR light leakage, ensure stable adhesion between the catapult and the substrate, and protect the samples from contamination. This needle storage space inside the shell features a stepped structure to achieve a controllable ejection distance (Fig. 5a), while an upper channel with a slightly larger diameter than the needle allows for orientation correction during needle ejection to achieve vertical insertion and reduce alignment difficulties. Note that the sampling needle is barbed to prevent sample loss during withdrawal (Fig. 5a). A tissue-like material (i.e., 5 wt% agarose gel) was used to visualize the penetration during the sampling process. A representative ejection sequence is shown in Fig. 5b and Supplementary Movie 6. When NIR light irradiates from the bottom of the system, the phase change-induced deformation inside the hydrogel catapult propels the needle at high speed, resulting in a penetration of ~ 6 mm into the agarose gel (the maximum allowed moving distance, predetermined by the shell structure, is 7 mm). The penetration depth can be adjusted through the shell structure and deformation control of the hydrogel catapult. Note that the penetration depth shows a limited difference between vertical and horizontal ejection events (Fig. 5c-inset). Subsequently, the sampling needle retracts into the protective shell via a tethered traction mechanism (Supplementary Fig. 38 and Supplementary Movie 6), preventing sample contamination and eliminating in vivo safety risks (Fig. 5b). Concurrently, the integrated barbed design ensures secure and reliable sample retention (Fig. 5d). We also conducted sampling tests in real tissues as shown in Fig. 5e. Results demonstrate that the needle can be easily inserted into deep tissues and acquire samples (Fig. 5f), highlighting the powerful force output and application potential of this developed hydrogel catapult. Note that the hydrogel catapult is encapsulated within the PDMS shell, ensuring that only limited temperature increase occurs at the robot-tissue interface (the measured peak temperature was 40.3 °C as shown in Supplementary Fig. 12), thereby preventing thermal damage to surrounding tissues.

Additionally, by integrating real-time imaging, we demonstrated that the developed biopsy system could be accurately guided to target areas within the porcine stomach using a tethered control method (Fig. 5g, h and Supplementary Movie 7). Deep tissue sampling is achieved by activating the deformation of the hydrogel catapult and subsequent needle retraction. The demonstrated biopsy process and application scenario indicate the effectiveness of this hydrogel catapult-based biopsy system. We also conducted a detailed comparison between our developed system and clinically used instruments to demonstrate the translational potential of our strategy. Details can be found in Supplementary Note 7.

## Discussion

Drawing on the physical analogy of sporangium ejection in *Sphaerobolus*, we developed an actuation strategy that utilizes phase transition-induced momentum transfer for impulsive ejection in miniature robotic systems. This approach harnesses rapid elastic deformation coupled with tripartite dynamic interactions among the deformed hydrogel, projectile, and substrate. Based on this strategy, we developed a hydrogel catapult capable of ejecting projectiles spanning 0–1000 times its body weight, achieving a power density of >8.74 × 10^4^ W/kg, a take-off velocity of 16.6 m/s, an actuation time of 1.6 ms, and controllable ejection direction. The hydrogel catapult is further demonstrated by a biopsy robot for deep tissue sampling in gastric environments. The reported kinetic energy transfer mechanism and ejection capabilities of the developed catapult provide an approach to achieving high-speed motion and high power/force output at miniature scales, supporting applications in miniature robotic systems.

It is worth noting that, similar to the most powerful natural ejection systems, the superior performance of the hydrogel catapult comes at the cost of limited reusability. The single-use characteristic allows the device to release all stored energy in one shot, generating power densities and velocities far exceeding those achievable with reversible mechanisms. For current biopsy applications, the single-use nature complies with FDA requirements for instrumentation in biopsy procedures, e.g., biopsy needles and syringes are intended for single use. Furthermore, through grid or array designs, programmable sequential ejection can be achieved, which helps to improve spatial selectivity and satisfy the requirements for scenarios demanding repeated actuation of the catapult.

## Methods

### Materials

Acrylic acid (AA), acrylamide (AM), *N*,*N*’-methylenebisacrylamide (MBA), 2,4,6-trimethylbenzoyl-diphenylphosphine oxide (TPO), sodium dodecyl sulfate (SDS), polyvinylpyrrolidone (PVP), agar and isopropyl alcohol (IPA) were purchased from Aladdin Chemicals. The graphene (Gr) and PDMS were purchased from Alibaba Co., Ltd., China.

### Preparation of the hydrogel catapult

In a typical experiment, 3 mL AA was neutralized with 1 mL NaOH solution (6.25 mol/L) under constant stirring. To this mixture, we sequentially added 3 g AM, 10.96 mL deionized water, and 20 mg MBA, stirring until complete dissolution. Subsequently, 80 mg water-soluble TPO photo initiator was added, which was fabricated following the method reported in the literature^50^. The mixture solution was stirred in the dark for 2 h to obtain a clear and transparent precursor solution for the bottom hydrogel layer. For the light-absorbing upper layer, we prepared black precursors by adding 50 mg graphene as a light absorber to the above transparent precursor solutions and dispersed by ultrasonication for 15 min under light-protected conditions to ensure uniform dispersion and prevent premature photoinitiation. A homemade 3D-printed square groove (6 cm × 6 cm) was used for polymerization of the hydrogel, note that the hydrogel catapult can be fabricated on a large-scale by simply adjusting the model size. The transparent precursors were first polymerized in the bottom layer under irradiation of UV light (~405 nm) for 3 min, followed by adding the black precursors and irradiating with UV light for 5 min. The double layer hydrogel catapult was successfully fabricated. The thickness of each layer was controlled by fixing the mold dimensions and precisely varying the volume of precursor solution added. Unless otherwise stated, the thicknesses of the upper (with graphene) and lower (without graphene) hydrogel layers are 1.35 and 1.65 mm, respectively (precursor volumes are 4.5 and 5.5 mL, respectively). The catapult can be cut into arbitrary shapes according to the application scenario, with default water and MBA content of 60 and 0.11 wt%, respectively. For long-term storage, the hydrogel catapult can be preserved by either tightly wrapping it in plastic film or freezing it at low temperatures (−20 °C), effectively preventing water loss (Supplementary Fig. 39).

### The fabrication of a light-driven biopsy robot and ex vivo biopsy demonstration

The light-driven biopsy system was fabricated as follows. First, the PDMS shell was fabricated by the template method. The PDMS precursor with a 10:1 weight ratio of the monomers and curing agents were stirred for 3 min, then poured into the 3D-printed mold and vacuum degassing for 10 min, the mold filled with precursor was then placed in oven (60 °C) for 2 h until platinum catalyzed polymerization process finished. The transparent PDMS shell was successfully prepared after the demolding and sealing process. Next, a round piece of glass plate was embedded in the middle of the shell to allow the NIR light cross and absorbed by the hydrogel catapult. The needle was fixed to a 3D-printed basement and placed on the hydrogel catapult inside the PDMS shell. The optical fiber was installed at the bottom of the PDMS shell to produce the NIR light, the entire structure was sealed with a membrane to prevent liquid penetration. Following component integration, the biopsy robot was successfully fabricated and functionally validated.

For the biopsy demonstration, the fresh porcine stomachs were obtained from a local commercial slaughterhouse in Hong Kong and transported to the laboratory in phosphate-buffered saline (PBS) at 4 °C. All experiments were performed within 3 h of tissue procurement to ensure tissue freshness and mechanical integrity. The developed biopsy robot navigated by endoscope to the target area in the porcine gastric, the biopsy procedures were performed on tissue from a single porcine stomach with more than three times technical replicates, i.e., independent needle ejection and sampling events at different locations on the gastric wall.

### Characterization

The high-speed images and videos were recorded by the Revealer M136 high-speed camera with a frame rate of 5000 fps. The light source was 808 nm NIR light from Beijing Leiyuan Technology Co., Ltd. Unless otherwise mentioned, the laser power and distance between the light source and soft robot were 21.26 W/cm^2^ and 5 cm, respectively. The tensile experiments, toughness measurement and adhesion force test were performed on the MACH-1 system. The temperature change and infrared thermal images were obtained by using a FLIR-A300 camera. The navigation of the biopsy robot was through an endoscope (GIF-HQ 190, Olympus).

## Supplementary information

Supplementary information

Description of Additional Supplementary Files

Supplementary Movie 1

Supplementary Movie 2

Supplementary Movie 3

Supplementary Movie 4

Supplementary Movie 5

Supplementary Movie 6

Supplementary Movie 7

Transparent Peer Review file

## Source data

Source data of Fig. 1

Source data of Fig. 2

Source data of Fig. 3

Source data of Fig. 4

Source data of Fig. 5

## Data Availability

All data were available in the Article or its Supplementary Information. Source data are provided with this paper.
