# Supplementary material for: Dynamic coupling enables impulsive ejection in a hydrogel catapult
Source: Nat Commun. 2026 Sep 7;17:10568. doi: 10.1038/s41467-026-77391-6 (PMC13642336; doi:10.1038/s41467-026-77391-6)
Supplement: Supplementary file 1 [file 41467_2026_77391_MOESM10_ESM.pdf]

nature portfolio

Peer Review File

Dynamic coupling enables impulsive ejection in a hydrogel catapult

Corresponding Author: Professor Li Zhang

This file contains all reviewer reports in order by version, followed by all author rebuttals in order by version.

Version 0:

Reviewer comments:

Reviewer #1

(Remarks to the Author)
Due to the inherent properties of soft materials that limit energy storage capacity and response speed, achieving high motion performance and high force/power output in miniature soft robotics remains very challenging. In this work, the authors present a bioinspired actuation strategy based on ultrafast momentum transfer for high-speed ejection. By leveraging the rapid deformation of a developed hydrogel catapult and the dynamic coupling among the catapult, projectile, and substrate, impressive ejection performance is achieved. Potential applications in biopsy robots for deep tissue sampling are also explored. Overall, this is an interesting work, and the data and theoretical analysis are substantial. The manuscript is clearly and concisely written. The results seem to be interesting and useful, and it would be better if some extra information was added and a number of points were clarified considering the following comments.

1. What's the function of graphene in the hydrogel catapult, and could the graphene be replaced with other functional materials? How does this substitution affect the deformation process of the hydrogel?

2. The transparent bottom hydrogel layer appears to play a crucial role in the catapult mechanism. The authors should discuss how its thickness influences the ejection performance.

3. For Supplementary Fig. 16, high-speed force sensor data could strengthen the claim of “ultrafast momentum transfer.” A locally magnified plot showing the timescale is needed.

4. Hydrogel materials are prone to water loss. How to achieve the long term storage of the catapult?

5. In the Methods section, please specify the exact graphene concentration and dispersion method for reproducibility. Also, the thickness distribution of the two layers of the catapult should be clearly described.

6. Some sentences are lengthy and could be simplified for clarity (e.g., in the Results section). Also, ensure consistent use of terms like “actuation time” and “response time.”

Reviewer #2

(Remarks to the Author)
Wang et al. propose a soft actuator based on a high–energy density bilayer hydrogel. By leveraging dynamic coupling among actuator deformation, projectile motion, and substrate interaction, the system achieves high power output and rapid kinetic energy release. The resulting momentum transfer and power density enable fast, impulsive locomotion. The authors further demonstrate a potential application in fine-needle biopsy. Overall, the concept of using laser actuated hydrogel for soft actuator seems interesting. But there is some concern about the needs to justify the novelty compared with existing works. Below are further comments.

1. Related work / positioning. The manuscript should discuss and compare against closely related hydrogel jump/roll actuators to better clarify novelty and performance. In particular, please consider:
o Li, M., Wang, X., Dong, B., and Sitti, M. (2020). In-air fast response and high speed jumping and rolling of a light-driven

hydrogel actuator. Nature Communications, 11(1), 3988.
A direct comparison of key metrics (e.g., response time, jump height/range, power density, payload-to-weight ratio, repeatability, and actuation conditions) would strengthen the contribution.
2. Directional control. How is the jumping direction controlled? Does it depend on the direction/position of laser illumination, actuator geometry, or substrate anisotropy? Please quantify directional repeatability (e.g., angular dispersion over n trials) and provide a controllability strategy if applicable.
3. Laser power dependence. How does laser power (and spot size/irradiance) affect jump height, velocity, and payload capacity? A parametric study linking optical input to mechanical output would improve rigor.
4. Scaling considerations. The manuscript demonstrates millimeter-scale actuators. What are the key limits and opportunities for scaling toward sub-millimeter dimensions (e.g., fabrication, heat/mass transfer timescales, mechanical robustness, and payload fraction)?
5. Operation in aqueous environments. Can the actuator function underwater or in wet conditions? If not, please discuss the primary failure modes (e.g., optical attenuation, altered friction, swelling kinetics, damping) and what design changes would be required.
6. Biopsy demonstration—clinical advantage. Figure 5 shows biopsy via needle punching driven by the actuator. What is the advantage of this approach compared with existing biopsy tools (manual/robotic fine-needle aspiration, spring-loaded biopsy devices, or catheter-based systems)? Please clarify the intended clinical scenario and provide comparative discussion (precision, delivered impulse, access, safety, and potential tissue trauma).
7. Substrate and payload effects. How does launch height (and/or distance) vary with substrate properties (roughness, stiffness, friction coefficient) and payload mass? A quantitative characterization would help assess real-world robustness and application feasibility.

Reviewer #3

(Remarks to the Author)
Wang et al. designed a hydrogel catapult inspired by the ejection of Sphaerobolus spores. While the initial idea and claims are novel, the final device design is limited, and the proposed biomedical application does not align with the core concept.
A significant issue with the hydrogel catapult is that it is single-use; after the initial explosion and fracture of the hydrogel network, the catapult must be replaced. This contradicts the traditional understanding of a "catapult," which implies a reusable mechanism. If the authors intend to demonstrate repeatable ejections with the same hydrogel catapult, they must quantify the number of consecutive successful ejections the device can perform to show its stability and lifetime.
Additionally, there is a lack of detailed material characterization beyond basic stress-strain tests. It is unclear what chemical changes or byproducts occur within the hydrogel network during actuation and phase transition.
Another critical issue is the proposed biomedical application of endoscopic sample collection. This application is highly impractical in realistic clinical scenarios, given the significant force applied, the required near-infrared light intensity, and the substantial temperature increase. The authors note that the temperature exceeds 120°C in the upper layer. Single-use, explosive-driven equipment is simply not viable for delicate tissue endoscopic applications. The authors should focus on non-medical applications of this technology instead. For these reasons, I believe this manuscript is currently unsuitable for publication in any scientific journal.
Other comments:
- While the authors claim this is a "hydrogel catapult," the system is essentially a composite of a PDMS shell, graphene, and P(AA-co-AM) layers. The authors should focus on designing a device to control explosion-induced ejection. Currently, there is a lack of structural device design to physically control explosion-induced ejection. The authors could improve controlled ejection by incorporating grids or additional metastructures, such as kirigami patterns or bistable mechanisms.
- While sample sizes are provided for some experiments, the authors must ensure that exact sample size information (e.g., n values) is included consistently across all main figure legends and in the Results section for clarity.
- The ex vivo experimental conditions are unclear. The authors cannot use organs in their studies without standardizing or registering them. The source of the tissue is unclear, and there is no detailed information about the properties of the porcine gastric tissue. For scientific transparency and ethical compliance, the source of the ex vivo tissue and the institutional registration number for animal byproduct use must be included. Ex vivo studies are also considered in life science study design. Please include details about the experimental design, such as biological replicates.
- Time stamps are missing from the supplementary videos. Additionally, the playback speed changes mid-action in some videos, which is unacceptable for rigorous scientific documentation. Specifically, Supplementary Video 3 lacks speed information entirely.

Version 1:

Reviewer comments:

Reviewer #1

(Remarks to the Author)
I have carefully read the authors'responses and examined their responses to other reviewers. The manuscript has been appropriately revised following the comments received, and I think it's now ready for publication.

Reviewer #2

(Remarks to the Author)
The authors have addressed most of my comments in this round of revision. However, key concerns still remain.

First, the novelty of this work remains a primary concern, as it appears to be significantly limited by previous studies. The fundamental mechanism is essentially the same as that reported in Li et al. 11(1), 3988, Nature Communications (2020), which was not cited in the original submission. Both studies employ NIR-light-driven photothermal heating to induce phase transitions of water within hydrogel networks. The authors also acknowledge that the two works share the same fundamental hydrogel actuation mechanism. Therefore, the current study appears to represent only an incremental advance, primarily by transferring the generated kinetic energy to external objects, while the demonstrations remain relatively straightforward and resemble discrete proof-of-concept showcases.

Moreover, the inherently unidirectional actuation nature of the projectile mechanism makes the system less compelling compared with previous studies focusing on repeatable hydrogel robot locomotion/actuation. The issue of lacking bidirectional momentum transfer is largely overlooked, which significantly limits the practical applicability of the proposed mechanism. One notable example is the biopsy unit demonstration. A major concern is the retrieval of the biopsy unit after sampling. Although the authors demonstrated sampling in a retroflexed region, it remains unclear how the biopsy unit can be safely and reliably retrieved afterward, which could present substantial technical challenges in practical implementation.

Lastly, additional literature support is needed for the claims related to the biopsy unit. The authors state that the ability to use a 14 G needle could reduce the number of biopsy passes; however, no supporting references are provided to substantiate the claim that “multiple punctures (typically 3–7 passes) are often required to obtain sufficient diagnostic material, which increases procedure time, patient discomfort, and the risk of complications.”

Reviewer #3

(Remarks to the Author)
I would like to express my gratitude to the authors. They provided thorough answers to all my inquiries regarding the article, ensuring its suitability for publication.

Version 2:

Reviewer comments:

Reviewer #2

(Remarks to the Author)
The authors have now carefully addressed all my comments in this revision. I recommend it to be published. Thanks!

Open Access This Peer Review File is licensed under a Creative Commons Attribution 4.0 International License, which permits use, sharing, adaptation, distribution and reproduction in any medium or format, as long as you give appropriate credit to the original author(s) and the source, provide a link to the Creative Commons license, and indicate if changes were

made.
In cases where reviewers are anonymous, credit should be given to 'Anonymous Referee' and the source.
The images or other third party material in this Peer Review File are included in the article's Creative Commons license, unless indicated otherwise in a credit line to the material. If material is not included in the article's Creative Commons license and your intended use is not permitted by statutory regulation or exceeds the permitted use, you will need to obtain permission directly from the copyright holder.
To view a copy of this license, visit https://creativecommons.org/licenses/by/4.0/

Reviewer #1:

Due to the inherent properties of soft materials that limit energy storage capacity and response speed, achieving high motion performance and high force/power output in miniature soft robotics remains very challenging. In this work, the authors present a bioinspired actuation strategy based on ultrafast momentum transfer for high-speed ejection. By leveraging the rapid deformation of a developed hydrogel catapult and the dynamic coupling among the catapult, projectile, and substrate, impressive ejection performance is achieved. Potential applications in biopsy robots for deep tissue sampling are also explored. Overall, this is an interesting work, and the data and theoretical analysis are substantial. The manuscript is clearly and concisely written. The results seem to be interesting and useful, and it would be better if some extra information was added and a number of points were clarified considering the following comments.

Many thanks to the reviewer for careful review and high evaluation of this work. Your constructive comments have helped us to further improve our manuscript. We have addressed each comment and made the appropriate changes in the revised version of manuscript. Please check the following point-by-point response for details.

1. What's the function of graphene in the hydrogel catapult, and could the graphene be replaced with other functional materials? How does this substitution affect the deformation process of the hydrogel?

The authors appreciate the reviewer's comments. The function of graphene in hydrogel catapult serves as energy transducer and optical barrier to control the phase transition occurred inside the hydrogel matrix. To address the reviewer's suggestion, we have revised the manuscript and added a discussion on substitutability and effects on the deformation process as Supplementary Note 1. The details are as follows,

(1) The function of graphene in hydrogel catapult serves as energy transducer and optical barrier to control the phase transition occurred inside the hydrogel matrix. First of all, the graphene serves exclusively as a photothermal conversion agent in the upper layer of the bilayer hydrogel catapult. Its function is to absorb near-infrared (NIR) light and convert it

into localized heat with high efficiency. This rapid heat generation raises the local temperature above the boiling point of water (exceeding 120 °C within ~2 s, as shown in Supplementary Fig. 16 (Fig. R1.1)), thereby triggering the ultrafast liquid-to-vapor phase transition of water confined within the hydrogel network. The resulting steam expansion drives the rapid deformation that constitutes the core actuation mechanism.

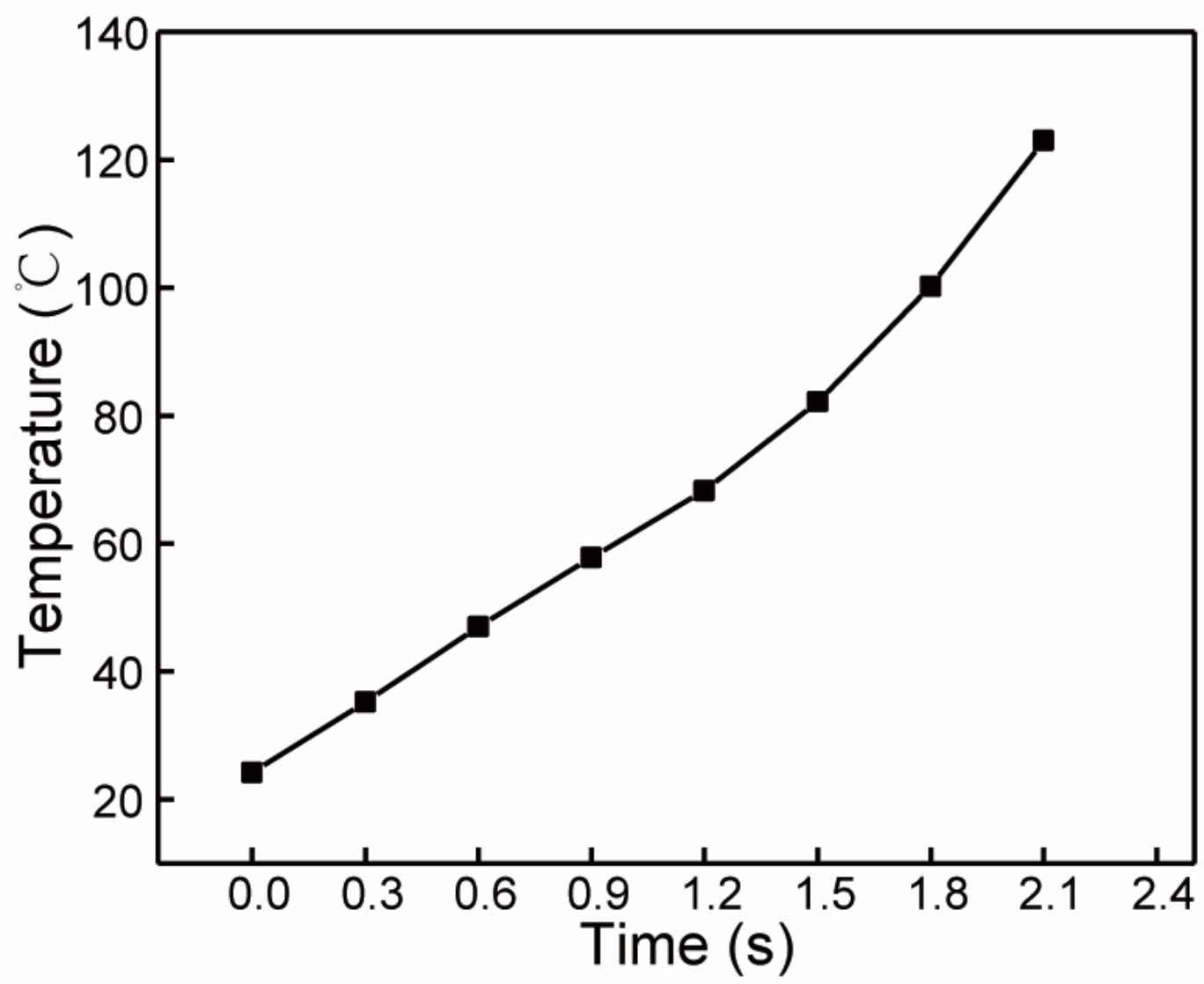

Fig. R1.1 (Supplementary Fig. 16). The temperature change of the hydrogel catapult irradiated with NIR light.

Beyond photothermal conversion, the black color of graphene plays an equally important role by attenuating the NIR light as it penetrates into the hydrogel. Due to the strong optical absorption of graphene, the incident NIR light cannot fully penetrate through the graphene-doped upper layer. Instead, the light energy is progressively absorbed within a limited penetration depth from the irradiated surface. This optical confinement effect ensures that the phase transition occurs internally within the hydrogel at a controlled depth beneath the surface rather than throughout the entire volume, which is essential for generating the localized internal pressure buildup that drives the directional deformation. To quantitatively verify this optical attenuation effect, we conducted additional experiments in which hydrogel samples with varying graphene concentrations were placed between the NIR light source and a power density meter. As shown in the Supplementary Fig. 1 (Fig. R1.2), the detected light intensity decreases progressively with increasing graphene

concentration, confirming that graphene effectively limits the light penetration, and consequently induced the phase transition occurred in a certain depth within the upper layer.

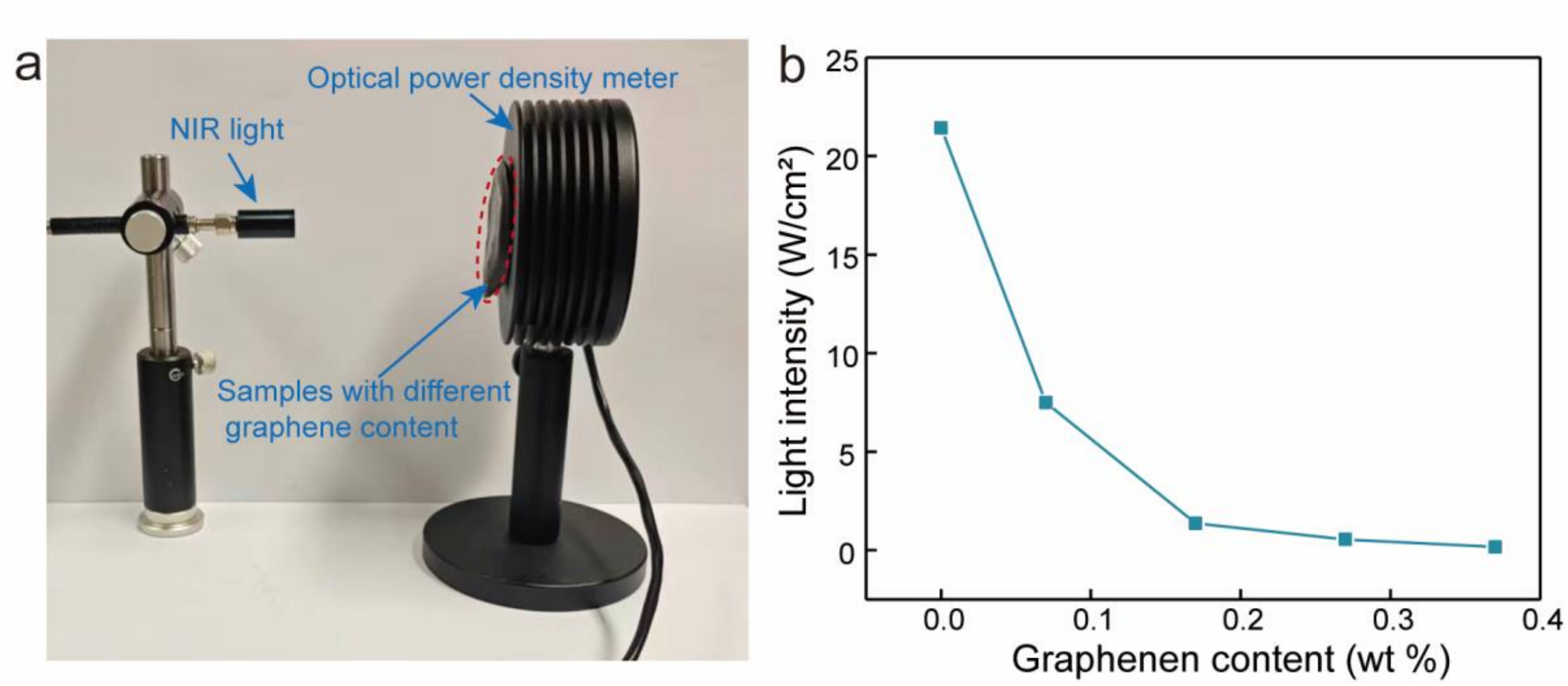

Fig. R1.2 (Supplementary Fig. 1). (a) Photograph of the experiment platform. The samples with different graphene content placed between light source and optical power density meter to test the optical attenuation effect. (b) The detected light intensity for samples with different graphene content. Note that the light intensity is 21.26 W/cm^2.

In addition, it is important to emphasize that graphene does not play a structural or mechanical role in the ejection process. The mechanical performance of the catapult, including its stretchability, toughness, and elastic recovery, is governed by the P(AA-co-AM) hydrogel network itself (Supplementary Fig. 8c), while graphene functions as both an energy transducer and an optical barrier to control the phase transition occurred inside the hydrogel matrix.

(2) Feasibility of substitution with other functional materials. Graphene can in principle be replaced by other materials, provided that the substitute fulfills both functions described above, i.e., efficient photothermal conversion and sufficient optical attenuation to spatially confine the phase transition. Following the reviewer's suggestion, we conducted substitution experiments using representative photothermal nanoparticles, including MXene and Fe_3O_4, in place of graphene. The results confirm that all tested substitutes successfully enabled the ejection process, albeit with varying performance levels (Supplementary Fig. 2, as shown in Fig. R1.3). We note that the ejection performance is influenced by the loading concentration of the photothermal agent, which we did not

optimize in these substitution experiments. The purpose here was to qualitatively demonstrate the feasibility of material substitution rather than to conduct a comprehensive parametric study.

To further investigate the effect of material substitution on the deformation process, we used high-speed camera imaging to analyze the deformation dynamics of a representative substitute system (i.e., Fe_3O_4-doped hydrogel catapult) and compared it with the original graphene-doped catapult. As shown in Supplementary Fig. 3a (Fig. R1.4a), the Fe_3O_4-doped catapult exhibited smaller deformation magnitude and earlier fracture compared to the graphene-doped catapult. In contrast, the graphene-doped catapult displayed more uniform deformation and a prolonged acceleration process for the projectile (i.e., 1.8 ms *vs* 1.0 ms as shown in Supplementary Fig. 3a (Fig. R1.4a)). The underlying reason is attributed to the dispersion homogeneity of the photothermal agent within the hydrogel matrix. As shown in Supplementary Fig. 3b (Fig. R1.4b), graphene can be dispersed relatively uniformly within the hydrogel, whereas Fe_3O_4 nanoparticles tend to aggregate during the polymerization process, resulting in a non-uniform spatial distribution. This inhomogeneous distribution leads to localized non-uniform heat generation and introduces structural defects in the hydrogel network, which in turn promote premature fracture and reduce the overall deformation and momentum transfer efficiency.

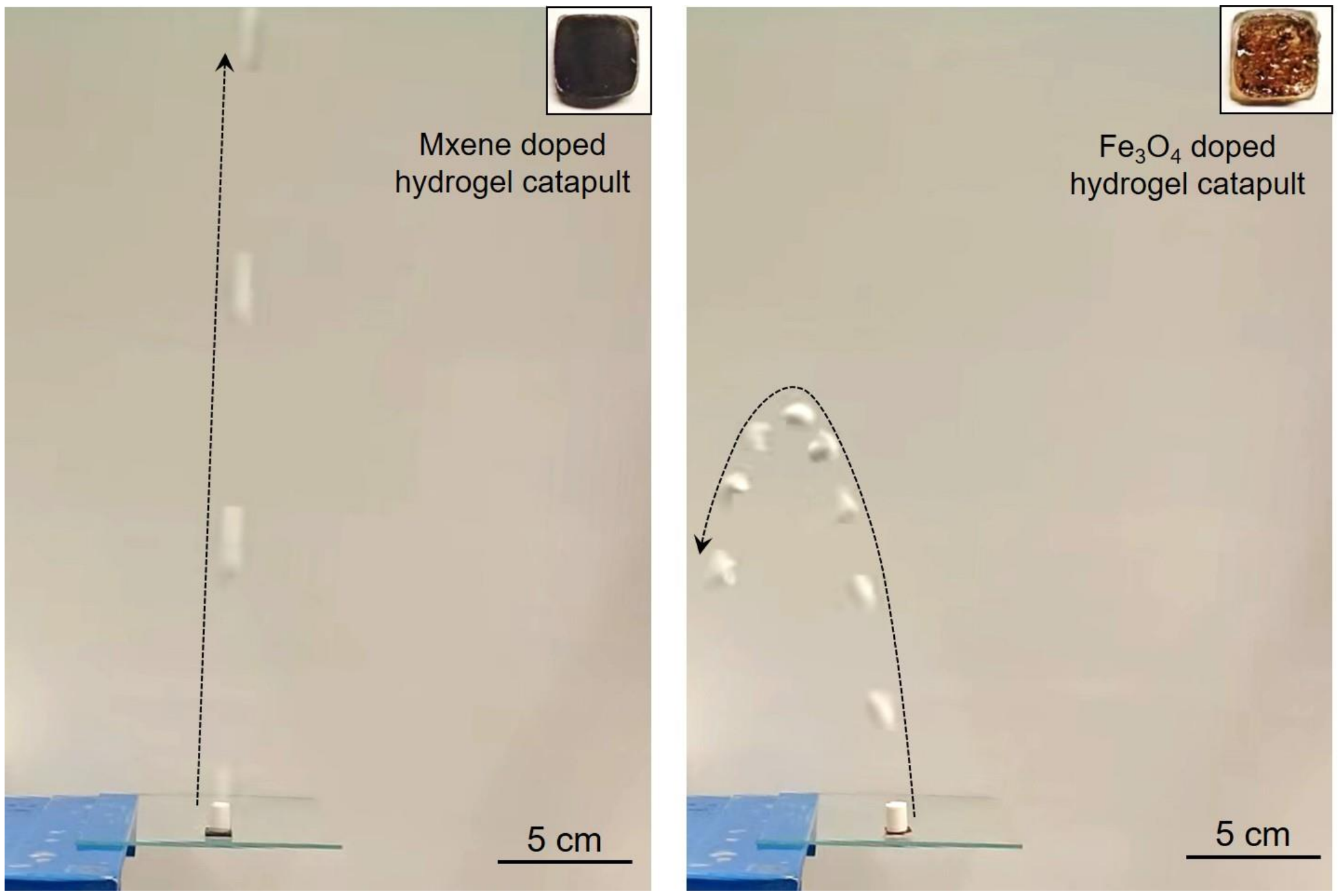

Fig. R1.3 (Supplementary Fig. 2). The overlaid images illustrating the ejection behavior of the Mxene and Fe_3O_4 doped hydrogel catapult.

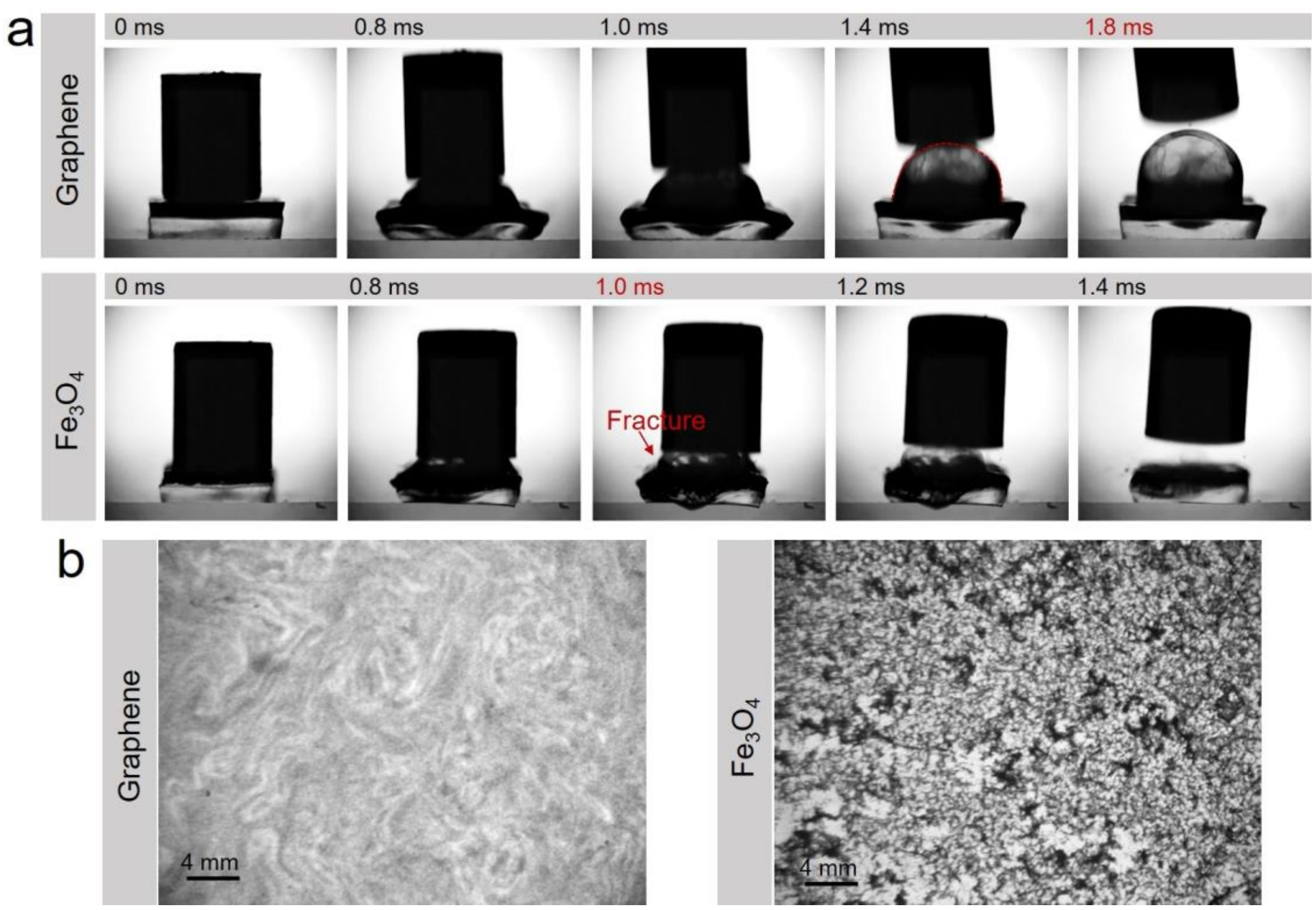

Fig. R1.4 (Supplementary Fig. 3). (a) The high-speed camera image illustrates the actuation process of the graphene and Fe_3O_4 doped hydrogel catapult. (b) The microscopy images showing the dispersion uniformity of the graphene and Fe_3O_4 nanoparticles in the hydrogel matrix.

Furthermore, we would like to clarify that the energy input is not restricted to NIR light. In principle, any heating method capable of inducing sufficiently rapid and localized phase transition, such as electrical resistive heating, focused ultrasound, microwave irradiation, or chemical exothermic reactions, could serve as an alternative energy source to trigger the same actuation mechanism. When using non-optical energy sources, the optical attenuation function of graphene becomes unnecessary, and the photothermal agent can be omitted entirely or replaced with materials optimized for the alternative energy modality. This versatility underscores that the core contribution of this work lies in the dynamic coupling framework, rather than in the specific choice of photothermal material or energy input method.

The above-mentioned figures and discussion were added in Supplementary information as Supplementary Note 1. We further edited the manuscript on page 7, line 125 as follows:
"This leads to rapid temperature increase (exceeding 120 °C within 2 s) in the upper layer of the irradiated area due to the photothermal effect of the doped graphene (Supplementary Fig. 16), the graphene acts as both photothermal converter and an optical barrier, thereby inducing the heat production occurred inside the hydrogel matrix. Note that the graphene-enabled rapid heat production is only a representative example, any other material system or trigger method capable of inducing a localized and rapid heat production inside the hydrogel can be employed (Supplementary Note 1)."

2. The transparent bottom hydrogel layer appears to play a crucial role in the catapult mechanism. The authors should discuss how its thickness influences the ejection performance.

The authors appreciate the reviewer's comments and agree that the transparent bottom hydrogel layer indeed plays a crucial role in the catapult mechanism, and its thickness is an important design parameter that governs the ejection performance. As the reviewer's suggestion, we have discussed the functions and tested the influence of transparent bottom layer's thickness as shown in Supplementary Fig. 21 (Fig. R1.5), the detailed discussion are as follows.

The transparent bottom hydrogel layer serves three essential functions in the ejection system. First of all, the bottom layer acts as a structural constraint that directs the phase-transition-induced deformation preferentially upward toward the projectile. Because the bottom layer is thicker than the upper graphene-doped layer, it provides greater resistance to downward expansion, forcing the steam-driven deformation toward the thinner, less resistant upper layer. This asymmetric thickness design is directly inspired by the functional role of the multilayer membrane structure in *Sphaerobolus*, where layers of different thickness and stiffness guide the deformation direction during sporangium ejection. Second, the transparent layer allows NIR light to pass through the bottom layer without significant absorption, ensuring that the photothermal energy conversion occurs selectively in the upper graphene-doped layer. Third, the bottom layer, in combination with the rigid substrate and interfacial adhesion, forms a pressure confinement barrier that blocks downward vapor expansion and redirects the airflow upward. The mechanical integrity and thickness of this layer directly determine the effectiveness of this pressure redirection.

The thickness of the bottom layer affects the ejection performance through the balance between constraint effectiveness and energy dissipation. We have tested the ejection performance of the hydrogel catapults with different thickness of transparent bottom layer as shown in Supplementary Fig. 21a (Fig. R1.5). We fixed the substrate in the test is glass substrate, and the graphene doped upper layer was 1.35 mm thick. All samples were successfully launching the projectile, and the ejection performance first increased and then decreased with increasing thickness of the bottom transparent layer.

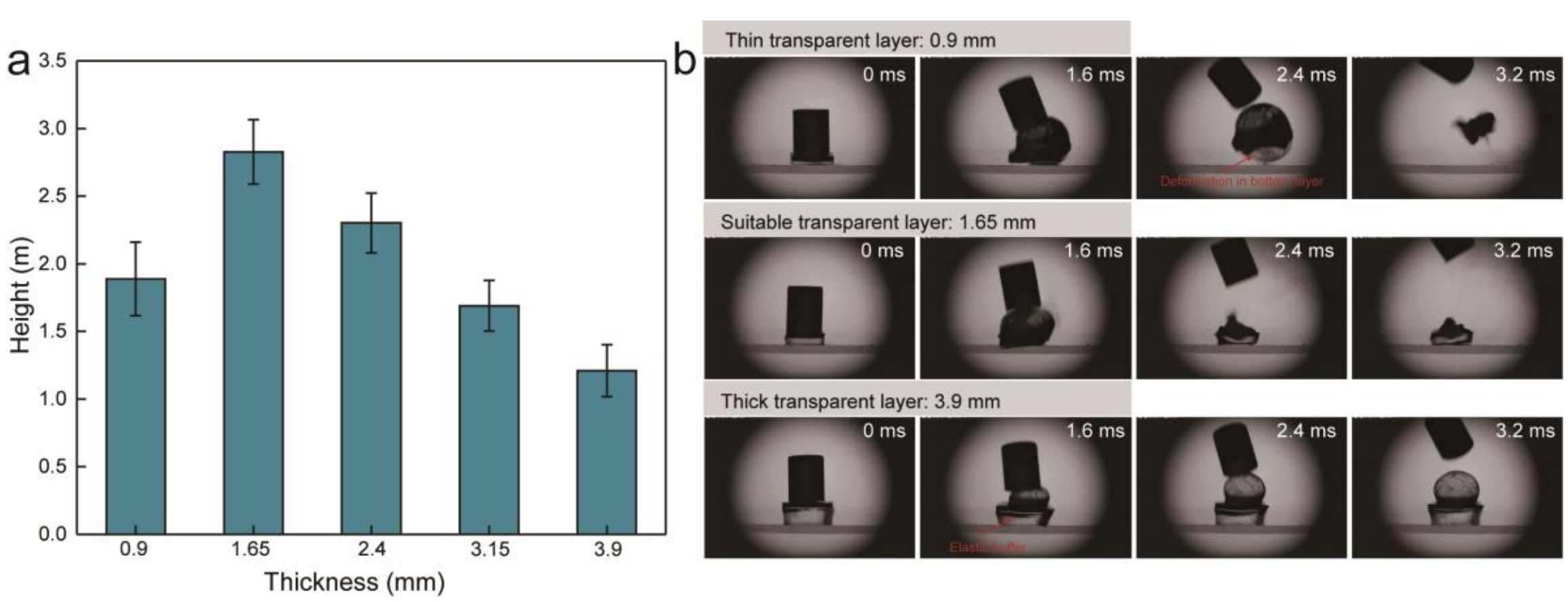

Fig. R1.5 (Supplementary Fig. 21). (a) The performance of catapults with different transparent layer thicknesses. Note that the thickness of graphene doped upper layer is 1.35 mm. The substrate is glass materials and the projectile's weight is 380 mg. (b) The representative high speed camera image of hydrogel catapult with thin, suitable and thick transparent layer.

When the bottom layer is too thin (e.g. 0.9 mm as shown in Supplementary Fig. 21b, (Fig. R1.6b)), it provides inadequate resistance to the downward vapor expansion. In this case, the phase-transition-induced pressure deforms both the upper and lower layers comparably, resulting in bidirectional deformation rather than preferential upward deformation. This reduces the net upward momentum transfer to the projectile. In extreme cases, an excessively thin bottom layer may rupture under the internal pressure before sufficient upward deformation is achieved, leading to premature energy release and poor ejection performance. Furthermore, a very thin bottom layer compromises the structural integrity needed for effective substrate adhesion and pressure confinement.

When the bottom layer is excessively thick (e.g. 3.9 mm as shown in Supplementary Fig. 21b, (Fig. R1.5b)), it provides strong directional constraint, but also absorbs a significant portion of the phase-transition energy through elastic deformation of the increased material volume. The thicker bottom layer acts as an elastic buffer that dissipates part of the vapor expansion energy before it can be redirected upward. Additionally, a thicker bottom layer increases the total distance between the phase-transition zone and the substrate, potentially reducing the efficiency of airflow reflection from the rigid substrate.

The bottom-to-upper layer thickness ratio of approximately 1.2 : 1 can achieve best ejection performance for our current hydrogel composition and catapult dimensions. Below this ratio, directional control is compromised; above this ratio, ejection height gradually decreases due to increased energy dissipation.

We have added the above Figure into Supplementary figures and revised the manuscript on page 8, line 148 as follows:

"However, the combination of the thicker bottom layer and the rigid substrate (a non-deformable glass slide), secured by interfacial adhesion, forms a strong constraint that blocks downward vapor expansion and redirects the airflow upward, where the thinner upper layer offers the less resistance (Supplementary Fig. 20). Consequently, the combined

effect of the initial upward airflow and the redirected downward airflow results in rapid upward deformation of the hydrogel catapult, generating the initial momentum for ejection (Fig. 1a). The thickness of the bottom layer plays an important role in controlling the deformation direction, maintaining the stability of the catapult, and inducing airflow rebound, the optimal thickness ratio of the upper layer to the bottom layer is 1:1.2 (Method and Supplementary Fig. 21)."

3. For Supplementary Fig. 16, high-speed force sensor data could strengthen the claim of "ultrafast momentum transfer." A locally magnified plot showing the timescale is needed.

The authors appreciate the reviewer's comments. We agree that a locally magnified plot would more clearly illustrate the ultrafast timescale of the momentum transfer process. As the reviewer's suggestion, we have added a magnified inset to the original Supplementary Fig. 16 (now is Supplementary Fig. 22, as shown in Fig. R1.6) in the revised manuscript, which highlights the force profile and the critical actuation window.

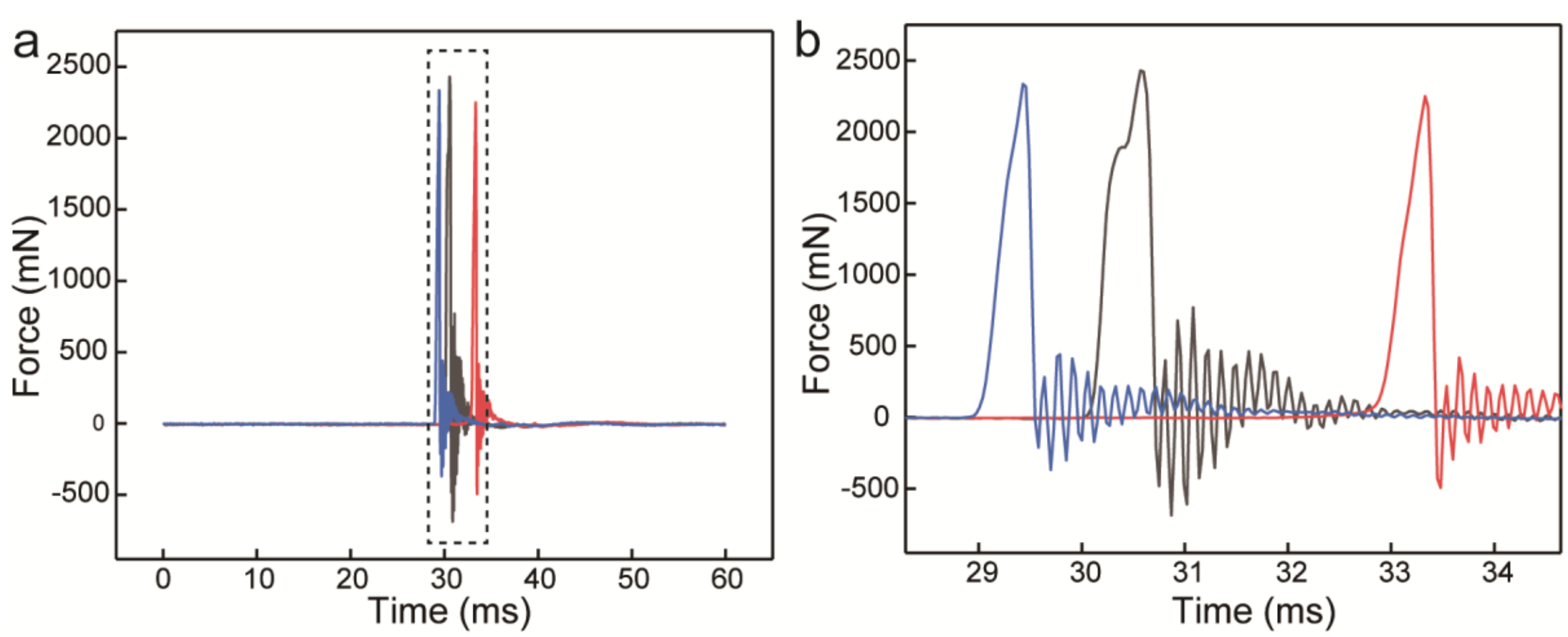

Fig. R1.6 (Supplementary Fig. 22). (a) The measured force output and (b) zoom in plot of the hydrogel catapult from three actuation tests. Note that the acquisition rate is 33.3 kHz of the sensor.

4. Hydrogel materials are prone to water loss. How to achieve the long term storage of the catapult?

The authors appreciate the reviewer's comments. As the reviewer's suggestion, we have supplied the following approaches to enable long-term storage of the hydrogel materials while preventing water loss. First, the fabricated hydrogel with initial weight of 274 mg can be tightly wrapped with plastic wrap and stored in a 4 °C refrigerator to avoid the water evaporation. To evaluate the water retention capability of this method, we measured and recorded the weight of the hydrogel material every three days. As shown in Supplementary Fig. 38 (Fig. R1.7), the hydrogel exhibited limited weight change (~ 2.96 % water loss) over a storage period of 24 days, indicating the current proposed method effectively prevents water loss. Note that the observed slight water loss could be attributed to evaporation that occurred during the measurement process in air.

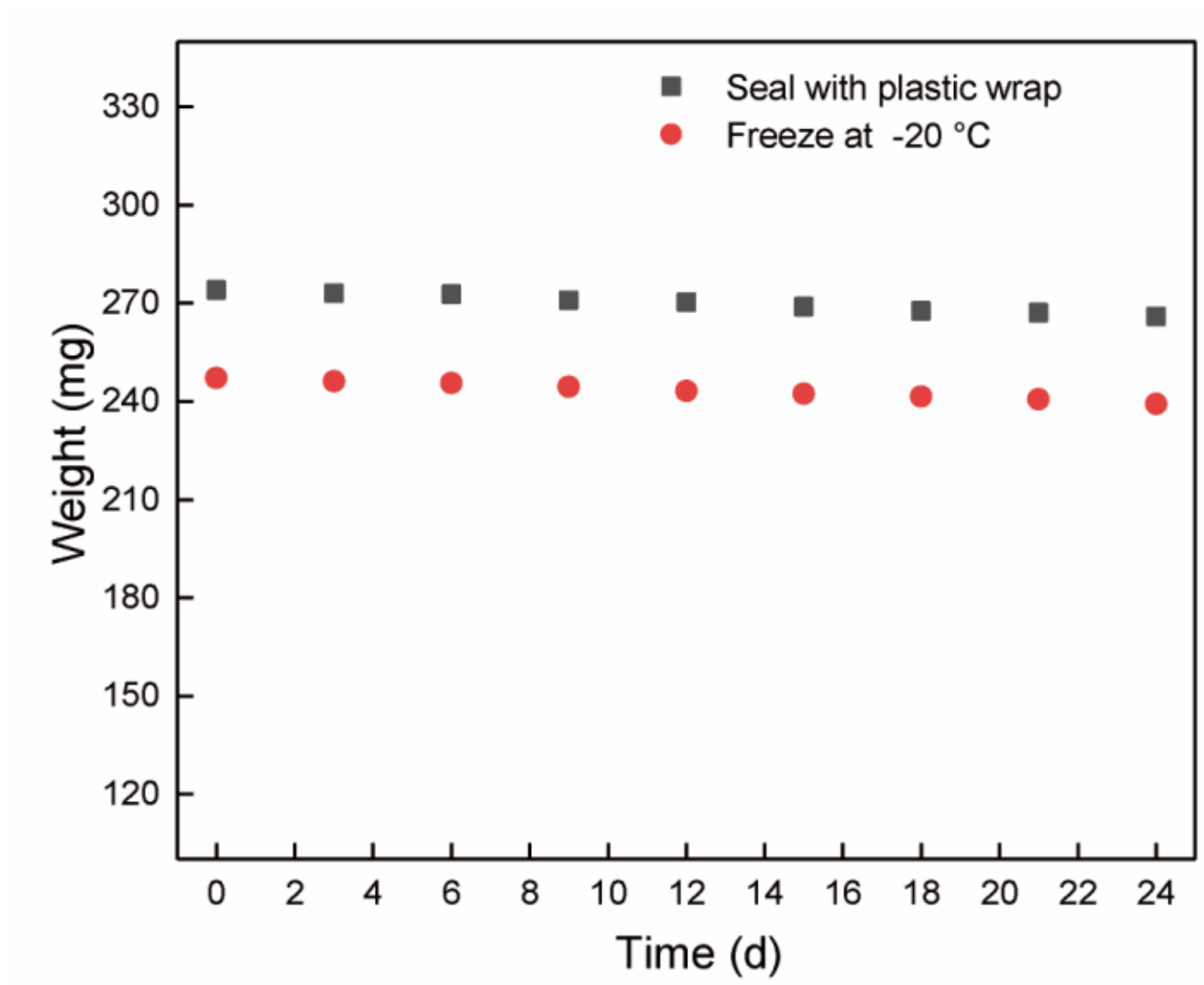

Fig. R1.7 (Supplementary Fig. 38). The weight change of the hydrogel during the long-time storage (i.e., wrapped with plastic wrap and frozen at -20 °C).

Furthermore, our hydrogel catapults are prepared via UV-induced polymerization as shown in Method section in Manuscript. To further mitigate the risk of water loss during storage, the precursor solution that containing monomer, photo initiator, crosslinker, and photothermal material can be prepared in advance and stored in the dark to avoid the polymerization. The hydrogel catapults can be fabricated simply before use by exposing the precursor to UV light to initiate polymerization of the hydrogel. This approach reduces the storage requirements for the hydrogel catapult without compromising its performance.

We have edited the manuscript on page 33, line 598 using the above-mentioned figure as follows:

“For long-term storage, the hydrogel catapult can be preserved by either tightly wrapping it in plastic film or freezing it at low temperatures (-20 °C), effectively preventing water loss (Supplementary Fig. 38).”

5. In the Methods section, please specify the exact graphene concentration and dispersion method for reproducibility. Also, the thickness distribution of the two layers of the catapult should be clearly described.

The authors appreciate the reviewer’s insightful comments. Following the reviewer’s suggestion, we have revised the Methods section to include a detailed description of the graphene dispersion protocol and the bilayer thickness control procedure. To achieve uniform dispersion of graphene nanoparticles within the hydrogel polymer network, it is essential that the graphene is first evenly dispersed in the precursor solution. Accordingly, 50 mg of graphene was added to the transparent precursor (with the detailed composition provided in the Methods section) and dispersed by ultrasonication for 15 minutes under light-protected conditions to ensure uniform dispersion and prevent premature photoinitiation.

In addition, the thickness of each layer was controlled by fixing the mold dimensions (6 cm × 6 cm lateral area) and precisely varying the volume of precursor solution added for each layer. Specifically, the transparent bottom layer was first cast by adding a defined volume of pristine precursor solution into the mold, followed by UV curing (405 nm, 3 min). Subsequently, a defined volume of graphene-doped precursor solution was added on top and cured under UV irradiation (405 nm, 5 min) to form the upper layer. By adjusting the volume ratio between the two layers, the thickness distribution can be precisely controlled. Unless otherwise stated, the thicknesses of the upper (with graphene) and lower (without graphene) hydrogel layers are 1.35 mm and 1.65 mm, respectively, i.e., precursor volumes of the hydrogel are 4.5 ml and 5.5 ml, respectively.

We have revised the Methods section in the manuscript on page 32, line 580 as follows:

"In a typical experiment, 3 mL AA was neutralized with 1 mL NaOH solution (6.25 mol/L) under constant stirring. To this mixture, we sequentially added 3 g AM, 10.96 mL deionized water, and 20 mg MBA, stirring until complete dissolution. Subsequently, 80 mg water-soluble TPO photo initiator was added which fabricated following the method reported in the literature[46]. The mixture solution was stirred in the dark for 2 h to obtain a clear and transparent precursor solution for the bottom hydrogel layer. For the light-absorbing upper layer, we prepared black precursors by adding 50 mg graphene as light absorber to the above transparent precursor solutions and dispersed by ultrasonication for 15 minutes under light-protected conditions to ensure uniform dispersion and prevent premature photoinitiation. A homemade 3D printed square groove (6 cm × 6 cm) was used for polymerization of the hydrogel, note that the hydrogel catapult can be fabricated on a large-scale by simply adjusting the model size. The transparent precursors were first polymerized in the bottom layer under irradiation of UV light (~ 405 nm) for 3 mins, followed by adding the black precursors and irradiated with UV light for 5 mins. The double layer hydrogel catapult was successfully fabricated. The thickness of each layer was controlled by fixing the mold dimensions and precisely varying the volume of precursor solution added. Unless otherwise stated, the thicknesses of the upper (with graphene) and lower (without graphene) hydrogel layers are 1.35 mm and 1.65 mm, respectively, (precursor volumes are 4.5 mL and 5.5 mL, respectively). The catapult can be cut into arbitrary shapes according to the application scenario, with default water and MBA contents of 60 wt% and 0.11 wt%, respectively."

6. Some sentences are lengthy and could be simplified for clarity (e.g., in the Results section). Also, ensure consistent use of terms like "actuation time" and "response time."

The authors appreciate the reviewer's comments. We have thoroughly reviewed the Results section and the entire manuscript to identify and revise overly lengthy sentences to improve readability. For example:

We have edited the manuscript on page 8, line 141 as follows:

“The kinetic energy transfer and ejection arise from the dynamic coupling among three elements: phase-change-induced hydrogel deformation, the substrate, and the projectile. As shown in Fig. 1a and Fig. 1b, the hydrogel catapult is placed on a rigid substrate (e.g., glass plate) to maximize ejection performance. Upon phase change, airflow is generated at the interface and impacts the elastic hydrogel symmetrically from the NIR irradiation center.”

On page 8, line 156,

“The phase transition induced deformation is completed within 1.6 ms (Fig. 1b), generating a peak force output ~ 2.34 N (Supplementary Fig. 22). During this process, the projectile’s gravity disperses the internal pressure within the deformed hydrogel network, delaying fracture and increasing overall deformation. Notably, the projectile acquires its momentum before the deformation reaches its maximum. We define this ~1.6 ms interaction period (i.e., from initial contact to complete projectile detachment) as the actuation time (Fig. 1b and Supplementary Movie 1).”

Similar revisions have been applied throughout the manuscript, particularly in the Results and Discussion sections, details can be found in revised manuscript.

In addition, following the reviewer’s suggestion, we have checked all sentence in the main text, figure captions, and Supplementary Information and use “actuation time” to ensure consistent usage, which is the time period during which the projectile interacts with the hydrogel catapult before complete detachment.

Reviewer #2:

Wang et al. propose a soft actuator based on a high–energy density bilayer hydrogel. By leveraging dynamic coupling among actuator deformation, projectile motion, and substrate interaction, the system achieves high power output and rapid kinetic energy release. The resulting momentum transfer and power density enable fast, impulsive locomotion. The authors further demonstrate a potential application in fine-needle biopsy. Overall, the concept of using laser actuated hydrogel for soft actuator seems interesting. But there is some concern about the needs to justify the novelty compared with existing works. Below are further comments.

We thank the reviewer for the careful review and positive feedback. Your constructive comments have helped us to further improve our manuscript. We have addressed every comment and made corresponding changes to the manuscript. Please check the following point-by-point response for details.

1. Related work / positioning. The manuscript should discuss and compare against closely related hydrogel jump/roll actuators to better clarify novelty and performance. In particular, please consider: Li, M., Wang, X., Dong, B., and Sitti, M. (2020). In-air fast response and high speed jumping and rolling of a light-driven hydrogel actuator. Nature Communications, 11(1), 3988.

We thank the reviewer for highlighting this important and closely related work. We fully agree that a thorough discussion and comparison with Li *et al.* (*Nat. Commun.* 2020, 11, 3988) is essential to clarify the novelty and positioning of our study. We have added a discussion and cited this reference in the manuscript. The detailed discussion and comparative analysis are shown as follows,

Our work and Li *et al.* share a critical common foundation, i.e., employ NIR-light-driven photothermal heating to induce phase transition of water within hydrogel networks, harnessing the rapid volume expansion and pressure buildup for mechanical output. Li *et al.* made a pioneering contribution by demonstrating that this phase-transition mechanism

can achieve high-speed self-locomotion (jumping at ~1.6 m/s and rolling) of hydrogel actuators, establishing an important benchmark for the field and inspiring subsequent developments including our work.

However, while sharing the same fundamental actuation mechanism of hydrogel actuator, our work departs from Li *et al.* in a critical aspect: we address the previously unexplored scientific question of how to efficiently transfer the phase-transition-generated kinetic energy to external objects. This shift (i.e., from self-locomotion to projectile ejection) introduces entirely new physics (tripartite dynamic coupling) and unlocks performance regimes inaccessible to self-contained actuation systems. Importantly, the ~10 times velocity enhancement (16.6 m/s *vs.* 1.6 m/s) and the more than 100 times payload ability improvements are not achieved by modifying the phase-transition mechanism itself, but rather by the dynamic coupling strategy explored in this work, thereby demonstrating that coupling design, not merely material or energy input, is the key to unlocking the full potential of phase-transition-driven soft actuators. Our work differs fundamentally from Li *et al.* in core scientific questions and contribution, actuation mechanism, performance metrics and universality. To facilitate your review, we have summarized the key distinctions in the table below (Table R2.1), followed by an in-depth discussion of the conceptual and performance differences.

Table R2.1. Comparison between this work and Li *et al.* (*Nat. Commun.* 2020, 11, 3988).

**Aspect**	**Li *et al.*, *Nat. Commun.* 2020, 11, 3988**	**This work**
Core scientific question	How to achieve fast actuation? (The actuation of most hydrogel actuators is relied on diffusion dependency)	How to transfer energy efficiently and achieve Newton level force output to external objects in miniature scale
Core scientific contribution	Phase-change based actuation method in non-aquatic environments	Tripartite dynamic coupling framework for kinetic energy transfer in miniature scale
Functional objective	Self-locomotion (jumping/rolling of actuator body)	Projectile ejection (launching external objects)

Mechanism for best performance	Precise control of deformation position; increase energy input	System-level coupling matching
Performance	Take-off velocity: 1.6 m/s<br>Response time: 800 ms<br>Jumping height: 15 cm (75 body lengths)<br>Payload capacity: several times self-weight (n×7.95 mg)<br>Force output: 4.05 mN	Take-off velocity: 16.6 m/s (projectile)<br>Response time: 1.6 ms<br>Ejection height: 6.15 m (2050 body lengths)<br>Payload capacity: 0-1000 times self-weight (max is 200 g)<br>Force output: ~ 2.34 N
Universality	Hydrogel materials	Any soft materials with large deformation ability

(1) Core scientific questions and contributions

Li *et al.* addressed the fundamental challenge that conventional hydrogel actuators rely on osmotic-pressure-driven swelling and deswelling processes, which are inherently slow and typically require an aqueous operating environment. To overcome this limitation, they developed a miniature actuator capable of rapid jumping and rolling in air, featuring a core-shell architecture (i.e., an inner hydrogel core and an outer PBR rubber shell). The actuation relies on photothermal phase transition of the inner hydrogel, which generates microscale vapor bubbles that deform the outer rubber layer. The core contribution of Li *et al.* lies in establishing a phase-transition hydrogel material design framework and demonstrating a fast actuation principle that operates independently of an aqueous environment.

In contrast, our work addresses a distinct challenge: how to achieve efficient kinetic energy transfer and high force output in miniature soft robotic devices. We developed a dynamic coupling mechanism for soft catapult systems that enables high-performance ejection and force output. The output performance is governed by the interaction matching among three coupled elements: ultrafast deformation, the projectile's weight, and the substrate. Specifically, we demonstrate that: (a) Rigid substrates reflect downward vapor expansion and redirect energy upward, minimizing energy dissipation (Fig. 2a-b). (b) Interfacial adhesion anchors the catapult and enhances pressure confinement (Fig. 2c-d). (c) Projectile mass delays hydrogel network fracture and extends the interaction period, thereby increasing total momentum transfer (Fig. 2e-f).

The core scientific contribution of our work is a dynamic coupling framework applicable to high-power, high-force ejection output of miniature soft devices. Our contribution lies in the design of momentum transfer and conversion rather than in the fuel or the material itself. This explains why the same phase-transition mechanism yields qualitatively different capabilities when embedded in a properly designed coupled system.

(2) Functional objective

Li *et al.* demonstrated a hydrogel actuator capable of high-speed jumping and rolling, where the actuator body itself undergoes locomotion. The functional objective is to achieve macroscopic spatial displacement and efficient locomotion of the device itself, such as traversing obstacles. In contrast, our hydrogel catapult is designed to launch external projectiles while the catapult remains positionally stable on the substrate. As noted in our Introduction, ejection differs from jumping in that it enables high-speed projectile launch while maintaining positional stability of the catapult, that is a capability critical for non-contact remote operation and force delivery in confined spaces (e.g., biopsy).

In addition, although the macroscopic motion behaviors of jumping and ejection may looks similar, the energy cascade pathways and outputs involved are fundamentally different. Compared with jumping, ejection involves a substantially more complex energy transfer process with greater energy dissipation, governed by interfacial interactions, impedance matching, and dynamic coupling, these factors are entirely absent in self-contained locomotion systems. This is perhaps why, in the soft actuator community, jumping devices are abundant while catapult systems remain exceedingly rare[1,2] (e.g., *Adv. Funct. Mater.* 2023, 33, 2300156; *Adv. Mater.* 2025, 37, 2502669).

(3) Mechanism for best performance

The optimal jumping performance of the hydrogel actuator developed by Li *et al.* is achieved by increasing energy input and precisely controlling the deformation location so that the resulting force output acts entirely against the substrate to generate a reaction force, thereby yielding a high initial acceleration. This actuation paradigm is constrained by water diffusion kinetics and the strain energy storage capacity of the polymer network. In contrast,

our work addresses a fundamentally different coupled energy transfer problem, i.e., how to efficiently transfer kinetic energy from a soft body to an external object. The optimal ejection performance in our system arises from the tripartite dynamic coupling among the deformation, the substrate, and the projectile. By systematically exploring the governing interaction rules among these three elements, we enhance kinetic energy transfer efficiency and achieve high power and high force output.

(4) Performance comparison

While we acknowledge that jumping and ejection represent two distinct motion behaviors and that scale-dependent factors make certain performance parameters difficult to compare directly, we nevertheless present key metrics to provide the reviewer with an intuitive performance comparison. Enabled by the different physical actuation mechanisms, our system achieves order-of-magnitude improvements across multiple metrics: a 10.4 times increase in initial velocity (16.6 m/s *vs.* 1.6 m/s), a 500 times improvement in response speed (1.6 ms *vs.* 800 ms), a 27 times enhancement in motion height (6.15 m (2050 body lengths) *vs.* 15 cm (75 body lengths)), an approximately 100 times expansion in payload capacity (up to 1000 self-weight *vs.* several times self-weight), and an approximately 577 times increase in force output (2.34 N *vs.* 4.05 mN).

(5) Universality

The innovative phase-transition based actuation mechanism proposed by Li *et al.* is inherently tied to a specific material system, i.e., the water phase transition within hydrogels serving as the energy source. However, we must clarify that the dynamic coupling framework demonstrated in our work provides quantitative design guidelines that are generalizable to any soft catapult system, regardless of the specific driving mechanism. The light-driven hydrogel catapult employed in our main text serves as a representative and convenient example. In principle, any soft material that can undergo ultrafast deformation, whether actuated pneumatically, or by spark ignited combustion and other rapid energy release mechanisms, can achieve high performance momentum transfer and force output under the coupling framework established in this work.

In summary, we gratefully acknowledge that Li *et al.* established the foundational principle of phase-transition hydrogel-based actuation mechanism. Our work makes a distinct and complementary contribution by demonstrating that the dynamic coupling framework, rather than the actuation mechanism alone, is the critical determinant of output performance. Together, the two works paint a more complete picture of what phase-transition-driven hydrogel systems can achieve, i.e., Li *et al.* revealed the potential of phase transition for high-speed self-locomotion, while our work unlocks the possibilities that emerge when this energy is strategically coupled to external objects and substrates for high-power ejection.

We have added a discussion in the manuscript on page 7, line 131 as follows:

"Continued irradiation of NIR light further induces the phase transition of the water (i.e., from liquid to vapor) inside the hydrogel network rapidly (Fig. 1b and Supplementary Fig. 17). This phase-transition process be proved to achieve high-speed self-actuation of hydrogel device that founded by Li *et al*[43], which is a physical process rather than a chemical reaction, no chemical bonds are formed or broken during this process (Supplementary Fig. 18)."

On page 9, line 170 as follows:

"In this study, phase-transition-induced rapid deformation hydrogel catapult serves merely as a convenient and representative example of such an ejection method. In addition, high performance ejection motion is not achieved by modifying the phase-transition mechanism itself or materials system design, but rather by the dynamic coupling strategy during the actuation, which is the key to unlocking the full potential of phase-transition-driven soft actuators. In principle, any actuators capable of producing similarly fast and large deformation could leverage the coupling relationships explored in this work to achieve high-force ejection output."

2. Directional control. How is the jumping direction controlled? Does it depend on the direction/position of laser illumination, actuator geometry, or substrate anisotropy?

Please quantify directional repeatability (e.g., angular dispersion over n trials) and provide a controllability strategy if applicable.

The authors appreciate the reviewer's comments. In our system, the catapult direction is determined primarily by the applied position of the NIR light illumination (i.e., the deformation region) relative to the projectile's bottom surface, rather than by the actuator geometry or substrate anisotropy (Supplementary Fig. 36, as shown in Fig. R2.1). This enables programmable, contactless directional steering without redesigning the structure. Eccentrically positioning the phase-change region under the projectile creates an asymmetric contact and a net impulse whose horizontal component points away from the activation site. Consequently, if the interaction region is on the left side of the projectile's bottom surface, the projectile launches to the right. If it is on the right side, the projectile launches to the left.

In theory, substrate anisotropy does not influence the ejection directions, which primarily changes the travel distance and energy transfer efficiency. To quantify the directional repeatability, as the reviewer's suggestion, we launched a relatively heavy projectile (5.3 g, 265 times the hydrogel catapult's weight) and performed ten repeated actuations for the same target direction on both a glass substrate and a PDMS substrate (40:1), the recorded landing position and deflection angle as shown in Supplementary Fig. 36b and Fig. 36c (Fig. R2.1b and Fig. R2.1c). Compared with PDMS substrate, glass substrate induced the longer travel distance and better repeatability. The maximum angular dispersion of glass substrate and PDMS soft substrate was 16.7°and 23°, respectively. The potential reason of larger dispersion on PDMS is the compliance extends the interaction time and perturbs the initial velocity vector, thereby increasing angular spread.

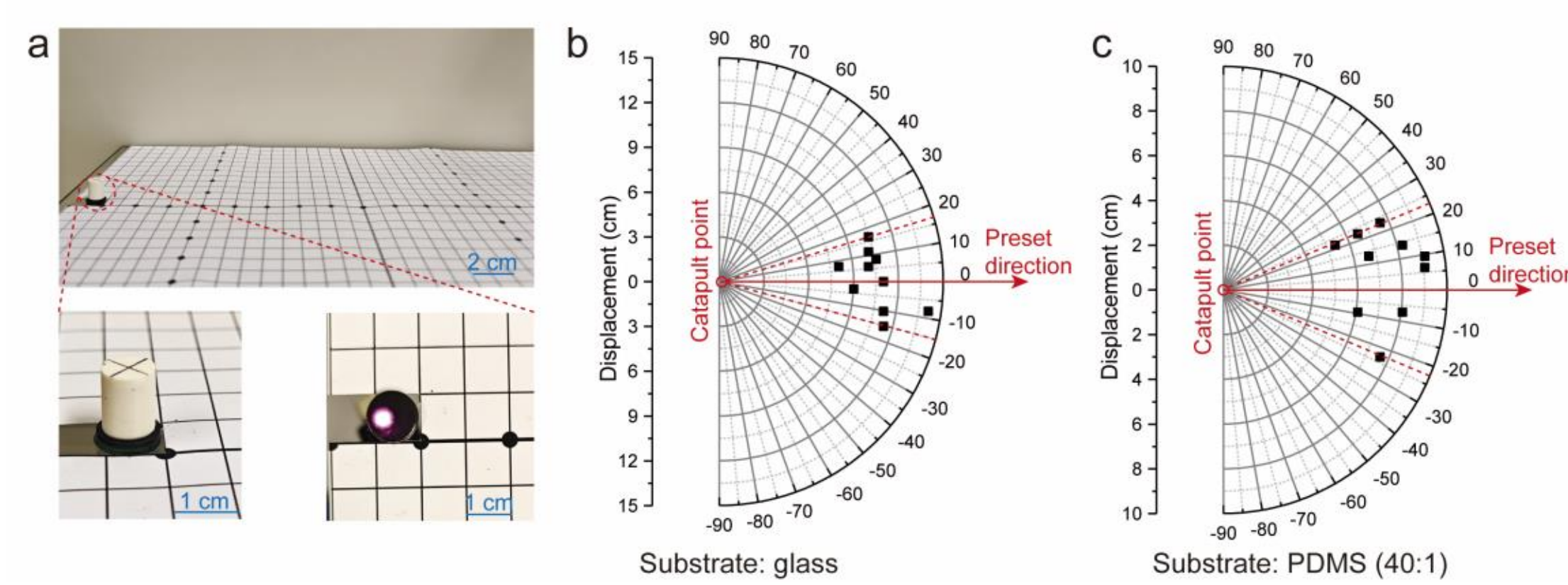

Fig. R2.1 (Supplementary Fig. 36). (a) The image showing the phase change region applied on the bottom surface of the projectile. (b) The angular dispersion and displacement of the 10 times actuation on the (b) glass substrate and (c) PDMS substrate (40: 1).

In addition, as the reviewer's suggestions, we provide the strategies to improve the controllability of the directional motion. (1) The optical fiber can be coupled to a micro positioning stage to precisely locate the phase-transition induced deformation region relative to the projectile's bottom surface. Under the same light intensity and substrate conditions, the force output is similar of the hydrogel catapult, thus, finer positional accuracy translates directly into finer directional control. (2) Integrate a patterned optical mask (selective transmission aperture) on the substrate so that light passes only through predefined regions, and add simple mechanical devices to control the projectiles on the same place. This ensures that the phase-change region consistently engages a fixed location on the projectile's bottom surface, enabling precise, repeatable launch directions. Overall, improved directional controllability stems from more accurate placement of the phase-change region relative to the projectile contact area.

Based on the above discussion, we have revised the description accordingly on page 21, line 360 as follows,

"In addition to the vertical and horizontal ejection capabilities of the hydrogel catapult, directional ejection motion can be realized by tailoring the position at which the output force is applied to the projectile, i.e., the deformation region relative to the projectile's bottom surface."

On page 21, line 374, as follows:

"The resulting actuation force enables precise ejection along the four cardinal directions (left, right, forward, backward) while maintaining uniform ejection height and travel distance (Fig. 4e and Supplementary Movie 5). The current directional control method exhibits the high repeatability and robustness, moreover, the property of substate shows limited influence on the direction control (Supplementary Fig. 36)."

3. Laser power dependence. How does laser power (and spot size/irradiance) affect jump height, velocity, and payload capacity? A parametric study linking optical input to mechanical output would improve rigor.

The authors appreciate the reviewer's comments. We agree that a systematic investigation of the influence of optical energy input on mechanical output enhances the rigor of the study. We have examined the effects of light intensity and spot size on ejection performance, and the corresponding results are presented in Supplementary Note 3. As the reviewer's comment, we have further analyzed the dependence of ejection height, velocity, and payload capacity on the optical energy input and have revised Supplementary Note 3 accordingly. Additionally, we have performed energy efficiency calculations (i.e., the ratio of kinetic energy to optical energy input) for the captured best ejection events, and the updated results are included in Supplementary Note 2.

We evaluated the influence of light intensity on ejection performance by measuring the ejection height of the projectile under varying illumination conditions. As shown in Supplementary Fig. 5a and Fig. 5b (Fig. R2.2a and b), the input of the NIR light energy shows significant influence on the ejection performance of the hydrogel catapult, a weak light intensity leads to the slow vaporization rate, prolonged irradiation period and limited deformation intensity and speed, that induces the reduced force output and energy transfer. The ejection height increases as the increases of the light intensity. However, it shows limited difference when the light intensity higher than 15 W/cm^2, potential reason is the sufficient light energy input in short period results in response time and deformation speed being very similar, which induced the similar power output. It is worth noting that under high-intensity illumination (i.e., >21.26 W/cm^2), the ejection performance exhibits greater

variability, as reflected by larger error bars. This may be attributed to an excessively rapid phase transition, which induces non-uniform deformation and consequently leads to variations in deformation speed and force output.

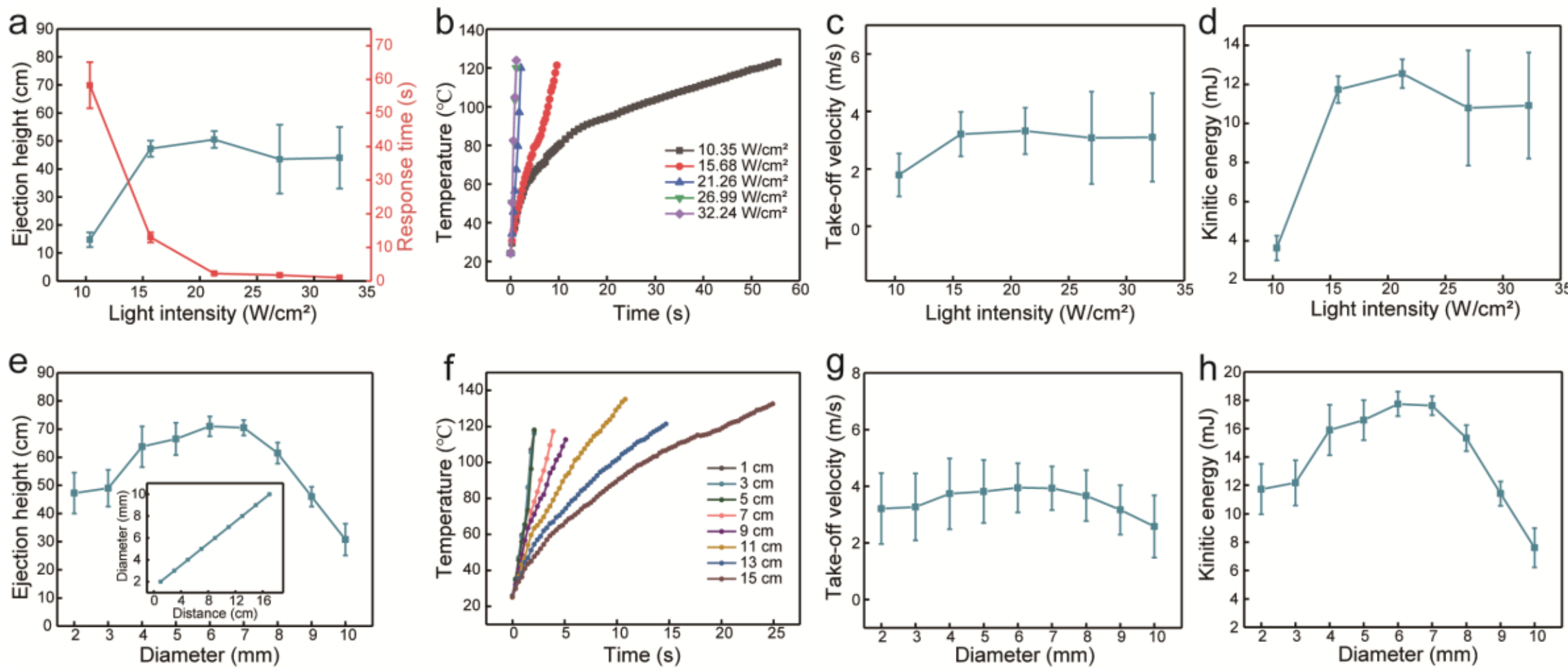

Fig. R2.2 (Supplementary Fig. 5). Light-dependent ejection performance of NIR-actuated hydrogel catapults. (a) The ejection height and response time of the hydrogel catapult with different light intensity. Data points are shown as mean ± s.d. (n = 4). (b) The temperature change of the upper layer for the catapult under NIR light irradiation with different light intensity. (c) The calculated take-off velocity and (d) kinetic energy of the ejected projectiles. (e) The ejection height of the hydrogel catapult with different diameter of light spot (Inset: The light spot diameter increases with the increase of the distance between light source and hydrogel catapult). Data points are shown as mean ± s.d. (n = 4). (f) The temperature change of the upper layer for the catapult under NIR light irradiation with different distance between light source and hydrogel catapult. (g) The calculated take-off velocity and (h) kinetic energy of the ejected projectiles. Note that the light intensity is 21.26 W/cm^2 and all the projectile's weight in Supplementary Fig 5 is 2280 mg.

Based on the recorded ejection height and accounting for air drag, we calculated the take-off velocity of the projectile, as presented in Supplementary Fig. 5c (Fig. R2.2c). The take-off velocity initially increases with rising light intensity and subsequently approaches a plateau. In addition, when considering the effect of light intensity on the payload capacity of the hydrogel catapult, it is important to recognize that the weight of the projectile influences the output dynamics (details shown in coupled framework proposed in Fig. 2 in the manuscript). As expected, an increase in projectile weight results in a macroscopic reduction in ejection height, which eventually approaches zero. However, under high-load conditions, the ejection output exhibits pronounced instability and is subject to the combined influences of both light intensity and projectile mass. Consequently, it is challenging to derive a precise quantitative reference for the maximum electable weight.

To address this, we qualitatively assess the payload capacity by evaluating the kinetic energy acquired by a projectile of fixed weight under varying light intensities. As illustrated in Supplementary Fig. 5d (Fig. R2.2d), the acquired kinetic energy increases with light intensity before reaching a plateau. This indicates that when the light intensity exceeds a certain threshold, the mechanical output of the hydrogel catapult stabilizes, and the corresponding payload capacity remains approximately constant.

In addition, the light spot area influences the power output by modulating both the phase transition volume and the response time. We adjusted the distance between the light source and the sample to vary the spot area (inset of Supplementary Fig. 5e (as shown in Fig. R2.2e)). As the spot area increases, the ejection height initially rises, which can be attributed to an enhanced force output resulting from a larger deformation volume. However, an excessively large spot area (i.e., diameter > 7 mm) leads to a reduction in optical power density and a prolonged response time (Supplementary Fig. 5f (as shown in Fig. R2.2f)), ultimately impairing both force output and ejection performance. A plausible explanation for this decline is that a larger spot area fails to induce a uniform phase transition across the entire irradiated region, thereby giving rise to heterogeneous deformation and inconsistent force output. Similarly, we calculated the initial velocity and kinetic energy of the projectile from the recorded ejection height, as shown in Supplementary Fig. 5g and Fig. 5h (Fig. R2.2g and Fig. R2.2h). Both the initial velocity and the acquired kinetic energy exhibit a non-monotonic dependence on the spot diameter, first increasing and then decreasing. The maximum payload capacity (i.e., the peak mechanical output) of the hydrogel catapult is achieved at a spot diameter of approximately 6 - 7 mm. A smaller spot size limits the overall deformation volume, whereas an excessively large spot size induces non-uniform deformation and premature structural failure, both of which compromise the payload capacity.

To further elucidate the relationship between optical energy input and mechanical output, we calculated the energy conversion efficiency for the captured best ejection event (as shown in Fig. 1c), in which a take-off velocity of 16.6 m/s was achieved under a light intensity of 21.26 W/cm^2. The corresponding results have been incorporated into Supplementary Note 2 and shown as follows.

"The kinetic energy is calculated to be 27.97 mJ based on the equation (9).

$$E_{ejection} = \frac{1}{2}mv^2 \quad (9)$$

The input energy (NIR light energy) can be calculated by:

$$E_{input} = P_{laser}t \quad (11)$$

where P_{laser} is the power of the applied laser, t is the laser irradiation time (~ 2s) before the deformation of the hydrogel catapult. The calculated external input energy is 21.36 J. The kinetic energy of the launched projectile is 27.97 mJ, which results in an energy efficiency of 0.13 %."

We have revised the Supplementary Note 2 and Note 3, details can be found in the revised Supplementary Information.

4. Scaling considerations. The manuscript demonstrates millimeter-scale actuators. What are the key limits and opportunities for scaling toward sub-millimeter dimensions (e.g., fabrication, heat/mass transfer timescales, mechanical robustness, and payload fraction)?

The authors appreciate the reviewer's comments. We agree that a further discussion of scaling effects is essential for understanding the applicable scope and future development of this mechanism. As noted by the reviewer, the primary limiting factor is the fabrication process. Currently, we prepare hydrogel catapult with a bilayer structure using centimeter-scale molds through a sequential curing and mold-cutting procedure. We have successfully realized the ejection motion through fabricated hydrogel catapult with a minimum dimension of approximately 1 × 1 × 0.8 mm, during the experiment, the collimator was used to reduce the spot size that enables precise alignment (Supplementary Fig. 34 (as shown in Fig. R2.3)). However, further reduction in size is difficult to achieve through manual cutting. Potential solutions include the adoption of micro modeling techniques or laser micromachining for the fabrication of hydrogel catapult with sub-millimeter dimensions. Moreover, given that the hydrogel system is photocurable, it is inherently

compatible with high-resolution 3D printing, which may enable the realization of micrometer-scale structures.

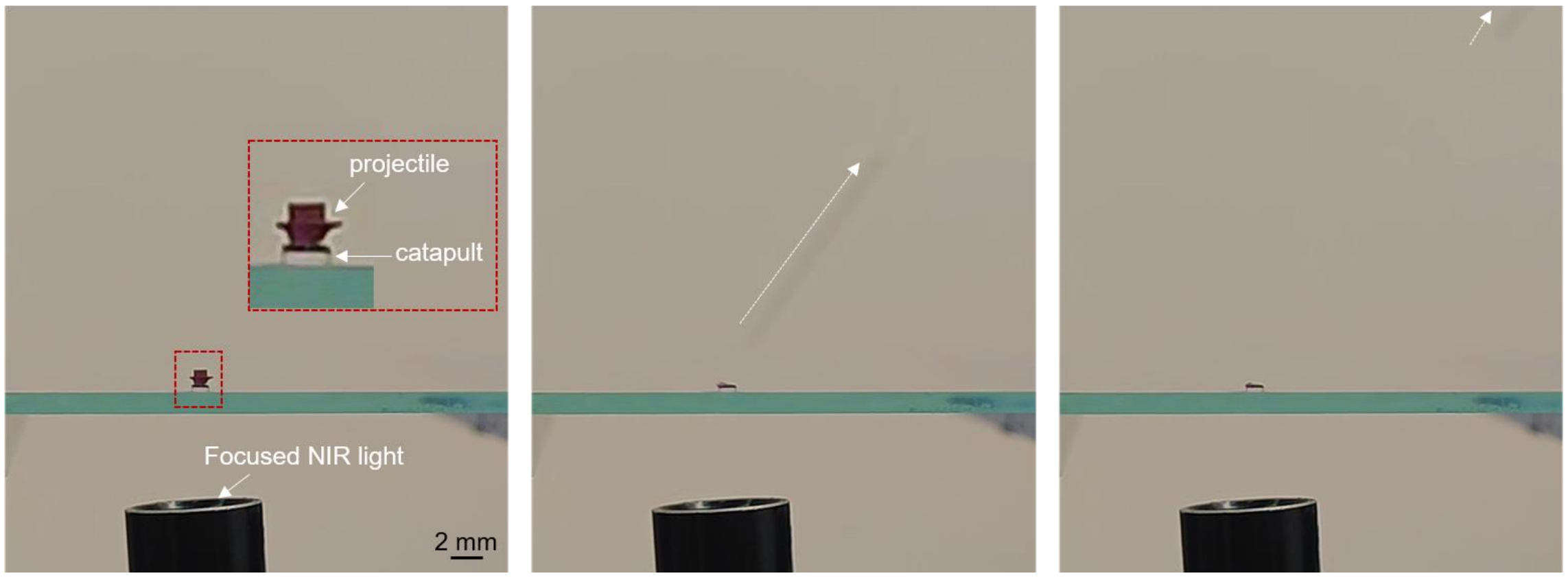

Fig. R 2.3 (Supplementary Fig. 34). The screenshots showing the ejection process of the hydrogel catapult with dimension of approximately 1 × 1 × 0.8 mm.

In addition, based on the proposed actuation mechanism and the dynamic coupling interaction between the hydrogel catapult, the substrate, and the projectile, achieving a phase transition that initiates internally and generates directional, rapid deformation at sub-millimeter scales poses challenges for the auxiliary equipment, specifically with respect to the light source dimensions and alignment precision. Therefore, either structural design improvements or a reduction in the light source size are required to induce internal phase transition and directional deformation. While advances in actuation strategies and fabrication techniques have facilitated the miniaturization of hydrogel catapult, the feasibility of achieving such phase transition behavior at reduced dimensions warrants further validation.

Furthermore, as the characteristic dimension decreases, the thermal conduction time scale is governed by $\tau \sim \frac{L^2}{\alpha}$, where L is the characteristic length and α represents the thermal diffusivity of the material. Consequently, temperature equilibration occurs more rapidly, enabling faster onset of the phase transition. Nevertheless, this is accompanied by accelerated heat dissipation into the surrounding environment, thus necessitating a higher power density of near-infrared light to supply sufficient energy for phase transition initiation. Moreover, owing to the reduced dimensions, the vapor generated during heating

may escape more readily from the hydrogel network rather than accumulating sufficient internal pressure. This issue may be addressed by further adjusting the crosslinking density of the hydrogel network or through encapsulation strategies to facilitate pressure buildup. It should be noted that modifications to the hydrogel network will inevitably alter its mechanical properties, the hydrogel matrix with higher toughness is conducive to greater energy accumulation, yet tailoring these mechanical characteristics concurrently introduces additional challenges.

Third, we have conducted analysis of the payload fraction that considered scaling effect, the payload fraction (β) can be expressed as:

$$\beta = \frac{F_{output}}{m_{projetile}g} = \frac{PA}{\rho V g} \sim \frac{PL^2}{\rho L^3 g}$$

where P is the pressure, A is the force application area, L is the characteristic length scale, and ρ is the density of the projectile. This relationship indicates that the payload fraction β is inversely proportional to the characteristic length L^{-1}. Therefore, the payload fraction increases as the L decreases, it is consistent well with observations in naturally occurring miniature ejection systems, where acceleration values in micro-scale catapult far exceed those in macroscopic counterparts. Consequently, further size reduction theoretically enables the attainment of higher accelerations.

We are fully agree with the reviewer that a comprehensive consideration of scaling effects is crucial for further miniaturization of the hydrogel catapult and for understanding the technology's potential development. Based on the above discussion, the primary challenges in achieving high-performance ejection at reduced dimensions are the fundamental limitation of phase transition feasibility, followed by fabrication precision and structural robustness, as well as the difficulty in pressure accumulation due to vapor escape at the sub-millimeter scale. Simultaneously, opportunities arise from enhanced thermal transport and an increased payload fraction, which may yield superior motion performance and faster actuation speeds. However, detailed experimental exploration of these scaling aspects lies beyond the scope of the present work. We would like to clarify that the central focus of this study is the dynamic coupling relationship among the catapult, the substrate,

and the projectile during the actuation process. The proposed dynamic coupling framework provides a universal guiding principle for achieving high-performance ejection behavior, independent of material selection and scale considerations. Any micro-actuator capable of generating rapid, large-magnitude deformation can potentially leverage this coupling relationship.

We have added the above Figure into Supplementary Information and added a discussion in the manuscript on page 18, line 335 as follows:

"The above demonstration validates the excellent ejection performance of the catapult enabled by the dynamic coupling framework. It not only exhibits wide applicability for different projectiles but also allows the catapult size to be adjusted according to the dimensions of the ejected objects. We have achieved a minimal hydrogel catapult (1 × 1 × 0.8 mm) along with high-speed ejection behavior (Supplementary Fig. 34), thereby proving the broad adaptability and size-independence of the proposed dynamic coupling framework."

5. Operation in aqueous environments. Can the actuator function underwater or in wet conditions? If not, please discuss the primary failure modes (e.g., optical attenuation, altered friction, swelling kinetics, damping) and what design changes would be required.

The authors appreciate the reviewer's comments. As suggested by the reviewer, we have measured the feasibility of underwater ejection motion of the hydrogel catapult. Specifically, we fabricated a hydrogel catapult and a 3D-printed projectile to evaluate the ejection performance in an aqueous environment, as illustrated in Supplementary Fig. 37 (Fig. R2.4). To maintain optical transparency, the entire ejection system was placed in a transparent container, and NIR light was directed from the bottom to irradiate the hydrogel catapult. The NIR irradiation induced the phase transition and deformation of the hydrogel catapult, which in turn propelled the projectile at a high velocity.

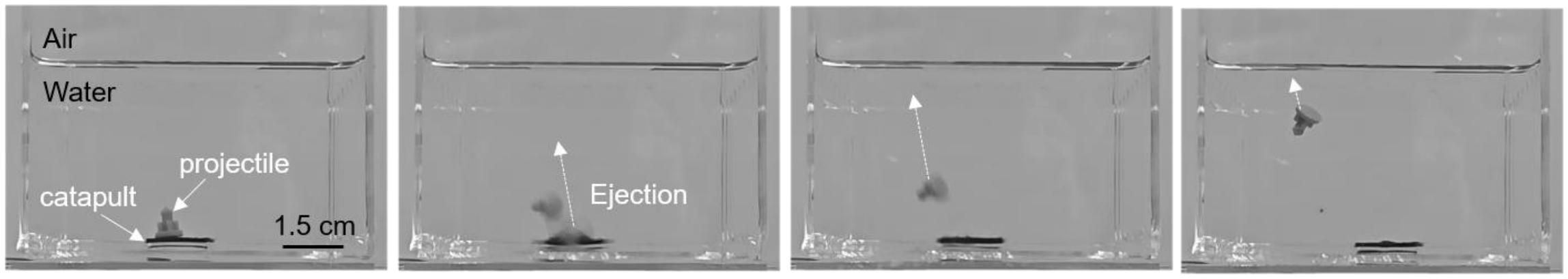

Fig. R2.4 (Supplementary Fig. 37). The screenshots showing the ejection process in an aquatic environment.

Although the hydrogel catapult can work well in the water environment, the ejection performance is substantially attenuated compared to that operation in air. This attenuation is primarily attributed to the reduction of the adhesion force at the interface between hydrogel catapult and substrate. The aqueous environment prevents the formation of hydrogen bonds between the substrate and hydrogel catapult, thereby compromising stable adhesion. As a result, displacement and deformation of the hydrogel at the base lead to significant energy dissipation, which in turn reduces both the speed and the magnitude of the upward deformation. This observation is consistent with the dynamic coupled mechanism proposed in our study and the results shown in Fig. 2 in the manuscript. Furthermore, the projectile encounters considerably greater viscous resistance when moving through water, as the damping coefficient of water is approximately 50 times higher than that of air. This enhanced damping leads to rapid dissipation of the projectile's kinetic energy.

If we want to realize the ejection motion in aqueous environments, complete encapsulation to isolate the device from water represents a promising solution. Indeed, as demonstrated in Fig. 5, we have demonstrated the PDMS-encapsulated biopsy devices which successfully achieved forceful ejection-based sampling in a wet porcine stomach, thereby confirming the feasibility of this approach.

We have revised the manuscript on page 24, line 414 as follows:

"The system incorporates a sampling needle with a larger diameter than traditional fine needles to increase single-sample volume, which is mounted on a 3D-printed base attached to the hydrogel catapult. The hydrogel catapult exhibits normal operation in aqueous or wet environments, which guarantees the feasibility of use in wet or mucous environments inside the body (Supplementary Fig. 37). However, the presence of water reduces the

adhesion between the hydrogel catapult and the substrate, leading to performance degradation. A customized PDMS shell is utilized to prevent NIR light leakage, ensure stable adhesion between the catapult and the substrate, and protect the samples from contamination."

6. Biopsy demonstration—clinical advantage. Figure 5 shows biopsy via needle punching driven by the actuator. What is the advantage of this approach compared with existing biopsy tools (manual/robotic fine-needle aspiration, spring-loaded biopsy devices, or catheter-based systems)? Please clarify the intended clinical scenario and provide comparative discussion (precision, delivered impulse, access, safety, and potential tissue trauma).

The authors appreciate the reviewer's comments, which helps us better clarify the intended clinical positioning and advantages of our hydrogel catapult-based biopsy system. We have revised the manuscript and added a comprehensive comparative discussion in Supplementary Information.

1. Clarification of intended clinical scenario

We wish to first clarify that the biopsy demonstration serves as a proof of concept to validate the force output and kinetic energy transfer capability of the hydrogel catapult in a medically relevant context, rather than claiming a clinically ready device. The intended clinical scenario is deep submucosal tissue sampling within the gastrointestinal (GI) tract, where conventional endoscopic tools face inherent limitations in force delivery, end force output, and single sampling volume.

In current clinical practice, endoscopic tissue sampling relies primarily on biopsy forceps and endoscopic ultrasound-guided fine-needle aspiration/biopsy (EUS-FNA/FNB). However, biopsy forceps acquire only superficial mucosal tissue, limiting diagnostic depth. EUS-FNA/FNB enables deeper sampling but is constrained by fine needle gauges (typically 19-25G)[3], often requiring multiple passes (typically 3-7 times) to obtain adequate

diagnostic material[4], which increases procedure time, patient discomfort, and complication risk.

It is important to note that although spring-loaded biopsy guns widely used in percutaneous applications that can deliver high forces and acquire core tissue samples with thick needles (14-18G)[5], they are designed exclusively for external, percutaneous use (e.g., breast, liver, and kidney biopsy). Their rigid construction, bulky firing mechanism, and requirement for transcutaneous needle insertion make them fundamentally incompatible with intraluminal GI tract procedures[5]. Consequently, for GI tract deep tissue sampling, clinicians must rely on endoscopic tools (biopsy forceps, FNA/FNB) whose force output and sampling adequacy are inherently limited by the constraints of endoscopic delivery.

This reveals a critical unmet need: Apply sufficient force within the GI tract to actuate the thicker sampling needle to obtain the sufficient samples in a single time biopsy. Our hydrogel catapult-based system directly addresses this gap by decoupling force generation from endoscopic transmission, providing sufficient instantaneous force output (~2.34 N) to drive a thicker needle into deep tissue in a single actuation event within a compact intraluminal device.

2. A particularly challenging clinical scenario: retroflexed endoscopy in the upper stomach.

To further illustrate the clinical relevance of our approach, we highlight a particularly challenging yet common clinical scenario that exemplifies the limitations of conventional endoscopic biopsy tools: tissue sampling in the gastric fundus and cardia under retroflexed (inverted) endoscopy.

The gastric fundus is widely recognized as a “blind spot” in standard forward-viewing endoscopy. To visualize and access this region, endoscopists must perform a retroflexion maneuver, i.e., inverting the endoscope tip within the gastric lumen to look back toward the upper stomach. While this technique enables visualization, performing precise deep tissue sampling in this configuration presents three well-documented categories of challenges: (i) Visual-spatial disorientation. In retroflexion, the endoscopic image is completely inverted relative to the operator’s hand movements, creating a mirror-image

coordination problem.[6] The confined space of the gastric fundus further restricts endoscope maneuverability, making it extremely difficult to identify optimal puncture sites and maintain stable positioning during needle insertion. (ii) Compromised force transmission. Conventional biopsy tools (forceps, FNA/FNB needles) transmit puncture force from the operator's hand through the entire length of the endoscope to its tip. In the retroflexed configuration, the sharply curved endoscope shaft severely attenuates force transmission efficiency. The resulting "reverse-angle" geometry often prevents the needle from achieving perpendicular contact with the gastric wall, leading to needle slippage, oblique insertion angles, and inadequate tissue penetration. (iii) Heightened perforation risk. The gastric fundus has a relatively thin wall compared to other gastric regions. Clinical studies have reported significantly elevated perforation rates during endoscopic submucosal dissection (ESD) in the gastric fundus compared to other gastric locations (reported rates vary from ~5 % to over 15 % depending on lesion characteristics, substantially higher than the 1-4 % observed in other regions)[7,8], underscoring the vulnerability of this region to mechanical injury. While biopsy involves less tissue disruption than ESD, the underlying risk principle is any loss of depth control during needle insertion in this thin-walled region poses a significant perforation hazard. The proximity of critical structures, including the diaphragm, spleen, and short gastric vessels, further compounds the consequences of inadvertent deep puncture.

3. The hydrogel catapult-based biopsy system for addressing the challenges discussed above.

(i) Eliminating the force transmission requirement. Unlike conventional tools that must transmit puncture force through the entire curved endoscope, our device generates force locally at the tissue interface through phase-transition-driven actuation (~ 2.34 N). This eliminates force attenuation caused by endoscope curvature, enabling consistent and sufficient penetration force regardless of the endoscope's configuration. This "local force generation at the end" paradigm fundamentally circumvents the mechanical limitations of remote force transmission through flexible instruments.

(ii) Reducing doctor's reliance on operational skills. The puncture depth and needle trajectory in our system are mechanically predetermined by the shell structure (stepped structure and guide channel as shown in Fig. 5a (Fig. R2.5)), rather than relying on the operator's real-time manual control under visually disorienting retroflexed conditions. This structural predetermination significantly reduces the doctor's operational skills in conventional retroflexed biopsy procedures.

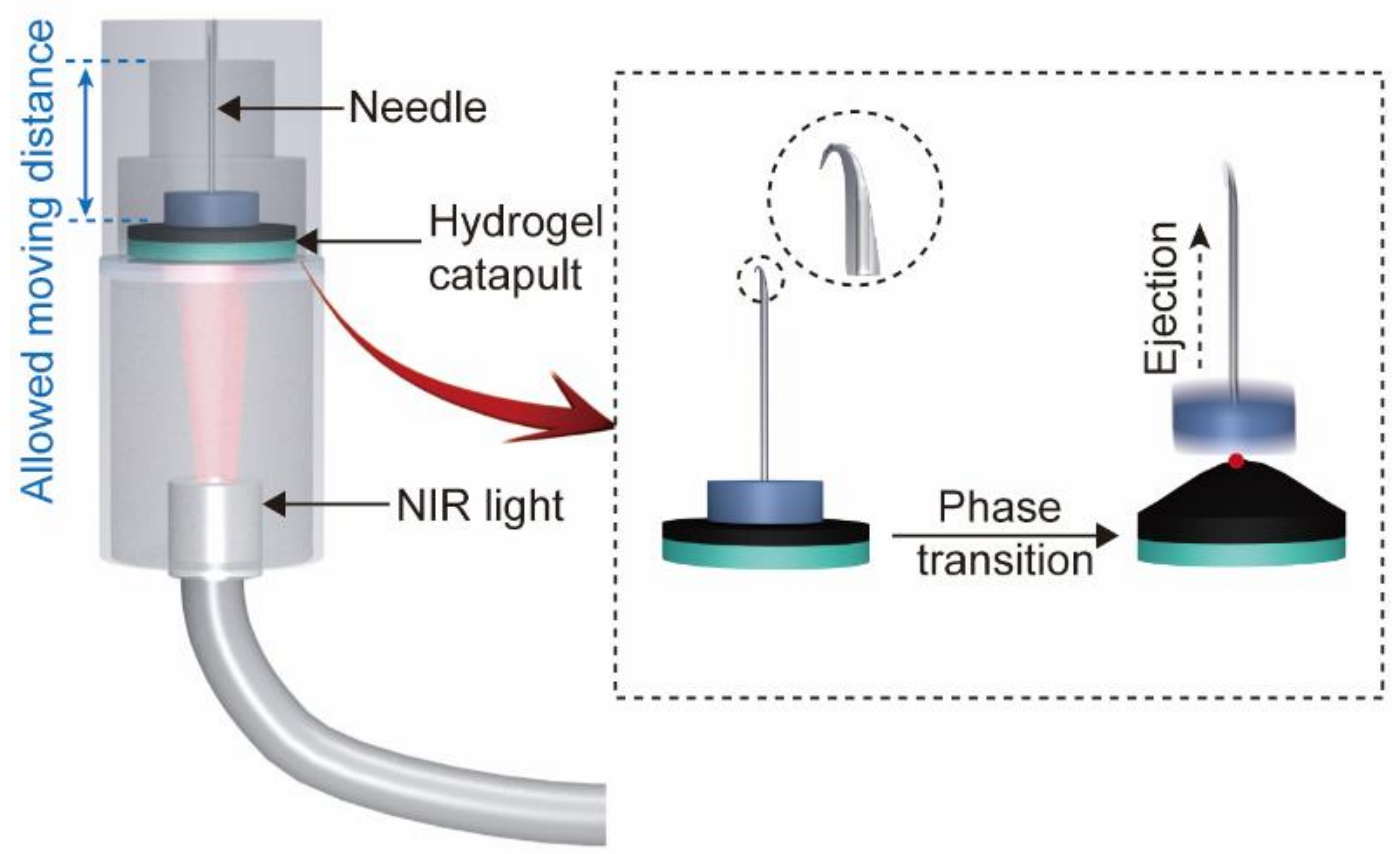

Fig. R2.5 (Fig. 5a). Schematic illustration of the structure and working principle of the developed biopsy robot.

(iii) Mitigating perforation risk through stepped structure enabled depth control. The stepped structure provides a hard physical stop that prevents over-penetration regardless of applied force or operator error, i.e., the insertion depth can be physically controlled (as shown in Fig. 5a (Fig. R2.5)). This physically controlled principle is especially critical in thin-walled regions such as the gastric fundus, where even small excesses in penetration depth can lead to perforation. In contrast, conventional manual biopsy relies entirely on the operator's tactile judgment for depth control, which is significantly compromised under retroflexed operating conditions.

4. Systematic comparison with existing biopsy tools

As the reviewer's suggestions, we provide a detailed comparison with existing biopsy tools across the five dimensions, as shown in Table R2.2 (Supplementary Table 3), the comparison reveals a fundamental trade-off in the current landscape, i.e., devices with sufficient force for deep tissue penetration (spring-loaded biopsy guns) are restricted to

percutaneous applications and cannot access the GI tract intraluminally, while devices compatible with intraluminal delivery (FNA/FNB, soft robotic systems) lack the force output for single-pass deep tissue acquisition. Our system is designed to bridge this gap.

Table R2.2 (Supplementary Table 3). Comparison of the hydrogel catapult biopsy system with existing biopsy tools.

**Aspect**	**Manual FNA/FNB**	**Spring-loaded biopsy devices**	**Catheter-based systems**	**This work**
Force output	~ 1-5 N[9] (operator dependent)	High (spring dependent)	Typically < 0.5 N;	2.34 N
Force generation mode	Remote (transmitted through endoscope)	Remote (spring pre-loaded externally)	Remote (transmitted through endoscope)	Local (generated at tissue interface)
Needle gauge	19-25 G[3,4]	14-18 G[5] (mainly for percutaneous biopsy)	19-25 G[10]	Customizable; The local force output allows the needle thicker than 14G
Single-pass adequacy	Often insufficient; typically 3-7 passes required	High (core tissue obtained)	Often insufficient	High (thicker needle and sufficient force)
GI tract intraluminal access	Yes	Not applicable	Yes	Yes
Depth control	Operator tactile judgment	Yes	Varies	Mechanical depth stop
Actuation speed	Manual (~s)	Fast (~ms); (mainly for percutaneous biopsy)	Typically slow (~s)	Fast (ms)

(i) Delivered impulse and force output. The hydrogel catapult delivers ~2.34 N of instantaneous force (Supplementary Fig. 22), falling within the clinically relevant range for soft tissue needle insertion (1-5 N)[9]. To place this in context, it is instructive to examine the landscape of existing biopsy tools. Spring-loaded biopsy guns can generate substantial

forces through pre-loaded spring mechanism, however, these are rigid, bulky handheld devices designed exclusively for percutaneous procedures (e.g., breast, liver, kidney biopsy) and are fundamentally incompatible with intraluminal GI tract access. They cannot be miniaturized to a form factor suitable for endoscopic delivery, nor can they be used within the GI lumen under any clinical scenario. On the other end of the spectrum, tools that can access the GI tract intraluminally, including EUS-FNA/FNB needles and catheter-based robotic systems, are limited to fine needle gauges and modest force output (typically < 0.5 N for soft robotic systems, and while manual FNA can nominally deliver 1-5 N, this force is significantly attenuated when transmitted through a curved endoscope, particularly under retroflexion)[9]. Our hydrogel catapult-based biopsy system bridges this critical gap by providing Newton-level, locally generated force (~ 2.34 N) that can be delivered into the GI lumen, a combination of force output and intraluminal accessibility that is currently unavailable in clinical practice.

(ii) Access, precision and penetration control. The key advantages of our device lie in its high output force and endoscope compatibility. Furthermore, our device can be further miniaturized, potentially enabling integration into wirelessly driven capsule robots. Penetration precision is achieved through two complementary mechanisms: (1) a stepped structure within the PDMS shell that mechanically limits the maximum needle travel distance, providing a hard physical stop analogous to the depth-limiting mechanisms in percutaneous biopsy guns, but now implemented in an intraluminal device. (2) A guide channel with a slightly larger diameter than the needle that corrects needle orientation during ejection, ensuring vertical insertion and reducing angular misalignment (as shown in Fig. 5a (Fig. R2.5)). These mechanical constraints provide reliable, operator-independent depth and directional control, which is valuable in the retroflexed configuration where manual precision is most severely compromised. Furthermore, the system is compatible with real-time endoscopic imaging for spatial guidance to the target site. While we acknowledge that the current directional precision is not yet comparable to dedicated robotic needle guidance systems, the mechanical constraints provide a robust baseline level of penetration control suitable for the proof-of-concept stage.

(iii) Safety and thermal considerations. Although the internal temperature of the graphene-doped hydrogel layer exceeds 120 °C during phase transition, the PDMS encapsulation provides effective thermal isolation. We used infrared thermography to record the temperature variation on the device surface (i.e., the surface in contact with the tissue) during actuation. The measured peak temperature at the robot-tissue interface was 40.3 °C as shown in Supplementary Fig. 12 (Fig. R2.6), which is below the widely accepted threshold for thermal tissue damage (generally defined as > 43 °C for sustained exposure exceeding 60 minutes, or > 50 °C for acute thermal injury)[11]. Moreover, the actuation event lasts only ~1.6 ms, resulting in negligible cumulative thermal dose to surrounding tissue. Additional safety features include the PDMS shell prevents NIR light leakage to tissue. The optical fiber is enclosed within the sealed structure, eliminating direct light exposure to tissue. And the mechanical depth limiter provides an additional critical safety layer, particularly important in thin-walled anatomical regions such as the gastric fundus where uncontrolled penetration depth poses significant perforation risk.

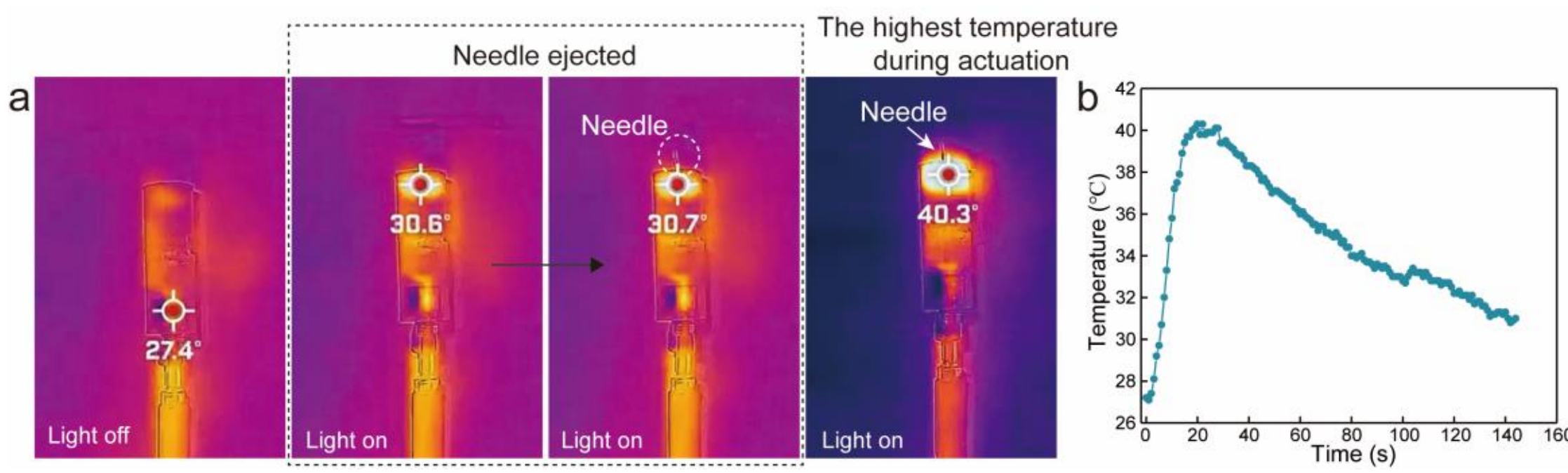

Fig. R2.6 (Supplementary Fig. 12). (a) A series of infrared thermal images and (b) temperature change profile showing the surface temperature of the device during the biopsy procedure.

(iv) Potential tissue trauma. We acknowledge that the use of a thicker needle inherently creates a larger puncture wound compared to fine-needle aspiration. However, this trade-off may be mitigated by two factors. First, the ultrafast actuation (~1.6 ms) means the needle reaches full insertion depth before significant tissue deformation occurs. Published studies on needle insertion biomechanics have demonstrated that higher insertion velocities reduce tissue displacement, dimpling, and associated mechanical trauma compared to slow manual insertion[12,13]. Second, the thicker needle enables adequate tissue acquisition in a single pass, potentially eliminating the need for multiple punctures. Furthermore, following

the reported module design principle[14], the hydrogel catapult is expected to be integrated into the repair module to avoid potential adverse consequences such as bleeding.

In summary, in the landscape of tissue sampling tools, it remains necessary to develop devices with sufficient force for deep tissue penetration and suitable integrated with endoscopy to acquire sufficient samples in single time biopsy. The hydrogel catapult-based biopsy system demonstrated in this work represents a first step toward resolving this challenge, by introducing a “local force generation” method that enables Newton-level force output within a miniaturized, endoscopically deliverable form factor. This capability is enabled by the dynamic coupling mechanism, the core scientific contribution of this work, which provides the physical foundation for achieving high power and force output in miniature soft systems.

In addition, as mentioned before, this biopsy device is at the proof-of-concept stage and has not undergone systematic biocompatibility or safety testing required for clinical translation. The purpose of this system we have developed is to help better demonstrate our core contribution, i.e., the dynamic coupling framework, and to better help readers understand how this mechanism enables the miniature robots achieve efficient remote and non-contact operation in confined spaces.

We have revised the manuscript on page 24, line 402 as follows:

“Biopsy is crucial for precision medicine, early disease detection, and patient stage monitoring. Currently, fine-needle aspiration biopsy (FNAB) and fine-needle capillary biopsy (FNCB) are widely employed for subsurface tissue sampling in endoscopic procedures[16,46]. However, due to the capacity of the fine needle, multiple punctures (usually 3-7 passes) are often required to obtain sufficient diagnostic material, which increases procedure time, patient discomfort, and the risk of complications. For regions that are difficult to access directly, such as gastric fundus sampling, the endoscope must be retroflexed, and the puncture force is transmitted from the operator’s hand through the entire length of the endoscope to its tip, resulting in insufficient force or inaccurate puncture. Therefore, using a thicker needle to obtain sufficient samples and generating

driving force at the tip are beneficial for reducing the need for repeated surgical maneuvers and improving precision."

In addition, we have added the above Table and corresponding descriptions in Supplementary information as Supplementary Note 7 and revised the manuscript on page 25, line 446 as follows,

"The demonstrated biopsy process and application scenario indicate the effectiveness of this hydrogel catapult-based biopsy system. We also conducted a detailed comparison between our developed system and clinically used instruments to demonstrate the translational potential of our strategy. Details can be found in Supplementary Note 7."

7. Substrate and payload effects. How does launch height (and/or distance) vary with substrate properties (roughness, stiffness, friction coefficient) and payload mass? A quantitative characterization would help assess real-world robustness and application feasibility.

The authors appreciate the reviewer's comments. We agree that a systematic quantitative characterization of substrate properties and payload mass effects is essential for assessing the robustness and application feasibility of the hydrogel catapult system. We note that our original manuscript already contained substantial data addressing several of these parameters (Fig. 2 and Supplementary Fig. 25 - Fig. 31), as the reviewer's suggestion, we have further significantly strengthened characterization with additional experiments and analysis in the revised manuscript, as detailed below.

1. Stiffness of the substrate

In our original manuscript, we compared the ejection dynamics under three substrate conditions (i.e., rigid (glass), flexible (PDMS with monomer-to-curing-agent ratio of 40:1), and free-standing (no substrate)), demonstrated that rigid substrates produce superior ejection performance through minimized energy dissipation and enhanced airflow reflection (Fig. 2a-b shown as follows and Supplementary Note 4).

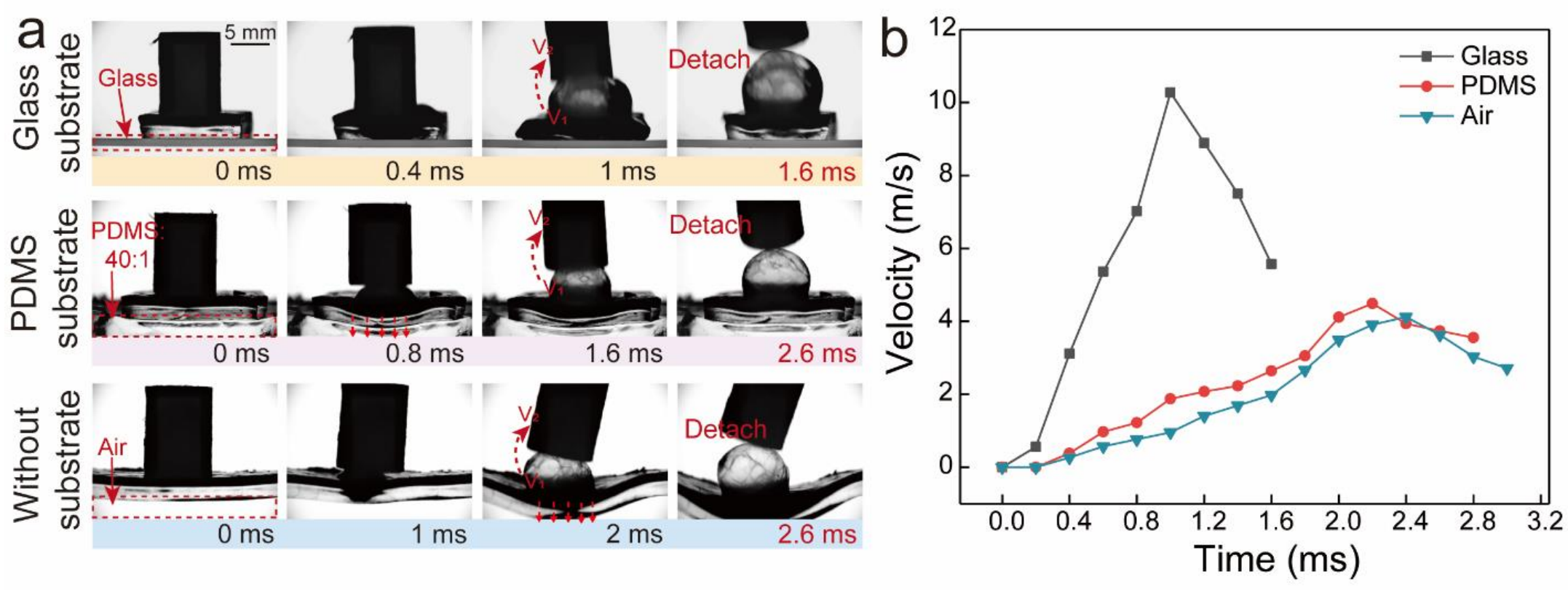

Fig. R2.7 (Fig. 2a and 2b). (a) The deformation and response time comparison for hydrogel catapults on glass substrate, PDMS substrate and free standing (without substrate). Note the weight of the projectile is 380 mg. (b) Projectile velocity profiles during actuation on the substrate of glass, PDMS, and free-standing.

Following the reviewer's suggestion, we conducted further experiments to establish a quantitative relationship between substrate stiffness and ejection performance. Specifically, we fabricated PDMS substrates with varying Young's modulus by adjusting the monomer-to-curing-agent ratio, and measured ejection heights and take-off velocity on these substrates. As shown in Supplementary Fig. 27a (Fig. R2.8a), the stiffness decreased as the increase of monomer-to-curing-agent ratio. The ejection height and take-velocity increase monotonically with increasing substrate stiffness (Supplementary Fig. 27b (as shown in Fig. R2.8b)). We also performed velocity analysis based on high-speed camera recordings (Supplementary Fig. 27c (as shown in Fig. R2.8c)). The results consist with the ejection height result that indicates rigid substrates produce superior ejection performance. The underlying reason is that compliant substrates undergo significant elastic deformation upon impact from the phase-transition-induced pressure, leading to substantial energy dissipation. This reduces both the reflected airflow that drives upward hydrogel deformation and the efficiency of kinetic energy transfer to the projectile. In practical applications, higher substrate stiffness results in lower energy loss and correspondingly better ejection performance. Therefore, non-deformable materials such as glass represent the preferred choice for the ejection launch platform.

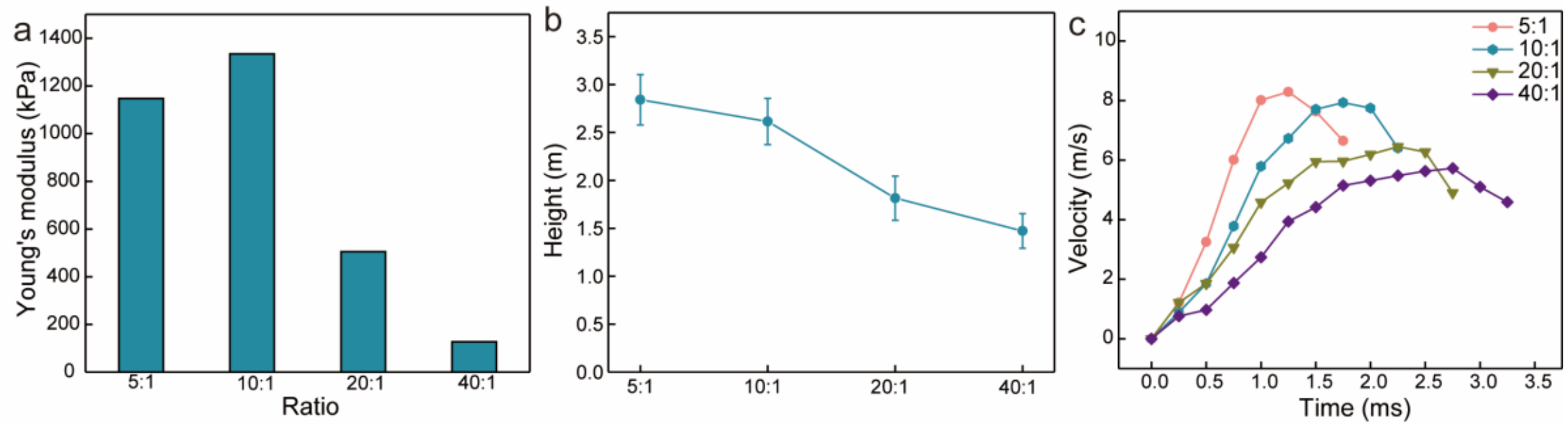

Fig. R2.8 (Supplementary Fig. 27). (a) The Young's modulus of the fabricated PDMS substrate with different ratio of the monomer and curing agent that obtained from stress-strain curve. (b) The ejection height and (c) projectile velocity profiles during actuation on the substrate of PDMS with different ratio of the monomer and curing agent. Note that the mass of projectile is 380 mg.

2. Interfacial adhesion and surface roughness

In our original manuscript, we demonstrated the critical role of interfacial adhesion through comparative experiments on glass, PET, and PE substrates with and without water lubrication (Fig. 2c - Fig. 2d shown as follows), adhesion force measurements (Supplementary Fig. 31), and stability analysis (Supplementary Fig. 29 - Fig. 30). The results established that higher interfacial adhesion force leads to improved ejection performance. Interfacial adhesion plays an essential role in the ejection system, it prevents the catapult from displacing during the phase-transition-induced impact, thereby reducing kinetic energy loss.

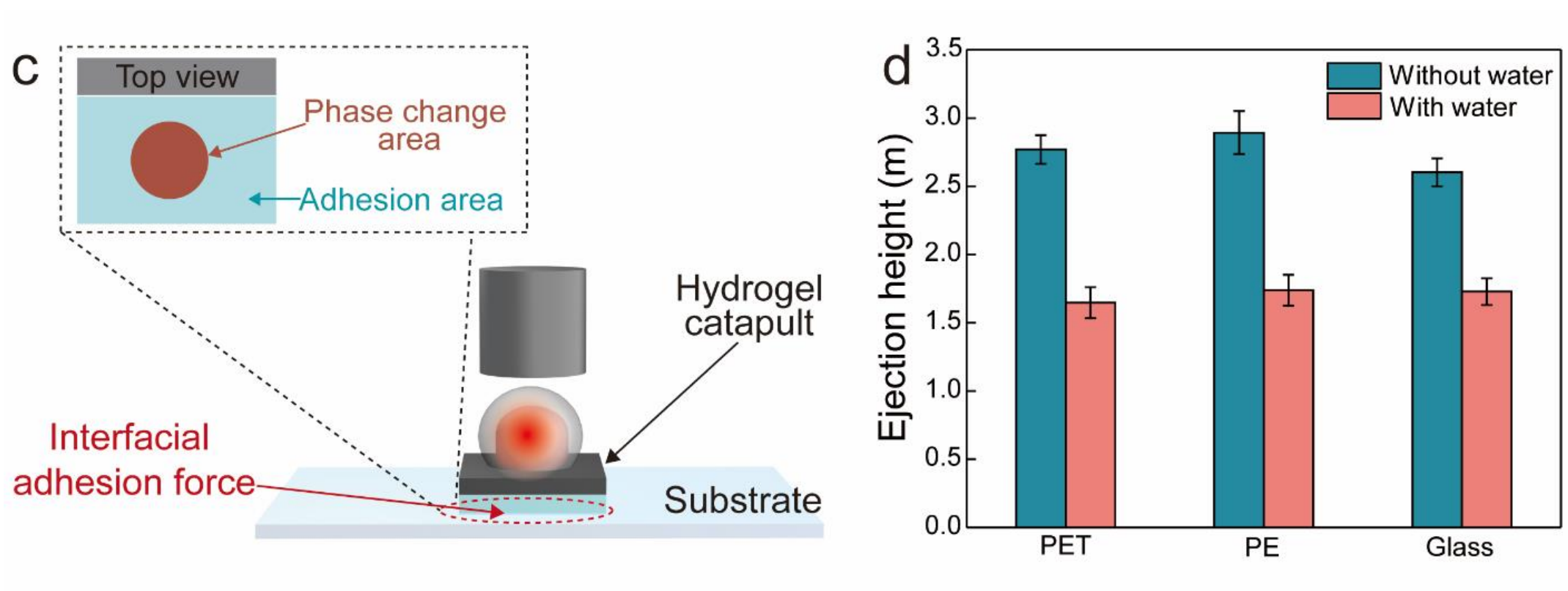

Fig. R2.9 (Fig. 2c and 2d). (c) Schematic of interfacial adhesion force between hydrogel catapult and substrate. (d) Ejection height comparison with/without water lubrication (blocks interfacial adhesion) across different substrates. Data points are shown as mean ± s.d. ($n = 4$)

Interfacial adhesion and surface roughness are closely correlated. As shown in the Supplementary Fig. 32 (Fig. R2.10), under conditions where the substrate material is held constant, rougher surfaces generate a smaller real contact area compared to smooth surfaces, which in turn leads to reduced adhesion force and consequently diminished ejection performance. We note that it is difficult to define a single quantitative threshold value for adhesion force or roughness that universally dictates ejection performance. Rather, our experimental data indicate that optimal ejection performance requires the catapult to remain positionally stable throughout the actuation process. In principle, under identical initial conditions, as long as no significant displacement of the hydrogel catapult occurs during actuation, the ejection performance remains comparable. Therefore, increasing interfacial adhesion and reducing surface roughness are both conducive to achieving optimal ejection performance, as they collectively ensure the positional stability of the catapult during the ultrafast actuation event.

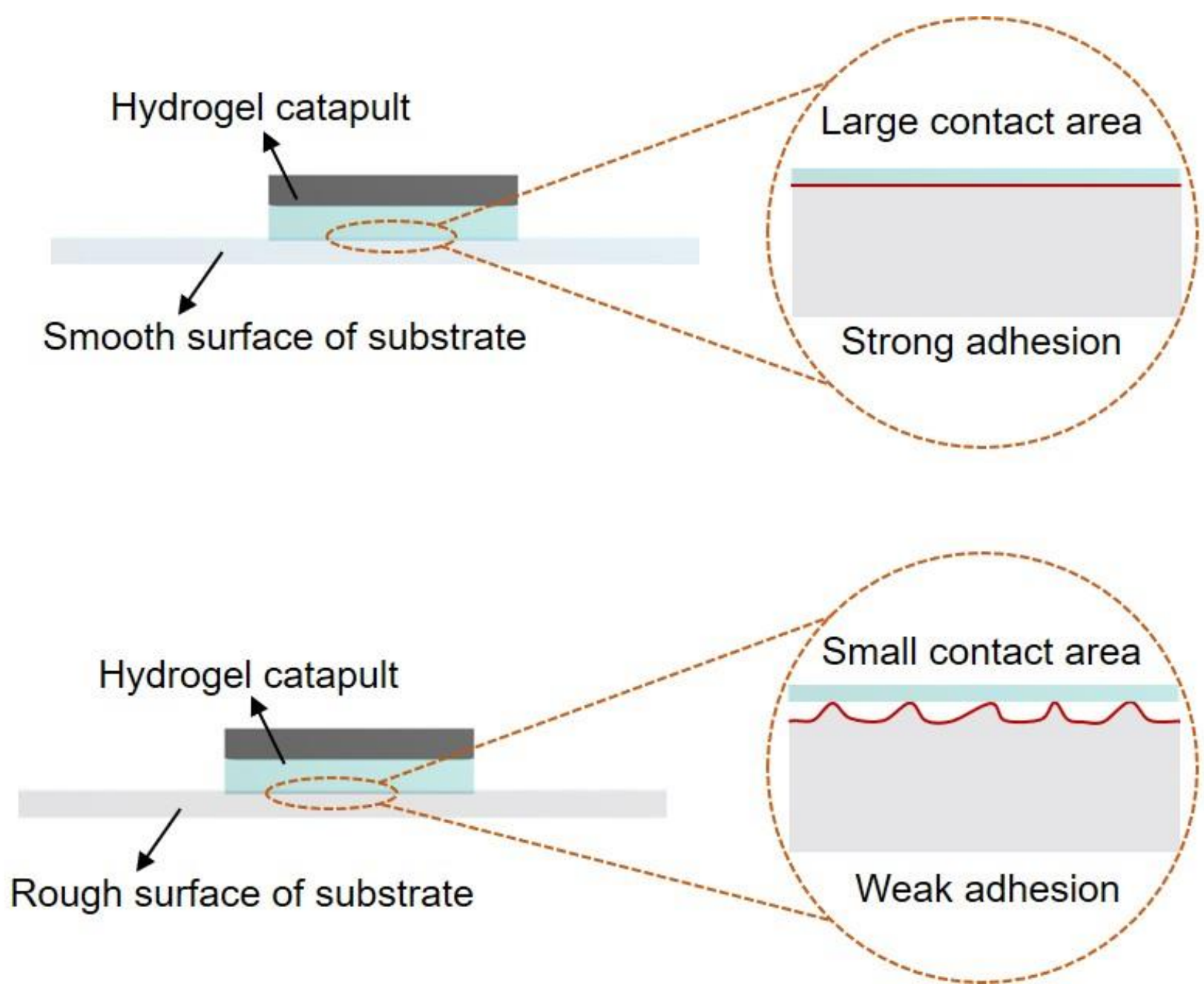

Fig. R2.10 (Supplementary Fig. 32). Schematic illustration of the adhesion force difference of the hydrogel catapult placed on the substrates with smooth surface or rough surface.

3. Payload influence on ejection performance

The weight of the projectile placed above the deformation zone also affects the internal pressure distribution during hydrogel deformation, thereby altering the interaction period and momentum transfer. As shown in Supplementary Fig. 33 (Fig. R2.11), the ejection height first increases and then decreases with increasing of the projectile's mass. Which arises from the combined effect of two competing factors. On one hand, increased projectile mass enhances momentum transfer from the hydrogel catapult (as discussed below). On the other hand, heavier projectiles require more kinetic energy to overcome gravitational potential energy during ascent, which reduces the achievable ejection height.

Furthermore, as shown in Fig. 2d that we have provided in the original manuscript, the kinetic energy transferred to the projectile initially rises with increasing mass and then reaches a plateau. This trend can be attributed to the dynamic coupling between the projectile and the deforming hydrogel. The weight of heavier projectiles disperses the impact pressure that would otherwise concentrate at the weakest point of the deformation zone, thereby delaying hydrogel network fracture and increasing the overall deformation. The extended interaction period results in greater cumulative momentum transfer to the projectile. We have also performed a theoretical analysis using a mathematical model (details can be found in Supplementary Note 5 in the Supplementary Information), and the results show strong agreement with our experimental findings.

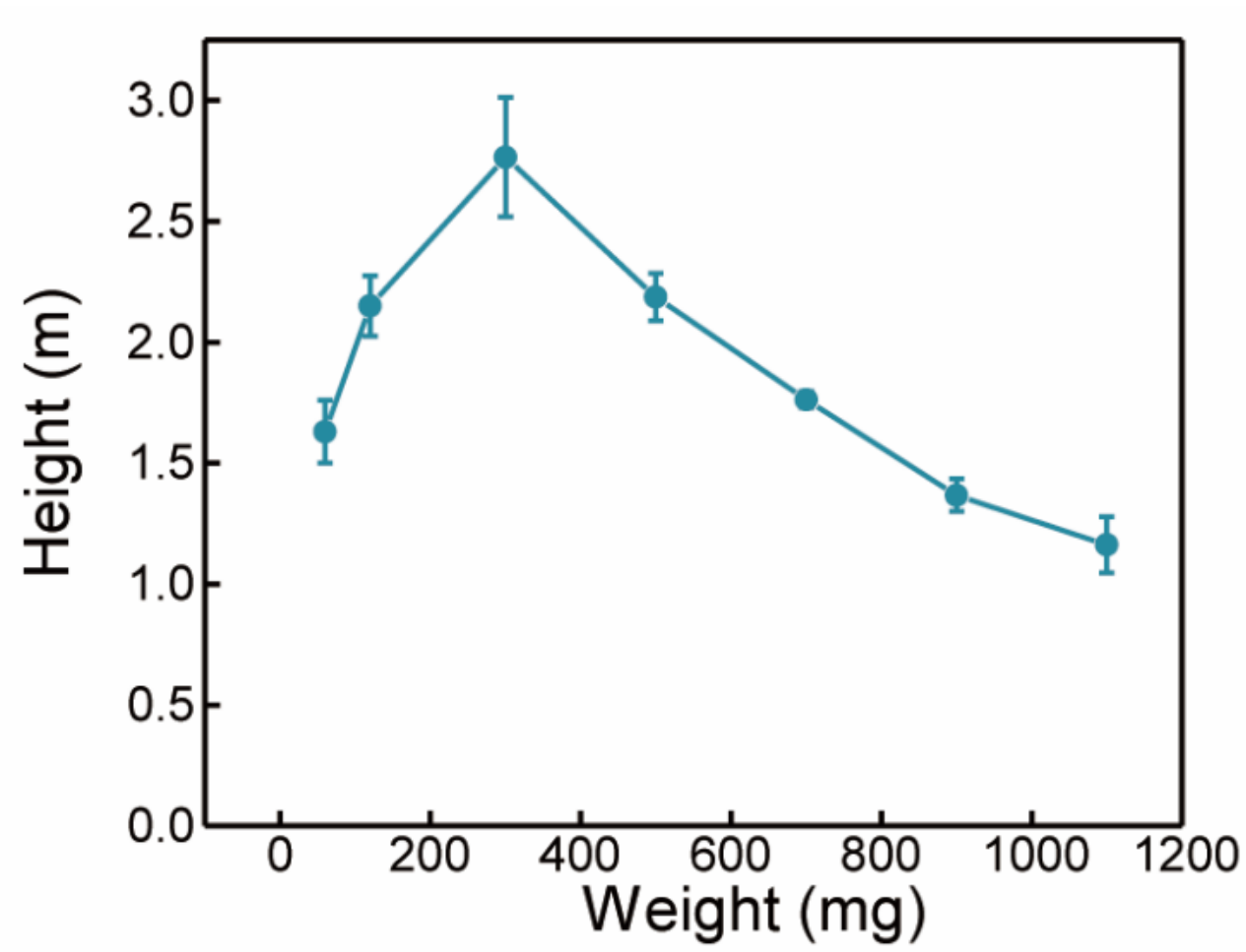

Fig. R2.11 (Supplementary Fig. 33). Ejection height of the projectiles with different weight. Note that all the projectiles are fabricated in cylindrical (diameter is 10 mm, height is 10 mm) with different density to reduce measurement errors. Data points are shown as mean ± s.d. (n = 4).

In summary, based on the comprehensive parametric data presented above, we can assess the robustness of the system for practical applications. The system performs optimally on rigid substrates with smooth surfaces, while maintaining functional ejection capability on compliant substrates and rougher surfaces while exhibited reduced performance. The optimal ejection performance arises from the dynamic coupling among three interacting factors, the substrate stiffness, interfacial adhesion, and projectile's weight. This tripartite coupling relationship provides quantitative design guidelines for tailoring the ejection system to specific application requirements.

We have revised the manuscript on page 13, line 257 as follows:

"Potential reason is that the interfacial adhesion reduces the kinetic energy loss caused by hydrogel displacement and reduces the rebound airflow dispersion, thereby improving initial kinetic energy generation and momentum transfer. Furthermore, the surface roughness alters the interfacial adhesion by changing the contact area between the hydrogel catapult and the substrate. A rougher surface reduces the contact area, thereby decreasing adhesion and altering the ejection performance (Supplementary Fig. 32)."

Reviewer #3:

Wang et al. designed a hydrogel catapult inspired by the ejection of Sphaerobolus spores. While the initial idea and claims are novel, the final device design is limited, and the proposed biomedical application does not align with the core concept. A significant issue with the hydrogel catapult is that it is single-use; after the initial explosion and fracture of the hydrogel network, the catapult must be replaced. This contradicts the traditional understanding of a "catapult," which implies a reusable mechanism. If the authors intend to demonstrate repeatable ejections with the same hydrogel catapult, they must quantify the number of consecutive successful ejections the device can perform to show its stability and lifetime.

We sincerely thank the reviewer for the detailed evaluation and for the positive assessment that the initial idea and claims are novel. We fully understand the reviewer's concerns about the single-use nature of the current device and the alignment of its application. In direct response to this feedback, we have now incorporated a comprehensive discussion into the Discussion section of the revised manuscript. Below, we respectfully provide clarifications that we believe resolve the issues raised.

(1) For the question of "single-use nature and the definition of catapult". The reviewer suggests that a "catapult" implies a reusable mechanism. However, we respectfully note that in the biological and biomechanical context, the vast majority of natural catapult systems are inherently single-use, irreversible mechanisms. For example, the biological prototype (i.e., *Sphaerobolus* sporangium ejection based on the irreversible membrane eversion) of this work[15], the *Impatiens* explosive seed dispersal[16], the Fern sporangium catapult[17] and the explosive seed dispersal by bending or coiling of pericarps[18]. Indeed, it is precisely the irreversible nature of these mechanisms that enables their extraordinary performance, the complete and one-time release of all stored energy produces power densities and velocities that far exceed what reusable (reversible) mechanisms can achieve. This is a well-established principle in biomechanics, i.e., the most powerful natural catapults universally sacrifice reusability for maximum single-event performance[19-21]. We have provided the corresponding literatures above for your convenience.

The term “catapult” in both the biomechanics and engineering literature describes the function of launching a projectile at high speed through rapid energy release, without implying reusability. Our usage is consistent with this established convention.

In addition, it is instructive to consider why single-use operation is not merely a limitation but is in fact physically necessary for achieving the performance levels reported in this work. The exceptional ejection performance (16.6 m/s take-off velocity, >8.74×10^4 W/kg power density) relies on the complete, ultrafast release of phase-transition energy within ~1.6 ms. This process involves the phase transition induced large deformation for energy storage and fracture induced ultrafast energy release. These processes are thermodynamically irreversible by nature. Any attempt to make the system reusable would require limiting the energy release (e.g., reducing light intensity, shortening irradiation time), which would proportionally reduce the deformation magnitude, momentum transfer, and ejection performance.

Furthermore, for the target application, rather than viewing the single-use nature as a limitation, we note that it is in fact a design advantage for the demonstrated applications. (i) For the current biopsy application, all clinical biopsy needles, cartridges, and disposable biopsy devices are single-use by regulatory requirement and clinical practice (e.g., FDA guidelines mandate single-use for percutaneous biopsy needles to prevent cross-contamination and ensure consistent performance). The single-use hydrogel catapult is fully consistent with, and well-suited to this clinical paradigm. (ii) The hydrogel catapult is fabricated from inexpensive, widely available materials (acrylic acid, acrylamide, graphene) using a simple UV-curing process, making single-use economically viable. The material cost per catapult is negligible compared to existing disposable medical devices. (iii) Single-use operation guarantees that each ejection event delivers maximum, reproducible performance without degradation from prior use cycles, that is a critical requirement for applications demanding reliable force output (e.g., tissue penetration).

While the individual hydrogel catapult is single-use, we note that repeated ejection functionality at the system level can be achieved through straightforward engineering strategies without modifying the fundamental actuation mechanism. For example, we can

fabricate the multiple hydrogel catapults that be arranged in an array, with each unit independently activated in sequence. This approach, analogous to a revolver cylinder, enables multiple ejection events from a single integrated device while each individual catapult operates in its optimal single-use mode.

(2) For the reviewer's comment related to the alignment of the biomedical application with the core concept. We respectfully disagree with the assessment that the biopsy application does not align with the core concept. The alignment is direct and specific. The core concept of this work is dynamic coupling for high-power ejection and force output in miniature soft systems. The biopsy application requires precisely this capability, i.e., high instantaneous force output (~2.34 N) from a miniature soft device to drive a needle into deep tissue in a single operation. The biopsy procedure is not an arbitrary application, rather, it specifically requires the unique capability enabled by our dynamic coupling mechanism, i.e., Newton-level force output in a millimeter-scale soft device.

We have conducted a comprehensive comparative analysis as shown in Supplementary Table 3 (Table R3.1) demonstrating that a critical unmet need exists in clinical gastroenterology, there is no existing tool simultaneously provides sufficient force for deep tissue penetration and a form factor compatible with intraluminal GI tract delivery. Spring-loaded biopsy guns provide adequate force but are rigid and percutaneous-only. The endoscopic tools (FNA/FNB) are intraluminally accessible but force-limited. Our system bridges this gap through the "local force generation" paradigm enabled by the dynamic coupling mechanism, which directly demonstrating the practical impact of the core scientific contribution.

Furthermore, as discussed above, the single-use nature of the catapult, which the reviewer identifies as a concern, is in fact standard clinical practice for biopsy devices, which making the application scenario and the device characteristics well-aligned rather than contradictory.

Table R3.1 (Supplementary Table 3). Comparison of the hydrogel catapult biopsy system with existing biopsy tools.

Aspect	Manual FNA/FNB	Spring-loaded biopsy devices	Catheter-based systems	This work
Force output	~ 1-5 N[9] (operator dependent)	High (spring dependent)	Typically < 0.5 N;	2.34 N
Force generation mode	Remote (transmitted through endoscope)	Remote (spring pre-loaded externally)	Remote (transmitted through endoscope)	Local (generated at tissue interface)
Needle gauge	19-25 G[3,4]	14-18 G[5] (mainly for percutaneous biopsy)	19-25 G[10]	Customizable; The local force output allows the needle thicker than 14G
Single-pass adequacy	Often insufficient; typically 3-7 passes required	High (core tissue obtained)	Often insufficient	High (thicker needle and sufficient force)
GI tract intraluminal access	Yes	Not applicable	Yes	Yes
Depth control	Operator tactile judgment	Yes	Varies	Mechanical depth stop
Actuation speed	Manual (~s)	Fast (~ms); (mainly for percutaneous biopsy)	Typically slow (~s)	Fast (ms)

(3) We wish to further reiterate that this work constitutes a fundamental study rather than an application-oriented effort toward medical device development. Our central focus lies in the dynamic coupling mechanism, with particular emphasis on elucidating the principles underlying high-performance output that arise from the mutual coupling among the deformation of the hydrogel catapult, the substrate, and the projectile. Our mechanism is universally applicable, the phase-transition based hydrogel catapult serves merely as a representative example. Any actuator capable of rapid deformation can leverage the dynamic coupling framework we proposed to achieve high-performance ejection. Our findings disentangle the role of dynamic coupling from structural complexity or material design. The performance surpasses most of the reported catapults (Fig. 1e and

Supplementary Table 1 and Table 2), demonstrating the powerful actuation capability afforded by the mechanism we propose.

We have added a discussion in the manuscript on page 27, line 464 as follows:

"It is worth noting that similar with the most powerful natural ejection systems, the superior performance of the hydrogel catapult comes at the cost of limited reusability. The single-use characteristic allows the device to release all stored energy in one shot, generating power densities and velocities far exceeding those achievable with reversible mechanisms. For current biopsy applications, the single-use nature complies with FDA requirements for instrumentation in biopsy procedures, e.g., biopsy needles and syringes are intended for single use. Furthermore, through grid or array designs, programmable sequential ejection can be achieved, which help to improve spatial selectivity and satisfy the requirements for scenarios demanding repeated actuation of the catapult."

Additionally, there is a lack of detailed material characterization beyond basic stress-strain tests. It is unclear what chemical changes or byproducts occur within the hydrogel network during actuation and phase transition.

We thank the reviewer for raising this important point regarding material characterization. We acknowledge that our original manuscript focused primarily on the mechanical and dynamic aspects of the catapult system, and we agree that a more detailed understanding of the chemical processes during actuation is valuable. Following the reviewer's suggestion, we have carried the material characterization and did comparation for the hydrogel catapult before and after the actuation. The results have added to the Supplementary Information, details are shown as follows.

We wish to clarify the fundamental nature of the actuation process. The actuation mechanism of our hydrogel catapult is a physical phase transition rather than a chemical reaction, i.e., the vaporization of water (from liquid to gas) confined within the hydrogel network. Specifically, the energy input (NIR light) is absorbed by graphene and converted into heat, the generated heat raises the local temperature inside the hydrogel above 100 °C,

causing water within the hydrogel network to undergo a liquid-to-vapor phase transition, which induced the steam expansion and ultrafast deformation of the hydrogel network. The weight loss is about 16.5 mg before and after the actuation (Supplementary Fig. 17a (as shown in Fig. R3.1a). In addition, to closely observe this process, we placed the hydrogel catapult at the bottom of the oil and recorded the ejection process of water vapor using high-speed imaging, as shown in Supplementary Fig. 17b (Fig. R3.1b). Owing to the immiscibility of water and oil, the vaporized water ejected after the hydrogel catapult fractured could be clearly visualized. The only "byproduct" of this process is water vapor, which is chemically identical to the original water within the hydrogel and is inherently non-toxic and non-hazardous. No chemical bonds are formed or broken in the driving medium (water) during this process. Therefore, the critical question is whether the polymer network (P(AA-co-AM)) and the photothermal agent (graphene) undergo any unintended chemical degradation under the conditions experienced during actuation (~120 °C peak temperature, ~1.6 ms duration). We address this through a combination of direct experimental characterization of our samples and established literature data on the thermal stability of the constituent materials.

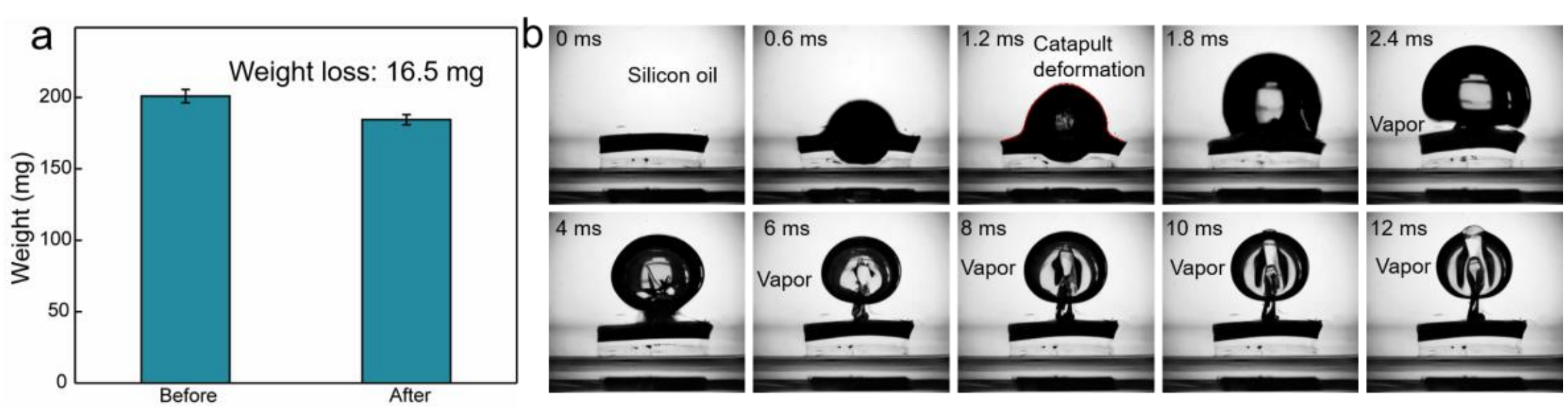

Fig. R3.1 (Supplementary Fig. 17). (a) The weight of the hydrogel catapult before and after the actuation. (b) High speed camera image illustration the vapor jetting process of the hydrogel catapult in the silicon oil.

The thermal stability of both constituent materials has been well characterized in the literature, the polymer backbone degradation of P(AA-co-AM) occurs above ~ 250 - 500 °C[22,23]. The graphene is thermally stable up to > 500 °C under ambient conditions[24]. The extremely short duration of the actuation event (~1.6 ms) further ensures that even if local temperature transiently reaches ~120 °C, the thermal exposure is insufficient to initiate any degradation process in either component. Polymer thermal decomposition is a

kinetically controlled process that requires sustained exposure at elevated temperatures (typically seconds to minutes), not the millisecond-scale transient heating experienced in our system.

In addition, to directly verify that the chemical structure of the hydrogel network remains intact after actuation in our specific material system, we performed Fourier-transform infrared (FTIR) spectroscopy on the hydrogel before and after the phase transition process. As shown in the Supplementary Fig. 18a (Fig. R3.2a), there is no new peaks appear in the post-actuation spectrum, confirming the absence of new chemical species or degradation products. And no existing peaks disappear, confirming that the functional groups of the polymer network remain intact. The relative peak intensities and positions are essentially unchanged, demonstrating that the chemical structure of the hydrogel is preserved through the actuation event. These results provide direct experimental confirmation that no chemical changes or degradation occur within the hydrogel network during the actuation process, consistent with the thermal stability analysis discussed above.

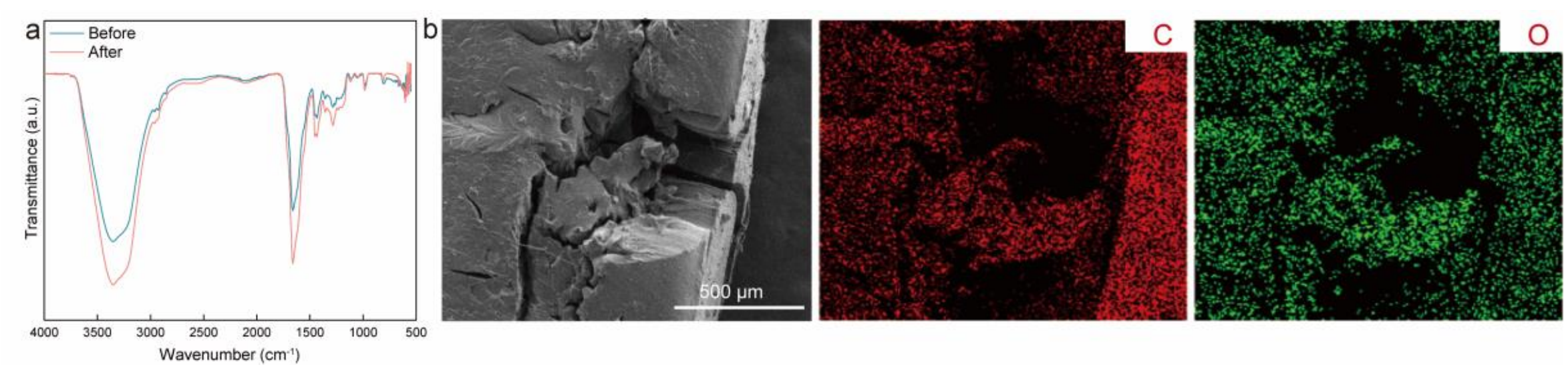

Fig. R3.2 (Supplementary Fig. 18). (a) The FTIR spectrum of the hydrogel catapult before and after the actuation. (b) The SEM and mapping images of the phase-transition region.

Furthermore, elemental analysis of the fractured region was conducted under SEM, as shown in Supplementary Fig. 18b (Fig. R3.2b). SEM observation clearly reveals the fractured morphology of the hydrogel matrix, while elemental mapping detects only the presence of carbon and oxygen. The absence of any other elements confirms that no byproducts are generated during the process. These results further substantiates that the actuation process is a purely physical (thermo-mechanical) event, and that the only irreversible change is the localized mechanical fracture caused by accumulated steam pressure after the projectile has detached.

In conclusion, the actuation process involves exclusively a physical phase transition of water, producing only water vapor as a byproduct. The polymer network and graphene remain chemically and structurally intact throughout the actuation event. The eventual fracture of the hydrogel network occurs after the ejection is complete, results from mechanical over-pressure rather from chemical degradation. These findings confirm the safety and chemical inertness of the actuation process, which is particularly relevant for the biomedical application demonstrated in this work.

We have added the above Figures into Supplementary Information and added a discussion in the manuscript on page 7, line 137 as follows:

"Continued irradiation of NIR light further induces the phase transition of the water (i.e., from liquid to vapor) inside the hydrogel network rapidly (Fig. 1b and Supplementary Fig. 17). This phase-transition process be proved to achieve high-speed self-actuation of hydrogel device that founded by Li *et al*[43], which is a physical process rather than a chemical reaction, no chemical bonds are formed or broken during this process (Supplementary Fig. 18)."

Another critical issue is the proposed biomedical application of endoscopic sample collection. This application is highly impractical in realistic clinical scenarios, given the significant force applied, the required near-infrared light intensity, and the substantial temperature increase. The authors note that the temperature exceeds 120°C in the upper layer.

The authors appreciate the reviewer's comments regarding the clinical practicality of the biopsy application. We respectfully submit that several aspects of the reviewer's concerns may stem from a misunderstanding of the encapsulation design, and we provide detailed clarifications below.

(1) Temperature and NIR light issues

We wish to respectfully clarify a critical distinction that we believe was overlooked, the 120 ℃ temperature cited by the reviewer refers exclusively to the internal graphene-doped

hydrogel layer, not to the tissue-contacting surface of the device. The hydrogel catapult is fully encapsulated within a PDMS shell (Fig. 5a), which provides effective thermal isolation between the internal actuation components and the external tissue-contacting surface. As shown in Supplementary Fig. 12 (Fig. R3.3), we have measured the temperature change of the device's surface which directly contact with the tissue, the highest temperature during the actuation is 40.3 °C, which is below the widely accepted threshold for thermal tissue damage. According to the thermal damage literature, tissue injury generally requires either sustained exposure above 43 °C for > 60 minutes, or acute exposure above 50 °C for several seconds[25]. Therefore, for our system produced the temperature of 40.3 °C and last only in several milliseconds, which resulting in a negligible cumulative thermal dose that is orders of magnitude below any clinically relevant injury threshold. Note that the highest temperature reached after the needle ejection due to the heat transfer process (Supplementary Fig. 12a, as shown in Fig. R3.3a).

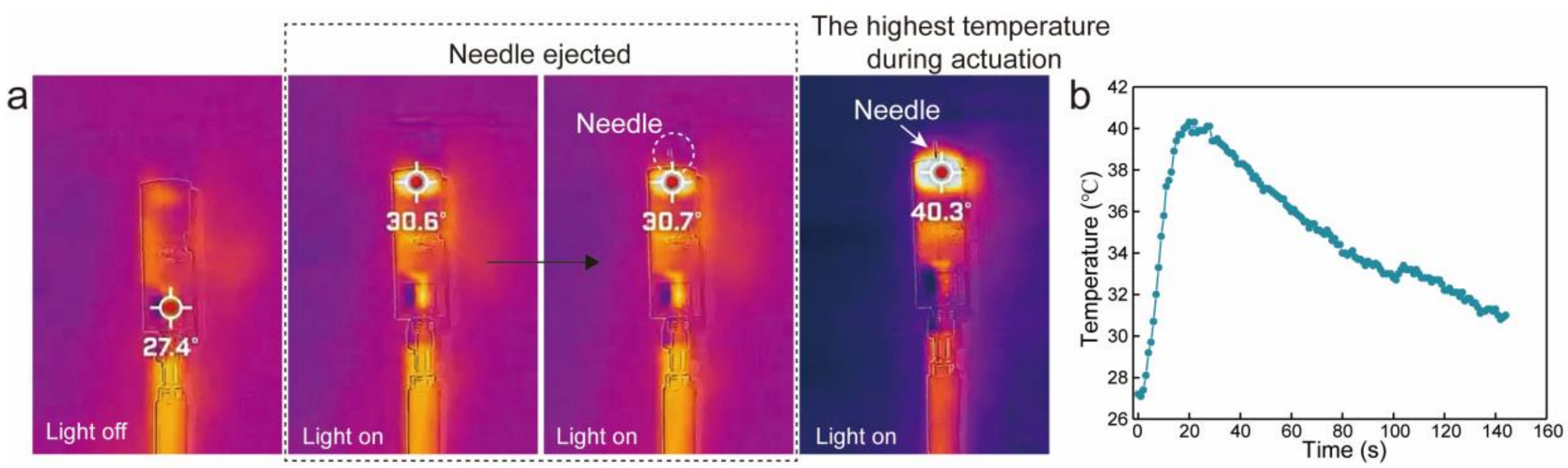

Fig. R3.3 (Supplementary Fig. 12). (a) A series of infrared thermal images and (b) temperature change profile showing the surface temperature of the device during the biopsy procedure.

In addition to the thermal insulation, the PDMS encapsulation serves multiple protective functions simultaneously, such as the NIR light containment (preventing leakage to tissue), mechanical protection (needle retraction after sampling), and environmental isolation (preventing liquid ingress in wet environments). For the reviewer's concern about NIR light intensity, we note that the NIR light will not reach the tissue during the actuation process. The light is confined entirely within the sealed encapsulation in our system. Furthermore, we can also modify the materials used (e.g., PTFE) in the preparation to further ensure the opaque properties.

(2) The applied force on the tissue

The reviewer expresses concern about "the significant force applied." We respectfully note that the force output of our system (~ 2.34 N, Supplementary Fig. 22) falls within the normal range for clinical tissue needle insertion, not above it.

Published studies on needle insertion biomechanics have established that forces of 1-5 N are typically required for needle penetration into soft tissues[9]. Our force output of ~2.34 N is within this range and is comparable to the force routinely applied by clinicians during manual fine-needle aspiration procedures. The key difference is not the magnitude of force, but the mode of delivery. Our system generates force locally at the tissue interface rather than transmitting it remotely through a curved endoscope, which is advantageous in terms of consistency and efficiency.

Furthermore, the penetration depth is mechanically constrained through the customizable stepped structure within the PDMS shell (Fig. 5a). This hard physical stop prevents over-penetration regardless of the applied force, providing a fail-safe mechanism analogous to the depth-limiting features of commercial spring-loaded biopsy guns. The combination of clinically appropriate force magnitude and mechanical depth limiting ensures that the system does not apply excessive force to tissue.

(3) Proof-of-concept positioning

We wish to reiterate that this work is fundamental research that focus on the dynamic coupling framework for the high-power output in the miniature soft robotics. The biopsy demonstration is presented as a **proof of concept** to validate the practical utility of the dynamic coupling mechanism for force delivery, not as a clinically ready device. We have stated this clearly in the revised manuscript. Nevertheless, as demonstrated above, the key safety parameters, such as tissue interface temperature, applied force and light exposure, are all within clinically acceptable ranges, suggesting that the fundamental approach is viable and warrants further translational development. Therefore, we respectfully maintain that the available data do not support the conclusion that the approach is "highly

impractical", rather, the evidence indicates that the safety-critical parameters are well-controlled within acceptable limits.

We have revised the manuscript on page 24, line 402 as follows:

"Biopsy is crucial for precision medicine, early disease detection, and patient stage monitoring. Currently, fine-needle aspiration biopsy (FNAB) and fine-needle capillary biopsy (FNCB) are widely employed for subsurface tissue sampling in endoscopic procedures[16,46]. However, due to the capacity of the fine needle, multiple punctures (usually 3-7 passes) are often required to obtain sufficient diagnostic material, which increases procedure time, patient discomfort, and the risk of complications. For regions that are difficult to access directly, such as gastric fundus sampling, the endoscope must be retroflexed, and the puncture force is transmitted from the operator's hand through the entire length of the endoscope to its tip, resulting in insufficient force or inaccurate puncture. Therefore, using a thicker needle to obtain sufficient samples and generating driving force at the tip are beneficial for reducing the need for repeated surgical maneuvers and improving precision. The kinetic energy transfer and force output enable the hydrogel catapult to be potentially suitable for the actuation of deep tissue biopsy robots. As a proof of concept, we developed a hydrogel-catapult-based biopsy system as shown in Fig. 5a."

Single-use, explosive-driven equipment is simply not viable for delicate tissue endoscopic applications. The authors should focus on non-medical applications of this technology instead. For these reasons, I believe this manuscript is currently unsuitable for publication in any scientific journal.

The authors appreciate the reviewer's comments. First of all, we respectfully clarify that the actuation mechanism of our hydrogel catapult is not an explosion. An explosion, by definition, involves a rapid, self-propagating chemical reaction (e.g., combustion, detonation) that generates gaseous products and a shock wave. Our system involves a fundamentally different process, i.e., the physical phase transition of water (from liquid to vapor) and released through mechanical fracture. There is no any byproduct except the

water vapor. This result also can be confirmed by our FTIR and SEM characterization above (Supplementary Fig. 18, as shown in Fig. R3.2).

Secondly, the reviewer states that single-use equipment is “simply not viable” for endoscopic applications. We respectfully note that this assertion is inconsistent with current clinical practice, where single-use (disposable) devices are not only viable but are in fact the predominant standard in interventional endoscopy. There are various medical devices is single use. For example, the EUS-FNA/FNB needles for tissue sampling, disposable biopsy forceps for mucosal biopsy, over-the-scope clips for perforation closure. The clinical trend is in fact moving toward greater adoption of single-use devices, driven by infection control concerns, manufacturing cost reductions, and the desire for consistent performance. Our single-use hydrogel catapult aligns with, rather than contradicts, this well-established clinical paradigm.

Moreover, the cost of our hydrogel catapult is negligible, it is fabricated from widely available, inexpensive materials (acrylic acid, acrylamide, graphene) using a simple UV-curing process, making it substantially less expensive than most existing single-use endoscopic accessories.

Third, the reviewer’s concern about applying this technology to delicate tissue is understandable. As mentioned in the answer of the above questions, we have already examined whether our system’s parameters fall within clinically established safety limits. (1) Our system delivers ~ 2.34 N, which falls within the 1-5 N range routinely applied during manual fine-needle aspiration biopsy in current clinical practice. This is not an excessive force and is suitable for effective tissue needle insertion. (2) We use customized stepped structure design to prevent any possibility of uncontrolled over-penetration. This fail-safe design is functionally equivalent to the depth-limiting mechanisms used in commercial biopsy guns. (3) The measured temperature at the tissue interface is 40.3 °C (Supplementary Fig. 12, as shown in Fig. R3.3), which is below the thermal damage threshold. (4) The faster needle insertion can reduce tissue deformation, dimpling, and drag compared to slow manual insertion, potentially reducing tissue trauma. The actuation time

of the hydrogel catapult (~ 1.6 ms) is substantially faster than manual insertion (~ seconds), which may be advantageous for minimizing tissue disturbance in delicate regions.

We also have demonstrated successful deep tissue sampling in tissue (Fig. 5e-f) and endoscopically guided biopsy within a porcine stomach (Fig. 5g-h, Supplementary Movie 7). These results provide direct experimental evidence that the system can operate effectively and safely on real tissue.

We appreciate the reviewer's suggestion to consider non-medical applications, and indeed our manuscript already demonstrates diverse non-medical capabilities in Fig. 1, Fig. 3 and Fig. 4. We have demonstrated a wide variety of ejection motion and control, and we believe our demonstrations will enable readers to understand and use our framework to apply it in the actuation of miniature soft robots. We believe that the biopsy application specifically demonstrates the core contribution of this work. The dynamic coupling mechanism enables Newton-level force output from a miniature soft device, which a capability whose most impactful and demanding application is precisely in the biomedical domain, where high force must be delivered from small, soft, intraluminal devices. Removing the biopsy demonstration would omit the most compelling validation of the practical significance of our work. In addition, there is a critical gap exists in current clinical gastroenterology, i.e., no existing tool simultaneously provides sufficient force for single-pass deep tissue penetration and a form factor compatible with intraluminal GI tract delivery. Our system is specifically designed to address this gap. And for the consideration of rigor, we have clearly positioned the biopsy demonstration as a proof of concept throughout the revised manuscript. We do not claim clinical readiness, but rather demonstrate the feasibility and motivate future translational development.

We respectfully note that the reviewer's conclusion, which describes the manuscript as "unsuitable for publication in any scientific journal," stems from a misunderstanding of our research field and its core contributions. We have given the point-by-point responses above. We believe the manuscript presents a significant advance in understanding dynamic coupling for high-power ejection in miniature soft systems, supported by comprehensive

characterization, theoretical analysis, and multiple application demonstrations including a clinically motivated proof of concept.

1. While the authors claim this is a "hydrogel catapult," the system is essentially a composite of a PDMS shell, graphene, and P(AA-co-AM) layers. The authors should focus on designing a device to control explosion-induced ejection. Currently, there is a lack of structural device design to physically control explosion-induced ejection. The authors could improve controlled ejection by incorporating grids or additional metastructures, such as kirigami patterns or bistable mechanisms.

The authors appreciate the reviewer's comments. We would like to clarify an important distinction between the core catapult and the application-level system. The term "hydrogel catapult" refers specifically to the bilayer P(AA-co-AM) hydrogel structure, which constitutes the core actuator responsible for all ejection dynamics characterized in the manuscript. Graphene is incorporated as a dopant within the hydrogel polymer network, it is not a separate structural component but a functional additive dispersed at the molecular/nanoscale level. The PDMS shell, 3D-printed components, and needle are employed in the biopsy robot demonstration (Fig. 5) as application-level packaging, which function is only help to demonstrate the dynamic coupling framework and force/output of the hydrogel catapult.

In addition, all fundamental ejection dynamics, performance characterization, and dynamic coupling analysis (Figs. 1 - 4) were conducted using the bare bilayer hydrogel structure without any PDMS or additional components. The term "hydrogel catapult" is therefore an accurate and appropriate descriptor of the core actuator.

While the bilayer hydrogel appears structurally simple, it incorporates multiple deliberate design that collectively control the ejection process. (1) The bilayer thickness ratio functions to directs deformation preferentially upward toward projectile. In our system, the upper layer thinner than bottom layer, the bottom layer serves as a constraint to directs the deformation that enables the controllable ejection. (2) The phase-change zone position be

controlled by the graphene content and light spot size. The light-blocking effect of graphene causes a phase transition to occur within the hydrogel, accumulating a large amount of energy. The area of the light spot determines the intensity of the phase transition, and these parameters can alter the force output of the hydrogel catapult. (3) The adhesion force, substrate coupling that have detailed illustrated in the Fig. 2, ensures positional stability during actuation and force output of the hydrogel catapult. These structural controls collectively govern the deformation direction, ejection magnitude, directional selectivity, and power output.

We wish to emphasize that the deliberate simplicity of the hydrogel catapult design serves an important scientific purpose in the context of this study. The core contribution of this work is the dynamic coupling framework, the discovery that the tripartite interaction among deformation, substrate, and projectile governs ejection performance. Isolating this mechanism from structural complexity is essential for establishing the fundamental physics. The fact that a simple bilayer hydrogel outperforms sophisticated latch-spring-actuated mechanical catapults across multiple performance metrics (Fig. 1e, Supplementary Table 1 and Table 2) directly demonstrates that the coupling mechanism, not structural elaboration, is the primary driver of the exceptional performance.

We agree that introduce metastructures represents a promising direction for future development. The dynamic coupling framework established in this paper provides a physical foundation for the innovation of such structures. Integration with metastructures and bistable mechanisms can further enhance their controllability and output performance. In addition, through grid or array designs, programmable sequential ejection can be achieved, effectively overcoming the single-use limitation of individual units while maintaining the full performance of each actuation event and improving spatial selectivity. Each of these structural enhancements will leverage the dynamic coupling relationships quantified in this study (substrate stiffness effects, adhesion effects, projectile mass effects) as design guidelines, positioning our framework as a foundation for future ejection systems with complex structures. Following the reviewer's suggestion, we have added a discussion in the Discussion section regarding promising directions based on the dynamic coupling framework established in this work, on page 27, line 464 as follows,

"It is worth noting that similar with the most powerful natural ejection systems, the superior performance of the hydrogel catapult comes at the cost of limited reusability. The single-use characteristic allows the device to release all stored energy in one shot, generating power densities and velocities far exceeding those achievable with reversible mechanisms. For current biopsy applications, the single-use nature complies with FDA requirements for instrumentation in biopsy procedures, e.g., biopsy needles and syringes are intended for single use. Furthermore, through grid or array designs, programmable sequential ejection can be achieved, which help to improve spatial selectivity and satisfy the requirements for scenarios demanding repeated actuation of the catapult."

2. While sample sizes are provided for some experiments, the authors must ensure that exact sample size information (e.g., n values) is included consistently across all main figure legends and in the Results section for clarity.

We thank the reviewer for this important point regarding reporting rigor. We fully agree that consistent reporting of sample sizes across all figures is essential for scientific transparency and reproducibility. As the reviewer's suggestions, we retested Fig. 5c using four replicates and averaged the results. The revised Fig. 5 is shown as follows,

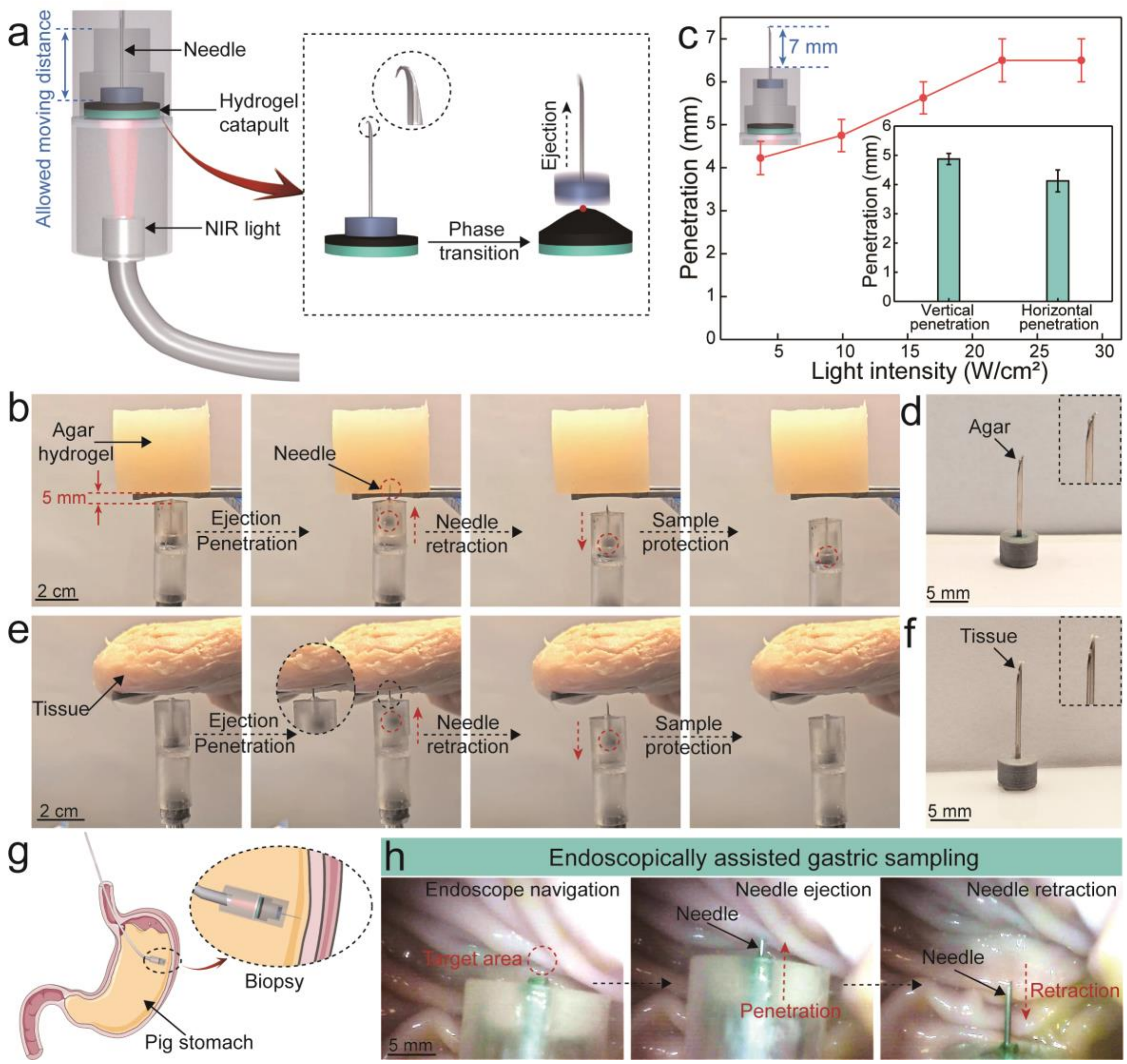

Fig. R3.4 (Fig. 5). Hydrogel catapult-actuated biopsy robot for gastric tissue sampling. (a) Schematic illustration of the structure and working principle of the developed biopsy robot. (b) A series of snapshots obtained from Supplementary Movie 6 illustrating the penetration process into tissue-like material (i.e., 5 wt% agarose gel). (c) The penetration depth in agarose gel with different light intensity. Inset: the comparison of the vertical and horizontal penetration depth. Data points are shown as mean ± s.d. ($n = 4$) (d) The image of the pulled-out needle from agarose showing the successful sampling. (e) A series of snapshots obtained from Supplementary Movie 6 and (f) image of needle after actuation indicating the successful sampling process. (g) Schematic illustration and (h) a series of snapshots obtained from Supplementary Movie 7 showing the biopsy process inside the pig's stomach. For representative images in Fig. 5 (photographs), technical repeated experiments (i.e. use one agarose gel, tissue and porcine stomachs) were independently carried at least 3 times with similar results, and representative data are shown.

In addition, we have checked for all main figures in the manuscript, for quantitative data plots (with error bars or statistical analysis), all figure legends explicitly state the sample size and statistical descriptors in a standardized format, i.e., "Data are presented as mean ± s.d. (n = 4)."

We would like to clarify that under the fixed test condition, the actuation process of the hydrogel catapult remains largely consistent. For representative images (e.g., high-speed camera snapshots, overlaid trajectories, photographs), we have added a statement in the figure legend for specifying the number of independent experiments from which the representative images were selected, details as follows,

"**Fig. 1. The ejection actuation mechanism of the hydrogel catapult.** (a) Schematic illustration of the ejection actuation mechanism. (b) The high-speed camera images obtained from Supplementary Movie 1 showing the ejection process of the hydrogel catapult. (c) An overlaid image obtained from Supplementary Movie 2 indicating the ejection height of the catapult actuated by NIR light. Inset: size comparison of hydrogel catapult and projectile used in Fig. 1c, the mass of the projectile is 203 mg that is similar with the BW of the catapult (200 mg). (d) An overlaid image obtained from Supplementary Movie 2 indicating the ejection of the projectile with the mass of 10 g (i.e., 50 BW of the hydrogel catapult). (e) The take-off velocity and projectile's mass comparison of the light driven hydrogel catapult with engineered catapult, plants/fungi and animals capable ejection motion in nature. Note that the data shown are limited in miniature organisms/engineered catapults with weight in *mg*-*g* level. For representative images in Fig. 1 (high-speed camera snapshots and overlaid trajectories), experiments were independently repeated at least 3 times with similar results, and representative data are shown."

"**Fig. 2. Factors influencing the ejection performance of hydrogel catapult.** (a) The deformation and response time comparison for hydrogel catapults on glass substrate, PDMS substrate and free standing (without substrate). Note the weight of the projectile is 380 mg. (b) Projectile velocity profiles during actuation on the substrate of glass, PDMS, and free-standing. (c) Schematic of interfacial adhesion force between hydrogel catapult and substrate. (d) Ejection height comparison with/without water lubrication (blocks interfacial adhesion) across different substrates. Data points are shown as mean ± s.d. ($n = 4$) (e) The kinetic energy acquisition of the projectiles with different weight and (f) response time of the hydrogel catapult. Note that all the projectiles are fabricated in cylindrical (diameter is 10 mm, height is 10 mm) with different density to reduce measurement errors. Data points are shown as mean ± s.d. ($n = 4$). For representative images in Fig. 2 (high-speed camera snapshots), experiments were independently repeated at least 3 times with similar results, and representative data are shown."

"**Fig. 3. Ejection performance of hydrogel catapult with diverse projectiles.** (a) Schematic and (b) snapshots obtained from Supplementary Movie 4 showing the powder ejection process. (c) Overlaid images

obtained from Supplementary Movie 4 showing the coin ejection process. (d) Schematic illustration and snapshots obtained from Supplementary Movie 4 showing hydrogel catapult as the actuator to drive the burette jump. (e) Schematic of a physical model representing sporangium-like ejection behavior. (f) High-speed images illustrate the ejection motion of the simulated projectiles (i.e., single spore, spore cluster and sporangium). (g) Location distribution for three representative ejection events. For representative images in Fig. 3 (photographs and overlaid trajectories), experiments were independently repeated at least 3 times with similar results, and representative data are shown."

"Fig. 4. Directional control of the ejection motion of hydrogel catapult. (a) Schematic illustration showing the directional control principle of the ejection motion. (b) The high-speed images in the activation moment and (c) launching trajectory obtained from Supplementary Movie 5 showing left and right ejection motion. Note that the light intensity and projectile is 15.68 W/cm^2 and 2280 mg, respectively. (d) Schematic illustration and (e) overlaid images obtained from Supplementary Movie 5 showing the multi-directional ejection capability. (f) Schematic and (g) trajectory analysis of magnet ejection to predefined target area on iron plate. For representative images in Fig. 4 (high-speed camera snapshots and overlaid trajectories), experiments were independently repeated at least 3 times with similar results, and representative data are shown."

"Fig. 5. Hydrogel catapult-actuated biopsy robot for gastric tissue sampling. (a) Schematic illustration of the structure and working principle of the developed biopsy robot. (b) A series of snapshots obtained from Supplementary Movie 6 illustrating the penetration process into tissue-like material (i.e., 5 wt% agarose gel). (c) The penetration depth in agarose gel with different light intensity. Inset: the comparison of the vertical and horizontal penetration depth. Data points are shown as mean ± s.d. ($n = 4$) (d) The image of the pulled-out needle from agarose showing the successful sampling. (e) A series of snapshots obtained from Supplementary Movie 6 and (f) image of needle after actuation indicating the successful sampling process. (g) Schematic illustration and (h) a series of snapshots obtained from Supplementary Movie 7 showing the biopsy process inside the pig's stomach. For representative images in Fig. 5 (photographs), technical repeated experiments (i.e. use one agarose gel, tissue and porcine stomachs) were independently carried at least 3 times with similar results, and representative data are shown."

3. The ex vivo experimental conditions are unclear. The authors cannot use organs in their studies without standardizing or registering them. The source of the tissue is unclear, and there is no detailed information about the properties of the porcine gastric tissue. For scientific transparency and ethical compliance, the source of the ex vivo tissue and the institutional registration number for animal byproduct use must be included. Ex vivo studies are also considered in life science study design. Please include details about the experimental design, such as biological replicates.

We sincerely thank the reviewer for raising this critical point regarding ethical compliance and experimental transparency. We fully agree that these details are essential for scientific rigor, as the reviewer's suggestion, we have added an "The fabrication of light driven biopsy robot and ex vivo biopsy demonstration" subsection in the Methods section to show the experiment details and porcine gastric tissue source.

We would like to clarify the porcine gastric tissue used in this study was obtained from a local commercial slaughterhouse in Hong Kong. All tissues were harvested post-mortem from healthy adult domestic pigs (Sus scrofa domesticus) slaughtered for routine commercial food production. No animals were sacrificed specifically for this study. As the tissues were obtained as byproducts of the commercial food supply chain, this study did not involve live animal experimentation. Based on the guidelines (https://aeec.cuhk.edu.hk/policy.html) of Animal Experimentation Ethics Committee (AEEC) in The Chinese University of Hong Kong (CUHK), the use of post-mortem animal tissues obtained from commercial food-processing facilities does not constitute animal experimentation under the guidelines of CUHK and therefore does not require AEEC approval. This exemption is consistent with the policies of major international regulatory bodies, which distinguish between the use of purpose-bred laboratory animals and the use of tissues obtained as byproducts of the food industry. All tissue handling procedures were conducted in compliance with institutional biosafety regulations.

In addition, we sincerely thanks for the reviewer point out that ex vivo studies are should considered in life science study design, we have revised the Reporting Summary and given the details of ex vivo biopsy demonstrations. For the ex vivo biopsy demonstration, sampling experiments were performed on tissue from a single porcine stomach with more than 3 times technical replicates (independent needle ejection and sampling events at different locations on the gastric wall). We wish to clarify that the purpose of these experiments is to demonstrate the functional capability of the device (i.e., whether the hydrogel catapult can generate sufficient force to drive a needle into gastric tissue and acquire a sample), rather than to characterize the biological variability of porcine gastric tissue properties. For this proof-of-concept validation, technical replicates demonstrating

consistent device performance across multiple independent actuation events are the primary metric of interest.

In the manuscript, we have added the "The fabrication of light driven biopsy robot and ex vivo biopsy demonstration" subsection in the Methods section on page 34, line 613, shown as follows,

"For the biopsy demonstration, the fresh porcine stomachs were obtained from a local commercial slaughterhouse in Hong Kong and transported to the laboratory in phosphate-buffered saline (PBS) at 4 °C. All experiments were performed within 3 hours of tissue procurement to ensure tissue freshness and mechanical integrity. The developed biopsy robot navigated by endoscope to target area in the porcine gastric, the biopsy procedures were performed on tissue from a single porcine stomach with more than 3 times technical replicates, i.e., independent needle ejection and sampling events at different locations on the gastric wall."

4. Time stamps are missing from the supplementary videos. Additionally, the playback speed changes mid-action in some videos, which is unacceptable for rigorous scientific documentation. Specifically, Supplementary Video 3 lacks speed information entirely.

We sincerely thank the reviewer for pointing it out. We have checked and comprehensively revised all the Supplementary Movies. We have added the time stamps inside the Supplementary movies which captured by high-speed camera (i.e., Supplementary Movie 1 and Supplementary Movie 3). In addition, speed information has been added to Supplementary Movie 3, and we have added playback speed information to all videos and ensured it remains constant throughout playback. Please find the details in the Revised Supplementary Movies.

Reference

1 Li, J. *et al.* Recent development of jumping motions based on soft actuators. *Adv. Funct. Mater.* **33**, 2300156 (2023).

2 Zhang, C. *et al.* Functional fluid-based soft robotic actuation. *Adv. Mater.* **37**, 2502669 (2025).

3 Wani, S., Muthusamy, V. R. & Komanduri, S. EUS-guided tissue acquisition: an evidence-based approach (with videos). *Gastrointest Endosc* **80**, 939-959 (2014).

4 Hébert-Magee, S. *et al.* The presence of a cytopathologist increases the diagnostic accuracy of endoscopic ultrasound-guided fine needle aspiration cytology for pancreatic adenocarcinoma: a meta-analysis. *Cytopathology* **24**, 159-171 (2013).

5 Parker, S. H. *et al.* Percutaneous large-core breast biopsy: a multi-institutional study. *Radiology* **193**, 359-364 (1994).

6 Huang, K. & Mashimo, H. Retroflexion in the stomach during endoscopy: importance of the examination and its utility. *Ann. N. Y. Acad. Sci.* **1554**, 343-352 (2025).

7 Hatta, W. *et al.* Treatment strategy after noncurative endoscopic resection for early gastric cancers in patients aged ≥ 85 years: a multicenter retrospective study in a highly aged area of Japan. *J. Gastroenterol.* **58**, 346-357 (2023).

8 Imagawa, A. *et al.* Endoscopic submucosal dissection for early gastric cancer: results and degrees of technical difficulty as well as success. *Endoscopy* **38**, 987-990 (2006).

9 Okamura, A. M., Simone, C. & O'Leary, M. D. Force modeling for needle insertion into soft tissue. *IEEE Trans. Biomed. Eng.* **51**, 1707-1716 (2004).

10 Son, D., Gilbert, H. & Sitti, M. Magnetically Actuated Soft Capsule Endoscope for Fine-needle Biopsy. *Soft Robot* **7**, 10-21 (2019).

11 Yarmolenko, P. S. *et al.* Thresholds for thermal damage to normal tissues: An update. *Int J Hyperther* **27**, 320-343 (2011).

12 Mahvash, M. & Dupont, P. E. Mechanics of Dynamic Needle Insertion into a Biological Material. *IEEE Trans. Biomed. Eng.* **57**, 934-943 (2010).

13 Abolhassani, N., Patel, R. & Moallem, M. Needle insertion into soft tissue: A survey. *Med Eng Phys* **29**, 413-431 (2007).

14 Hao, B. *et al.* Focused ultrasound enables selective actuation and Newton-level force output of untethered soft robots. *Nat. Commun.* **15**, 5197 (2024).

15 Money, N. P., Stolze, J. & Fischer, M. W. F. Mechanics of the artillery fungus. *Fungal Biol.* **128**, 2334-2340 (2023).

16 Hayashi, M., Feilich, K. L. & Ellerby, D. J. The mechanics of explosive seed dispersal in orange jewelweed (Impatiens capensis). *J. Exp. Bot.* **60**, 2045-2053 (2009).

17 Noblin, X. *et al.* The fern sporangium: a unique catapult. *Science* **335**, 1322-1322 (2012).

18 Huang, L. J. & Fu, W. L. A water drop-shaped slingshot in plants: geometry and mechanics in the explosive seed dispersal of Orixa japonica (Rutaceae). *Ann. Bot.* **127**, 765-774 (2021).

19 Ilton, M. *et al.* The principles of cascading power limits in small, fast biological and engineered systems. *Science* **360**, eaao1082 (2018).

20 Patek, S. N. Latch-mediated spring actuation (LaMSA): the power of integrated biomechanical systems. *J Exp Biol* **226**, jeb245262 (2023).

21 Money, N. P. Microballistics in fungi and plants. *Curr. Biol.* **34**, R977-R981 (2024).

22 Zhu, L. H., Wang, F. Y., Liu, Y., Tang, H. D. & Guan, C. D. Preparation and absorption properties of poly (acrylic acid-co-acrylamide)/graphite oxide superabsorbent composite.

Adv. Polym. Technol. **37**, 3680-3688 (2018).

23 Maurer, J. J. & Harvey, G. D. Thermal-degradation characteristics of poly(acrylamide-co-acrylic acid) and poly(acrylamide-co-dodium acrylate) copolymers. *Thermochim. Acta* **121**, 295-306 (1987).

24 Nan, H. Y. *et al.* The thermal stability of graphene in air investigated by Raman spectroscopy. *J. Raman Spectrosc.* **44**, 1018-1021 (2013).

25 Yarmolenko, P. S. *et al.* Thresholds for thermal damage to normal tissues: An update. *Int. J. Hyperthermia* **27**, 320-343 (2011).

Reviewer #1:

I have carefully read the author's responses and examined their responses to other reviewers. The manuscript has been appropriately revised following the comments received, and I think it's now ready for publication.

We sincerely thank the reviewer for the positive comments.

<u>Reviewer #2:</u>

The authors have addressed most of my comments in this round of revision. However, key concerns still remain.

We thank the reviewer for the careful review. Your constructive comments have helped us to further improve our manuscript. We have addressed every comment and made corresponding changes to the manuscript. Please check the following point-by-point response for details.

First, the novelty of this work remains a primary concern, as it appears to be significantly limited by previous studies. The fundamental mechanism is essentially the same as that reported in Li et al. 11(1), 3988, Nature Communications (2020), which was not cited in the original submission. Both studies employ NIR-light-driven photothermal heating to induce phase transitions of water within hydrogel networks. The authors also acknowledge that the two works share the same fundamental hydrogel actuation mechanism. Therefore, the current study appears to represent only an incremental advance, primarily by transferring the generated kinetic energy to external objects, while the demonstrations remain relatively straightforward and resemble discrete proof-of-concept showcases.

We sincerely thank the reviewer for carefully evaluating our revised manuscript and for highlighting the important prior work by Li *et al.* (Nature Communications, 2020, 11, 3988). We fully acknowledge that Li *et al.* established the foundational principle of NIR-light-driven phase-transition actuation in hydrogel systems, and we have now explicitly cited and discussed this work in the revised manuscript.

However, we would like to respectfully clarify that the present study addresses a distinct scientific question. Our central conclusion is that the coupling framework, rather than the NIR-triggered phase transition itself, is the key determinant of high-power soft ejection. Li *et al.* focused on achieving rapid self-actuation of hydrogel devices in air, thereby overcoming the slow response typically associated with diffusion-dependent hydrogel actuators. In contrast, our work investigates how the kinetic energy generated by a rapidly deforming soft hydrogel can be efficiently transferred to an external object, and what physical principles govern this transfer. This hydrogel-substrate-projectile dynamic

coupling problem is absent in self-contained jumping or bending actuators and has not been addressed in Li *et al*.

This distinction is critical because projectile ejection is not a simple extension of self-locomotion. In a jumping actuator, the energy-generating body and the moving body are the same system. However, in a catapulting system, energy must be transmitted across an interface to a separate object with different mass, geometry, and inertia, while the actuator itself remains largely stationary. Therefore, the output performance depends not only on the actuation trigger, but also on interfacial adhesion, substrate stiffness, projectile mass, and the resulting dynamic impedance matching.

Consistent with this distinction, the major performance improvements in our system do not arise from changing the phase-transition chemistry or the hydrogel material system. Instead, they arise from the dynamic coupling strategy. Compared with Li *et al.*, our system achieves substantially higher velocity, faster response, larger payload capacity, and higher force output. These performance gains support the conclusion that optimizing energy transfer through dynamic coupling, rather than modifying the underlying phase-transition mechanism, is the primary route to achieving high-power soft ejection.

We also respectfully clarify that the demonstrations in this work are not isolated proof-of-concept examples. The substrate stiffness study, interfacial adhesion study, projectile mass study, directional ejection experiments, and biopsy demonstration are each designed to test a specific component of the dynamic coupling framework. Together with the theoretical analyses in Supplementary Notes 4 and 5, these experiments establish a coherent set of design principles for soft catapult systems. The biopsy device further illustrates how this framework can generate localized Newton-level force output in a miniaturized, endoscopically deliverable format. We agree that this device is not yet a clinical product, but it provides a physically grounded validation of the proposed design principle.

Accordingly, while we fully recognize the foundational contribution of Li *et al.* in phase-transition-based hydrogel actuation, our work makes a distinct contribution by revealing and validating the physical principles governing kinetic energy transfer from a rapidly deforming soft actuator to an external projectile. We have revised the manuscript to explicitly cite Li *et al*. and to clarify the difference between self-actuation and projectile ejection.

We have added a discussion in the manuscript on page 7, line 133 as follows:

"This phase-transition process has been demonstrated to achieve high-speed self-actuation of hydrogel devices, as pioneered by Li *et al.*[43]. As a physical process rather than a chemical reaction, no chemical bonds are formed or broken during this transition (Supplementary Fig. 18). In addition, the jumping and ejection represent two fundamentally distinct modes of motion, ejection require more complex and difficult energy transfer process, we adopted this phase-change triggering method to explore potential coupling principles with interfaces and projectiles, thereby achieving the high power output of the catapult. Specifically, the rapid steam expansion drives the highly stretchable and tough hydrogel matrix to undergo fast, drastic deformation, generating substantial initial momentum that is subsequently transferred to the projectile to activate the ejection process. Crucially, this deformation is directionally guided toward the projectile by introducing an asymmetric thickness profile. The constraint layer at the bottom of the catapult is deliberately designed to be thicker than the upper layer near the phase-change area (i.e., the thinner upper surface provides less resistance to expansion), thereby ensuring targeted energy focus (Supplementary Fig. 19)."

Moreover, the inherently unidirectional actuation nature of the projectile mechanism makes the system less compelling compared with previous studies focusing on repeatable hydrogel robot locomotion/actuation. The issue of lacking bidirectional momentum transfer is largely overlooked, which significantly limits the practical applicability of the proposed mechanism. One notable example is the biopsy unit demonstration. A major concern is the retrieval of the biopsy unit after sampling. Although the authors demonstrated sampling in a retroflexed region, it remains unclear how the biopsy unit can be safely and reliably retrieved afterward, which could present substantial technical challenges in practical implementation.

The authors appreciate the reviewer's comments and would like to respectfully clarify the fundamental design principles underlying our work. Inspired by nature, our catapult system achieves an exceptionally high power density and impulsive force output through a dynamic coupling design and irreversible energy release. In fact, this ballistic design principle is well-precedented and widely accepted in both biological and engineering

systems. As discussed in the manuscript, the most powerful natural ejection systems, e.g., *Sphaerobolus* sporangium dispersal, explosive seed ejection in plants, and mantis shrimp strikes, are similarly unidirectional and non-repeatable at the individual event level, but we must acknowledge that these behaviors represent the pinnacle of biological power amplification. The trade-off between reusability and peak performance is a fundamental physical constraint, i.e., reversible actuators must store and release energy within the elastic limit of the material, which inherently caps the achievable power density. By contrast, irreversible actuation allows all stored energy to be released in a single event, enabling power densities (>8.74×10^4 W/kg) that far exceed those of any reported repeatable soft actuator system.

In addition, we would like to point out that repeatable locomotion systems are optimized for sustained mobility and energy efficiency over multiple cycles. Our catapult system is optimized for instantaneous high-power output and force delivery, this performance regime that is fundamentally inaccessible to reversible mechanisms. These represent complementary, rather than competing capabilities in the soft robotics landscape. We believe that the developed catapult and repeatable actuation devices are in equal levels that designed for different engineering purposes and satisfying the different application needs. Moreover, the materials we employed are inexpensive, and the fabrication method is straightforward, large-scale production of the catapult can be achieved through simple UV photopolymerization. Furthermore, the limitations associated with single use can be significantly reduced by adopting array or grid design strategies, which raised by the reviewer in the last round review. Through programmable sequential actuation of individually addressable catapult units arranged in an array, repeated and spatially selective ejection events can be achieved.

(2) We thank the reviewer for the question regarding whether the catapult can be actuated in both the ejection and retraction directions. We agree with the reviewer's comment that the lack of bidirectional actuation capability leads to this outcome. However, we would like to clarify that this is an inherent trade-off in our design. As explained above, the high-power output of the catapult is achieved at the expense of reversible actuation, yet this represents an equally important design philosophies similar with the repeatable actuation for the miniature soft robotics.

For the target application scenario, i.e., biopsy, complete engineering solutions already exist for retrieval of the biopsy needle. For example, spring-driven or manual push-pull via a tethered method can retract the needle into the shell after sampling. These designs can all be integrated into our endoscope-assisted biopsy system. Drawing on established clinical engineering designs, as shown in Supplementary Fig. 38 (Fig. R2.1), we integrated a tethered pulling mechanism into the base of the biopsy needle, we achieved retraction of the needle into a protective shell after the endoscopic-assisted sampling process, thereby preventing contamination and loss during retrieval. The related demonstration has been added to the Supplementary Movie 6.

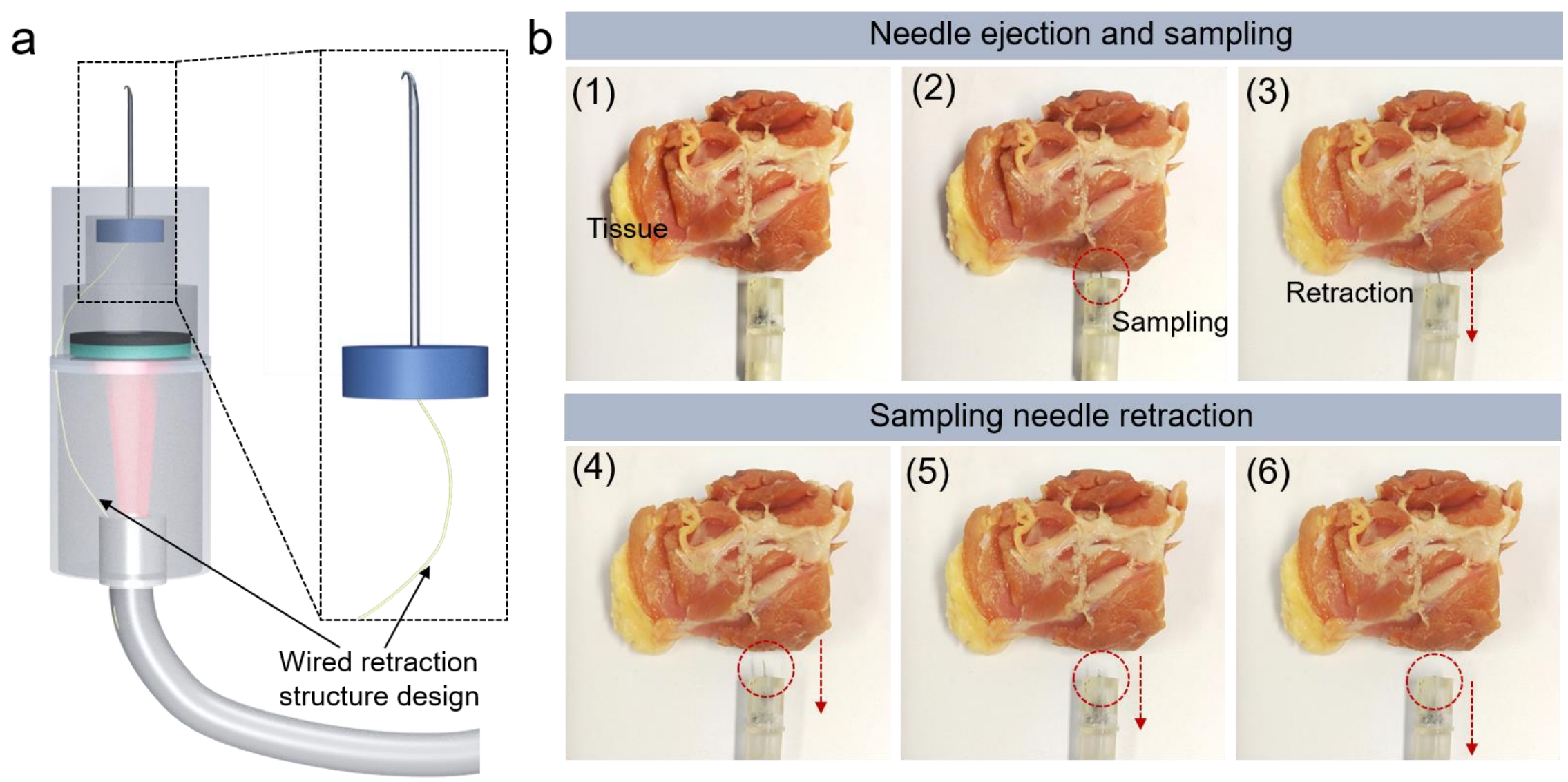

Fig. R2.1 (Supplementary Fig. 38). (a) The schematic showing the structure design for retraction of needle. (b) A serious of images obtained from Supplementary Movie 6 showing the sampling and retraction process of the developed biopsy device.

Furthermore, we would like to emphasize that our biopsy demonstration serves as a foundational proof-of-concept validation rather than an immediate pre-clinical product development. The significance of this demonstration lies in proving that our dynamic coupling principle can elicit an exceptionally robust impulsive output. This strategy proffers a promising solution to mitigate the limitations of current biopsy techniques, specifically by bypassing the need for repetitive needle passes and the restrictive usage of ultra-fine needles.

We have added the aforementioned figure to the Supplementary Information as Supplementary Fig. 38, and the video demonstrating the retraction process has been included as Supplementary Movie 6. Furthermore, we have revised the manuscript on page 26, line 439, as follows:

"Subsequently, the sampling needle retracts into the protective shell via a tethered traction mechanism (Supplementary Fig. 38 and Supplementary Movie 6), preventing sample contamination and eliminating in vivo safety risks (Fig. 5b). Concurrently, the integrated barbed design ensures secure and reliable sample retention (Fig. 5d)."

Lastly, additional literature support is needed for the claims related to the biopsy unit. The authors state that the ability to use a 14 G needle could reduce the number of biopsy passes; however, no supporting references are provided to substantiate the claim that "multiple punctures (typically 3–7 passes) are often required to obtain sufficient diagnostic material, which increases procedure time, patient discomfort, and the risk of complications.

The authors appreciate the reviewer's comments. We entirely agree with the reviewer's comment that the claim regarding the number of biopsy passes required should be supported by appropriate clinical literature references. As the reviewer's suggestions, we have added the specific supporting references to substantiate this statement in the revised manuscript.

Extensive literature has established that larger needle diameters yield greater sample volumes per single puncture[1-3]. Currently, 22 G and 25 G needles predominate in fine-needle biopsy (FNB) procedures. Consequently, performing multiple needle passes remains a standard clinical strategy to compensate for the volume limitations inherent to these smaller-gauge needles.

Multiple prospective studies and meta-analyses have systematically investigated the number of passes required to achieve adequate tissue sampling in FNB and FNA technologies (Note: Detailed reference information for the cited literature can be found at the end of this response). For example, Mitsuhashi *et al.* evaluated specimen adequacy, diagnostic criteria for various pancreatic neoplasms and gastrointestinal (GI) tract contamination during endoscopic ultrasound-guided fine needle aspiration (EUS-FNA)[4].

They explicitly stated that, “It has been recommended that three to six passes be performed when no pathologist is available for adequacy assessment at the time of the procedure.” In addition, de Moura *et al.* compared FNA and FNB techniques under combined rapid on-site evaluation (ROSE) assessment, demonstrating that FNB required fewer passes than FNA to obtain a sufficient sample size for ROSE assessment (3.85 ± 1.34 *vs* 4.53 ± 1.28, which represent the mean number of needle passes for these operations)[5]. Furthermore, Gan *et al.* evaluated the utilization of EUS-FNA and EUS-FNB in the diagnosis of pancreatic ductal adenocarcinoma (PDAC). Their data (as detailed in Table 2 of their study)[6] indicated that the average number of passes required to obtain a sufficient sample volume using ProCore (Cook Medical, Bloomington, Indiana) and Acquire (Boston Scientific, Marlborough, Massachusetts) needles (ranging from 22 G to 25 G) were 4.6 and 3.4 times, respectively. Moreover, Wani *et al.* evaluated impact of on-site cytopathology evaluation (OCE) during endoscopic EUS-FNA of pancreatic masses, which previously cited in our last round of response. They reported that the procedures with OCE necessitated a significantly lower number of passes compared to those without OCE (3.7 *vs* 7). Therefore, based on the aforementioned literature, performing clinical biopsies using FNA or FNB (typically utilizing 19 G-25 G needles) routinely requires 3-7 needle passes to ensure the acquisition of an adequate sample.

The clinical consequences of multiple passes are also well-documented. For example, Salah *et al.* discussed the influence of needle type and size on the diagnostic accuracy, adequacy of sample size and number of passes needed during EUS-FNA, they clearly stated[7] that “The ability to obtain a diagnosis with fewer passes has multiple advantages and may lead to shorter procedure duration and improved patient safety.” Therefore, optimizing needle size to secure sufficient tissue samples within a single penetration represents a critical strategy for shortening intervention times and optimizing patient safety.

We have added the above corresponded supporting references in the manuscript on page 25, line 409, as follows:

“However, due to the limited capacity of the fine needle, multiple punctures (usually 3-7 passes) are often required to obtain sufficient diagnostic material[47], which increases procedure time, patient discomfort, and the risk of complications[48]. For regions that are difficult to access directly, such as gastric fundus sampling, the endoscope must be

retroflexed[49], and the puncture force is transmitted from the operator's hand through the entire length of the endoscope to its tip, resulting in insufficient force or inaccurate puncture."

References

1 Liffman, R. & Courtman, N. Fine needle aspiration of abdominal organs: a review of current recommendations for achieving a diagnostic sample. *J Small Anim Pract* **58**, 599-609 (2017).

2 Haseler, L. J. *et al.* Syringe and needle size, syringe type, vacuum generation, and needle control in aspiration procedures. *Cardiovasc Inter Rad* **34**, 590-600 (2011).

3 Shang, R. M. *et al.* Comparing needle types and aspiration techniques in EUS-TA to optimize diagnostic efficacy and specimen quality in patients with pancreatic lesions. *Front Med-Lausanne* **11** (2024).

4 Mitsuhashi, T., Ghafari, S., Chang, C. Y. & Gu, M. Endoscopic ultrasound-guided fine needle aspiration of the pancreas: cytomorphological evaluation with emphasis on adequacy assessment, diagnostic criteria and contamination from the gastrointestinal tract. *Cytopathology* **17**, 34-41 (2006).

5 de Moura, D. T. H. *et al.* EUS-guided fine-needle biopsy sampling versus FNA in the diagnosis of subepithelial lesions: a large multicenter study. *Gastrointest Endosc* **92**, 108-119 (2020).

6 Gan, Q. *et al.* Adequacy evaluation and use of pancreatic adenocarcinoma specimens for next-generation sequencing acquired by endoscopic ultrasound-guided FNA and FNB. *Cancer Cytopathology* **130**, 275-283 (2022).

7 Salah, W. & Faigel, D. O. When to puncture, when not to puncture: Submucosal tumors. *Endoscopic Ultrasound* **3**, 98-108 (2014).

Reviewer #3:

I would like to express my gratitude to the authors. They provided thorough answers to all my inquiries regarding the article, ensuring its suitability for publication.

We sincerely thank the reviewer for the positive comments.
