# Supplementary material for: Dynamic coupling enables impulsive ejection in a hydrogel catapult
Source: Nat Commun. 2026 Sep 7;17:10568. doi: 10.1038/s41467-026-77391-6 (PMC13642336; doi:10.1038/s41467-026-77391-6)
Supplement: Supplementary file 2 [file 41467_2026_77391_MOESM1_ESM.pdf]

Supplementary Information

Dynamic coupling enables impulsive ejection in a hydrogel catapult

Xin Wang[1†], Neng Xia[1†], Chengfeng Pan[2*†], Bo Hao[1], Chaoyu Yang[1], Xinyu Wu[3], Tiantian Xu[3*], Qingsong Xu[4*], Li Zhang[1,5,6,7*]

[1]Department of Mechanical and Automation Engineering, The Chinese University of Hong Kong, Hong Kong, SAR 999077, P.R. China

[2]The State Key Laboratory of Fluid Power and Mechatronic Systems, College of Mechanical Engineering, Zhejiang University, Hangzhou, Zhejiang 310027, P.R. China

[3]Guangdong Provincial Key Laboratory of Robotics and Intelligent System, Shenzhen Institutes of Advanced Technology, Chinese Academy of Sciences, Shenzhen, P. R. China

[4]Department of Electromechanical Engineering, Faculty of Science and Technology, University of Macau, Macau, P.R. China

[5]CUHK T Stone Robotics Institute, The Chinese University of Hong Kong, Hong Kong, SAR 999077, P.R. China

[6]Chow Yuk Ho Technology Center for Innovative Medicine, The Chinese University of Hong Kong, Hong Kong, SAR 999077, P.R. China

[7]Department of Surgery, The Chinese University of Hong Kong, Hong Kong, SAR 999077 P.R. China

*Corresponding Author: cfpan@zju.edu.cn (C.P.); tt.xu@siat.ac.cn (T.X.); qsxu@um.edu.mo (Q.X.); lizhang@mae.cuhk.edu.hk (L.Z.)

†These authors contributed equally to this work.

1. Supplementary Tables

Supplementary Table 1: Comparison of the ejection performance of the hydrogel catapult in the current study with previously reported engineered catapult, representative plants/fungi and animals with ejection motion behavior in nature

Name	Take-off velocity (m/s)	Take-off acceleration (g)	Response time (ms)	Ejection distance (cm)	Projectiles mass (mg)	Peak power output (W/kg)	Ref.
This work	**16.6**	**1037.5**	**1.6**	**615**	**203**	**8.74×10^4**	**This work**
**Engineered catapult**							
LCE actuator	2.5	6.25	40*	20	35	-	*1*
LCE actuator	0.4*	0.36	110*	13	1	-	*2*
LCE actuator	0.0495	0.0275	180	21	30	-	*3*
4D-printed catapult	3	39	7.7	-	2*	-	*4*
Mantis shrimp throwing microrobot	10.6	176.66	6*	-	65	-	*5*
**Plants**							
Cornus canadensis	3.1	2446	0.5	2.5	0.024	7.3×10^4	*6*
Adiantum peruvianum	2.4	6320	-	5.7	-	1.48×10^5	*6*
Impatiens capensis	1.24	29.52	4.2	175	10.7	-	*7*
Impatiens glandulifera	6.196	206.53	3	0-1000	20.7	-	*8*
Cardamine parviflora	6.29	133.83	4.7	94	0.15	1.9×10^4	*9*
Erodium cicutarium	6	300	2*	0.51	5	-	*10*

**Fungi**							
Aleurodiscus gigasporus	0.53	883.33	0.06*	0.18	0.017	-	*11*
Auricularia auricula	1.23	12000	0.01	0.18	3.7×10^{-7}	2.8×10^{4}	*12*
Ascobolus immersus	14	183486	0.06	30	0.001	-	*13*
Itersonilia perplexans	0.67	25484	-	-	1.5×10^{-6}	1.64×10^{5}	*6*
**Animals**							
Chameleo melleri	6	38.1	20	-	4000	1584	*14*
Bolitoglossa dofleini	3.8	177	7	2	790	4109	*15*
Eurycea guttolineata	2.3	80.7	7	0.9	1290	778	*16*
Dyscophus guineti	1.2	14.7	16.6	-	810	316.8	*17*
Rana pipiens	0.6-3.2	45.8	17-92	0.9-3.8	900	885	*18*
Hydromantes imperialis	4	82.6	13	33	1040	2923	*15*
Furcifer lateralis	4.1	74	32.3	11	-	4080	*19*
Brookesia superciliaris	4.4	137.6	18.7	9	-	9080	*19*

*Data is obtained by high-speed images or estimation/calculation that provided in the original articles.

Supplementary Table 2: Comparison of the ejection performance of the hydrogel catapult in the current study with previously reported engineered jumpers.

Name	Take-off velocity (m/s)	Take-off acceleration (g)	Response time (ms)	Peak power output (W/kg)	Ref.
This work	**16.6**	**1037.5**	**1.6**	**8.74×10^4**	**This work**
Fracture driven hydrogel actuator	7.46	2540	0.3	9.3×10^4	20
LCE actuator	1.76	5.68	1	1548.8	*21*
LCE soft robot	1.6	3.2	50	25.6	*22*
PDMS/GO hybrid actuators	2	12.5	16	125	*23*
Light driven hydrogel actuator	1.6	24.73	6.47	197.8362	*24*
Dual-responsive all-carbon actuators	0.505	0.63	80	1.593906	*25*
Swelling hydrogel actuator	0.26	2.17	12	2.816667	*26*
conjugated ladder polymer based actuator	0.71	2.37	30	8.401667	*27*
PDMS actuator	3	150	2	2250	*28*
Magnetic driven gearbox	2.3	306.67	0.75	3526.667	*29*
Electric driven dielectric elastomer soft robot	0.39	3.98	10	7.605	*30*
Spring driven jumping robot	28	311.11	9	43555.56	*31*
SMA driven jumping robot	2.7	270	1	3645	*32*

Note: For the calculations of power output of engineered actuation systems with jumping motion, we assume that the catapult converts almost all the stored energy into the kinetic energy of the projectile to estimate the energy storage for a fair comparison. Therefore, the energy was estimated through $E_{kinetic\ energy} = 1/2\ mv^2_{take\text{-}off}$.

2. Supplementary Notes

Supplementary Note 1: The function of graphene and substitutability

The function of graphene in hydrogel catapult serves as energy transducer and optical barrier to control the phase transition occurred inside the hydrogel matrix. First of all, the graphene serves as a photothermal agent to produce the heat due to the photothermal effect, thus inducing the ultrafast liquid-to-vapor phase transition of water confined within the hydrogel network. Secondly, the black color of graphene plays an equally important role by attenuating the NIR light as it penetrates into the hydrogel. Due to the strong optical absorption of graphene, the incident NIR light cannot fully penetrate through the graphene-doped upper layer (Supplementary Fig. 1). Instead, the light energy is progressively absorbed within a limited penetration depth from the irradiated surface. This optical confinement effect ensures that the phase transition occurs internally within the hydrogel at a controlled depth beneath the surface rather than throughout the entire volume, which is essential for generating the localized internal pressure buildup that drives the directional deformation.

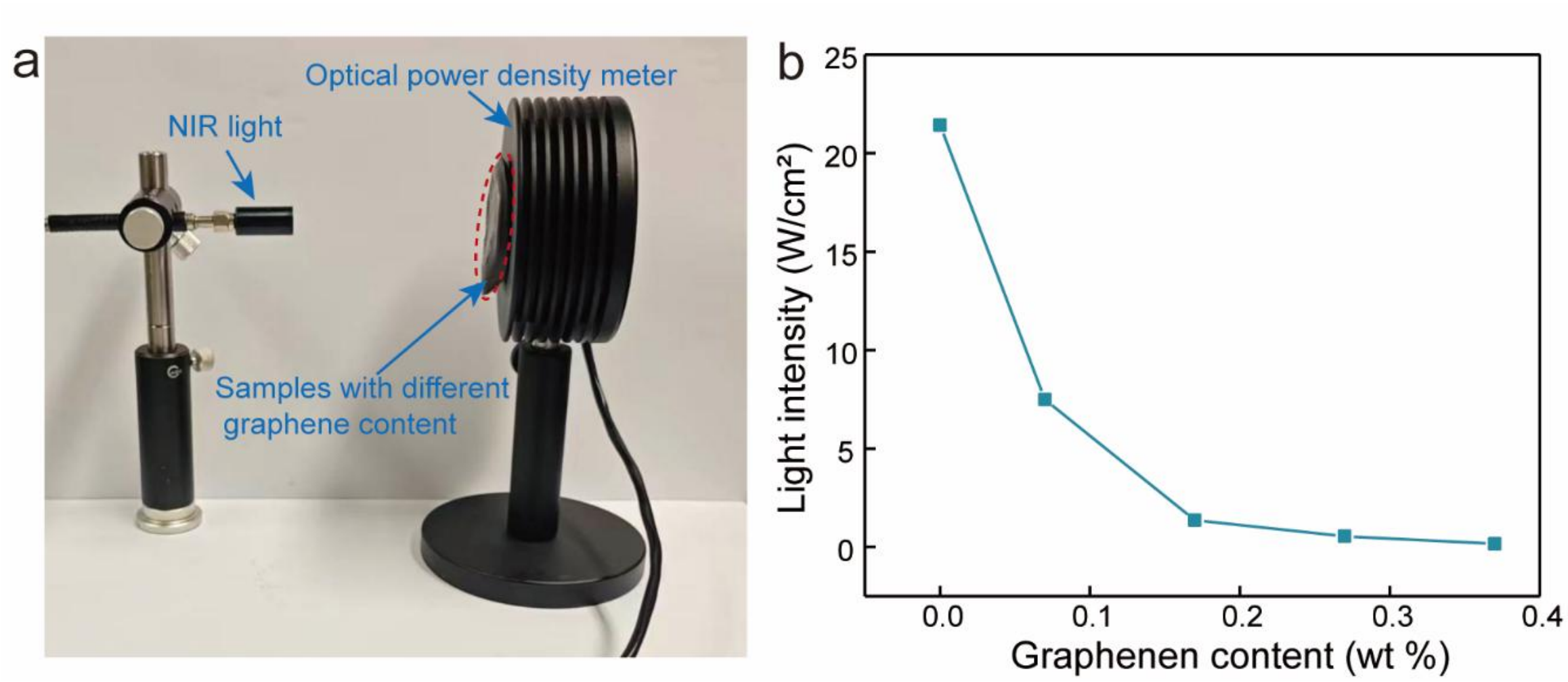

Supplementary Fig. 1. (a) Photograph of the experiment platform. The samples with different graphene content placed between light source and optical power density meter to test the optical attenuation effect. (b) The detected light intensity for samples with different graphene content. Note that the light intensity is 21.26 W/cm^2.

Graphene can in principle be replaced by other materials, provided that the substitute fulfills both functions described above, i.e., efficient photothermal conversion and sufficient optical attenuation to spatially confine the phase transition. We conducted substitution experiments using representative photothermal nanoparticles, including MXene and Fe_3O_4, in place of graphene. The results confirm that

all tested substitutes successfully enabled the ejection process, albeit with varying performance levels (Supplementary Fig. 2)

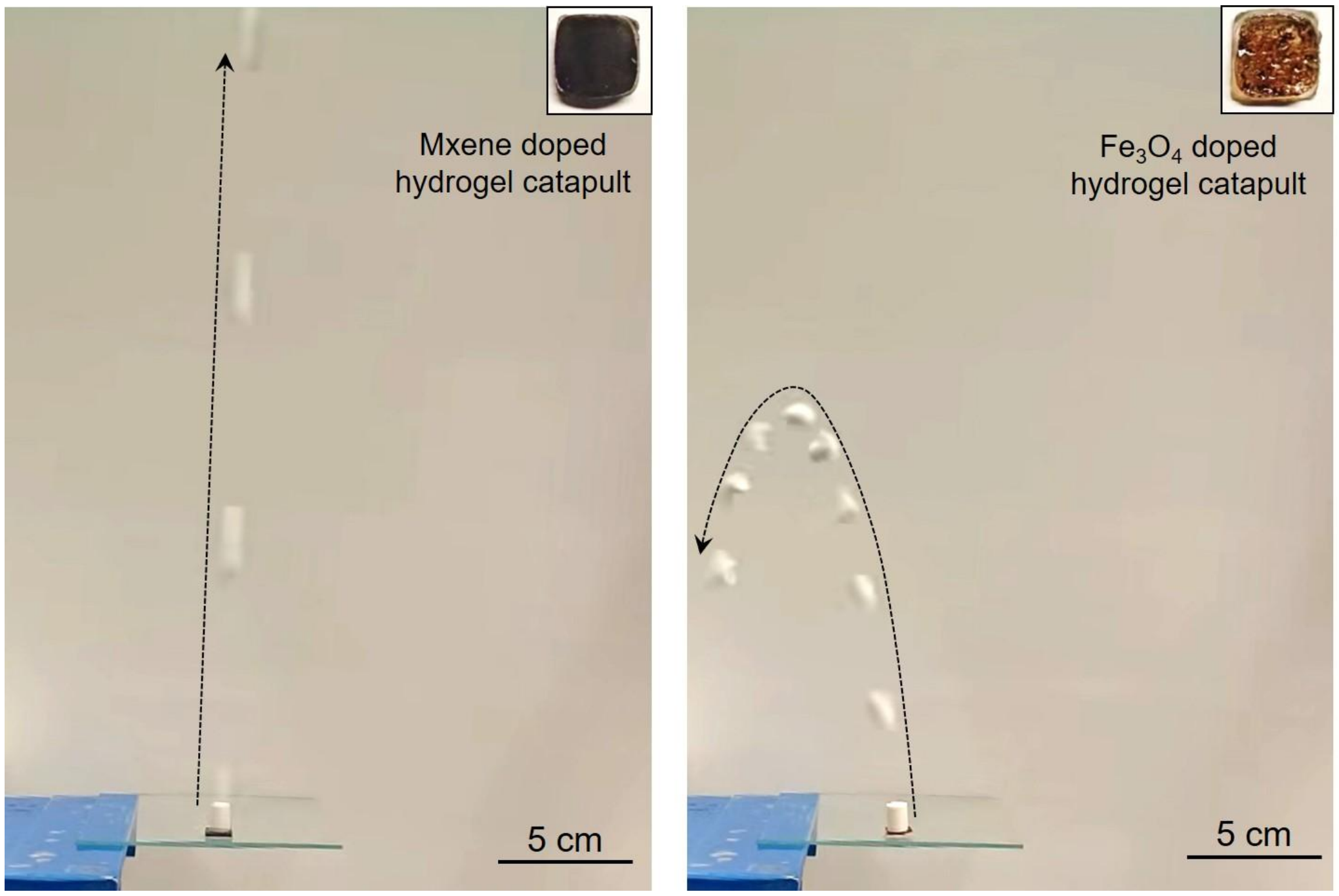

Supplementary Fig. 2. The overlaid images illustrating the ejection behavior of the Mxene and Fe_3O_4 doped hydrogel catapult.

To further investigate the effect of material substitution on the deformation process, we used high-speed camera imaging to analyze the deformation dynamics of a representative substitute system (i.e., Fe_3O_4-doped hydrogel catapult) and compared it with the original graphene-doped catapult. As shown in the Supplementary Fig. 3a, the Fe_3O_4-doped catapult exhibited smaller deformation magnitude and earlier fracture compared to the graphene-doped catapult. In contrast, the graphene-doped catapult displayed more uniform deformation and a prolonged acceleration process for the projectile (i.e., 1.8 ms *vs* 1.0 ms as shown in Supplementary Fig. 3a). The underlying reason is attributed to the dispersion homogeneity of the photothermal agent within the hydrogel matrix. As shown in Supplementary Fig. 3b, graphene can be dispersed relatively uniformly within the hydrogel, whereas Fe_3O_4 nanoparticles tend to aggregate during the polymerization process, resulting in a non-uniform spatial distribution. This inhomogeneous distribution leads to localized non-uniform heat generation and introduces structural defects in the hydrogel network, which in turn promote premature fracture and reduce the overall deformation and momentum transfer efficiency.

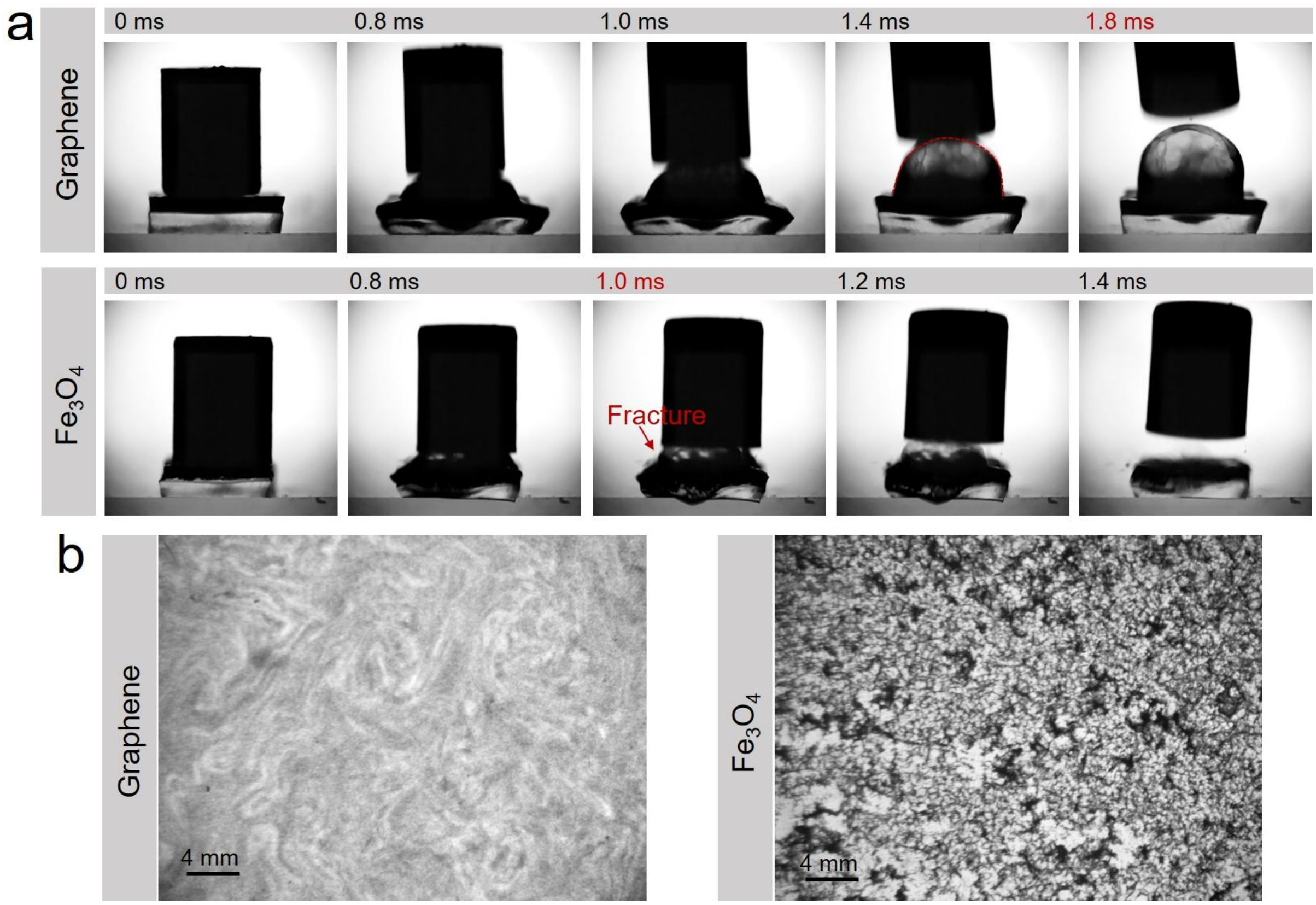

Supplementary Fig. 3. (a) The high-speed camera image illustrates the actuation process of the graphene and Fe_3O_4 doped hydrogel catapult. (b) The microscopy images showing the dispersion uniformity of the graphene and Fe_3O_4 nanoparticles in the hydrogel matrix.

Furthermore, we would like to clarify that the energy input is not restricted to NIR light. In principle, any heating method capable of inducing sufficiently rapid and localized phase transition, such as electrical resistive heating, focused ultrasound, microwave irradiation, or chemical exothermic reactions, could serve as an alternative energy source to trigger the same actuation mechanism. When using non-optical energy sources, the optical attenuation function of graphene becomes unnecessary, and the photothermal agent can be omitted entirely or replaced with materials optimized for the alternative energy modality. This versatility underscores that the core contribution of this work lies in the dynamic coupling framework, rather than in the specific choice of photothermal material or energy input method.

Supplementary Note 2: Mathematical model of the energy storage during the deformation.

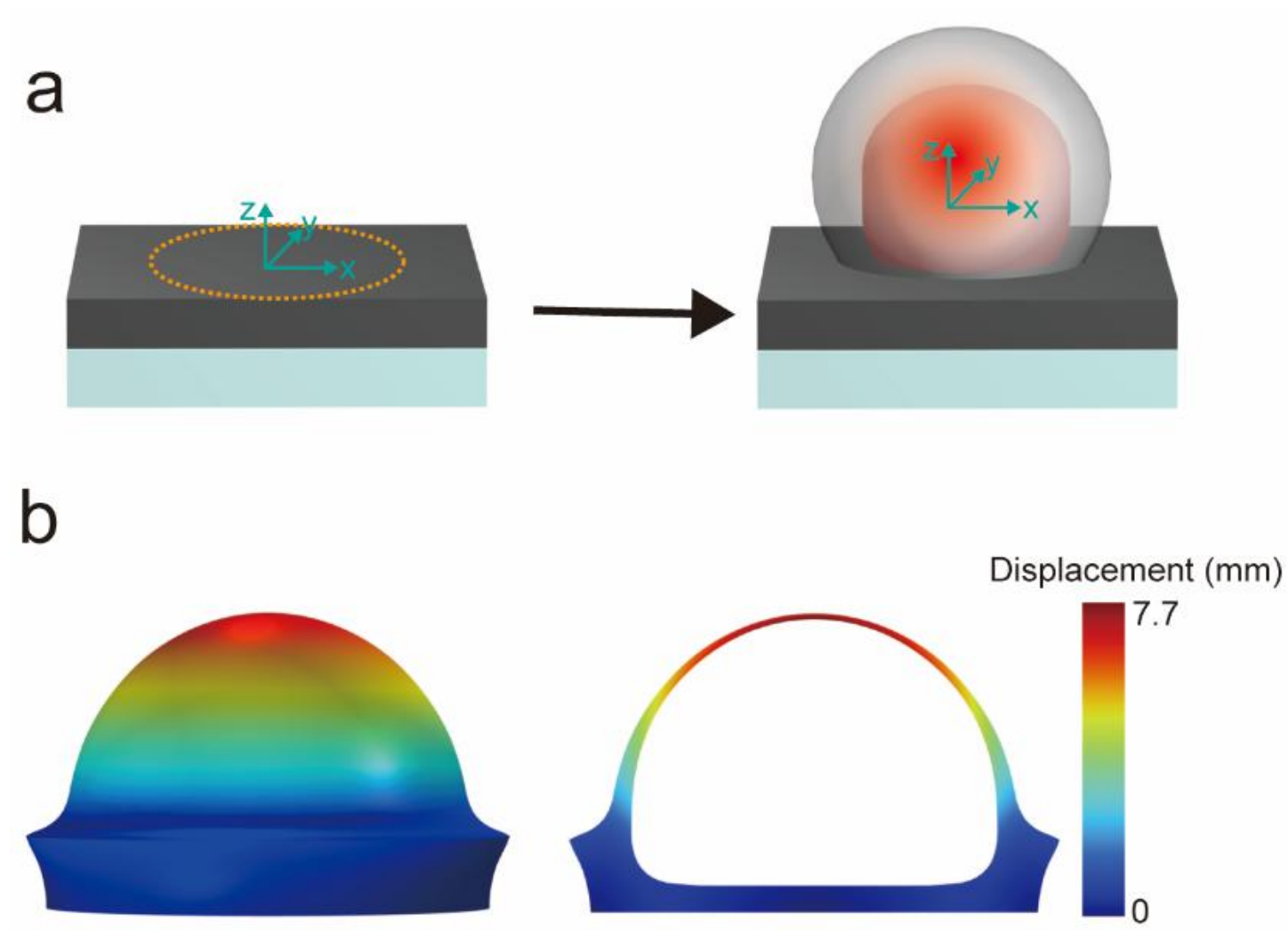

Supplementary Fig. 4. (a) Schematic illustrates the deformation process of elastic hydrogel catapult. (b) The simulation results of phase-change induced maximum deformation.

Phase transition induces the hydrogel catapult experience a rapid deformation (i.e., the elastic hydrogel membrane exhibits the spherical deformation actuated by increased internal gas pressure as shown in Supplementary Fig. 4a). The total elastic energy can be expressed as follows:

$$U_{total} = U_s + U_b \tag{1}$$

Where U_s and U_b are the strain energy caused by stretching and bending, respectively.

Among others, the U_s can be expressed as:

$$U_s = \frac{\pi E h}{1-v^2}\int_0^{r_0}(\varepsilon_r^2 + \varepsilon_t^2 + 2v\varepsilon_r\varepsilon_t)rdr \tag{2}$$

And the U_b can be expressed as:

$$U_b = \frac{D}{2}\int_0^{2\pi}\int_0^{r_0}\left[\left(\frac{\partial^2 w}{\partial r^2}\right)^2 + \frac{1}{r^2}\left(\frac{\partial w}{\partial r}\right)^2 + \frac{2v}{r}\frac{\partial w}{\partial r}\frac{\partial^2 w}{\partial r^2}\right]rdrd\theta \tag{3}$$

Where E is the Young's modulus, and h is distance from the upper surface to central of the phase change volume. r_0 is the diameter of the phase transition volume. v is the Poisson's ratio (0.48) of the material[32], r is the distance between the position of the integral differential element and the rotation axis (i.e., z-axis shown in Supplementary Fig. 4a). w is the relative longitudinal deformation displacement. And the ε_r and ε_t are the radial strain and tangential strain, respectively.

Here, the ε_r and ε_t are defined as:

$$\varepsilon_r \approx \frac{du}{dr} + \frac{1}{2}\left(\frac{dw}{dr}\right)^2 \tag{4}$$

$$\varepsilon_t = \frac{u}{r} \tag{5}$$

Where w and u are the longitudinal displacement and radial displacement, respectively, which can be measured by high-speed camera images.

According to the geometric mapping relationship before and after the deformation of hydrogel catapult, we can get:

$$u = r_1 sin\alpha - r \tag{6}$$

$$w = r_1 cos\alpha + z_1 \tag{7}$$

$$\alpha = \left(\pi - cos^{-1}\left(\frac{z_1}{r_1}\right)\right) \times \left(\frac{r}{r_0}\right) \tag{8}$$

Where r_1 is the radius of the spherical deformation of the hydrogel catapult. z_1 is the longitudinal height of spherical deformation. We simulated the deformation process as shown in the Supplementary Fig. 4b, the maximum deformation shows limited errors compared to the high-speed camera images (Fig. 1c), which depends on the mechanical properties of the hydrogel material. Based on the deformation related information obtained from Fig. 1c and Supplementary Fig. 4b, the total energy (equation (1)) can be calculated is 84 mJ.

The kinetic energy $E_{ejection}$ that projectile acquired can be calculated as follows:

$$E_{ejection} = \frac{1}{2}mv^2 \tag{9}$$

We use the data shown in Fig. 1c to do the calculation. Where m is the mass of projectiles (203 mg), The take-off velocity of the projectile can be calculated from the recorded launching height (h) by considering the post-take-off motion of projectile until reaching the maximum launching height (Fig. 1c) as a deceleration motion driven by the gravity and the air drag force[33]:

$$v_{take-off} = \sqrt{\frac{\left[e^{\frac{hC_d\rho\pi r_0^2}{m}} - 1\right] \times 2mg}{C_d\rho\pi r_0^2}} \tag{10}$$

Where C_d is the drag coefficient (0.77)[34], ρ is the density of air, r_0 is the basal radius of cylindrical projectile (r_0 = 4 mm), m is the mass of the projectile (203 mg) and g is the gravitational acceleration.

The kinetic energy is calculated to be 27.97 mJ based on the equation (9). The strain-kinetic energy transfer efficiency can be calculated to 33.29%.

The input energy (NIR light energy) can be calculated by:

$$E_{input} = P_{laser}t \tag{11}$$

where P_{laser} is the power of the applied laser, t is the laser irradiation time (~ 2 s) before the deformation of the hydrogel catapult. The calculated external input energy is 21.36 J. The kinetic energy of the launched projectile is 27.97 mJ, which results in an energy efficiency of 0.13 %

The power density can be calculated through:

$$P_m = \frac{P}{m_{catapult}} \tag{12}$$

Where P_m is the power density, $m_{catapult}$ is the mass of the hydrogel catapult. P is the peak power output of the hydrogel catapult, which can be calculated as:

$$P = \frac{E_{ejection}}{t} \tag{13}$$

Where t is the actuation time (1.6 ms), $E_{ejection}$ is 27.97 mJ based on the equation (9), therefore the power density can be calculated to 8.74×10^4 W/kg based on the equation (9) - (13).

Supplementary Note 3: Influence factors of the ejection performance of the hydrogel catapult.

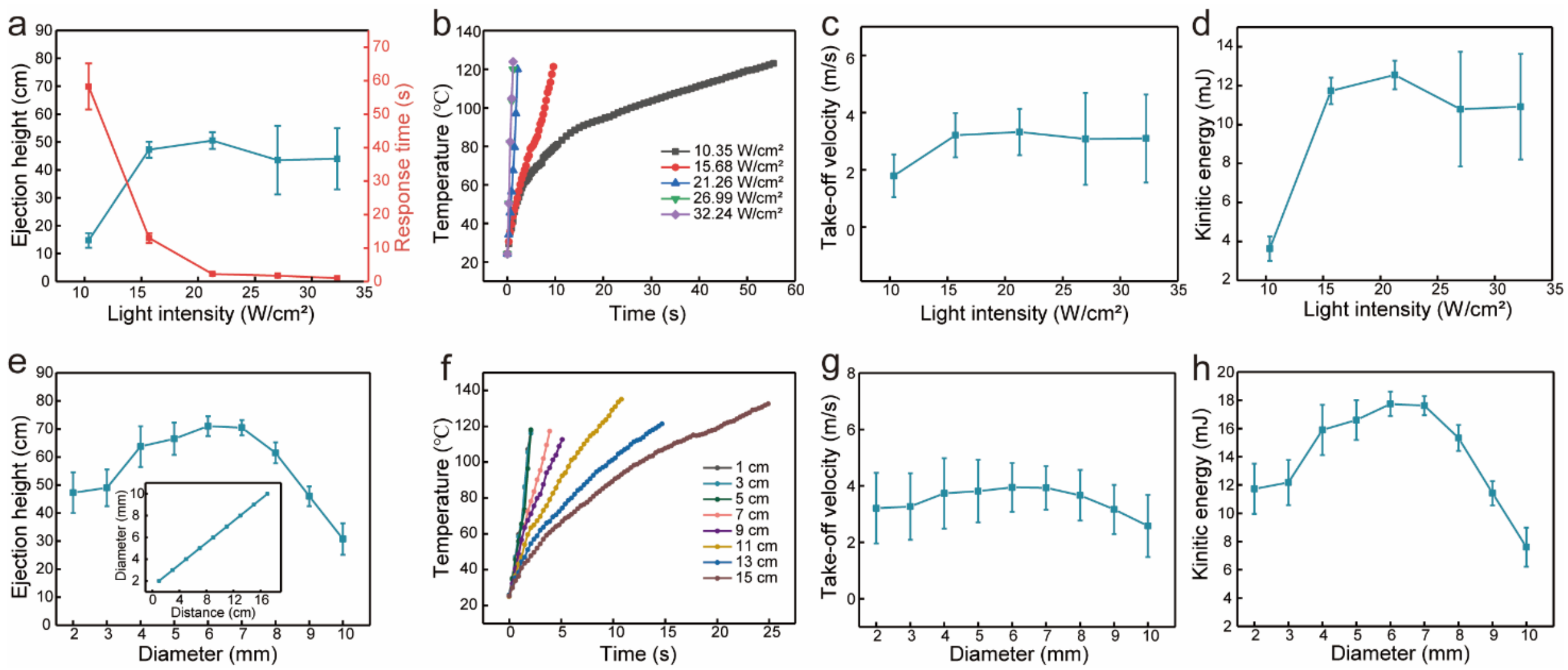

Supplementary Fig. 5. Light-dependent ejection performance of NIR-actuated hydrogel catapults. (a) The ejection height and response time of the hydrogel catapult with different light intensity. Data points are shown as mean ± s.d. (n = 4). (b) The temperature change of the upper layer for the catapult under NIR light irradiation with different light intensity. (c) The calculated take-off velocity and (d) kinetic energy of the ejected projectiles. Data points are shown as mean ± s.d. (n = 4) (e) The ejection height of the hydrogel catapult with different diameter of light spot (Inset: The light spot diameter increases with the increase of the distance between light source and hydrogel catapult). Data points are shown as mean ± s.d. (n = 4). (f) The temperature change of the upper layer for the catapult under NIR light irradiation with different distance between light source and hydrogel catapult. (g) The calculated take-off velocity and (h) kinetic energy of the ejected projectiles. Data points are shown as mean ± s.d. (n = 4). Note that the light intensity is 21.26 W/cm^2 and all the projectile's weight in Supplementary Fig. 5 is 2280 mg.

We investigate the factors that influence the ejection performance of the hydrogel catapult in terms of light energy input (light intensity and light spot size), geometric design (thickness of functional layer), and materials composition (graphene content, water content and crosslinker (MBA) content). First, the input of the NIR light energy influence the ejection performance of the hydrogel catapult. As shown in Supplementary Fig. 5a and Fig. 5b, a weak light intensity leads to the slow vaporization rate, prolonged irradiation period and limited deformation intensity and speed, that induces the reduced force output and energy transfer. The ejection height increases as the increases of the light intensity. However, it shows limited difference when the light intensity higher than 15 W/cm^2, potential reason is the sufficient light energy input in short period results in response time and deformation speed being very similar, which induced the similar power output. It is worth noting that under high-intensity illumination (i.e., >21.26 W/cm²), the ejection performance exhibits greater variability, as reflected by larger error

bars. This may be attributed to an excessively rapid phase transition, which induces non-uniform deformation and consequently leads to variations in deformation speed and force output.

Based on the recorded ejection height and accounting for air drag, we calculated the take-off velocity of the projectile, as presented in Supplementary Fig. 5c. The take-off velocity initially increases with rising light intensity and subsequently approaches a plateau. In addition, when considering the effect of light intensity on the payload capacity of the hydrogel catapult, it is important to recognize that the weight of the projectile influences the output dynamics (details shown in coupled framework proposed in Fig. 2). As expected, an increase in projectile weight results in a macroscopic reduction in ejection height, which eventually approaches zero. However, under high-load conditions, the ejection output exhibits pronounced instability and is subject to the combined influences of both light intensity and projectile mass. Consequently, it is challenging to derive a precise quantitative reference for the maximum electable weight. To address this, we qualitatively assess the payload capacity by evaluating the kinetic energy acquired by a projectile of fixed weight under varying light intensities. As illustrated in Supplementary Fig. 5d, the acquired kinetic energy increases with light intensity before reaching a plateau. This indicates that when the light intensity exceeds a certain threshold, the mechanical output of the hydrogel catapult stabilizes, and the corresponding payload capacity remains approximately constant.

In addition, the light spot area influences the power output by modulating both the phase transition volume and the response time. We adjusted the distance between the light source and the sample to vary the spot area (inset of Supplementary Fig. 5e). As the spot area increases, the ejection height initially rises, which can be attributed to an enhanced force output resulting from a larger deformation volume. However, an excessively large spot area (i.e., diameter > 7 mm) leads to a reduction in optical power density and a prolonged response time (Supplementary Fig. 5f), ultimately impairing both force output and ejection performance. A plausible explanation for this decline is that a larger spot area fails to induce a uniform phase transition across the entire irradiated region, thereby giving rise to heterogeneous deformation and inconsistent force output. Similarly, we calculated the initial velocity and kinetic energy of the projectile from the recorded ejection height, as shown in Supplementary Fig. 5g and 5h. Both the initial velocity and the acquired kinetic energy exhibit a non-monotonic dependence on the spot diameter, first increasing and then decreasing. The maximum payload capacity (i.e., the peak mechanical output) of the hydrogel catapult is achieved at a spot diameter of approximately 6 - 7 mm. A smaller spot size limits the overall deformation volume, whereas an excessively large spot size induces non-uniform deformation and premature structural failure, both of which compromise the payload capacity.

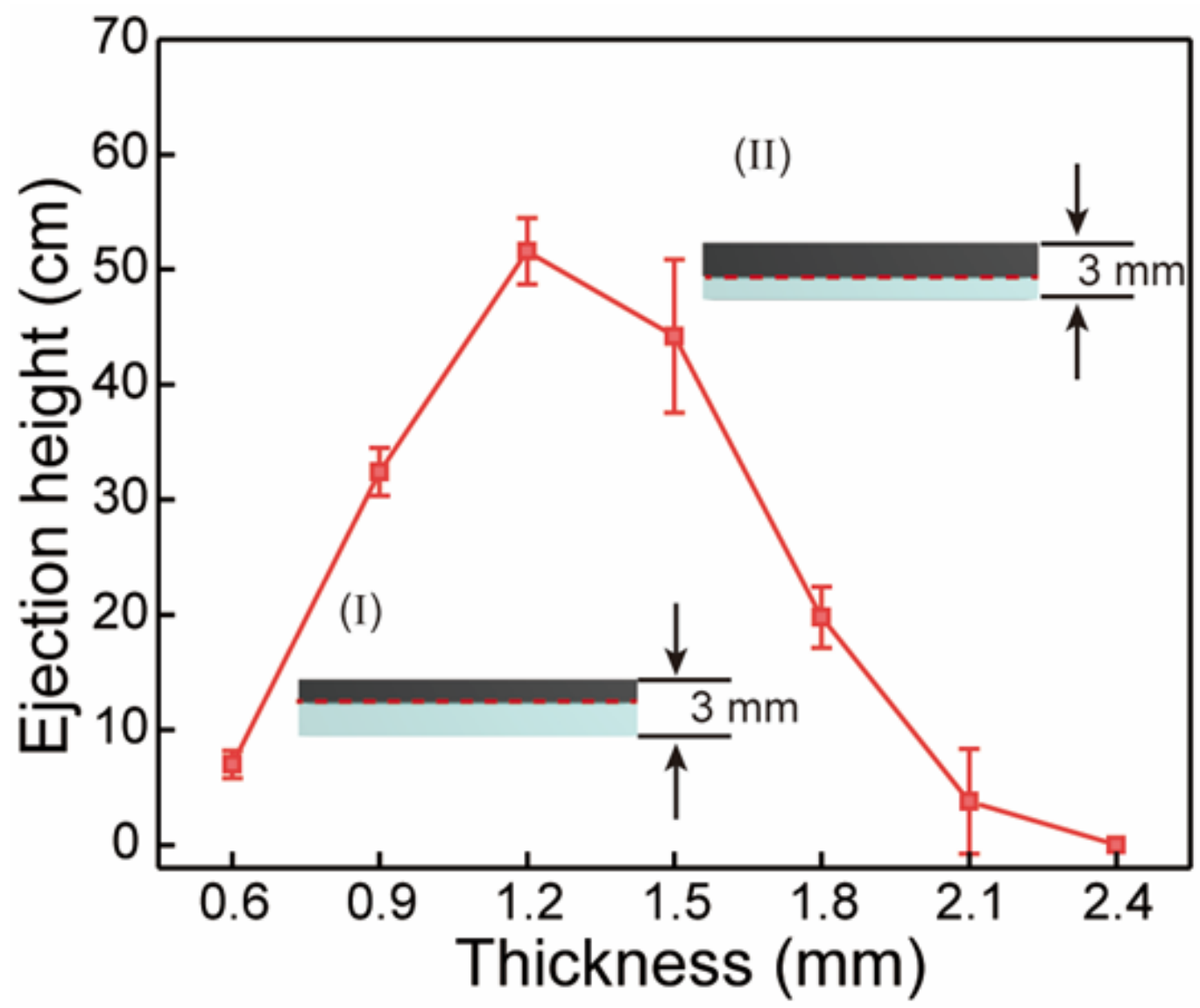

Supplementary Fig. 6. The ejection height of the hydrogel catapult with different thickness of the functional layer (upper layer). Note that the total thickness of the hydrogel catapult maintained as 3 mm. Data points are shown as mean ± s.d. (n = 5).

The thickness of the functional layer (i.e., the upper layer doped with graphene) significantly effects the ejection performance. As illustrated in Supplementary Fig. 6, the overall thickness of the hydrogel catapult is maintained at 3 mm, with variations in the functional layer thickness. Due to the blocking effect of graphene, the penetration depth of NIR light is limited. Consequently, changes in the thickness of the functional layer can alter the phase-transition volume, which in turn affects the strength and speed of the deformation. For instance, if the functional layer is too thin, the phase transition occurs relatively close to the upper surface, allowing only limited deformation and decreased energy transfer period that inducing the reduced ejection performance. Conversely, as the thickness of functional layer continues increased until thicker than 1.2 mm, make the upward deformation process difficult and reduced deformation amount, the excessive energy loss in overcoming the elastic constraint thus inducing the ejection performance decreased. Therefore, the functional layer needs to be carefully adjusted in order to balance the trade-off between functional layer thickness and elastic deformation (Supplementary Fig. 6).

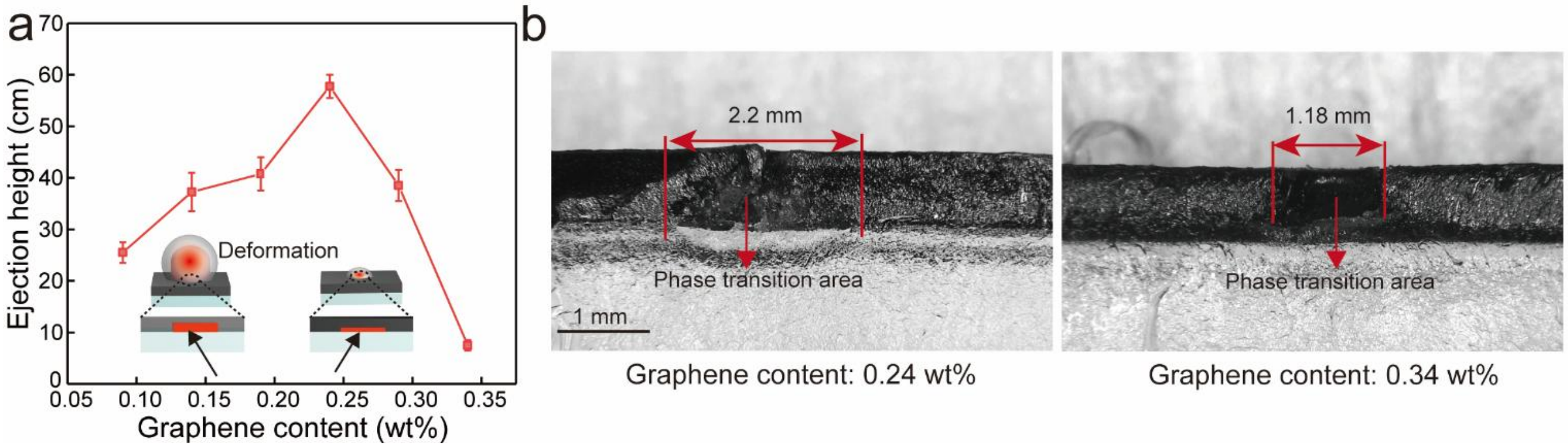

Supplementary Fig. 7. (a) The ejection height of the hydrogel catapult with different graphene content. Data points are shown as mean ± s.d. (n = 4). (b) The microscope images of the cross-section of hydrogel catapult with different graphene content after actuation indicating the difference of the phase transition volume.

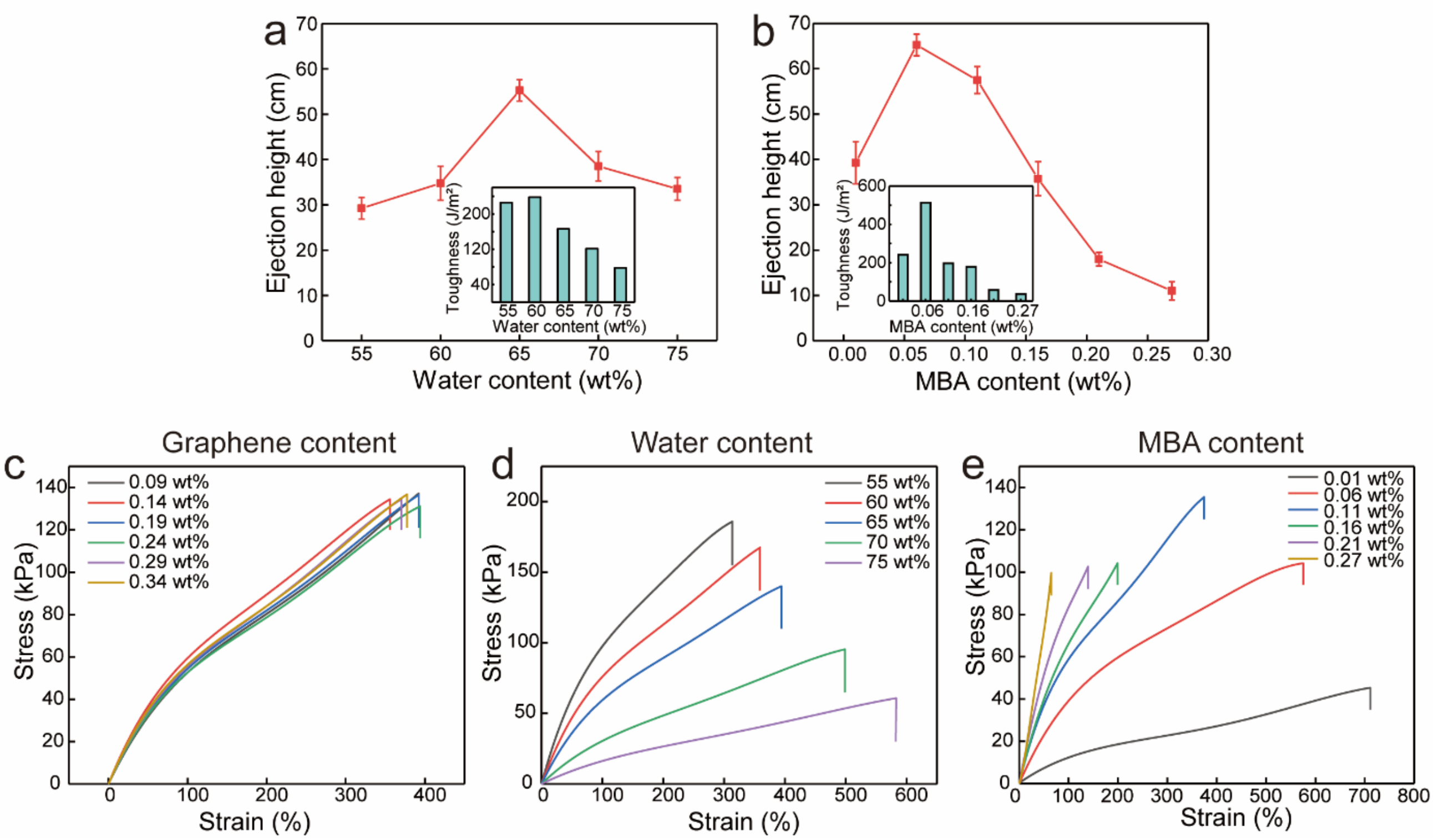

Supplementary Fig. 8. The ejection height of the hydrogel catapult with different (a) water content and (b) crosslinker (MBA) content. Data points are shown as mean ± s.d. (n = 4). (Inset): The toughness of the hydrogel with different (a) water content and (b) MBA content. (c) The stress-strain curves of hydrogel samples with different (c) graphene content, (d) water content and (e) MBA content.

The materials composition (i.e., graphene content, water content and crosslinker content) of the catapult tailors the deformation and ejection performance. As shown in Supplementary Fig. 7a, the deformation originates from the stream expansion (phase change of water) inside the hydrogel caused by photothermal effect of the graphene, the ejection performance initially increased first as the graphene content increases

from 0.09 wt% to 0.24 wt%. Higher graphene content induces the more thermal production and water vaporization within short actuation period, endowing the deformation with higher intensity and faster speed, thus realizing the better ejection performance. However, the excess graphene content (i.e., > 0.24 wt%) can induces the phase change volume decreased due to the limited penetration depth caused by the blocking effect of the black graphene and extremely quick response speed, thus produce the small deformation and decreased ejection performance (Supplementary Fig. 7a and Fig. 7b). In addition, the water content and MBA content effects the energy accumulation through changing the mechanical properties of hydrogel catapult that resulting in different deformation speed and force output. As shown in Supplementary Fig. 8a, the ejection performance increases first and then decrease as the water content increases, which related to the energy accumulation ability depends on the toughness of the hydrogel network as shown in the inset of Supplementary Fig. 8a (i.e., similar trend of toughness first increase and then decrease). The more energy accumulation means bigger deformation and resistance ability of faster deformation speed induced impact, which increase the momentum acquisition of the projectiles. Moreover, the effect of the MBA content on the ejection performance exhibits a similar tendency with water content, as presented in Supplementary Fig. 8b. The best performance is achieved at a water content and MBA content of around 65 wt% and 0.06 wt%, respectively. It should be noted that the mechanical properties are mainly adjusted through the water content and MBA content, while the graphene content does not affect it in a significant way (Supplementary Fig. 8c-8e).

Supplementary Note 4: Influence factor of substrate on ejection performance of the hydrogel catapult

To analyze the influence of substrate stiffness on the momentum transfer to the projectile, we established a spring-mass model as shown in Supplementary Fig. 9. In this model, the projectile serves as the mass block. The ejection process of the hydrogel catapult can be simplified as a spring is compressed to store energy and then released to drive the mass block. The spring is anchored to a rigid substrate to simulate the glass substrate with a fixed constraint.

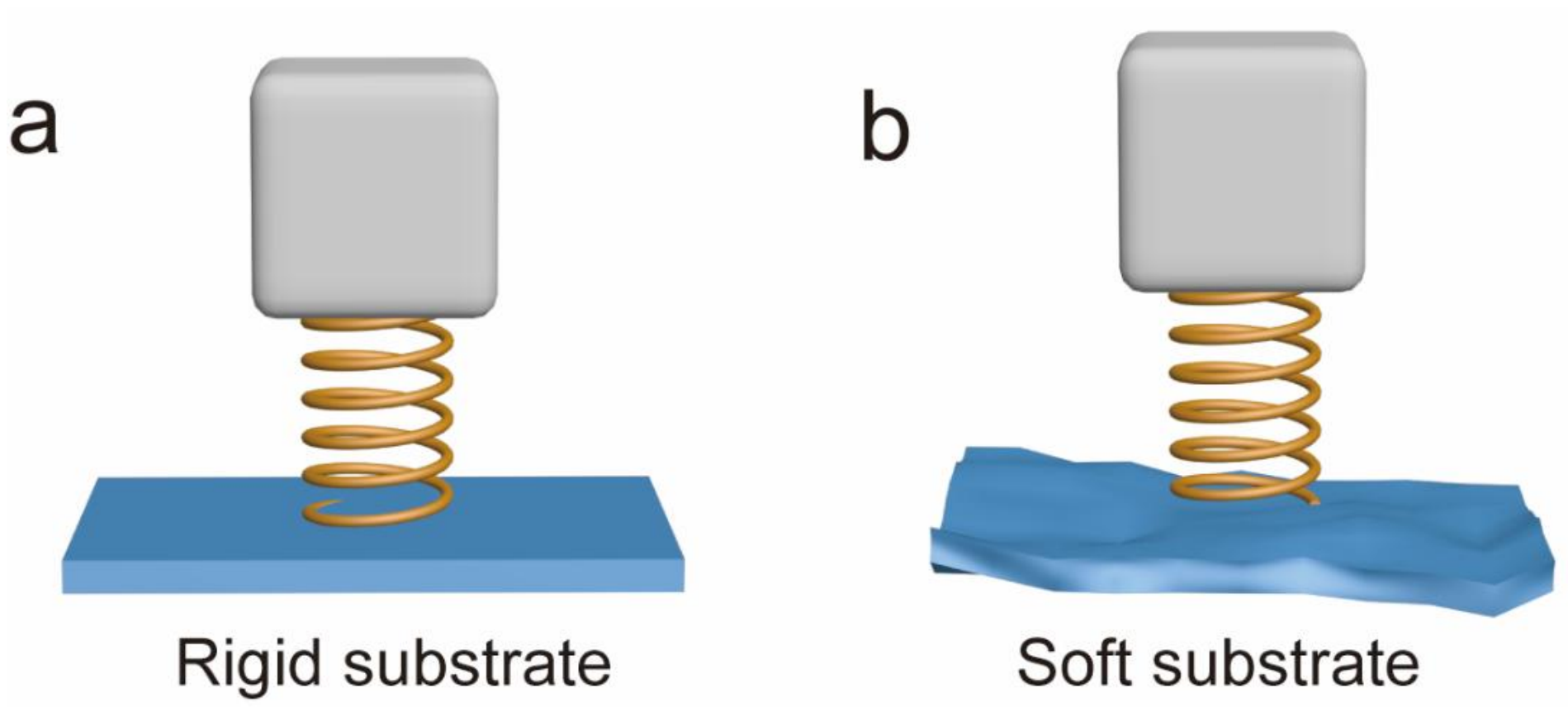

Supplementary Fig. 9. Schematic illustration of the mass-spring model on rigid substrate and soft substrate.

The kinetic energy obtained of the mass block is shown as follows (rigid substrate case),

$$\frac{1}{2}mv^2 = E_{spring} = \frac{1}{2}kx_0^2 \tag{14}$$

When the substrate is a viscoelastic soft material, the mass-spring-damper series model is used to analyze the energy transfer mechanism, the motion of the system can be expressed as follows:

$$m\ddot{x} + c\dot{x} + kx = 0 \tag{15}$$

Where *m* is the mass of the mass block, *c* represents the damping coefficient of the damper, *k* is the stiffness coefficient of the spring.

The displacement response and velocity response shown as follows,

$$x(t) = x_0 e^{-\zeta\omega_n t} cos(\omega_d t) \tag{16}$$

$$v(t) = -x_0\omega_n e^{-\zeta\omega_n t}\left[\zeta cos(\omega_d t) + \sqrt{1-\zeta^2} sin(\omega_d t)\right] \tag{17}$$

Where $\zeta = \frac{c}{2\sqrt{km}}$ is the damping ratio, m is the mass and k is the stiffness of the substrate, respectively. The undamped natural frequency (ω_n) and damped natural frequency (ω_d) are shown as follows:

$$\omega_n = \sqrt{\frac{k}{m}}, \omega_d = \omega_n\sqrt{1-\zeta^2} \tag{18}$$

Assuming that when the spring returns to its original length, the mass block begins to separate from the spring, and the initial velocity of the mass block can be expressed as:

$$v = \sqrt{\frac{k}{m}} x_0 \sqrt{1-\zeta^2} e^{-\frac{\zeta\pi}{2\sqrt{1-\zeta^2}}} \tag{19}$$

Based on equations (14) and (19), soft substrate induces a damping effect that that reduces the initial momentum acquisition. Compared to the substrate fabricate with soft material, the rigid substrate with higher stiffness endows the mass block a faster initial velocity.

Supplementary Note 5: Influence factor of projectile weight on ejection performance

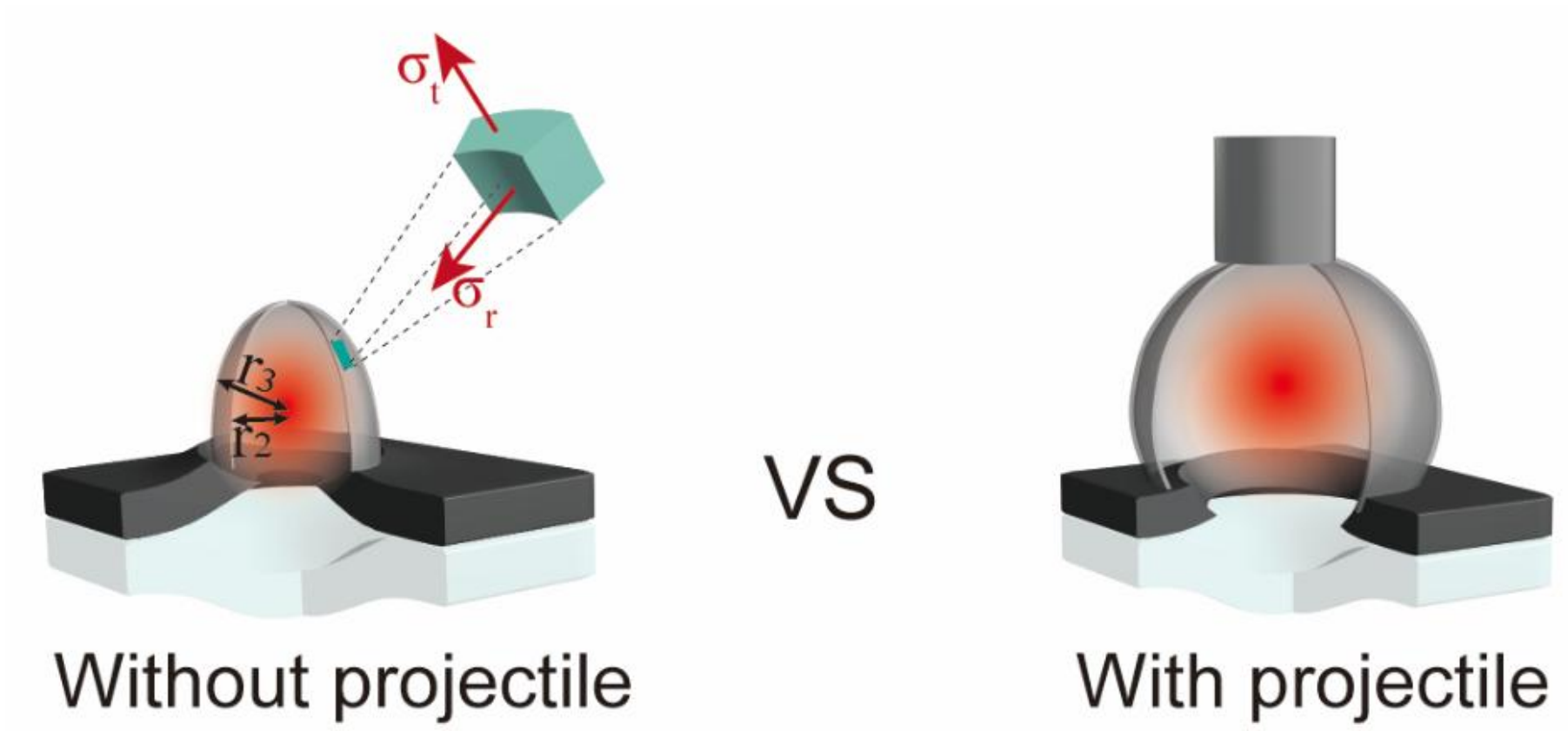

Supplementary Fig. 10. Schematic of the force analysis for a deformed hydrogel with and without a projectile on its upper surface.

The weight of the projectile can affect the internal pressure distribution during the hydrogel deformation and thus changing the energy storage and release. In case of no projectile on the hydrogel catapult, the stress equilibrium equation of the spherical deformation can be expressed as (Supplementary Fig. 10):

$$\frac{d\sigma_r}{dr} + \frac{2(\sigma_r - \sigma_t)}{r} = 0 \tag{20}$$

Where σ_r is the radial stress and σ_t is the tangential stress. Through the introduction of the boundary conditions,

The internal surface of the deformed hydrogel catapult is shown as follows:

$$\sigma_r = -p \ (r = r_2) \tag{21}$$

The external surface of the deformed hydrogel catapult can be expressed as:

$$\sigma_r = 0 \ (r = r_3) \tag{22}$$

Where p is the pressure inside the deformation volume. r_2 and r_3 are the radius from inner surface and outer surface of the spherical deformation, respectively.

Combining the boundary conditions and constitutive model, the expression of its internal stress distribution can be obtained:

$$\sigma_r = p \frac{r_2^3}{r_3^3 - r_2^3}\left(1 - \frac{r_3^3}{r^3}\right) \tag{23}$$

$$\sigma_t = p\frac{r_2^3}{r_3^3 - r_2^3}\left(1 + \frac{r_3^3}{2r_2^3}\right) \tag{24}$$

The internal pressure increases as the phase-transition induced the gas increases, and the maximum tangential stress can be expressed as:

$$[\sigma_t]_{max} = p\frac{2r_2^3 + r_3^3}{2(r_3^3 - r_2^3)} \tag{25}$$

When a projectile is placed on the top surface of the deformed hydrogel catapult, the total stress within the spherical deformation is the sum of the stress induced by the internal gas pressure and the stress caused by the weight of the projectile. The effect of the projectile weight is simplified by considering the stress act uniformly on the outer surface of the deformed hydrogel membrane, which is equivalent to an external pressure. Based on the principle of superposition, the stress distribution under the combined force of the projectile weight and internal pressure can be expressed as:

$$\sigma_r = \frac{1}{r_3^3 - r_2^3}\left[pr_2^3\left(1 - \frac{r_3^3}{r^3}\right) - \frac{Fr_3^3}{4\pi}\left(1 - \frac{r_2^3}{r^3}\right)\right] \tag{26}$$

$$\sigma_t = \frac{1}{r_3^3 - r_2^3}\left[pr_2^3\left(1 + \frac{r_3^3}{2r^3}\right) - \frac{Fr_3^3}{4\pi}\left(1 + \frac{r_2^3}{2r^3}\right)\right] \tag{27}$$

Where r represents the radius of curvature of the element on the spherical shell and the F is the gravity of projectile, respectively.

The maximum stress of the internal surface can be expressed as:

$$[\sigma_t]_{max} = p\frac{2r_2^3 + r_3^3}{2(r_3^3 - r_2^3)} - \frac{3Fr_3^3}{8\pi(r_3^3 - r_2^3)} \tag{28}$$

Compared to the equation (25) that without the projectile on the deformed hydrogel catapult, the maximum internal tangential stress will be reduced as the projectile weight increased, delaying the fracture of the elastomer and inducing more kinetic energy transferred to projectile.

Supplementary Note 6: Physical model analysis for different projectiles in Fig. 3f with different travel distance.

We use hydrogel microspheres as the fundamental unit to simulate spores for systematic comparison, i.e., a single hydrogel sphere (representing individual spores), unencapsulated aggregate (a cluster combined with 20 hydrogel spheres), and sporangium analogue (20 hydrogel spheres encapsulated in an elastic film). The results of the landing statistics reveal that the sporangium analogue achieved the longest ejection distance. The three types of projectiles acquire the same initial take-off velocity during the actuation process, however, their travel distances are different due to variations in gravitational effects and air resistance encountered during flight. The detailed analysis is as follows:

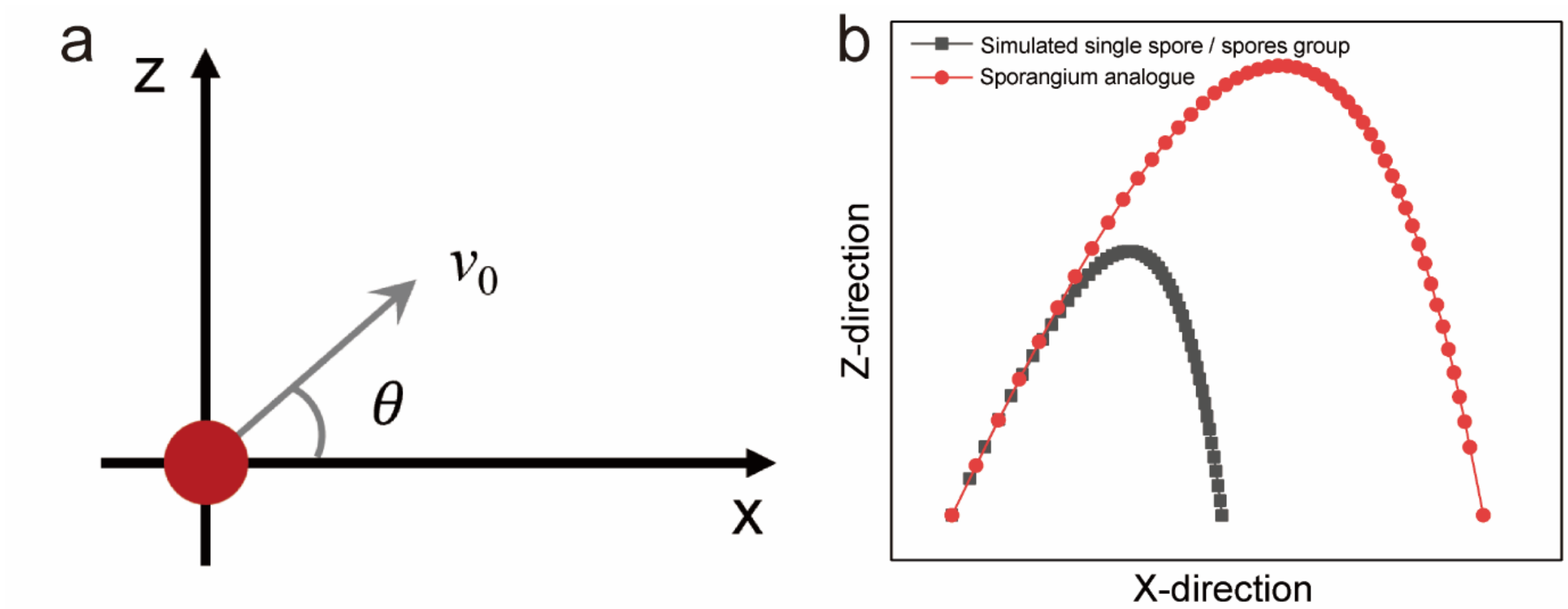

Supplementary Fig. 11. (a) Schematic illustration of the take-off velocity. (b) The trajectory comparison of the simulated single spore / spores group with sporangium analogue that considered air resistance.

The projectiles were assumed to be spherical, and the trajectories were analyzed under aerodynamic drag. During flight, each projectile is subjected to both gravitational force and air resistance, with an initial velocity of v_0 and an ejection angle of θ (Supplementary Fig. 11a), the flying speed is v, the drag force (F_d) can be calculated through:

$$F_d = \frac{1}{2} C_d \rho A v^2 \tag{29}$$

Where C_d is the drag coefficient, ρ is the density of air, A represents the frontal area of the projectile that can be calculated by $A = \pi r^2$, where r is the ratio of the projectile.

During flight, the force for launched projectiles in both horizontal and vertical directions can be expressed as:

$$m \frac{dv_x}{dt} = -\frac{1}{2} C_d \rho A \sqrt{v_x^2 + v_y^2} \cdot v_x \tag{30}$$

$$m \frac{dv_y}{dt} = -mg - \frac{1}{2} C_d \rho A \sqrt{v_x^2 + v_y^2} \cdot v_y \tag{31}$$

$$v_x = \frac{dx}{dt} \tag{32}$$

$$v_y = \frac{dy}{dt} \tag{33}$$

Where m is the mass of the projectiles. v_y and v_x represent the vertical and horizontal component of the velocity, respectively.

Combined with the initial conditions:

$$\begin{cases} v_x(t=0) = v_0 cos\theta \\ v_y(t=0) = v_0 sin\theta \\ x(t=0) = 0 \\ y(t=0) = 0 \end{cases} \tag{34}$$

Where v_0 is the take-off velocity of the projectiles. θ is the ejection angle. The trajectory of the projectile and landing distance can be determined for a given initial velocity v_0 and ejection angle θ.

A comparative analysis of the initial conditions is conducted for the following two scenarios:

(1) For the unencapsulated aggregate (i.e. a cluster combined with N identical hydrogel spheres simultaneously, each with mass m_{sphere} and initial velocity v_{0i})

(2) For the sporangium analogue (assume with shape of sphere) that composed of the N hydrogel spheres, with total mass $M=N\cdot m_{sphere}$ and the initial velocity is v_0.

The initial kinetic energy is equal in both cases:

$$N \cdot \left(\frac{1}{2} m v_{0i}^2\right) = \frac{1}{2} M v_0^2 \tag{35}$$

We can get the $v_{0i} = v_0$, it means that the take-off velocity is same for the unencapsulated aggregate and sporangium analogue.

After the calculation based on the above equations, the comparison of the trajectory for unencapsulated aggregate (one of a hydrogel sphere for the cluster) and sporangium analogue (20 hydrogel spheres encapsulated in an elastic film) are shown as Supplementary Fig. 11b. The results and physical analysis indicate that the sporangium analogue exhibits longer flight duration and travel distance compared to a single spore (i.e., hydrogel sphere). The influence factors mainly from air resistance.

Supplementary Note 7: Systematic comparison of developed biopsy device with existing biopsy tools.

We have compared the developed biopsy device with existing tools across the five dimensions, as shown in Supplementary Table 3.

(i) Delivered impulse and force output. The hydrogel catapult delivers ~2.34 N of instantaneous force (Supplementary Fig. 22), falling within the clinically relevant range for soft tissue needle insertion (1-5 N)[35]. To place this in context, it is instructive to examine the landscape of existing biopsy tools. Spring-loaded biopsy guns can generate substantial forces through pre-loaded spring mechanism, however, these are rigid, bulky handheld devices designed exclusively for percutaneous procedures (e.g., breast, liver, kidney biopsy) and are fundamentally incompatible with intraluminal GI tract access. They cannot be miniaturized to a form factor suitable for endoscopic delivery, nor can they be used within the GI lumen under any clinical scenario. On the other end of the spectrum, tools that can access the GI tract intraluminally, including EUS-FNA/FNB needles and catheter-based robotic systems, are limited to fine needle gauges and modest force output (typically < 0.5 N for soft robotic systems, and while manual FNA can nominally deliver 1-5 N, this force is significantly attenuated when transmitted through a curved endoscope, particularly under retroflexion)[35]. Our hydrogel catapult-based biopsy system bridges this critical gap by providing Newton-level, locally generated force (~ 2.34 N) that can be delivered into the GI lumen, a combination of force output and intraluminal accessibility that is currently unavailable in clinical practice.

Supplementary Table 3. Comparison of the hydrogel catapult biopsy system with existing biopsy tools.

**Aspect**	**Manual FNA/FNB**	**Spring-loaded biopsy devices**	**Catheter-based systems**	**This work**
Force output	~ 1-5 N[35] (operator dependent)	High (spring dependent)	Typically < 0.5 N;	2.34 N
Force generation mode	Remote (transmitted through endoscope)	Remote (spring pre-loaded externally)	Remote (transmitted through endoscope)	Local (generated at tissue interface)
Needle gauge	19-25 G[36, 37]	14-18 G[38] (mainly for percutaneous biopsy)	19-25 G[39]	Customizable; The local force output allows the needle thicker than 14G
Single-pass adequacy	Often insufficient; typically 3-7 passes required	High (core tissue obtained)	Often insufficient	High (thicker needle and sufficient force)

GI tract intraluminal access	Yes	Not applicable	Yes	Yes
Depth control	Operator tactile judgment	Yes	Varies	Mechanical depth stop
Actuation speed	Manual (~s)	Fast (~ms); (mainly for percutaneous biopsy)	Typically slow (~s)	Fast (ms)

(ii) Access, precision and penetration control. The key advantages of our device lie in its high output force and endoscope compatibility. Furthermore, our device can be further miniaturized, potentially enabling integration into wirelessly driven capsule robots. Penetration precision is achieved through two complementary mechanisms: (1) a stepped structure within the PDMS shell that mechanically limits the maximum needle travel distance, providing a hard physical stop analogous to the depth-limiting mechanisms in percutaneous biopsy guns, but now implemented in an intraluminal device. (2) A guide channel with a slightly larger diameter than the needle that corrects needle orientation during ejection, ensuring vertical insertion and reducing angular misalignment (as shown in Fig. 5a). These mechanical constraints provide reliable, operator-independent depth and directional control, which is valuable in the retroflexed configuration where manual precision is most severely compromised. Furthermore, the system is compatible with real-time endoscopic imaging for spatial guidance to the target site. While we acknowledge that the current directional precision is not yet comparable to dedicated robotic needle guidance systems, the mechanical constraints provide a robust baseline level of penetration control suitable for the proof-of-concept stage.

(iii) Safety and thermal considerations.

Although the internal temperature of the graphene-doped hydrogel layer exceeds 120 °C during phase transition, the PDMS encapsulation provides effective thermal isolation. We used infrared thermography to record the temperature variation on the device surface (i.e., the surface in contact with the tissue) during actuation. The measured peak temperature at the robot-tissue interface was 40.3 °C as shown in Supplementary Fig. 12, which is below the widely accepted threshold for thermal tissue damage (generally defined as > 43 °C for sustained exposure exceeding 60 minutes, or > 50 °C for acute thermal injury)[40]. Moreover, the actuation event lasts only ~1.6 ms, resulting in negligible cumulative thermal dose to surrounding tissue. Additional safety features include the PDMS shell prevents NIR light leakage to tissue. The optical fiber is enclosed within the sealed structure, eliminating direct light exposure to tissue. And the mechanical depth limiter provides an additional critical safety layer, particularly

important in thin-walled anatomical regions such as the gastric fundus where uncontrolled penetration depth poses significant perforation risk.

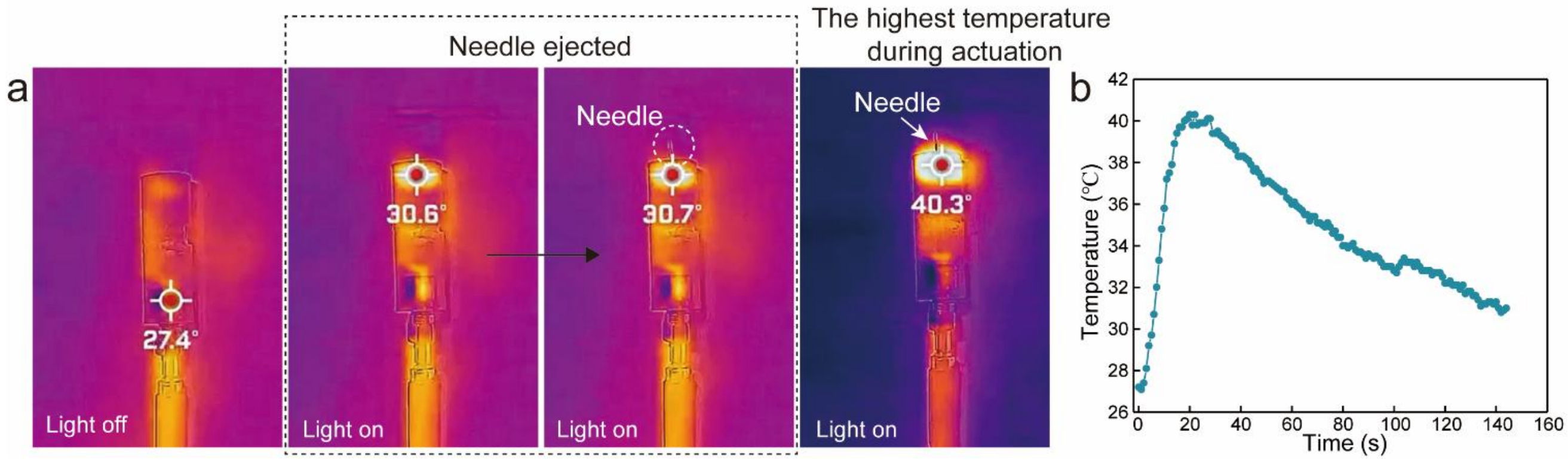

Supplementary Fig. 12. (a) A series of infrared thermal images and (b) temperature change profile showing the surface temperature during the biopsy procedure.

(iv) Potential tissue trauma. We acknowledge that the use of a thicker needle inherently creates a larger puncture wound compared to fine-needle aspiration. However, this trade-off may be mitigated by two factors. First, the ultrafast actuation (~1.6 ms) means the needle reaches full insertion depth before significant tissue deformation occurs. Published studies on needle insertion biomechanics have demonstrated that higher insertion velocities reduce tissue displacement, dimpling, and associated mechanical trauma compared to slow manual insertion[41, 42]. Second, the thicker needle enables adequate tissue acquisition in a single pass, potentially eliminating the need for multiple punctures. Furthermore, following the reported module design principle, the hydrogel catapult is expected to be integrated into the repair module to avoid potential adverse consequences such as bleeding.

In summary, in the landscape of tissue sampling tools, it remains necessary to develop devices with sufficient force for deep tissue penetration and suitable integrated with endoscopy to acquire sufficient samples in single time biopsy. The hydrogel catapult-based biopsy system demonstrated in this work represents a first step toward resolving this challenge, by introducing a "local force generation" method that enables Newton-level force output within a miniaturized, endoscopically deliverable form factor. This capability is enabled by the dynamic coupling mechanism, the core scientific contribution of this work, which provides the physical foundation for achieving high power and force output in miniature soft systems.

In addition, this biopsy device is at the proof-of-concept stage and has not undergone systematic biocompatibility or safety testing required for clinical translation. The purpose of this system we have developed is to help better demonstrate our core contribution, i.e., the dynamic coupling framework, and

to better help readers understand how this mechanism enables the miniature robots achieve efficient remote and non-contact operation in confined spaces.

3. Supplementary Figures

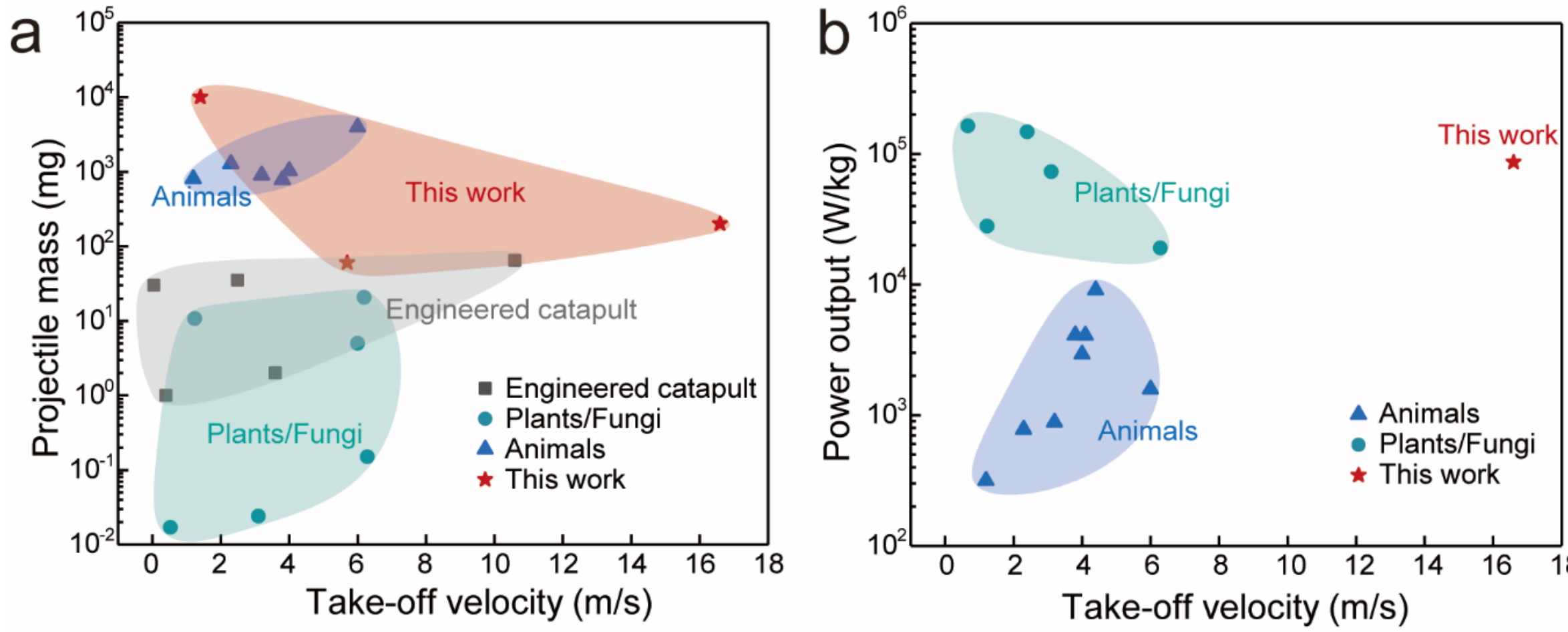

Supplementary Fig. 13. (a) Ashby plot of the projectiles mass and take-off velocity comparison of the light driven hydrogel catapult with engineered catapult, plants/fungi and animals capable ejection motion in nature. (b) Ashby plot of take-off velocity and power output comparison of the hydrogel catapult with animals and plants/fungi capable ejection motion in nature.

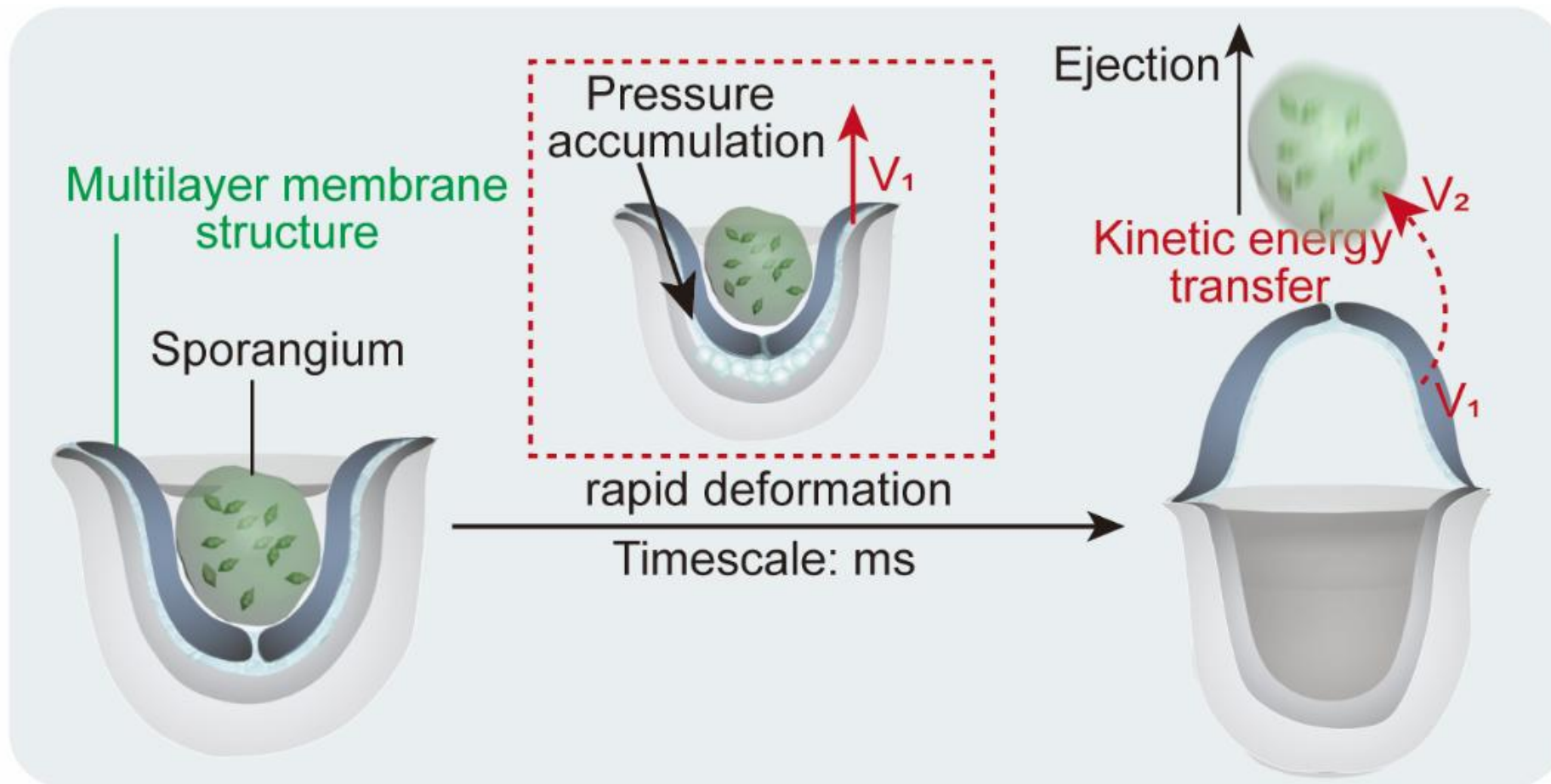

Supplementary Fig. 14. Schematic illustration of the structure and the sporangium dispersal mechanism of *Sphaerobolus*. The *Sphaerobolus* is a fungus with separable multilayer elastic membrane structure. The long-distance reproduction relies on the instantaneous eversion of the elastic exoperidium membrane, which is actuated by increased internal pressure during growth process to provide initial momentum, and the dynamic coupling with sporangium and substrate thus realizes the ejection motion.

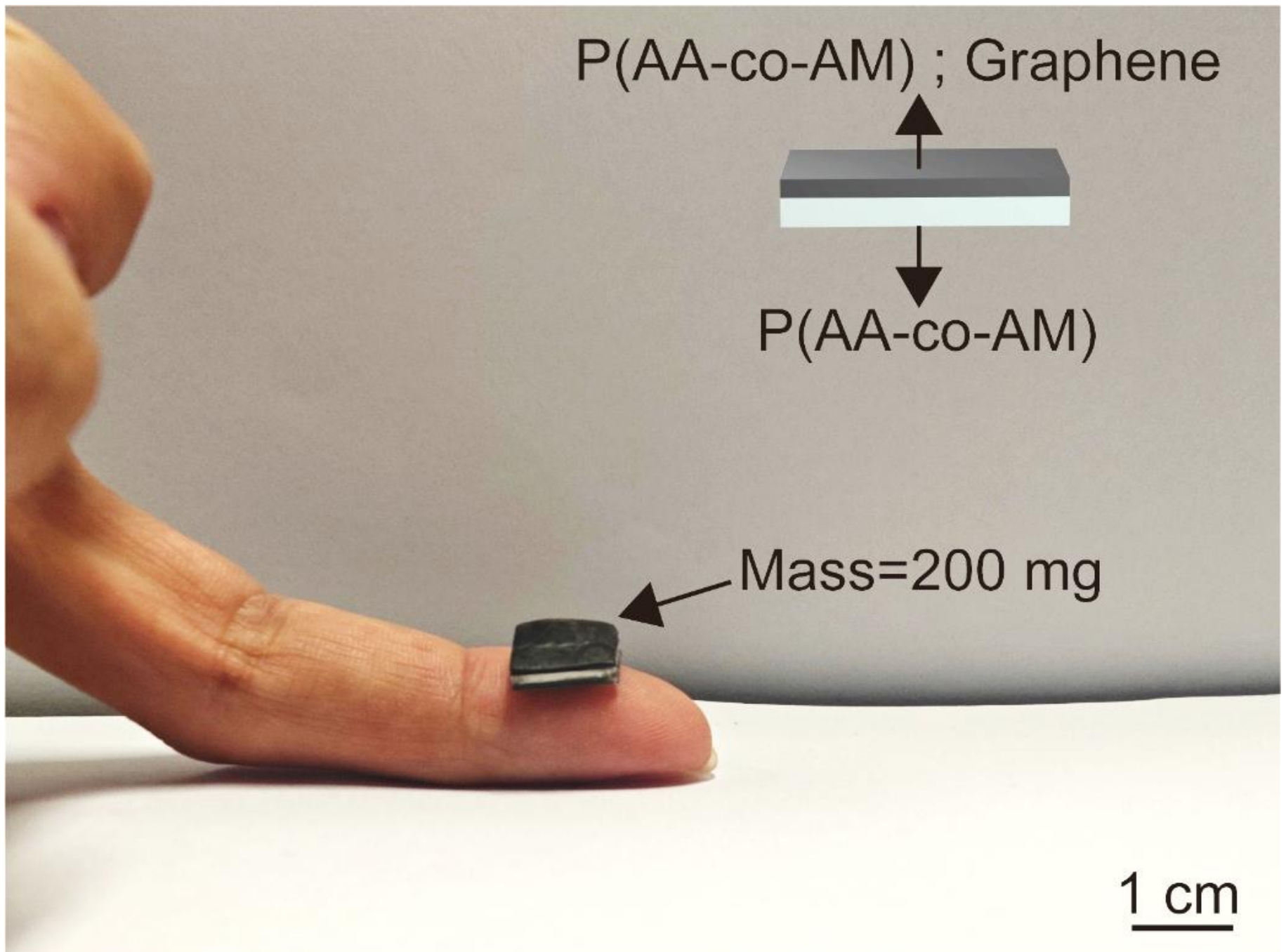

Supplementary Fig. 15. Photograph of the hydrogel catapult with the double layer structure and customizable size and shape.

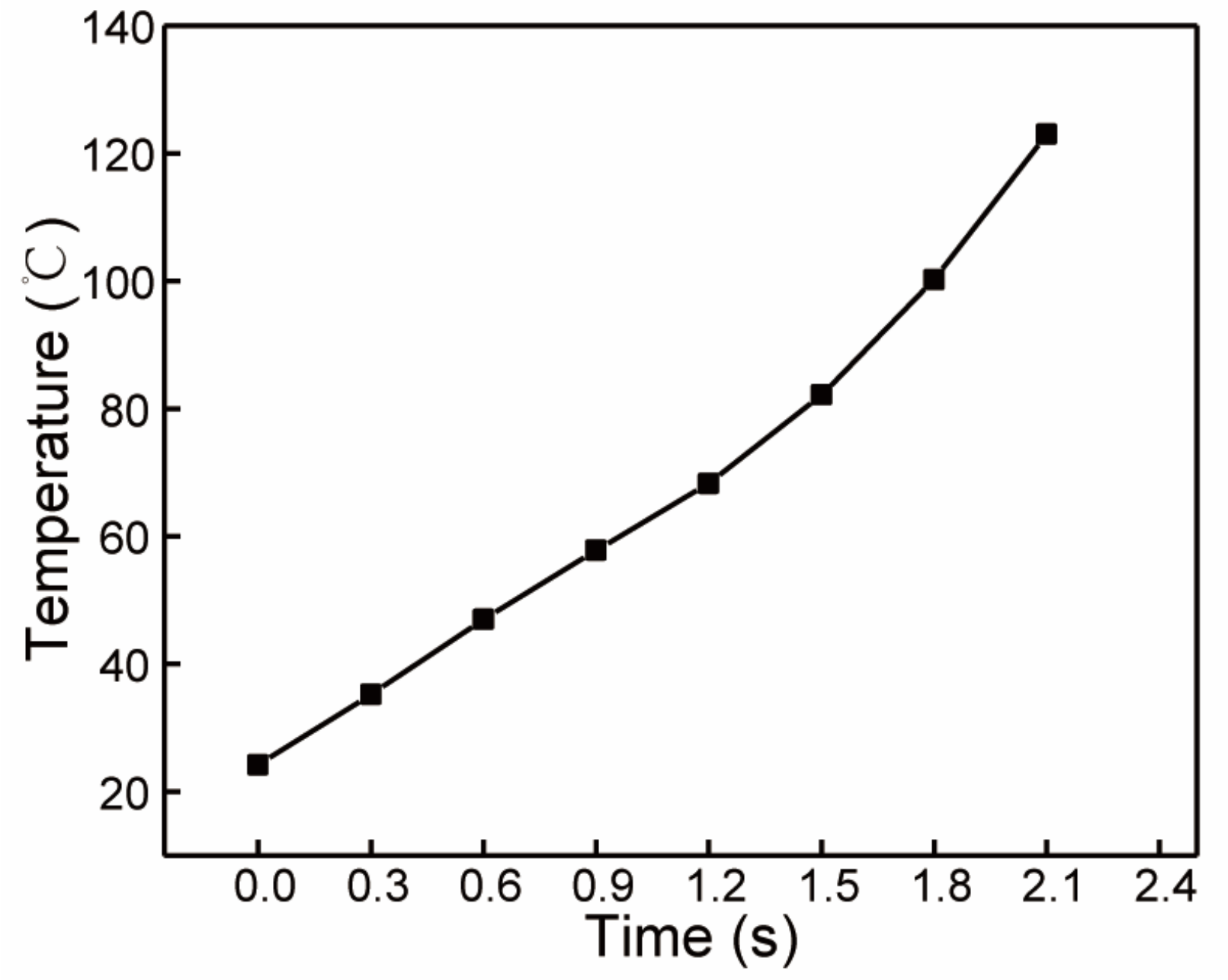

Supplementary Fig. 16. The temperature change of the hydrogel catapult irradiated with NIR light.

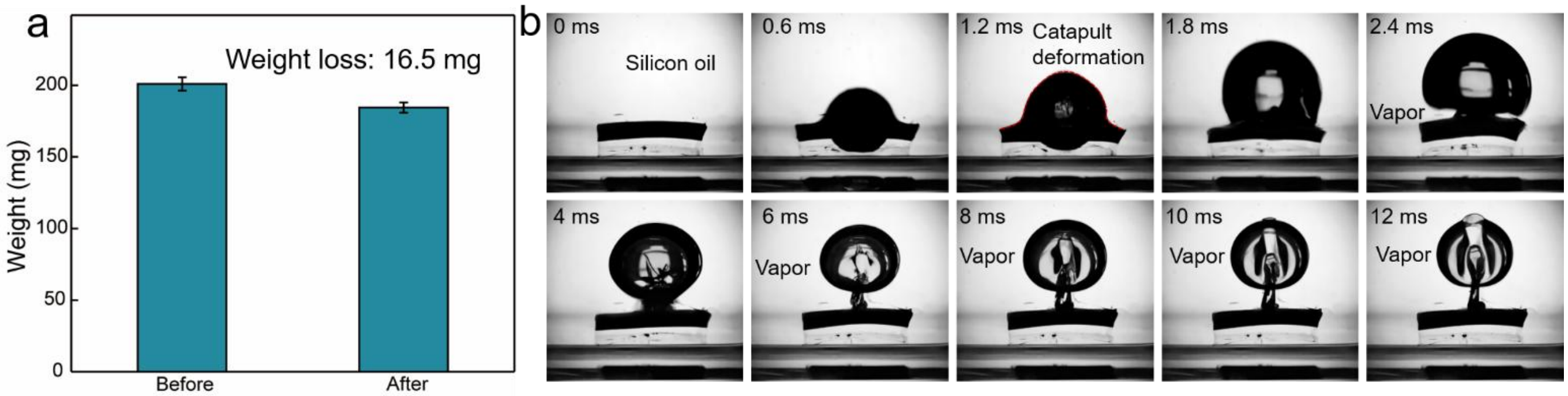

Supplementary Fig. 17. (a) The weight of the hydrogel catapult before and after the actuation. Data points are shown as mean ± s.d. (n = 3). (b) High speed camera image illustration the vapor jetting process of the hydrogel catapult in the silicon oil.

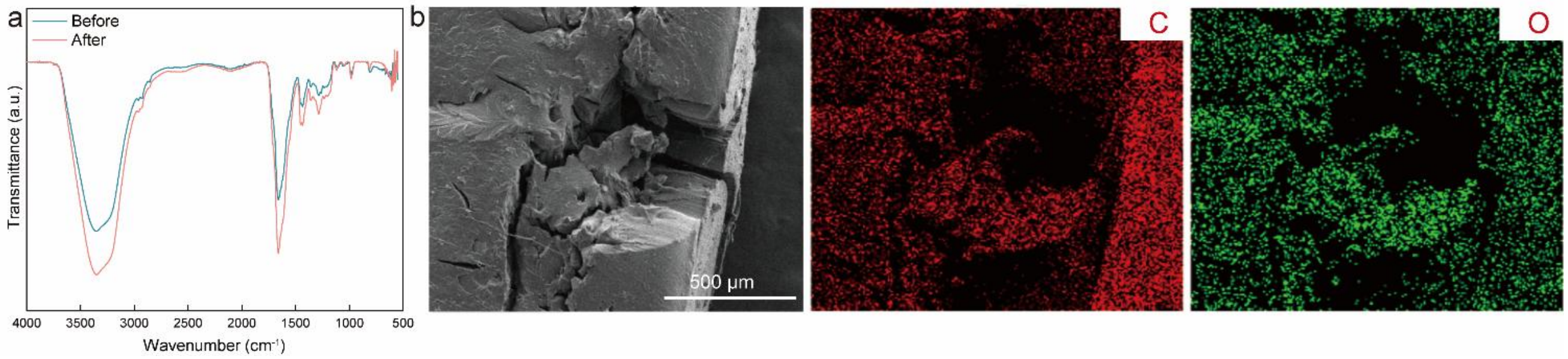

Supplementary Fig. 18. (a) The FTIR spectrum of the hydrogel catapult before and after the actuation. (b) The SEM and mapping images of the phase-transition region.

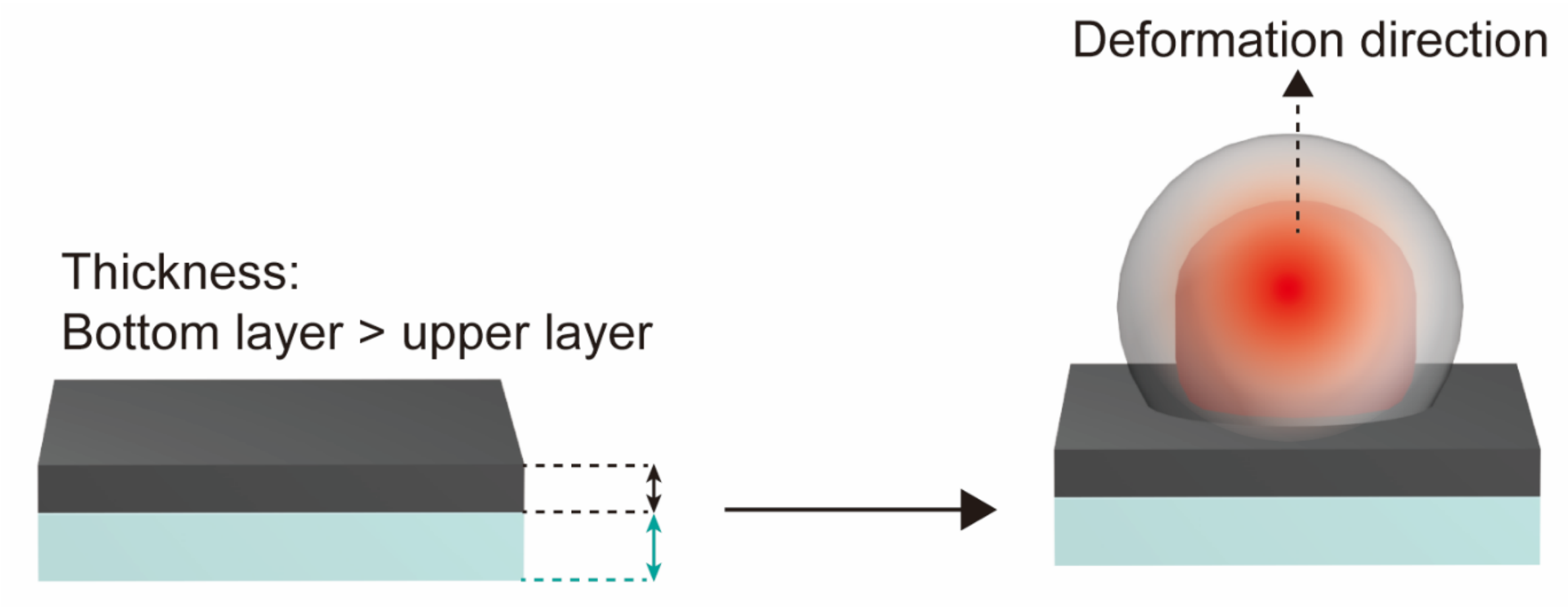

Supplementary Fig. 19. Schematic illustration of the thickness difference of the two layers to control the deformation direction towards to the projectile.

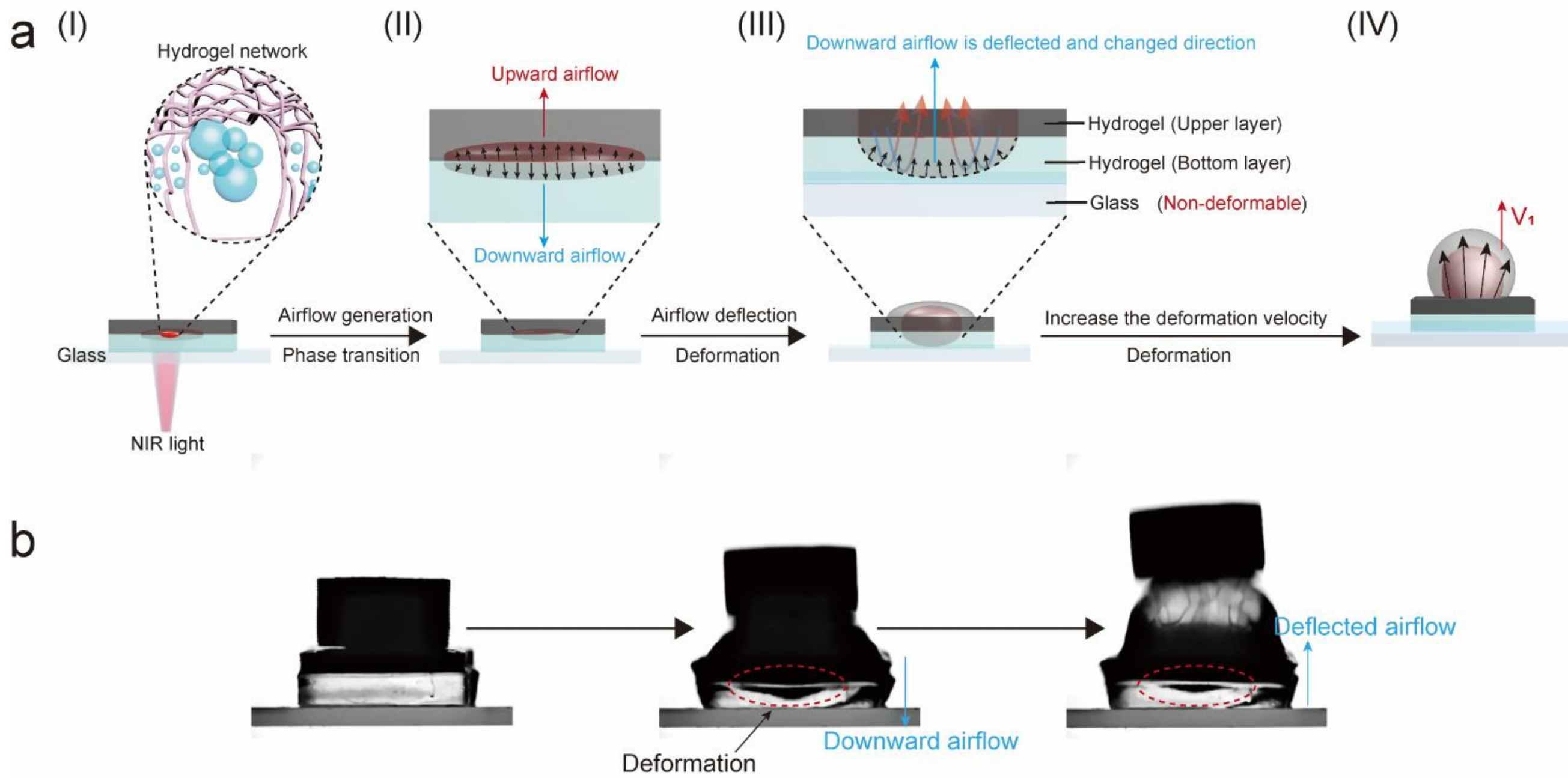

Supplementary Fig. 20. (a) Schematic illustration of the change inside the hydrogel during the deformation process. (b) The high-speed camera images showing the deformation process bottom layer inside the hydrogel catapult.

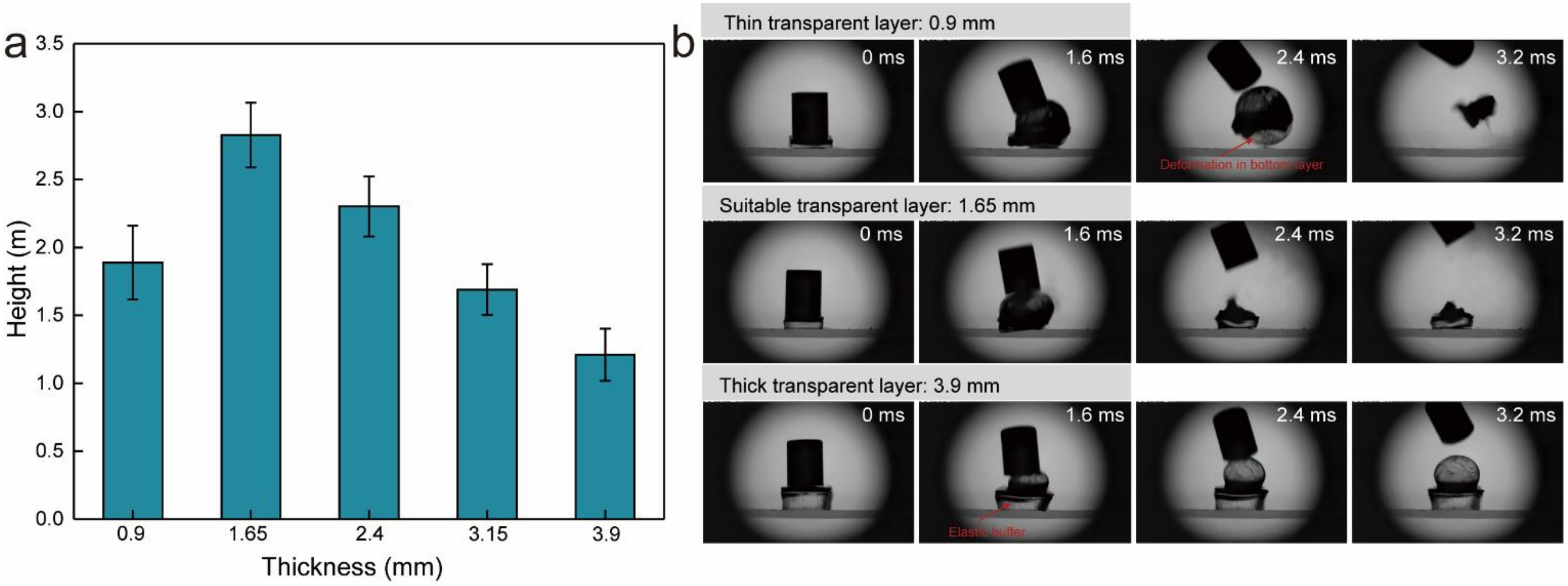

Supplementary Fig. 21. (a) The performance of catapults with different transparent layer thicknesses, data points are shown as mean ± s.d. (n = 4). Note that the thickness of graphene doped upper layer is 1.35 mm. The substrate is glass materials and the projectile's weight is 380 mg. (b) The representative high speed camera image of hydrogel catapult with thin, suitable and thick transparent layer.

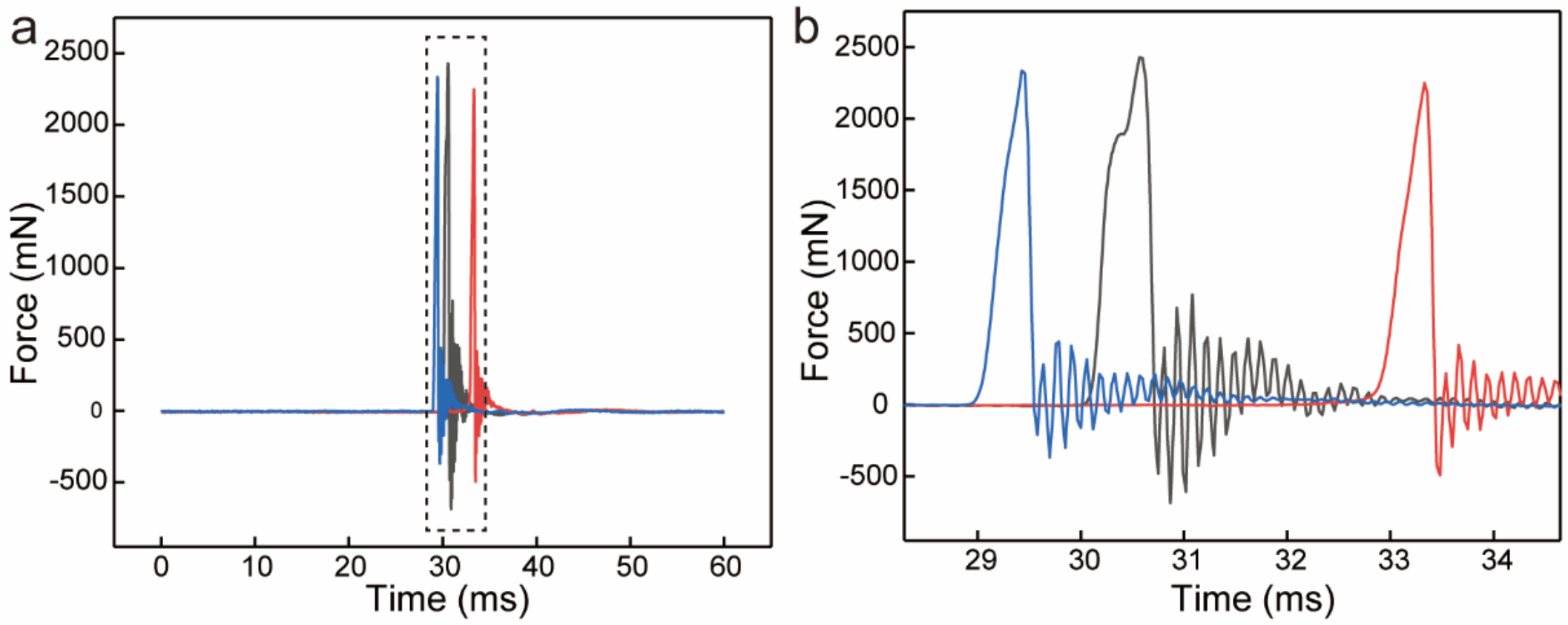

Supplementary Fig. 22. (a) The measured force output and (b) zoom in plot of the hydrogel catapult from three actuation tests. Note that the acquisition rate is 33.3 kHz of the sensor.

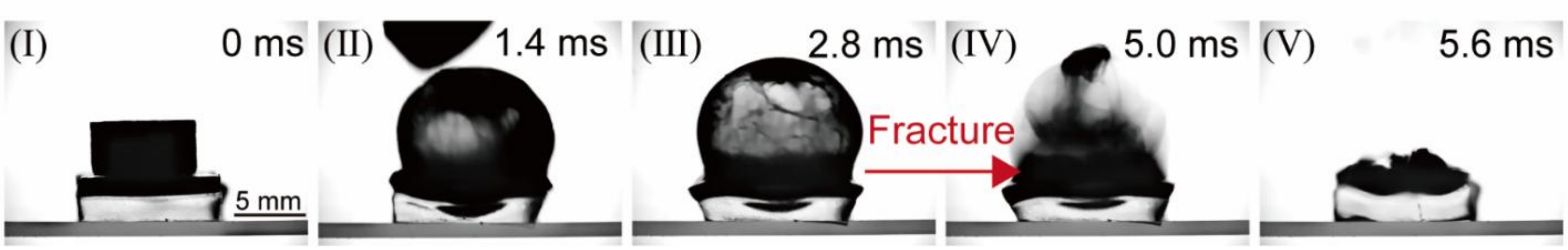

Supplementary Fig. 23. The high-speed camera images showing the deformation and fracture process of the light driven hydrogel catapult.

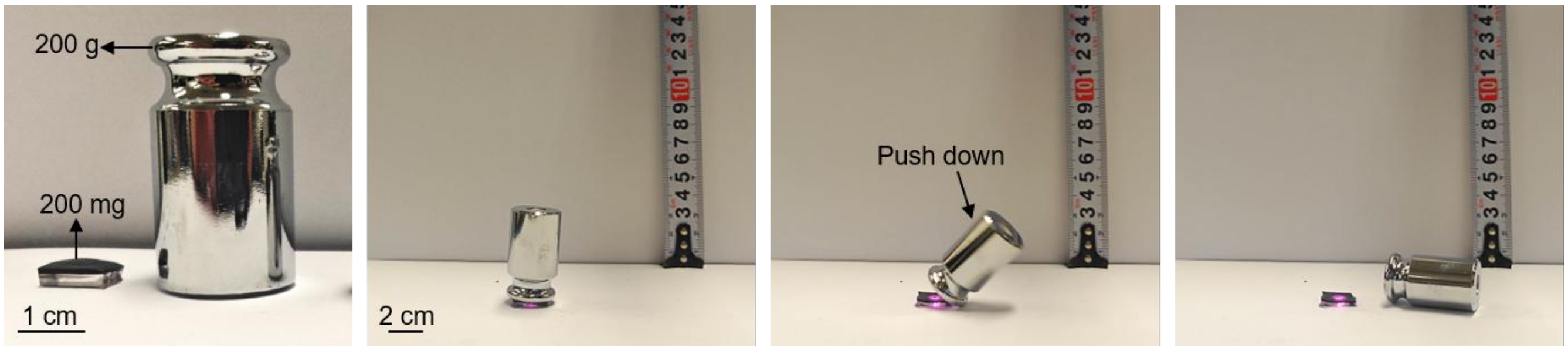

Supplementary Fig. 24. A serious of images illustrating a 200 mg hydrogel catapult topple a 200 g-weight (i.e., 1000 times the weight of hydrogel catapult).

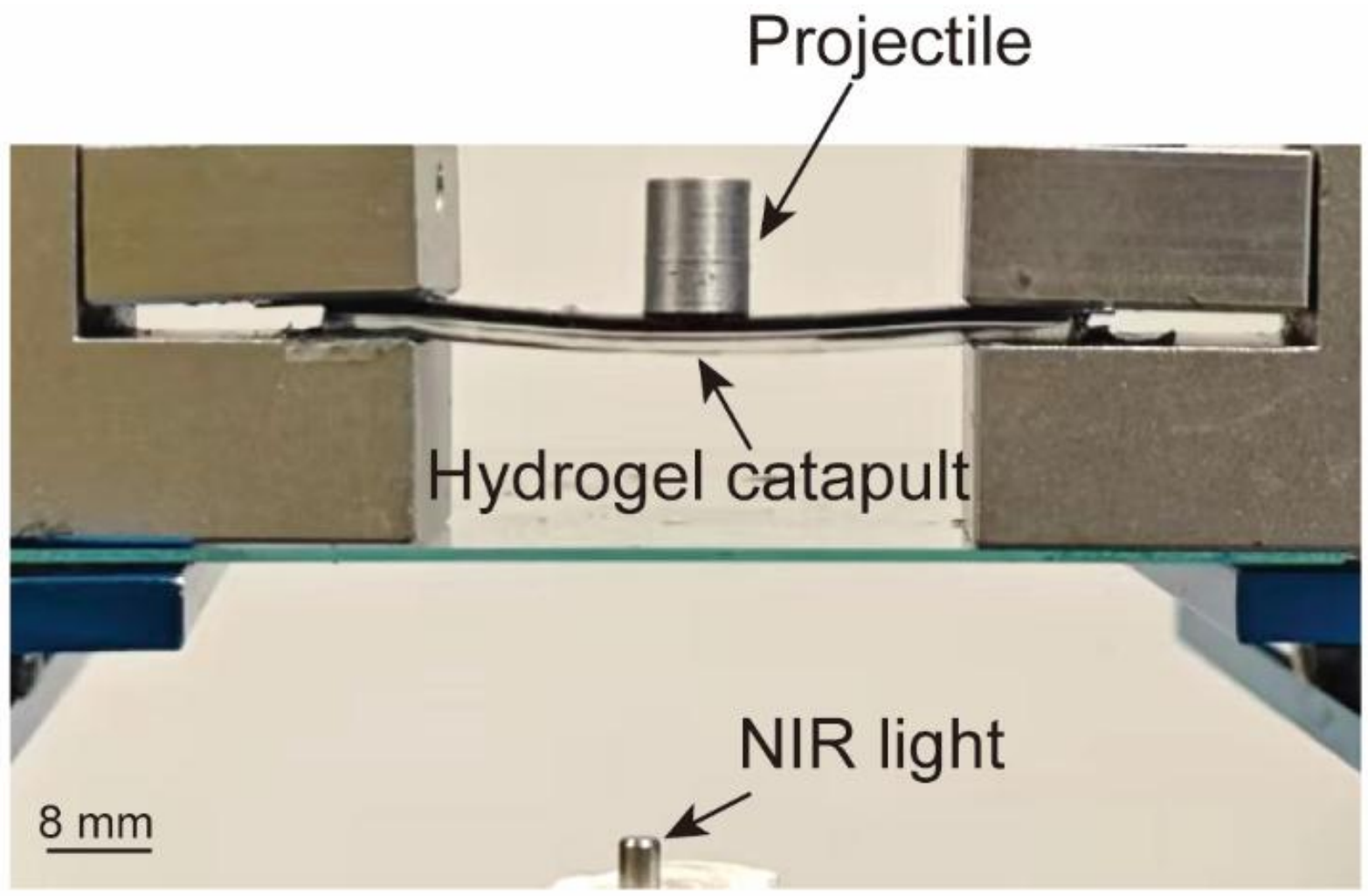

Supplementary Fig. 25. Experimental setup of the hydrogel catapult without substrate.

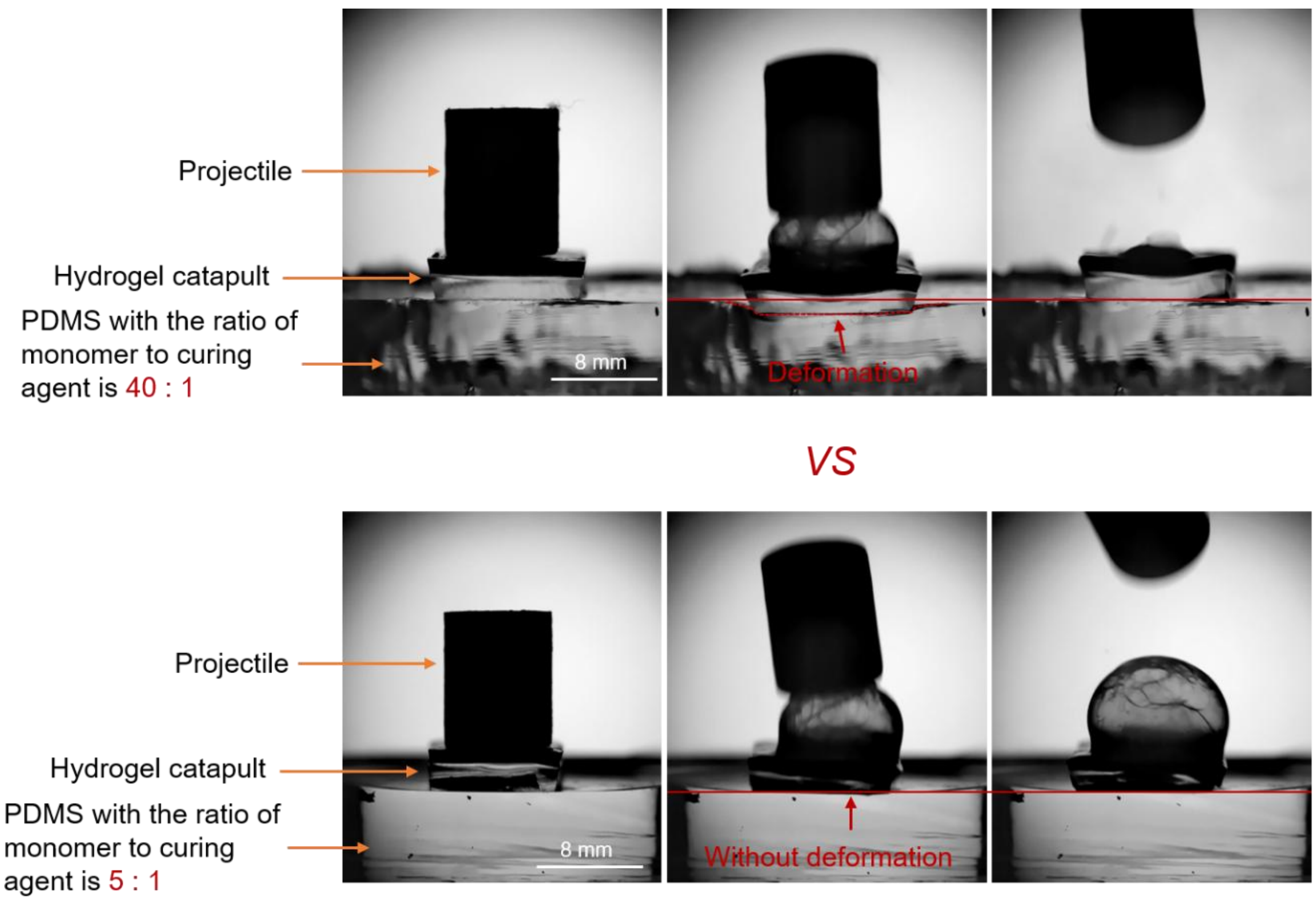

Supplementary Fig. 26. The comparison of the actuation process on the PDMS substrate with different ratio of monomer and curing agent (i.e., 40 :1 with 5 : 1).

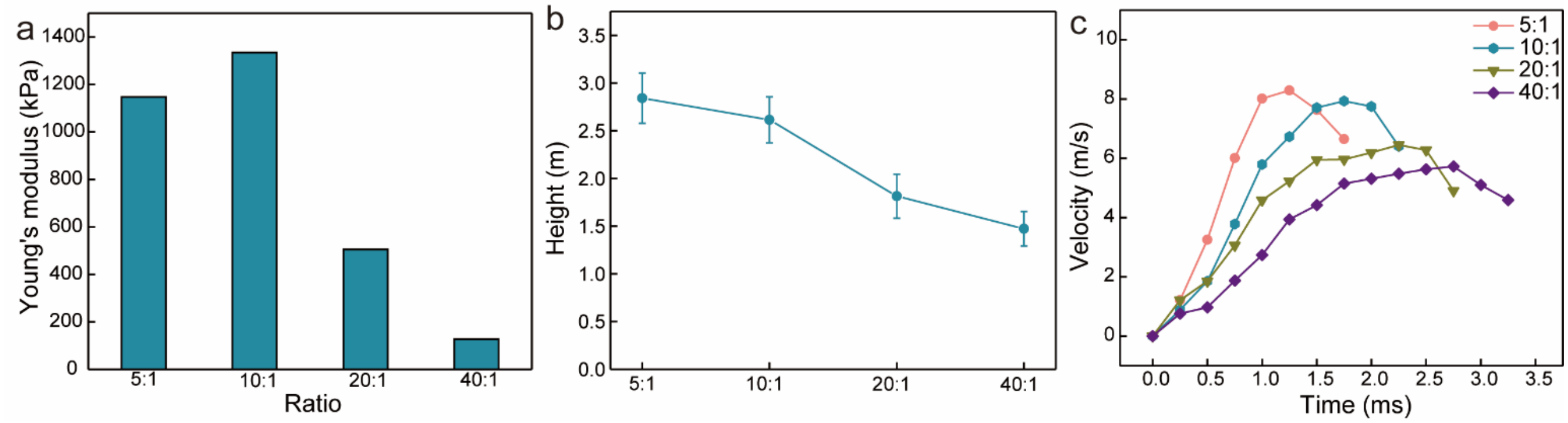

Supplementary Fig. 27. (a) The Young's modulus of the fabricated PDMS substrate with different ratio of the monomer and curing agent that obtained from stress-strain curve. Data points are shown as mean ± s.d. (n = 4). (b) The ejection height and (c) projectile velocity profiles during actuation on the substrate of PDMS with different ratio of the monomer and curing agent. Note that the mass of projectile is 380 mg.

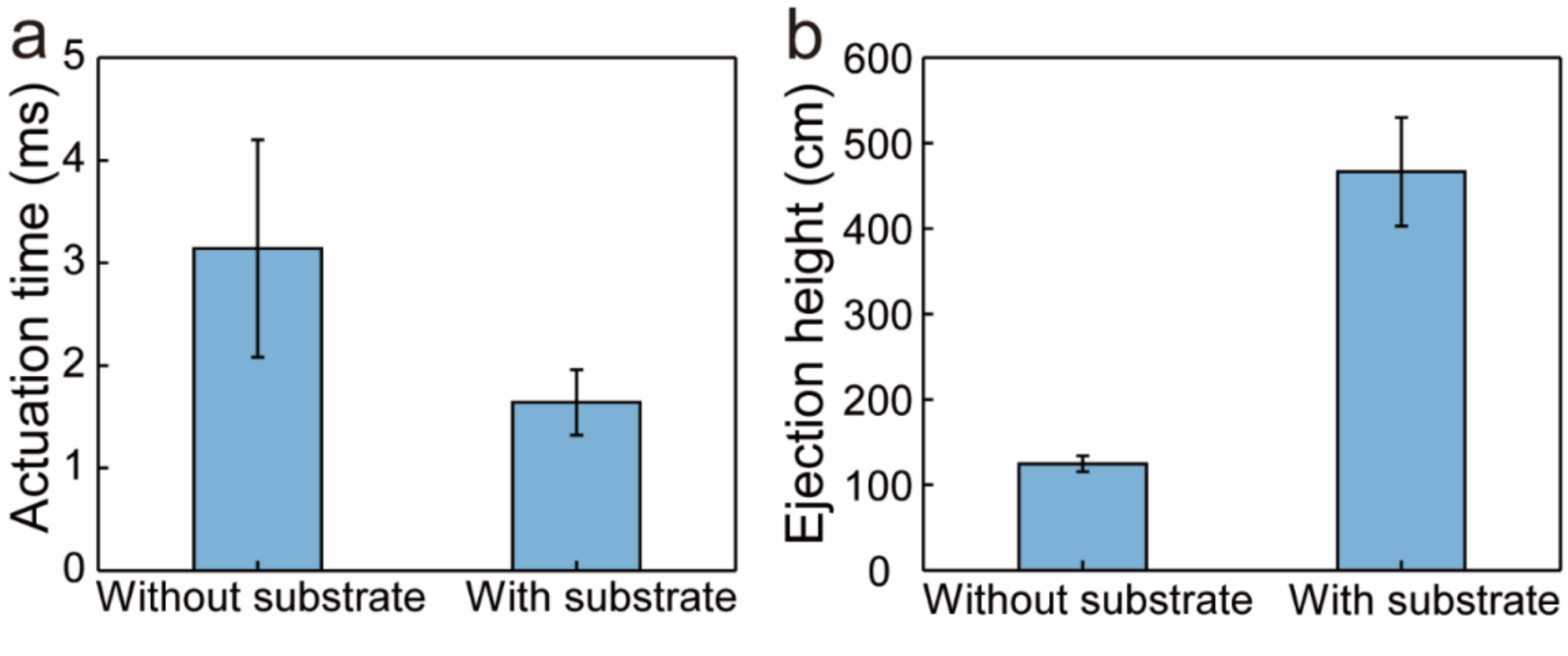

Supplementary Fig. 28. The comparison of (a) actuation time and (b) ejection height for hydrogel catapults on glass substrate versus free-standing configuration. Data points are shown as mean ± s.d. (n = 7).

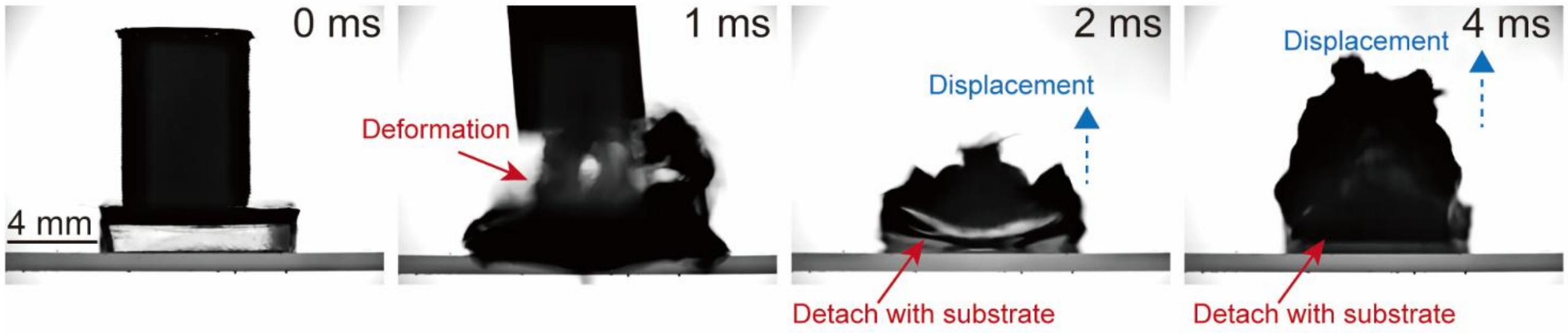

Supplementary Fig. 29. The high-speed camera images showing the actuation process of the hydrogel catapult in the absence of sufficient interfacial adhesion.

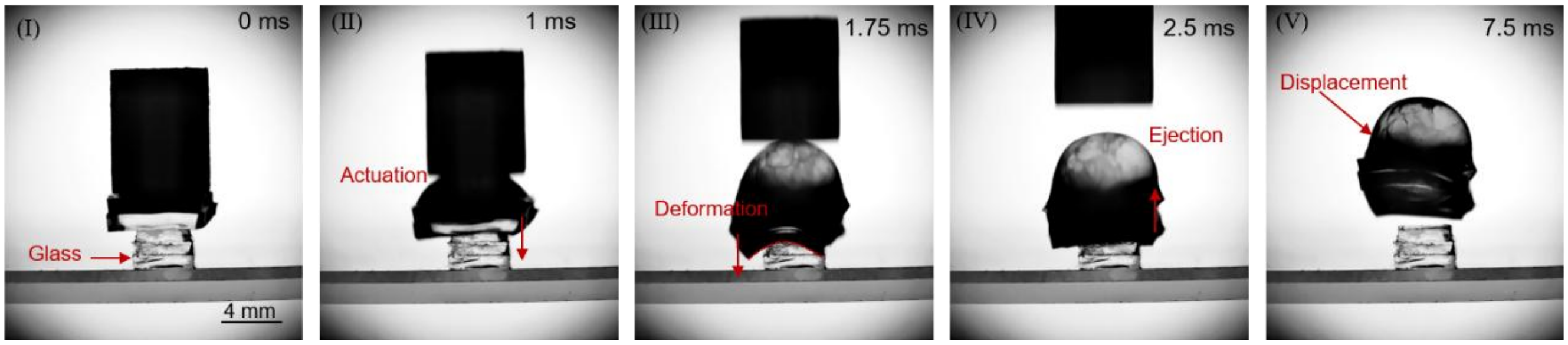

Supplementary Fig. 30. The high-speed images showing the actuation process from the glass substrate with small area (i.e., 0.4 cm × 0.4 cm).

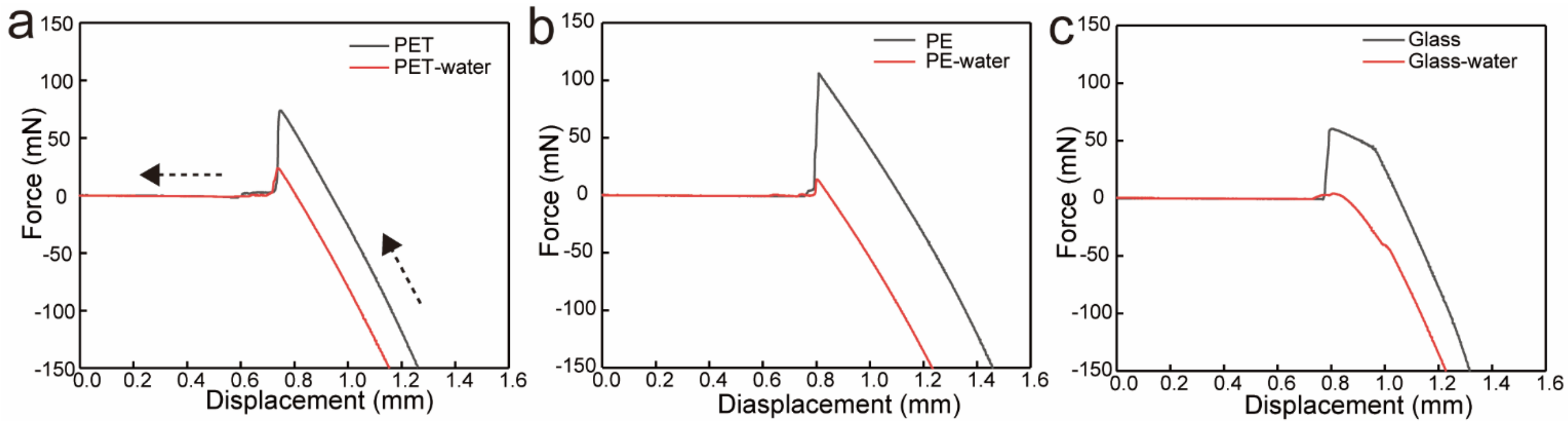

Supplementary Fig. 31. The adhesion force measurement of the hydrogel catapult on (a) PET, (b) PE and (c) glass surface in the presence and absence of water. Note that the cross section of the hydrogel catapult is 1 × 1 cm.

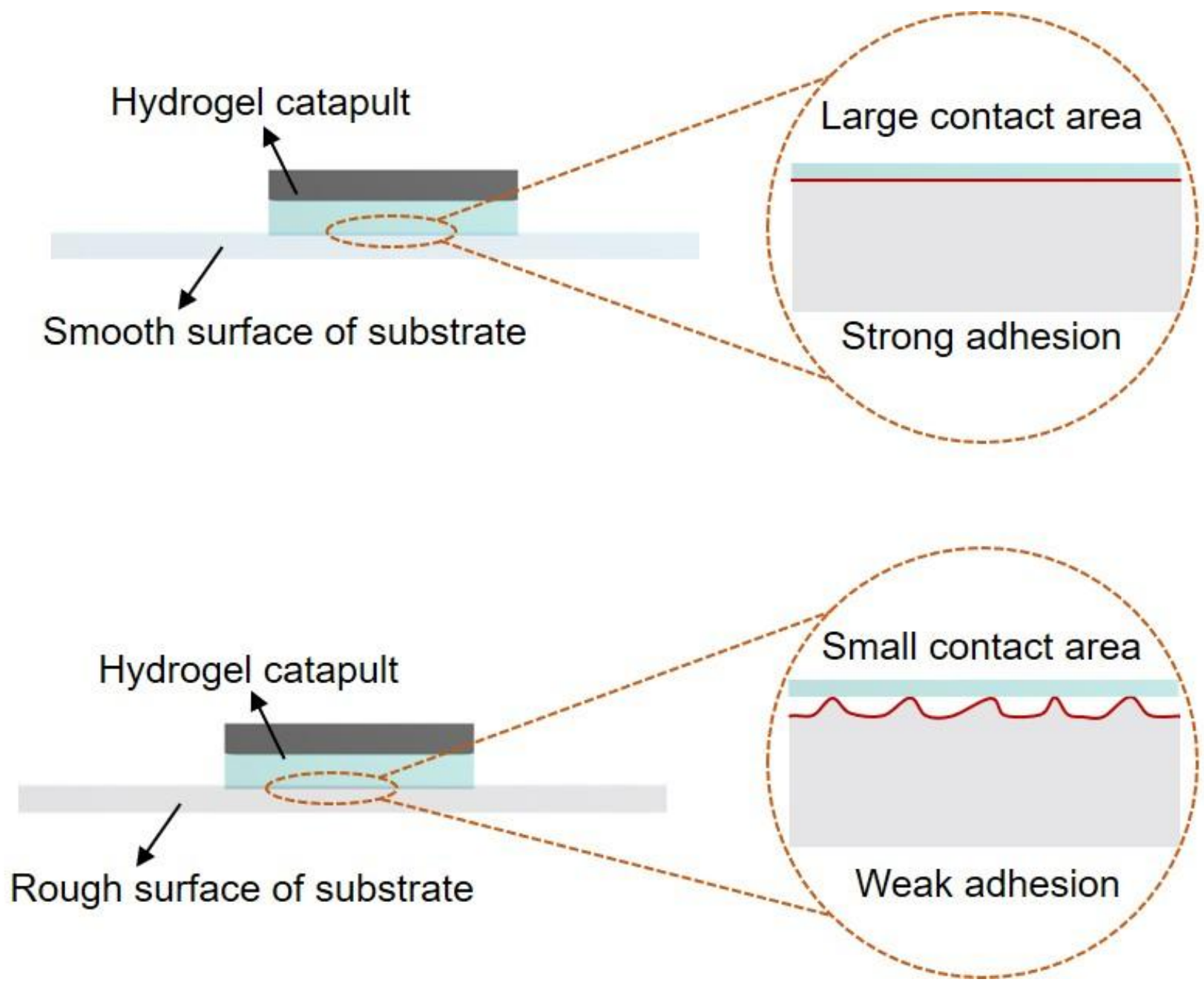

Supplementary Fig. 32. Schematic illustration of the adhesion force difference of the hydrogel catapult placed on the substrates with smooth surface or rough surface.

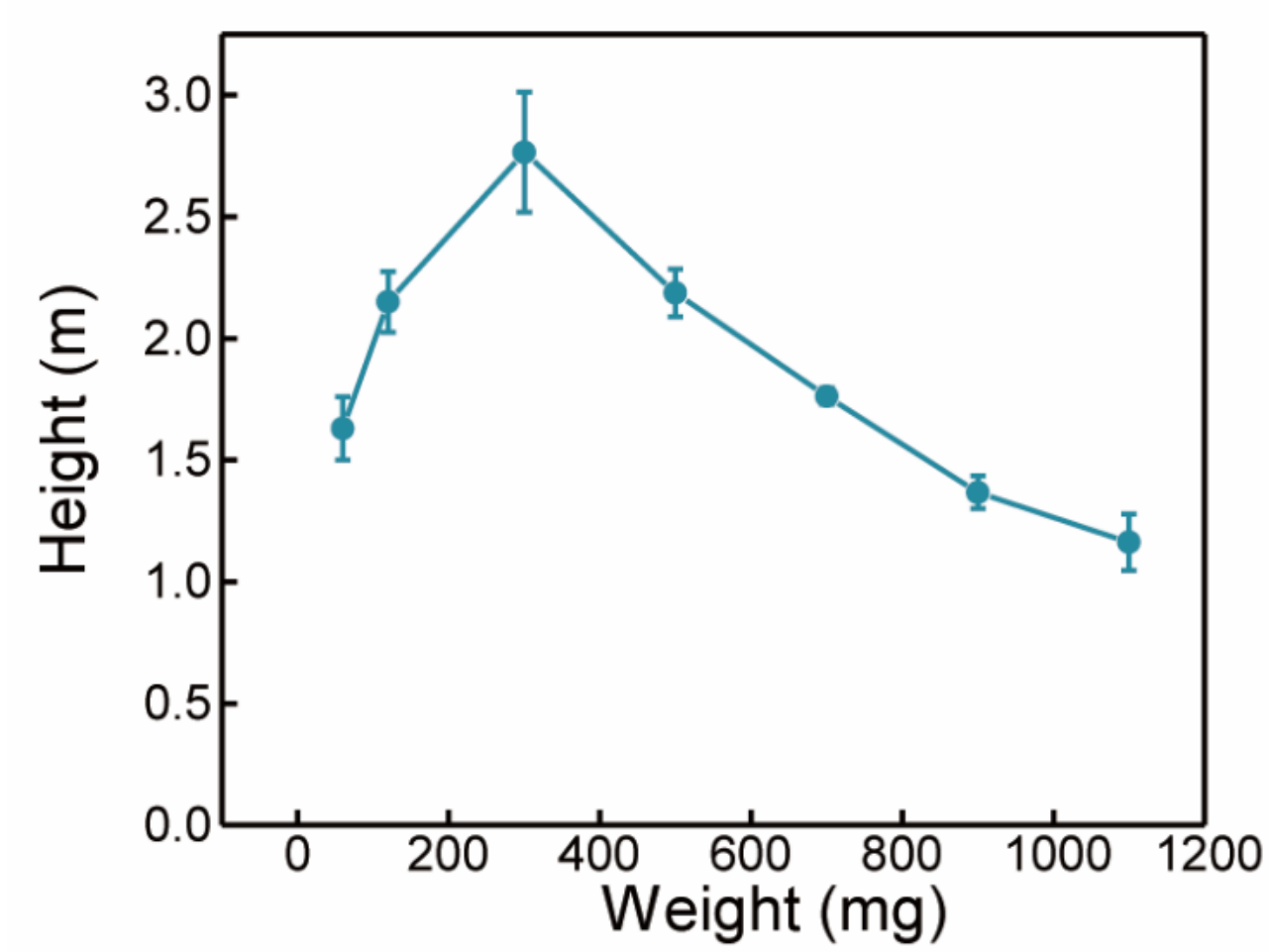

Supplementary Fig. 33. Ejection height of the projectiles with different weight. Note that all the projectiles are fabricated in cylindrical (diameter is 10 mm, height is 10 mm) with different density to reduce measurement errors. Data points are shown as mean ± s.d. (n = 4).

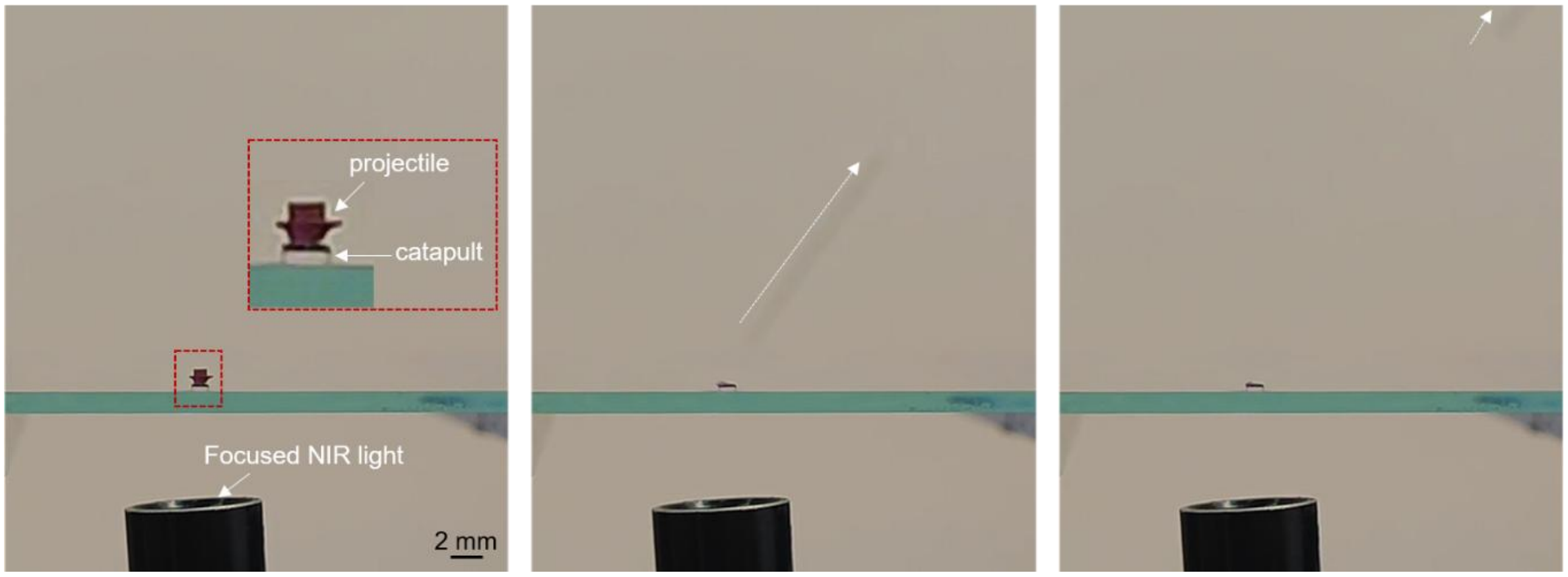

Supplementary Fig. 34. The screenshots showing the ejection process of the hydrogel catapult with dimension of approximately 1 × 1 × 0.8 mm.

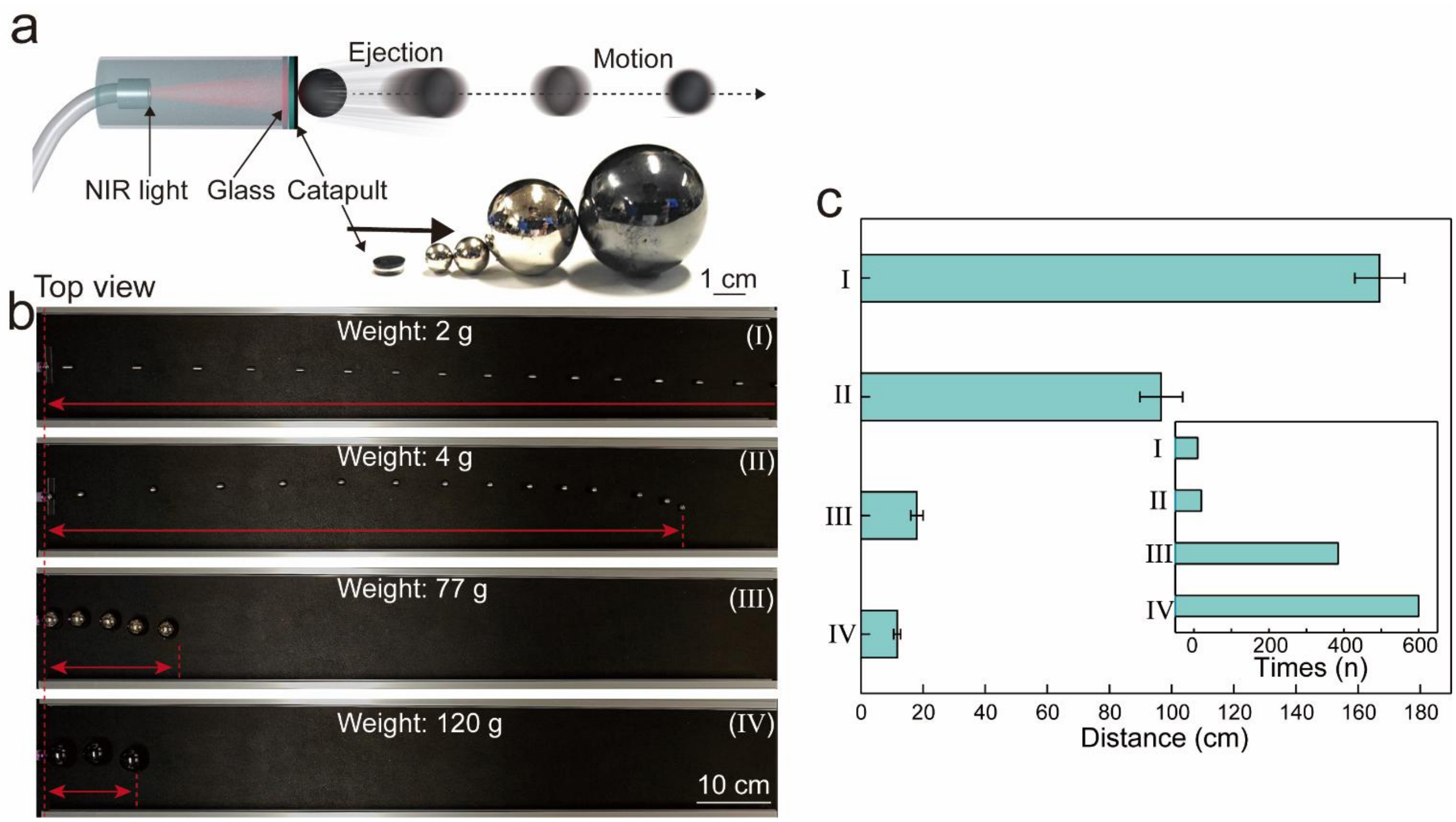

Supplementary Fig. 35. (a) Schematic illustration and (b) overlaid images obtained from Supplementary Movie 4 indicating the horizontal ejection process of the iron ball with different volume and mass. (c) The ejection distance of the iron balls with different volume and mass. Data points are shown as mean ± s.d. (n = 3). Inset: The comparison of the iron ball's mass relative to the hydrogel catapult weight (i.e., n = $m_{ball}/m_{catapult}$).

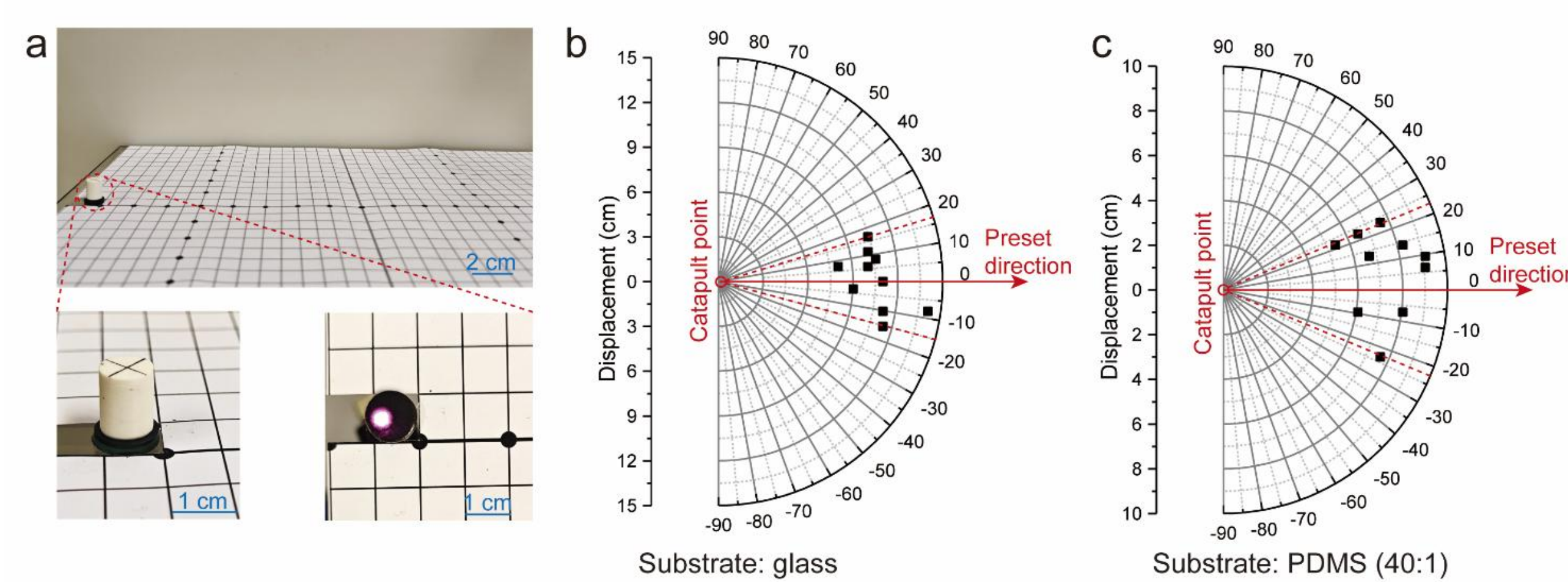

Supplementary Fig. 36. (a) The image showing the phase change region applied on the bottom surface of the projectile. (b) The angular dispersion and displacement of the 10 times actuation on the (b) glass substrate and (c) PDMS substrate (40: 1).

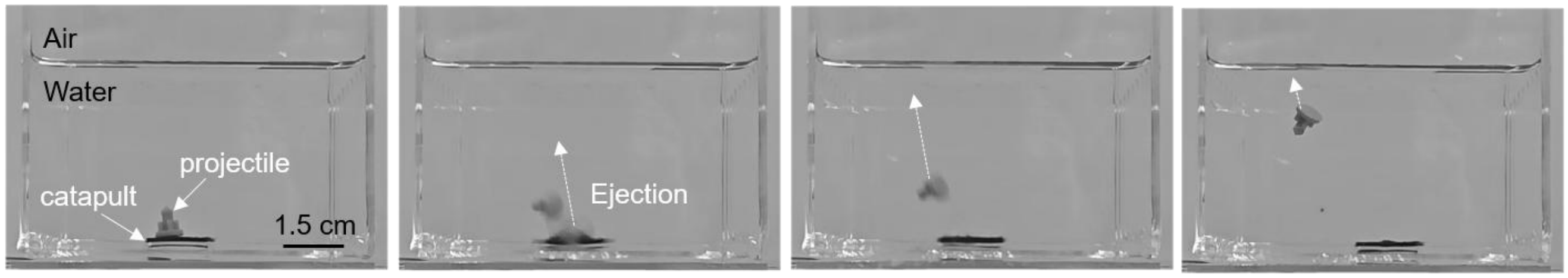

Supplementary Fig. 37. The screenshots showing the ejection process in an aquatic environment.

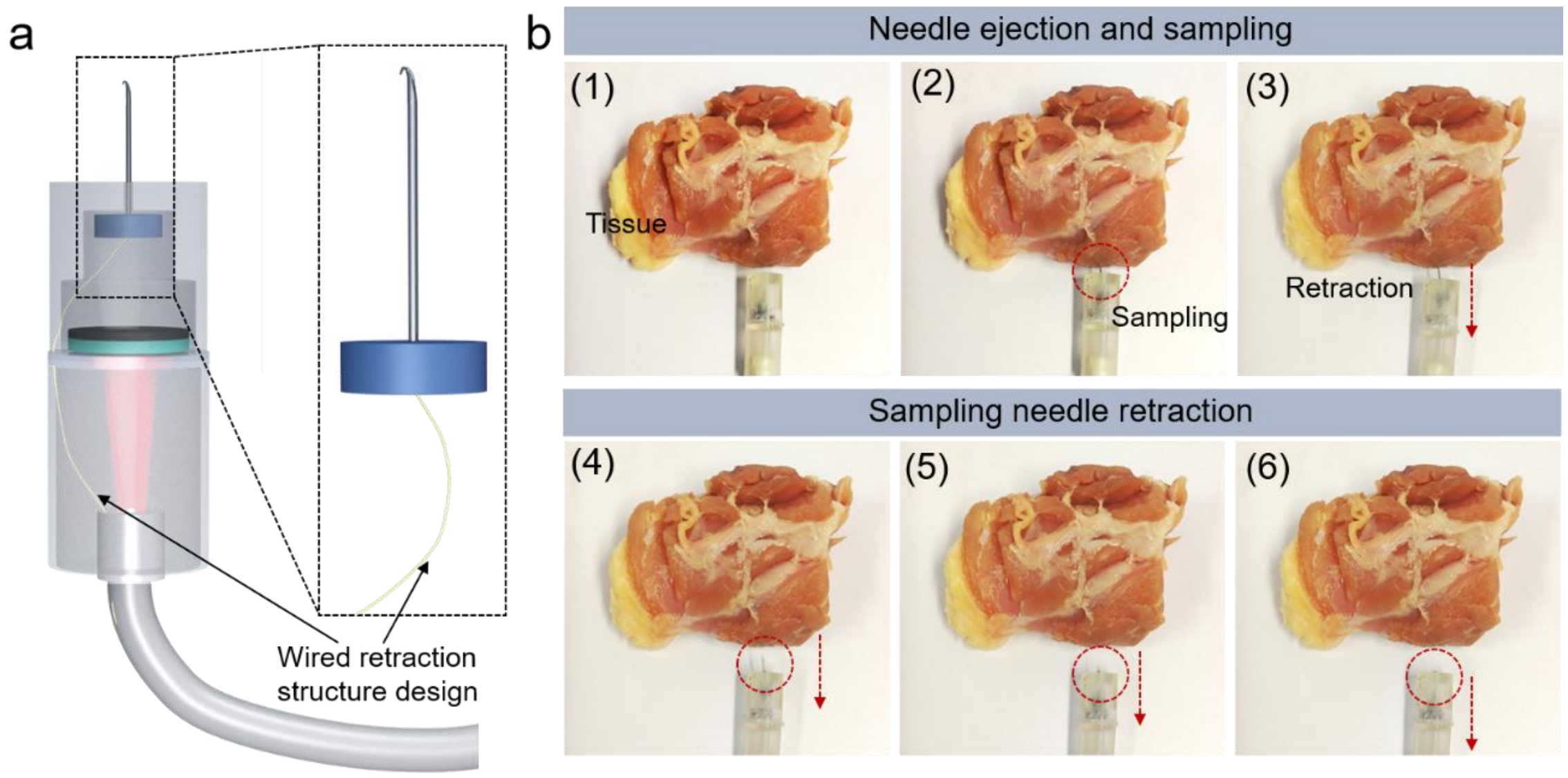

Supplementary Fig. 38. (a) The schematic showing the structure design for retraction of needle. (b) A serious of images obtained from Supplementary Movie 6 showing the sampling and retraction process of the developed biopsy device.

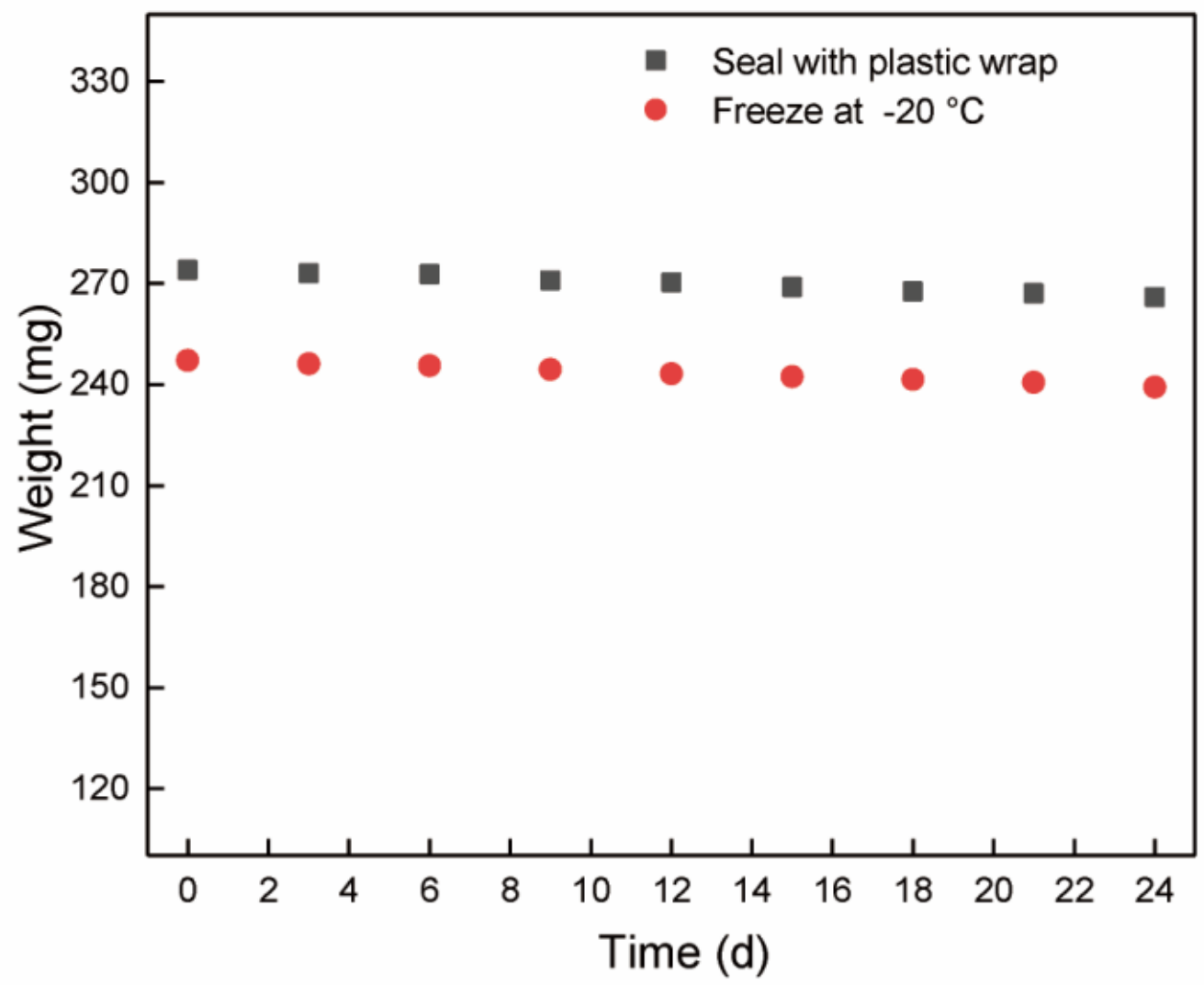

Supplementary Fig. 39. The weight change of the hydrogel during the long-time storage (i.e., wrapped with plastic wrap and frozen at -20 °C).

3. Supplementary Reference

1 Wang, J., Zhao, T. H., Fan, Y. Y., Wu, H. M. & Lv, J. A. Leveraging bioinspired structural constraints for tunable and programmable snapping dynamics in high-speed soft actuators. *Adv. Funct. Mater.* **33**, 2209798 (2022).

2 Lei, B., Wen, Z. Y., Wang, H. K., Gao, J. & Chen, L. J. Bioinspired jumping soft actuators of the liquid crystal elastomer enabled by photo-mechanical coupling. *ACS Appl. Mater. Interfaces* **16**, 1596-1604 (2024).

3 Lei, B., Wang, H., Wu, K., Gao, J. & Chen, L. Light-driven folding liquid crystal elastomer catapult with improved morphing velocity. *Adv. Intell. Syst.* **5**, 2200360 (2023).

4 Li, G. *et al.* Biomimetic 4D printing catapult: from biological prototype to practical implementation. *Adv. Funct. Mater.* **33**, 2301286 (2023).

5 Barré, C., Steinhardt, E. & Wood, R. J. The fastest microrobot pitcher: design exploration for a throwing microrobot. *IEEE Rob. Autom. Lett.* **8**, 4315-4321 (2023).

6 Sakes, A. *et al.* Shooting mechanisms in nature: a systematic review. *Plos One* **11**, 1-46 (2016).

7 Hayashi, M., Feilich, K. L. & Ellerby, D. J. The mechanics of explosive seed dispersal in orange jewelweed (Impatiens capensis). *J. Exp. Bot.* **60**, 2045-2053 (2009).

8 Deegan, R. D. Finessing the fracture energy barrier in ballistic seed dispersal. *Proc. Natl. Acad. Sci. U.S.A.* **109**, 5166-5169 (2012).

9 Hayashi, M., Gerry, S. P. & Ellerby, D. J. The seed dispersal catapult of Cardamine parviflora (Brassicaceae) is efficient but unreliable. *Am J Bot* **97**, 1595-1601 (2010).

10 Evangelista, D., Hotton, S. & Dumais, J. The mechanics of explosive dispersal and self-burial in the seeds of the filaree, Erodium cicutarium (Geraniaceae). *J. Exp. Biol.* **214**, 521-529 (2011).

11 Fischer, M. W. F., Stolze-Rybczynski, J. L., Cui, Y. L. & Money, N. P. How far and how fast can mushroom spores fly? Physical limits on ballistospore size and discharge distance in the Basidiomycota. *Fungal Biol.* **114**, 669-675 (2010).

12 Pringle, A., Patek, S. N., Fischer, M., Stolze, J. & Money, N. P. The captured launch of a ballistospore. *Mycologia* **97**, 866-871 (2005).

13 Yafetto, L. *et al.* The fastest flights in nature: high-speed spore discharge mechanisms among fungi. *Plos One* **3**, e3237 (2008).

14 de Groot, J. H. & van Leeuwen, J. L. Evidence for an elastic projection mechanism in the chameleon tongue. *P R Soc B Biol Sci.* **271**, 761-770 (2004).

15 Deban, S. M., O'Reilly, J. C., Dicke, U. & van Leeuwen, J. L. Extremely high-power tongue projection in plethodontid salamanders. *J Exp Biol* **210**, 655-667 (2007).

16 Anderson, C. V., Larghi, N. P. & Deban, S. M. Thermal effects on the performance, motor control and muscle dynamics of ballistic feeding in the salamander. *J Exp Biol* **217**, 3146-3158 (2014).

17 Monroy, J. A. & Nishikawa, K. C. Prey location, biomechanical constraints, and motor program choice during prey capture in the tomato frog, Dyscophus guineti. *J Comp Physiol A* **195**, 843-852 (2009).

18 Sandusky, P. E. & Deban, S. M. Temperature effects on the biomechanics of prey capture in the frog rana pipiens. *J Exp Zool Part A* **317a**, 595-607 (2012).

19 Anderson, C. V. Off like a shot: scaling of ballistic tongue projection reveals extremely high performance in small chameleons. *Sci. Rep.* **6** (2016).

20 Wang, X. *et al.* Fracture-driven power amplification in a hydrogel launcher. *Nat. Mater.* **23**, 1428-1435 (2024).

21 Jeon, J. *et al.* Continuous and programmable photomechanical jumping of polymer monoliths. *Mater Today* **49**, 97-106 (2021).

22 Ahn, C. Y., Liang, X. D. & Cai, S. Q. Bioinspired design of light-powered crawling, squeezing, and jumping untethered soft robot. *Adv. Mater. Technol.* **4**, 1900185 (2019).
23 Xiang, Y. Y. *et al.* Toward a multifunctional light-driven biomimetic mudskipper-like robot for various application scenarios. *ACS Appl. Mater. Interfaces* **14**, 20291-20302 (2022).
24 Li, M. T., Wang, X., Dong, B. & Sitti, M. In-air fast response and high speed jumping and rolling of a light-driven hydrogel actuator. *Nat. Commun.* **11**, 3988 (2020).
25 Li, H. & Wang, J. F. Ultrafast yet controllable dual-responsive all-carbon actuators for implementing unusual mechanical movements. *ACS Appl. Mater. Interfaces* **11**, 10218-10225 (2019).
26 Lee, H., Xia, C. & Fang, N. X. First jump of microgel; actuation speed enhancement by elastic instability. *Soft Matter* **6**, 4342 (2010).
27 Yu, K. Q. *et al.* Robust jumping actuator with a shrimp-shell architecture. *Adv. Mater.* **33**, 2104558 (2021).
28 Kim, Y., van den Berg, J. & Crosby, A. J. Autonomous snapping and jumping polymer gels. *Nat. Mater.* **20**, 1695-1701 (2021).
29 Hong, C. *et al.* Magnetically actuated gearbox for the wireless control of millimeter-scale robots. *Sci. Robot.* **7**, eabo4401 (2022).
30 Chen, R. *et al.* Legless soft robots capable of rapid, continuous, and steered jumping. *Nat. Commun.* **12**, 7028 (2021).
31 Hawkes, E. W. *et al.* Engineered jumpers overcome biological limits via work multiplication. *Nature* **604**, 657-661 (2022).
32 Chippada, U., Yurke, B. & Langrana, N. A. Simultaneous determination of Young's modulus, shear modulus, and Poisson's ratio of soft hydrogels. *J. Biomed. Mater. Res.* **25**, 545-555 (2010).
33 Koh, J. S., Jung, S. P., Wood, R. J. & Cho, K. J. A jumping robotic insect based on a torque reversal catapult mechanism. *In Proceedings of the 2013 IEEE/RSJ International Conference on Intelligent Robots and Systems*, 3796-3801 (2013).
34 Bennetclark, H. C. & Alder, G. M. Effect of air resistance on the jumping performance of insects. *J. Exp. Biol.* **82**, 105-121 (1979).
35 Okamura, A.M., Simone, C. & O'Leary, M.D. Force modeling for needle insertion into soft tissue. *IEEE Trans. Biomed. Eng.* **51**, 1707-1716 (2004).
36 Wani, S., Muthusamy, V.R. & Komanduri, S. EUS-guided tissue acquisition: an evidence-based approach (with videos). *Gastrointest Endosc* **80**, 939-959 (2014).
37 Hébert-Magee, S. et al. The presence of a cytopathologist increases the diagnostic accuracy of endoscopic ultrasound-guided fine needle aspiration cytology for pancreatic adenocarcinoma: a meta-analysis. *Cytopathology* **24**, 159-171 (2013).
38 Parker, S.H. et al. Percutaneous large-core breast biopsy: a multi-institutional study. *Radiology* **193**, 359-364 (1994).
39 Son, D., Gilbert, H. & Sitti, M. Magnetically actuated soft capsule endoscope for fine-needle biopsy. *Soft Robot* **7**, 10-21 (2020).
40 Yarmolenko, P.S. et al. Thresholds for thermal damage to normal tissues: An update. *Int J Hyperther* **27**, 320-343 (2011).
41 Mahvash, M. & Dupont, P.E. Mechanics of Dynamic Needle Insertion into a Biological Material. *IEEE Trans. Biomed. Eng.* **57**, 934-943 (2010).
42 Abolhassani, N., Patel, R. & Moallem, M. Needle insertion into soft tissue: A survey. *Med Eng Phys* **29**, 413-431 (2007).
