# Supplementary material for: Dynamic coupling enables impulsive ejection in a hydrogel catapult
Source: Nat Commun. 2026 Sep 7;17:10568. doi: 10.1038/s41467-026-77391-6 (PMC13642336; doi:10.1038/s41467-026-77391-6)
Supplement: Supplementary file 3 [file 41467_2026_77391_MOESM2_ESM.pdf]

Description of Additional Supplementary Files

Supplementary Movie 1. The ultrafast deformation actuation process of the hydrogel catapult recorded by a high-speed camera.

Supplementary Movie 2. The ejection height of the hydrogel catapult: > 6.15 m for projectile with mass of 203 mg (similar with the weight of hydrogel catapult (200 mg)) and ~ 10 cm for projectile with mass of 10 g (50 times the weight of hydrogel catapult).

Supplementary Movie 3. The comparison of the deformation and response time for hydrogel catapults on glass substrate, PDMS substrate and free standing (without spubstrate). Among others, the hydrogel catapult actuated from the glass substrate showed the largest deformation and fastest response speed, proving that the rigid substrate helps improve the ejection performance and momentum generation and transfer.

Supplementary Movie 4. The wide applicability of hydrogel catapult for projectiles with different weight due to the ultrafast deformation and significant force output, we have demonstrated the ejection motion of representative lightweight projectiles of powder and heavier projectiles of coin, the hydrogel catapult also can be used as actuator to actuate the system (burette with water) for jumping. In addition, we demonstrated the horizontal direction ejection motion of iron balls with different volumes and masses. The iron balls with different masses are ejected at different speeds and distances, the smaller the mass of the ball, the faster and longer distance it rolls.

Supplementary Movie 5. The directional ejection motion can be realized by tailoring the position where the outputted force applied on the projectile. Through constructing the asymmetric interaction, we have demonstrated the left, backward, right, and forward directional ejection motion with similar launching height and travel distance.

Supplementary Movie 6. The penetration process into the agarose gel and real tissue of hydrogel catapult actuated biopsy robot. The needle can be pushed into the gel and tissue with designed depth based on the significant force output of hydrogel catapult. The needle recovery after puncture can be achieved through tethered traction.

Supplementary Movie 7. Endoscopically assisted gastric sampling process. Combined with the real-time observation of the imaging, we demonstrated the light driven biopsy system can be controlled to reach to the target area inside the pig’s stomach and followed deep tissue sampling.
